# Identification of Candidate Olfactory Genes in the Antennal Transcriptome of *Loxostege sticticalis* Trapped by Three Different Sex Pheromone Blends

**DOI:** 10.3390/insects16020152

**Published:** 2025-02-03

**Authors:** Mengke Zhang, Sumei Zhao, Zhiping Xue, Jiaying Sun, Jiangning Hao, Fengzhi Deng, Junxia Huang, Caroline Du, Yongjun Du

**Affiliations:** 1 Institute of Pesticides and Environmental Toxicology, Zhejiang University, Hangzhou 310058, China; zmk15737027673@163.com; 2 Ispxtech Inc., Hangzhou 310018, China; carolined@ispxtech.com; 3 Inner Mongolia Autonomous Region Plant Protection and Quarantine Center, Hohhot 010010, China; smzhaohh@126.com (S.Z.); sssunjy@163.com (J.S.); hao1120585385@163.com (J.H.); dengfengzhi321@163.com (F.D.); elainewang1118@163.com (J.H.); 4 Baotou Agricultural and Animal Husbandry Science Research Institute, Baotou 014030, China; xzp532@163.com

**Keywords:** *Loxostege sticticalis*, olfactory system, olfactory genes, antennal transcriptome

## Abstract

Insects sense intraspecific or interspecific information about the chemical substances in the habitat through the sensitive olfactory system to carry out foraging, mating, oviposition, and other activities. The antennae serve as the primary olfactory organs in insects. The olfactory process involves the participation of many proteins, such as odorant-binding proteins (OBPs) and odorant receptors (ORs), but ORs play a central role in olfactory specificity and sensitivity. The beet webworm, *Loxostege sticticalis,* is an omnivorous agricultural pest that endangers crops and poses a significant risk to the agricultural and animal husbandry production in northern China. In this study, Illumina sequencing was conducted on the antennal transcriptome of male *L. sticticalis* trapped by three different sex pheromone blends. A total of 10,320 DEGs were identified, from which 46 candidate olfactory genes were selected for further analysis. These candidate olfactory genes comprise 13 odorant receptors, 6 ionotropic receptors (IRs), 3 gustatory receptors (GRs), 12 odorant-binding proteins, and 13 chemosensory proteins (CSPs). In summary, we analyzed the antennal transcriptome of male *L. sticticalis* trapped by three different sex pheromone blends and identified several candidate olfactory genes. This discovery offers a foundation for further molecular-level investigations into the olfactory system of *L. sticticalis*.

## Simple Summary

*Loxostege sticticalis* is a highly destructive and migratory agriculture pest that feeds on key crops, such as soybeans, corn, and potatoes during its larval stage, causing significant damage to crops. Insects rely on antennal expression receptors to detect olfactory signals and perform essential tasks like finding hosts, attracting mates, and selecting oviposition sites. However, there are few reports on olfactory-related genes in *L. sticticalis*. The olfactory system plays a central role in the perception of the external environment and the detection of chemical signals released by females. This process involves the synergy of multiple proteins. In this study, the antennal transcriptomes of male *L. sticticalis* trapped by three different sex pheromone blends were sequenced and characterized. In total, 10,320 differentially expressed genes (DEGs) were identified through transcriptome sequencing and bioinformatics analysis. Furthermore, five gene families that are crucial to insect olfaction were identified among the DEGs, including odorant receptors (ORs), ionotropic receptors (IRs), gustatory receptors (GRs), odorant-binding proteins (OBPs), and chemosensory proteins (CSPs). These results provide a molecular foundation for the further study of the chemical acceptance mechanisms involved in host recognition, localization, and oviposition preference.

## 1. Introduction

Lepidopteran insects possess a highly accurate and sensitive olfactory system that enables them to detect and discriminate diverse environmental odors with remarkable sensitivity and specificity [1,2]. This capability is essential for locating habitats and food sources, identifying mating partners and suitable oviposition sites, and evading natural enemies. The antenna surface is distributed with sensilla, specialized hair-like and multi-pore structures, and there are several types. The olfactory receptor neurons (ORNs) are distributed in the root of the sensilla [3] of insect antennae, constituting the fundamental unit of olfaction. Several olfactory proteins have been identified in ORNs, including odorant-binding proteins (OBPs), chemosensory proteins (CSPs), odorant receptors (ORs), ionotropic receptors (IRs), sensory neuron membrane proteins (SNMPs), and gustatory receptors (GRs) [4,5]. They play a crucial role in the detection, recognition, and degradation of peripheral sex pheromones [6,7].

As a rule, insect ORs can be categorized into two types: one is the conventional ligand-binding OR with diversity, and the other is the highly conserved non-conventional odorant receptor co-receptor (Orco) [8,9,10,11]. In Lepidoptera, the ORs that detect sex pheromone components, known as pheromone receptors (PRs), exhibit relatively high amino acid sequence conservation and play a crucial role in odor recognition [12]. OBPs are considered the first proteins involved in the olfactory signal transduction procedure, mainly expressed in the antennae of both sexes of insects [13,14]. Based on the specific odorants that they bind and transport, OBPs can be further classified into pheromone-binding proteins (PBPs), general odorant-binding proteins (GOBPs), antenna-binding proteins (ABPs), and other types [15,16]. The two subfamilies of OBPs, GOBPs, and PBPs are responsible for the transfer of host plant volatiles and pheromones to ORs and ensure that they are protected from degradation by odorant-degrading enzymes (ODEs) [17,18]. In addition to OBPs, there are other types of soluble proteins, namely CSPs, in the secretions of the sensillum lymph [19]. In insect olfactory perception, hydrophobic odor molecules enter the sensillum lymph through micropores in the sensillum wall. Within the lymph, the OBPs or CSPs bind to these odorants and transport them to receptors located on the dendritic membranes of ORNs, including ORs and IRs [20]. Afterward, the chemical signals are transduced into electrical signals and transmitted via axons to the antennal lobe (AL) through the axons. These signals will be processed and integrated through the downstream multi-level neural pathways and ultimately mediate the behavior of insects [7]. In addition, SNMPs, another protein family of the dendritic membrane, also play a key role in odor perception [21].

The beet webworm, *Loxostege stictialis* L. (Lepidoptera: Pyralidae), is an important migratory pest endangering agriculture and animal husbandry production in northern China [22,23]. It exhibits characteristics of binge eating and periodic outbreaks [24]. Since 1949, there have been three obvious outbreak cycles in China. In a year of its major occurrence, local crop yield losses in China can reach up to 60%, potentially resulting in complete crop failure. This poses a significant threat to the production security of agriculture and animal husbandry, particularly for grain, oil, and forage crops [23]. It has been reported that *L. sticticalis* prefers specific crops, such as beets, legumes, alfalfa, corn, vegetables [25,26,27], etc. This phenomenon may be related to its highly developed olfactory system [28,29].

In Lepidopterans, the olfactory function of insects depends on their unique antennal organ. Researchers have explored many olfactory genes through antennal transcriptome analysis for some moths [30,31]. However, there is almost no transcriptome study on the antennae of male adults of *L. sticticalis* trapped by different types of three different sex pheromone blends, X, Y, and Z, and its olfactory gene database seems to be incomplete. The identification and analysis of olfactory genes and the further elucidation of the olfactory mechanism will be of great significance for the design of olfaction-based prevention and control technologies for *L. sticticalis*.

In this study, we employed Illumina sequencing technology to conduct the first comprehensive transcriptomic analysis of the antennae of male adults of *L. sticticalis* trapped by three different sex pheromone blends. We identified all odorant receptors among the DEGs. Our findings encompass sequencing, gene annotation, GO annotation, and specifically, the identification of 13 ORs, 5 IRs, 3 GRs, 12 OBPs, and 13 CSPs. These results provide a critical foundation for elucidating the olfactory molecular mechanisms of *L. sticticalis*.

## 2. Materials and Methods

### 2.1. Insects and Quality Control

The *L. sticticalis* used in this study was trapped in Dashimen Town, Keshiketeng Banner, Inner Mongolia Autonomous Region (43.13° N 117.22° E), China, in July 2024. In the afternoon of the previous day, three different sex pheromone blends of X (E11-14:Ac 600 ug/lure and E11-14:OH 54 ug/lure), Y (E11-16:Ac 600 ug/lure), and Z (14:Ac 600 ug/lure), purchased from Ningbo Newcom Biotechnology Co., Ltd., Ningbo, China, were put into the lures and placed in the field, respectively. The male insects were collected in the morning of the next day. The antennae of individual males were collected with tweezers and immediately froze in liquid nitrogen. Three biological replicates were prepared for each sample, and the antennae of 30 male individuals were collected for each biological replicate. Trizol reagent (TAKARA, Beijing, China) was used to extract total RNA from the collected antennae according to the manufacturer’s protocol. The purity and integrity of total RNA were evaluated by a NanoDrop One spectrophotometer (Thermo Fisher Scientific, Waltham, MA, USA) and agarose gel electrophoresis, respectively.

### 2.2. cDNA Library Construction and Sequencing

The cDNA library construction and Illumina sequencing of our RNA samples were performed at Nohe Technology Co., Ltd., in Beijing, China. First, Oligo (dT) magnetic beads were used to enrich mRNA. After fragmentation, the first strand of cDNA was synthesized using random hexamer primers. Then, the second strand of cDNA was synthesized using dUTP. After amplification and purification, AMPure XP beads were used to screen the appropriate library fragments to create a cDNA library. Agilent 2100 bioanalyzer (Agilent, Santa Clara, CA, USA) was used to detect the insert size of the library, and the different libraries were pooled according to the effective concentration and the target offline data volume. Finally, samples of different antennae were sequenced using Illumina HiSeq^TM^ 2500 (Illumina, San Diego, CA, USA).

### 2.3. Assembly and Functional Annotation

Clean data (clean reads) were obtained by filtering out a small number of reads with sequencing joints or low sequencing quality from the original data. The clean reads were spliced and assembled using Trinity v2.15.1 software [32], and the spliced transcript sequence information was stored in FASTA format. Then, the transcripts were clustered using the Corset website [33] to obtain the longest cluster sequence for subsequent analysis. Using NCBI BLASTx (https://blast.ncbi.nlm.nih.gov/Blast.cgi?PROGRAM=blastx&PAGE_TYPE=BlastSearch&LINK_LOC=blasthome, accessed on 9 July 2024), the unigenes were annotated for the comprehensive information of the non-redundant (nr) database and the SwissProt protein sequence library with an e-value < 1 × 10^−5^. Subsequently, the BLAST results were imported into the Blast2GO system [34] for gene ontology (GO) annotation. The unigene sequences were further analyzed using the Eukaryotic Orthologous Groups (KOGs) database to predict and classify their functional attributes. Based on the results of BLAST analysis, the protein-coding region was predicted using the OrfPredictor (https://www.ncbi.nlm.nih.gov/orffinder/, accessed on 9 July 2024) [35].

### 2.4. Gene Expression Levels and Differential Expression Analysis

RSEM v1.2.8 software [36] was used to statistically analyze the comparison results of bowtie. Considering the influence of sequencing depth and gene length on the fragment count, the number of fragments per million mapped fragments (FPKMs) from a gene per thousand base length was used to estimate the gene expression level. Then, DEGseq2 software [37] was used to analyze the significance of gene expression differences. Genes with fold changes greater than 2 (|log2FoldChange| ≥ 1) and *p*-values less than 0.05 (padj < 0.5) were identified as DEGs.

### 2.5. Sequence Analysis

The open reading frames (ORFs) of the candidate olfactory genes in *L. sticticalis* were predicted using ORF Finder (http://www.ncbi.nlm.nih.gov/gorf/gorf.html, accessed on 18 September 2024). NCBI-BLASTp (http://blast.ncbi.nlm.nih.gov/, accessed on 18 September 2024) was used for a similarity search. SignalP 5.0 [36] (https://services.healthtech.dtu.dk/services/SignalP-5.0/, accessed on 18 September 2024) and TMHMM 2.0 (https://services.healthtech.dtu.dk/services/TMHMM-2.0/, accessed on 18 September 2024) were used to predict the signal peptides and transmembrane domains (TMDs) of the candidate olfactory genes. The nucleotide sequences of all identified olfactory genes are provided in Supplementary Table S4.

### 2.6. Phylogenetic Analyses

The amino acid sequences of the candidate olfactory genes of *L. sticticalis* were aligned using ClustalX 2.0 software. The MEGA 7.0 software [38] was used to construct phylogenetic trees based on the maximum likelihood method [39], with a bootstrap value of 1000. The chemosensory gene sequences of other insects were retrieved from the NCBI website (https://www.ncbi.nlm.nih.gov/, accessed on 20 September 2024), and the amino acid sequences were in Supplementary Table S5.

## 3. Results

### 3.1. Transcriptome Overview

**Figure 1:**
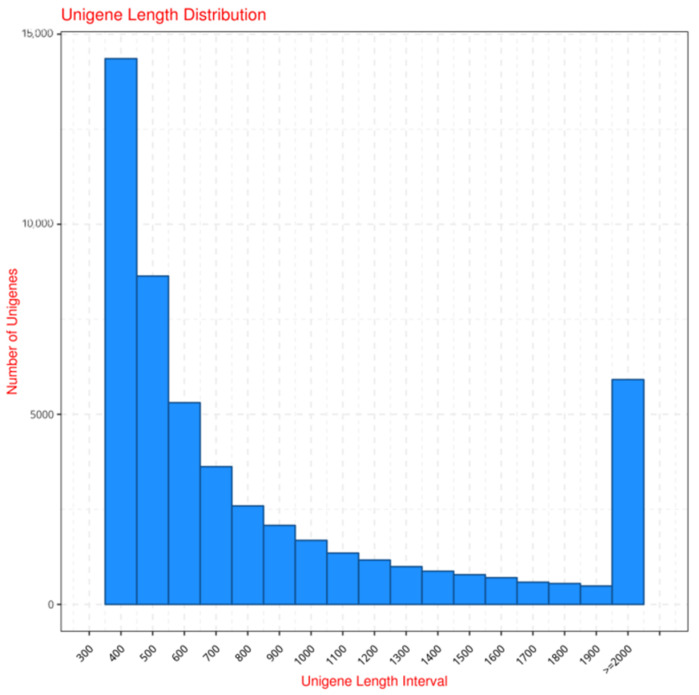
The size distribution of the assembled unigenes by antennal transcriptome of *L. sticticalis* trapped by three different sex pheromone blends.

To further understand the antennal transcriptome of male *L. sticticalis* trapped by three different sex pheromone blends, the cDNA library we constructed was subjected to next-generation sequencing, and 245.7 million clean reads (24.57 Gb) were obtained. The content of Q30 bases in all samples was greater than 93.01%. A total of 150,454 transcripts were assembled, with a mean length of 880 bp and an N50 length of 1238 bp, and 62,885 unigenes were identified, with a mean length of 894 bp and an N50 length of 1270 bp. Among these unigenes, 32,598 (51.84%) were longer than 500 bp, and 14,270 (22.69%) were longer than 1 kb (Figure 1 and Table 1). We compared our transcriptome assembly quality with the published antennal transcriptome data of Lepidopteran insects, specifically focusing on *Chilo suppressalis* (66,560 unigenes, mean length of 761 bp, N50 of 1271 bp) [20] and *Ostrinia furnacalis* (37,687 unigenes, mean length of 818 bp, N50 of 1022 bp) [40] in the Pyralidae and Crambidae species. The results show that our assembly quality not only meets the standard but also outperforms most of the similar transcriptomes.

**Figure 2:**
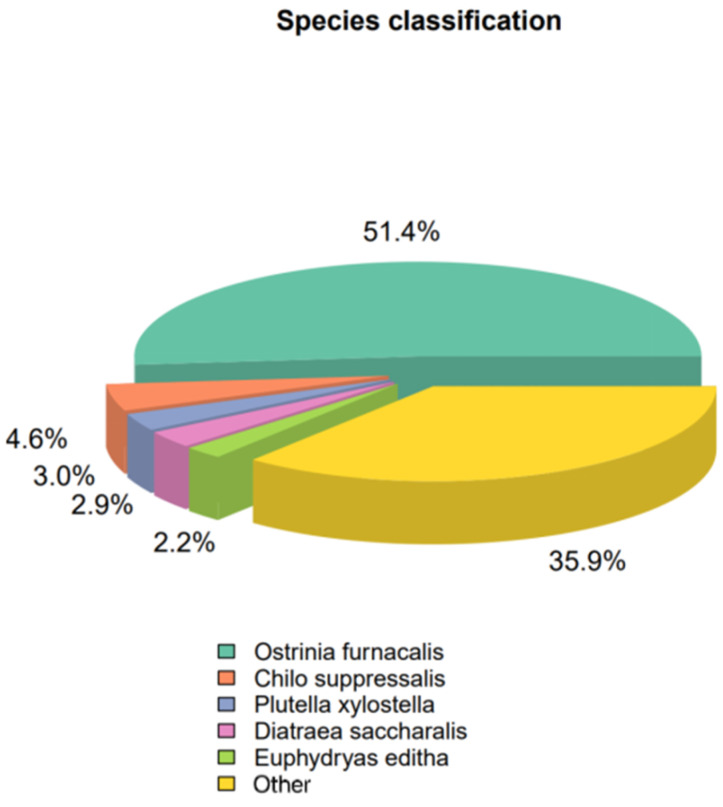
Species distribution of unigenes in nr database is compared.

By BLASTx annotation, 26,120 unigenes (41.53%) were matched to known proteins in the nr and SwissProt databases with an e-value < 1 × 10^−5^. The remaining unigenes did not achieve a significant match under this criterion. Among the annotated unigenes, 57.00% showed the highest similarity to Lepidopteran sequences, predominantly from *O. furnacalis* (51.40%), followed by *C. suppressalis* (4.60%), and *Plutella xylostella* (3.00%) (Figure 2).

**Figure 3:**
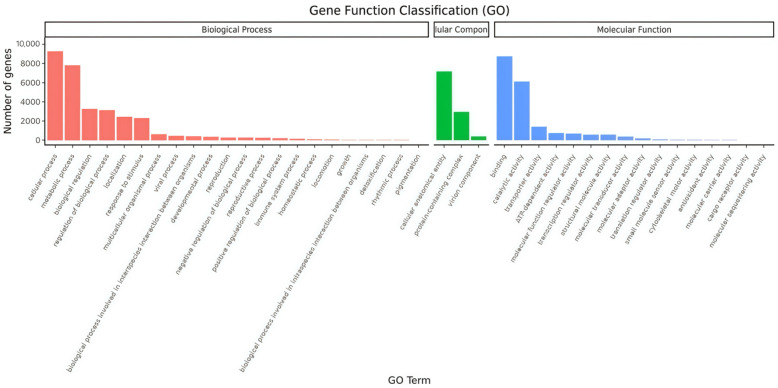
Gene ontology classified annotation of the *L. sticticalis* unigenes.

Based on the BLASTx search results of nr, after the gene ontology (GO) annotation of unigenes by Blast2GO, the successfully annotated genes were classified according to the next level of the three major categories of GO. Of the 62,885 unigenes, 16,863 (26.82%) were annotated with GO terms. Among these annotated unigenes, a higher proportion were categorized under biological process terms than molecular function and cellular component terms. In the biological process category, cellular and metabolic processes were the most prominently represented. In the cellular component category, the cellular anatomical entity was the most abundant group. In the molecular function category, genes expressed in the antennae were predominantly enriched for molecular binding activity and catalytic activity (Figure 3).

**Figure 4:**
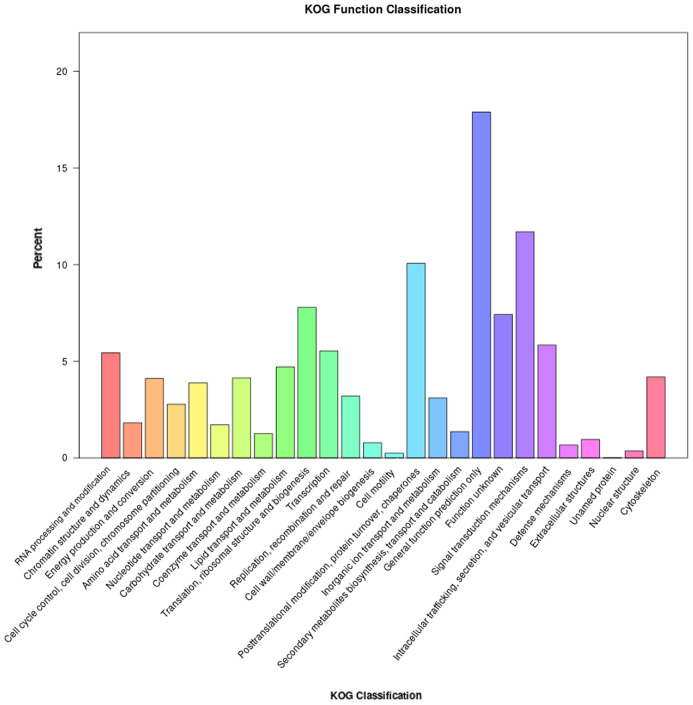
KOG classification of the *L. sticticalis* unigenes.

In addition, the unigenes successfully annotated by KOG were classified according to the KOG group (Figure 4). Consequently, a total of 7642 unigenes could be assigned to 26 specific categories. Among them, the largest category was “general function prediction”, comprising 1367 unigenes (17.89%). The second largest category was “signal transduction mechanisms” with 894 unigenes (11.69%). Conversely, the smallest categories were “cell motility” (19 unigenes, 0.24%) and “unnamed protein” (1 unigene, 0.01%).

### 3.2. Identified Differentially Expressed Genes in Antennae of L. sticticalis

**Figure 5:**
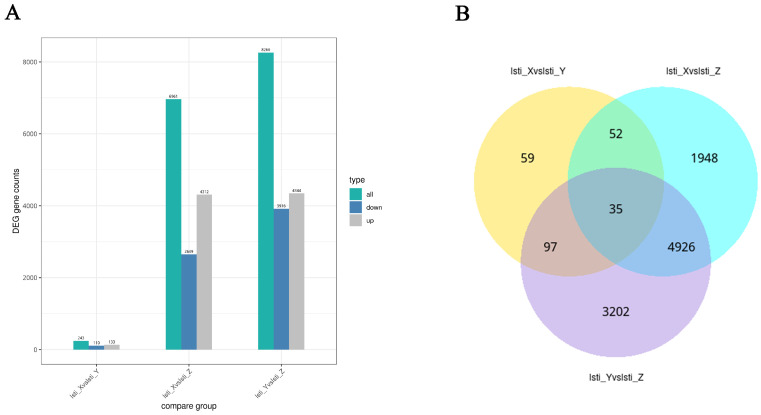
Number of differentially expressed genes in various comparison groups. (**A**) Number of DEGs with (|log2Fold change| ≥1 and *p*-value < 0.05) across various comparative groups. (**B**) Venn diagram illustrating the overlap of DEGs among various comparative groups.

In the transcriptome sequencing results, the FPKM values show that 10,320 genes were differentially expressed between the antennae of male *L. sticticalis* trapped by three different sex pheromone blends. After the global analysis of transcriptome variations, we found some up- or down-regulation of genes. According to the analysis results, there were 8260 DEGs in the comparison of Y vs. Z, followed by 6961 DEGs in the comparison of X vs. Z. When comparing X vs. Y, the number of DEGs was the least, only 243 (Figure 5A). The Venn diagram depicts 35 DEGs identified from the pairwise comparison of groups (Figure 5B).

### 3.3. Functional Analysis of DEGs

**Figure 6:**
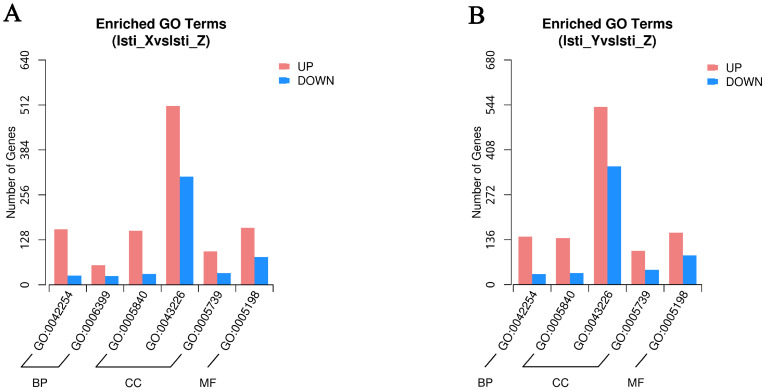
Number of pathways enriched in GO database across various comparative groups. (**A**) Number of up- or down-regulated GO terms enriched in the comparison of X vs. Z. (**B**) Number of up- or down-regulated GO terms enriched in the comparison of Y vs. Z.

To explore the function roles of antennal gene expression in male *L. sticticalis* moths captured using three different sex pheromones blends, all DEGs were mapped to terms within the gene ontology (GO) database. The analysis revealed that 0, 1134, and 502 GO terms were enriched with up-regulation across the three comparative groups, while 0, 1083, and 558 GO terms were enriched with down-regulation (Figure 6A,B). Notably, the DEGs identified in the comparison of X vs. Z revealed the most abundant GO enrichment, with the Y vs. Z comparison coming in second. Conversely, among the down-regulated GO terms, the Y vs. Z showed the most abundant enrichment, trailed by the X vs. Z (Figure 6A,B). There was no significant GO enrichment in the DEGs produced by the X vs. Y comparison.

### 3.4. Identification of Candidate ORs for Differentially Expressed Genes

In this study, 13 candidate ORs were identified among the DEGs of *L. sticticalis* through a keyword search in BLASTx annotation. The predicted protein sequences of these unigenes were further analyzed using NCBI BLASTp against known Lepidopteran ORs to identify additional candidate ORs. Among these, four unigenes contained full-length ORFs encoding proteins ranging from 325 to 422 amino acids, while eight unigenes represented partial sequences as determined by NCBI BLASTp analysis. All 13 OR sequences showed the best hit to Lepidopteran sequences with an e-value < 1 × 10^−5^ (Table 2). Furthermore, the TMHMM 2.0 analysis revealed that all 13 candidate OR sequences possessed between zero and seven TMDs.

**Figure 7:**
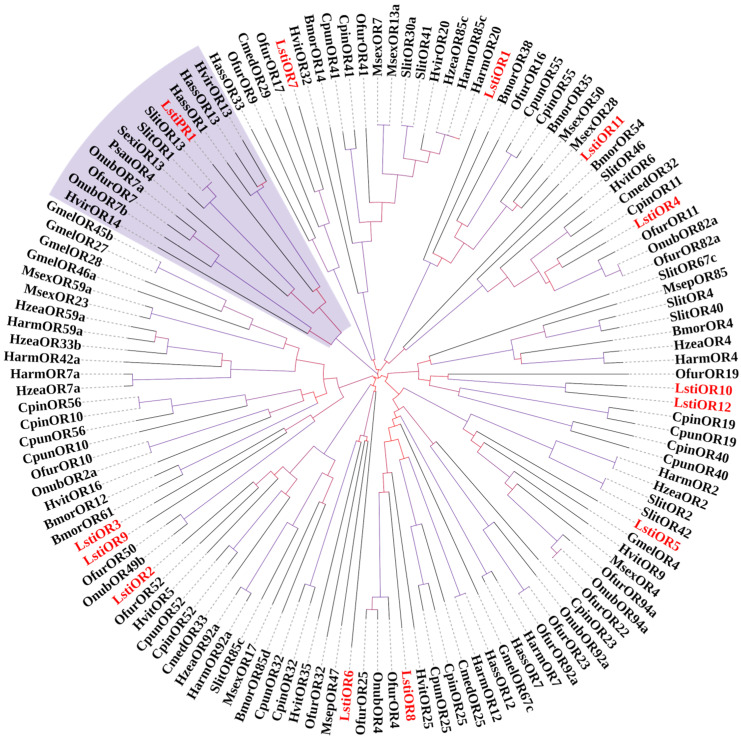
Phylogenetic tree of candidate LstiORs with known Lepidopteran OR sequences. Hvir: *H. virescens*; Hass: *H. assulta*; Harm: *H. armigera*; Slit: *S. litura*; Sexi: *S. exigua*; Psau: *Peridroma saucia*; Onub: *O. nubilalis*; Ofur: *O. furnacalis*; Gmel: *Galleria mellonella*; Msex: *Manduca sexta*; Hzea: *H. zea*; Cpin: *C pinicolalis*; Cpun: *C. punctiferalis*; Bmor: *B. mori*; Msep: *Mythimna separata*; Cmed: *Cnaphalocrocis medinalis*. The red text represents the genes we identified. The clade in purple indicates the PR gene clade.

A unigene, named “LstiPR1”, was identified as a PR due to its significant similarity to previously characterized Lepidopteran PRs and its clustering within the same subgroup in the phylogenetic tree (Figure 7). The other 12 LstiOR unigenes exhibited high divergence from other insect ORs, a characteristic commonly observed among insect olfactory receptor genes. These unigenes were designated as “LstiORn” (n = 1~12), with the numeral indicating the descending order of their coding region lengths.

The phylogenetic trees of ORs and PRs of *O. furnacalis*, *Helicoverpa armigera*, *C. punctiferalis*, and some Lepidopteran insects were constructed (Figure 7). For the relatively conserved PR gene, the LstiPR1 was clustered with the noctuidae pheromone receptor 1 and 13. Nearly all LstiOR candidates clustered with at least one orthologous gene from the Lepidopteran in the phylogenetic tree. No evidence of specific OR family expansion was observed for *L. sticticalis* based on this phylogenetic analysis.

### 3.5. Identification of Candidate IRs and GRs for Differentially Expressed Genes

In this research, six candidate IRs were identified among the DEGs of *L. sticticalis* through a keyword search of BLASTx annotation. Two sequences exhibit full-length ORFs, while the remaining four sequences are designated as incomplete due to the absence of a complete 5′ or 3′ terminus. TMHMM2.0 prediction revealed these six candidate *L. sticticalis* IRs with 0–4 TMDs (Table S1).

**Figure 8:**
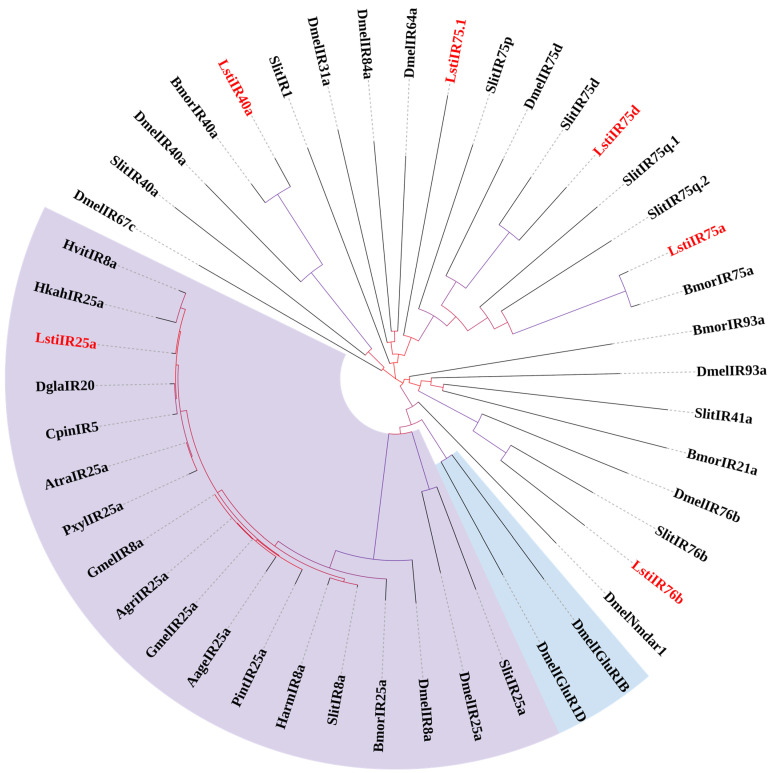
Phylogenetic tree of candidate LstiIRs with known Lepidopteran IRs and iGluRs. Dmel: *D. melanogaster*; Bmor: *B. mori*; Slit: *S. littoralis*; Atra: *Amyelois transitella*; Hvit: *Heortia vitessoides*; Hkah: *Hyposmocoma kahamanoa*; Dgla: *Diaphania glauculalis*; Cpin: *Conogethes pinicolalis*; Pxyl: *P. xylostella*; Gmel: *G. mellonella*; Agri: *Achroia grisella*; Harm: *H. armigera*; Pint: *Plodia interpunctella*; Aage: *Aricia agestis*. The purple-highlighted clade represents the IR8a/IR25a clade; the blue-highlighted clade represents the iGluR gene clade.

The phylogenetic tree was constructed using the IRs from *L. sticticalis* and some Lepidopteran insects (Figure 8). Phylogenetic analysis revealed a clear separation between Dmel ionotropic glutamate receptors (DmeliGluRs) and insect IRs, with the candidate IRs forming a distinct clade separate from them.

The five candidate *L. sticticalis* IRs were named using the numbering and suffix of the IR orthologues of Lepidoptera insects within the same clade according to their positions in the phylogenetic tree. The remaining IR sequence, Cluster-2624.42427, along with its orthologues, has not been detected in other insects, was clustered into the Dmel/Slit/Bmor IR75 clades, and has reliable bootstrap support. This sequence was named “LstiIR75.1” (Table S1). The sequences of all six IRs are provided in Supplementary Table S4.

**Figure 9:**
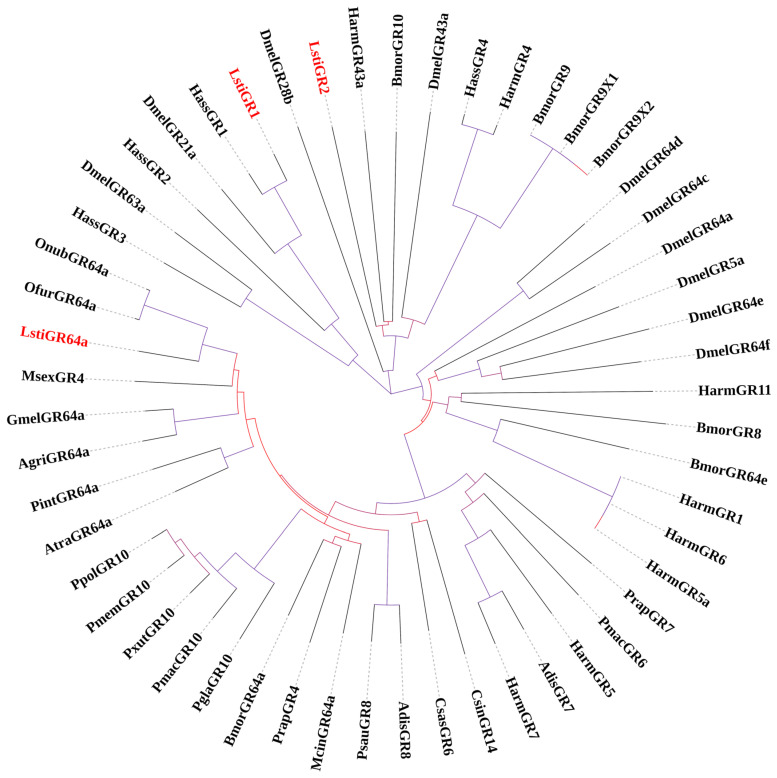
Phylogenetic tree of candidate LstiGRs with known Lepidopteran GRs. Dmel: *D. melanogaster*; Bmor: *B. mori*; Harm: *H. armigera;* Hass: *H. assulta*; Onub: *O. nubilalis*; Ofur: *O. furnacalis*; Pint: *P. interpunctella*; Gmel: *G. mellonella*; Agri: *A. grisella*; Atra: *A. transitella*; Mcin: *Melitaea cinxia*; Psau: *P. saucia*; Csas: *Carposina sasakii*; Pgla: *Papilio glaucus*; Prap: *Pieris rapae*; Msex: *Manduca sexta*; Pxut: *P. Xuthus*; Ppol: *P. polytes*; Pmem: *P. Memnon*; Csin: *Conopomorpha sinensis*; Pmac: *P. machaon*; Adis: *Athetis dissimilis*; Pmac: *P. machao*.

A phylogenetic tree was constructed based on the amino acid sequences of *L. sticticalis* candidate GRs and their Lepidopteran insect homologous genes (Figure 9). In total, three candidate GR genes were identified in *L. sticticalis*, each containing full-length ORFs and six TMDs. Among these, two sequences were named LstiGR1 and LstiGR64a based on their positions in the phylogenetic tree and their clustering with Bmor/Ofur/Onmb GRs clades (Figure 9). The third sequence, Cluster-2624.4010, which exhibited a low bootstrap value and could not be definitively placed on the phylogenetic tree, was named LstiGR2.

### 3.6. Identification of Candidate OBPs for Differentially Expressed Genes

Within the transcript set of DEGs, we identified twelve different sequences that encode OBPs, which comprise three PBPs and one GOBP. Analyzing the sequences revealed six unigenes with full-length ORFs, while the other six unigenes were partial sequences. Additionally, SignalP 5.0 predicted that seven unigenes contained signal peptides (Table S2).

**Figure 10:**
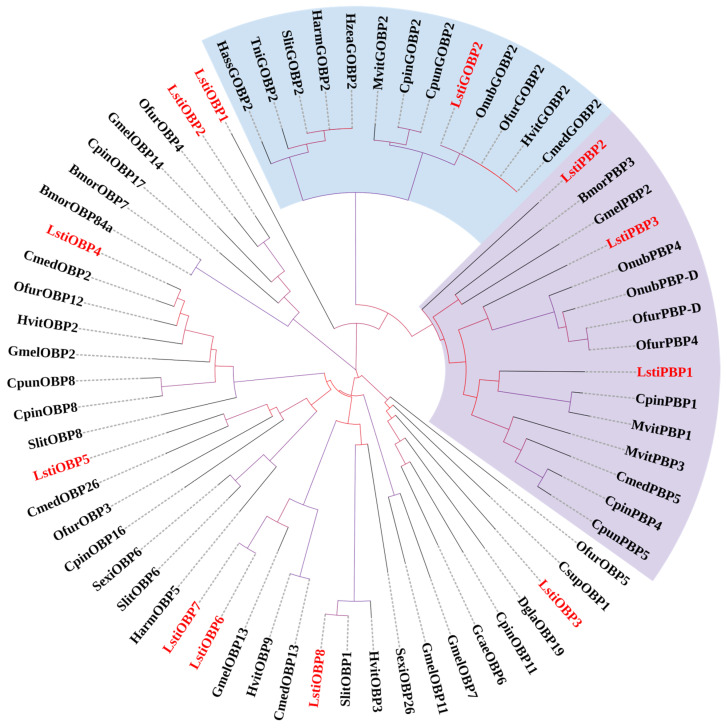
Phylogenetic tree of candidate LstiOBPs with known Lepidopteran OBPs. Bmor: *B. mori*; Onub: *O. nubilalis*; Ofur: *O. furnacalis*; Gmel: *G. mellonella*; Hzea: *H. zea*; Harm: *H. armigera*; Hass: *H. assulta*; Cpin: *C. pinicolalis*; Cpun: *C. punctiferalis*; Hvit: *H. vitessoides*; Meit: *Maruca vitrata*; Tni: *Trichoplusia ni*; Cmed: *C. medinalis*; Slit: *S. litura*; Sexi: *S. exigua*. The clade in purple indicates the PBP gene clade; the clade in blue indicates the GOBP clade.

In the phylogenetic tree, the candidate PBP and GOBP sequences of *L. sticticalis* were clustered into their respective PBP and GOBP clades as anticipated. Specifically, three unigenes (Cluster-2624.31796, Cluster-2624.32604, Cluster-2624.31900) were named as LstiPBP1, LstiPBP2, and LstiPBP3 based on the descending order of their coding region length, and one unigene (Cluster-2624.32578) was named LstiGOBP2 according to its position in the phylogenetic tree (Figure 10). The remaining eight LstiOBP unigenes were named “LstiOBPn” (n = 1–8), with numerals assigned in descending order of coding region length.

### 3.7. Identification of Candidate CSPs for Differentially Expressed Genes

**Figure 11:**
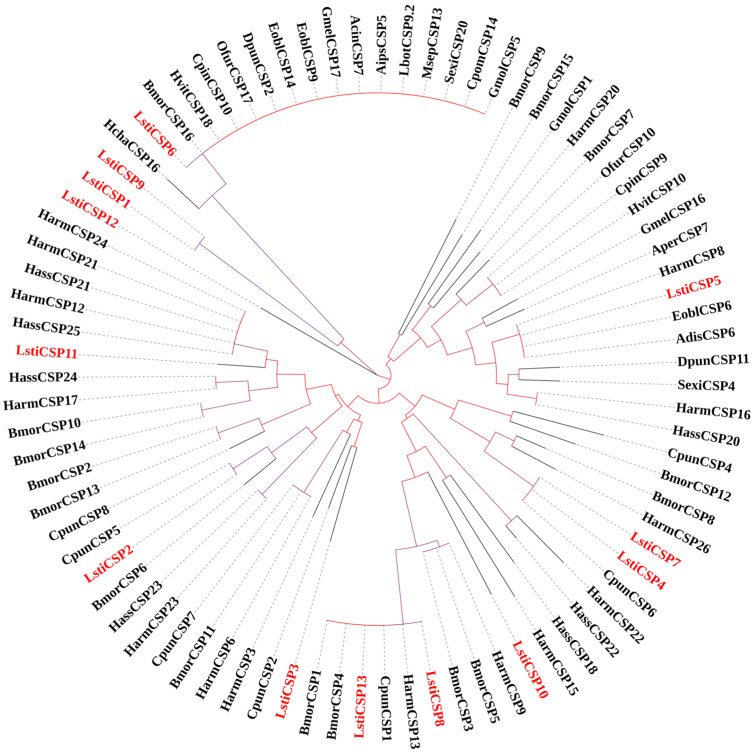
Phylogenetic tree of candidate LstiCSPs with known Lepidopteran CSPs. Bmor: *B. mori*; Harm: *H. armigera*; Hass: *H. assulta*; Cpun: *C. punctiferalis*; Hvit: *H. vitessoides*; Cpin: *C. pinicolalis*; Ofur: *O. furnacalis*; Dpun: *Dendrolimus punctatus*; Eobl: *Ectropis obliqua*; Gmel: *G. mellonella*; Acin: Apocheima cinerarius; Hcha: Heliconius charithonia; Aips: Agrotis ipsilon; Lbot: *Lobesia botrana*; Msep: *M. separata*; Sexi: *S. exigua*; Gmol: *Grapholita molesta*; Cpom: *Cydia pomonella*; Adis: *A. dissimilis*; Aper: *Antheraea pernyi*.

Through bioinformatics analysis, thirteen candidate CSP sequences of *L. sticticalis* were identified in the DEGs. Sequence analysis revealed that among the thirteen unigenes, five unigenes contained full-length ORFs, while the remaining eight unigenes were partial sequences. Furthermore, six unigenes failed in the SignalP 5.0 test. The 13 candidate CSPs of *L. sticticalis* exhibited the highest similarity to Lepidopteran sequences, with an e-value < 1 × 10^−5^, and sequence identity exceeding 76.67% (Table S3). These unigenes were named “LstiCSPn” (n = 1–13) and numbered in descending order based on the length of their coding regions. Phylogenetic tree analysis indicated that all 13 sequences clustered within the Lepidopteran insect orthologous genes (Figure 11).

## 4. Discussion

Antennae are the primary olfactory organs in Lepidoptera that include various olfactory receptors that allow them to perceive chemical signals within their environment [41]. The molecular basis of chemoreception in many Lepidopteran insects has been thoroughly studied, but studies on *L. sticticalis* are relatively rare. Therefore, for the first time, we conducted transcriptome sequencing on the antennae of male *L. sticticalis* adults trapped by three different sex pheromone blends. We further analyzed these sequences by identifying DEGs to elucidate the olfactory mechanism of this important agricultural and migratory pest. The findings from this study provide a critical foundation for further research into the olfactory process and molecular mechanism of *L. sticticalis*.

Our study obtained 62,885 unigenes of which 16,863 (26.82%) were annotated with GO terms. Genes expressed in the antennae were predominantly enriched in molecular binding and catalytic activity. In total, 10,320 genes were differentially expressed across the X, Y, and Z groups. These findings are consistent with the transcriptomic profiles of adult antennae, adult legs, and larvae of *L. sticticalis* [42] reported by Wei et al. Among them, there were 243 DEGs in the comparison between X vs. Y comparison, 6961 DEGs in the comparison between X vs. Z comparison, and 8260 DEGs in the comparison between Y vs. Z comparison. The reason for the large difference in the number of differentially expressed genes in different comparison combinations may be due to the different components of X, Y, and Z sex pheromones, and the ability of the sex pheromones of different components to attract the male adults of *L. sticticalis,* and the strains are also different. We speculate that the male insects trapped by these three sex pheromones also belong to three different strains, which need further study. In summary, our antennal transcriptome sequencing DEGs provide a dataset of 13 ORs, 6 IRs, 3 GRs, 12 OBPs, and 13 CSPs. Compared to the antennal transcriptomes of *C. medinalis* (29 ORs) [43], *C. pomonella* (43 ORs) [44], and *H. armigera* (47 ORs) [45], our dataset contains a relatively smaller number of OR datasets. This might be because we only identified olfactory genes in DEGs, so the number of identified genes is different.

As a group of ORs, pheromone receptors have been extensively studied in various insect species. In Lepidopteran insects, a subfamily of the conserved ORs branch known as the traditional PR clade is specifically tuned to detect female sex pheromones [46]. Phylogenetic analyses suggest that sex pheromone receptors evolved from a common ancestral OR gene [47]. The predicted protein sequences of thirteen candidate OR genes were subjected to sequence similarity comparison, and conducting physiological analysis with known Lepidopteran OR genes using NCBI BLASTp, twelve candidate OR genes and one candidate PR gene were identified. In *H. assulta*, the major component is Z9-16:Ald, but the neurons expressing OR13 in at1 sensilla responded specifically to the sex pheromone component Z11-16:Ald at a dose of 1 mg [48]. Phylogenetic analysis showed that LstiPR1 in the transcriptome was closely related to one or more PRs named OR1 and OR13 of *H. assulta* and *H. virescens*, indicating that LstiPR1 has similar functions with the corresponding orthologous and needs further study.

IRs are a phylogenetically older and structurally more conserved receptor than ORs [49]. The ionotropic receptor, a newly identified member of the chemosensory receptor family, was first identified in *D. melanogaster* through genome sequencing [50,51]. The ionotropic receptor family belongs to a mutant iGluR subfamily. As the receptor of excitatory neurotransmitter glutamate, animal iGluRs play an important role in synaptic transmission [52]. IR8a and IR25a co-expressed with other cell-type specific IRs, while iGluRs consist of heteromeric subunits [53]. It can be reasonably speculated that the expression localization of IR is transferred from interneurons to a sensilla neuron, which may be related to an iGluR [53]. In our study, based on their positions in the phylogenetic tree and strong bootstrap support, five of the six analyzed *L. sticticalis* IR candidates clustered with the IR25a, IR40a, IR75a, IR75d, and IR76b groups, forming small expansions with other putative genes. This clustering pattern is consistent with findings in *S. littoralis* IRs [48] and *H. armigera* IRs [54]. Given the high sequence conservation and similar expression patterns of LstiIRs, we can speculate that the function of LstiIRs may also remain similar to that of IRs in other Lepidopteran species.

Lepidoptera PBPs and GOBPs form a unique insect OBPs lineage [7]. PBPs in moths bind multiple pheromone components with high ligand specificity, thereby contributing to the species-specific nature of pheromone communication in moths [55]. In noctuid species, PBP1-3 is usually found to be expressed in antennae, and another type of PBP, PBP4, is expressed in non-antennae tissues [56]. We identified twelve OBP genes from DEGs in the antennal transcriptome, including three PBP genes and one GOBP gene. A previous study of *S. litura* revealed the effect of PBP knockout on insect gene function, in which PBP1 showed a more critical role in identifying pheromone components than PBP2 and PBP3 [57]. Considering that LstiPBP has high sequence conservation and expression similarity, we can speculate that the function of LstiPBP may be similar to that of *S. litura* PBPs. It is worth noting that all moths contain two GOBP genes, and the number of PBPs varies among different species [58]. In *C. punctiferalis*, after knocking out the GOBP2, the perception of larvae and adults toward feeding and odorants decreased significantly, respectively [59]. In the phylogenetic tree, LstiGOBP2 clustered with CpunGOBP2, and we speculate that they may perform similar functions.

In general, CSPs exhibit greater conservation conserved to OBPs, often displaying 40–50% identical residues even between species from different orders [19]. Our results also revealed high homology among related species, with all 13 LstiCSPs showing amino acid sequence identities ranging from 82% to 100% compared to their CSP homologs. In addition, half of the full-length CSPs consist of about 120 aa, and the remaining half of the CSPs is a partial sequence. When constructing the phylogenetic tree, CSP genes with high sequence consistency tend to be clustered at the same level, suggesting that these genes may perform similar functions [60]. However, the functions of our putative LstiCSPs warrant further research.

In this study, the transcriptomes of the antennae of male adults of *L. sticticalis* trapped by three different sex pheromones blends of X, Y, and Z were analyzed by bioinformatics, and the differences in gene expression between the antennae of three different strains were revealed to identify more candidate olfactory genes. Previous studies have studied the transcriptomes of different tissues of the indoor population of *L. sticticalis* [42], but the analysis of differentially expressed genes in the antennal transcriptomes of different strains of male insects trapped by three sex pheromone blends in the field has not been studied. Our study fills this gap and provides a solid foundation for the future identification of more olfactory genes and the further analysis of the effects of these three mixed pheromone components on *L. sticticalis*.

## 5. Conclusions

In summary, our study presents the first comprehensive transcriptome analysis of antennae from the male adults of *L. sticticalis,* trapped using three different sex pheromone blends. A total of 62,885 unigenes were assembled, of which 26,120 were annotated to known proteins. Among the 10,320 DEGs identified, 46 unigenes were identified as olfactory-related genes, including 13 ORs, 6 IRs, 3 GRs, 12 OBPs, and 13 CSPs. Our results lay a foundation for a deeper understanding of the olfactory genes in *L. sticticalis* and offer valuable guidance for investigating its olfactory system.

## Figures and Tables

**Table 1:** Summary of transcriptome assembly of *L. sticticalis*.

Statistics Item	Total Number	Total Length (nt)	Mean Length (nt)	N50	Q30 (%)
Clean reads	245,724,938				>93.01%
Unigenes	62,885	56,213,711	894	1270	
Transcripts	150,454	132,354,926	880	1238	

Note: Q30: the percentage of bases with Phred values greater than 30 in total bases.

**Table 2:** Unigenes of candidate olfactory receptors.

Gene Name	Length (nt)	ORF (aa)	Unigene Reference	Status	TMD (No.)	E-value	Ident	BLASTp Best Hit
Pheromone receptor								
LstiPR1	726	241	Cluster-2624.14381	Complete ORF	3	4 × 10^−80^	51.05%	gb |AGH58121.1| odorant receptor 13 [*Spodoptera exigua*]
Other odorant receptor								
LstiOR1	1269	422	Cluster-2624.12894	Complete ORF	5	0.00	65.37%	gb |ARO76455.1| odorant receptor 50 [*Conogethes punctiferalis*]
LstiOR2	1227	408	Cluster-2624.8556	Complete ORF	6	0.00	69.61%	ref |XP_063823045.1| putative odorant receptor 92a [*Ostrinia nubilalis*]
LstiOR3	1182	393	Cluster-2624.28955	Complete ORF	7	9 × 10^−129^	46.94%	ref |XP_063827762.1| odorant receptor 49b-like [*Ostrinia nubilalis*]
LstiOR4	1044	347	Cluster-2624.24537	3’lost	6	0.00	77.26%	ref |XP_028162186.1| odorant receptor 82a-like [*Ostrinia furnacalis*]
LstiOR5	978	325	Cluster-2624.43243	Complete ORF	4	2 × 10^−172^	69.85%	ref |XP_028171368.1| odorant receptor 94a-like [*Ostrinia furnacalis*]
LstiOR6	876	291	Cluster-2624.35048	3’lost	5	3 × 10^−179^	78.77%	gb |BAR43483.1| putative olfactory receptor 41 [*Ostrinia furnacalis*]
LstiOR7	813	270	Cluster-2624.38479	5’lost	3	4 × 10^−146^	81.78%	gb |BAR43459.1| putative olfactory receptor 17, partial [*Ostrinia furnacalis*]
LstiOR8	792	263	Cluster-2624.53277	5’lost	4	6 × 10^−126^	65.40%	ref |XP_028167115.1| odorant receptor 4-like [*Ostrinia furnacalis*]
LstiOR9	768	255	Cluster-2624.20941	5’lost	4	2 × 10^−87^	48.63%	ref |XP_063827762.1| odorant receptor 49b-like [*Ostrinia nubilalis*]
LstiOR10	744	247	Cluster-2624.29671	3’lost	5	4 × 10^−58^	43.62%	gb |BAR43461.1| putative olfactory receptor 19 [*Ostrinia furnacalis*]
LstiOR11	597	198	Cluster-2624.48663	5’lost	2	2.00 × 10^−90^	65.64%	gb |BAR43474.1| putative olfactory receptor 32 [*Ostrinia furnacalis*]
LstiOR12	381	126	Cluster-2624.37565	3’lost	2	2.00 × 10^−29^	46.56%	gb |BAR43461.1| putative olfactory receptor 19 [*Ostrinia furnacalis*]

## Data Availability

Data will be made available on request.

## Supplementary Materials

The following information can be downloaded at https://www.mdpi.com/article/10.3390/insects16020152/s1. Table S1: Unigenes of candidate ionotropic receptors and gustatory receptors. Table S2: Unigenes of candidate odorant-binding proteins. Table S3: Unigenes of candidate chemosensory proteins. Table S4: Nucleotide sequences of *L. sticticalis* candidate olfactory genes. Table S5: The sequences used for phylogenetic trees of chemosensory genes (ORs, IRs, GRs, OBPs, CSPs) in *L. sticticalis*.
