# Supplementary material for: Identification of Candidate Olfactory Genes in the Antennal Transcriptome of Loxostege sticticalis Trapped by Three Different Sex Pheromone Blends
Source: Insects. 2025 Feb 3;16(2):152. doi: 10.3390/insects16020152 (PMC11855687; doi:10.3390/insects16020152)
Supplement: Supplementary file 1 [file insects-16-00152-s001.zip › insects-3435517-supplementary.pdf]

Table S1. Unigenes of candidate ionotropic receptors and gustatory receptors

Gene name	Length (nt)	ORF (aa)	Unigene reference	Status	TMD (No.)	Evalue	Ident	BLASTp best hit
					L. sticticalis IR			
LstiIR25a	489	162	Cluster-2624.35050	3'lost	0	1e-113	99.39%	gb | AZT78939.2 | ionotropic receptor, partial [*Dioryctria abietella*]
LstiIR40a	858	285	Cluster-2624.47244	5'lost	1	6e-180	92.28%	ref | XP_028158630.1 | ionotropic receptor 40a [*Ostrinia furnacalis*]
LstiIR75.1	708	235	Cluster-2624.42427	3'lost	0	1e-141	84.75%	ref | XP_063836707.1 | ionotropic receptor 25a [*Ostrinia nubilalis*]
LstiIR75a	1248	415	Cluster-2624.50941	Compete ORF	4	0.00	93.00%	ref | XP_028169063.1 | ionotropic receptor 75a-like [*Ostrinia furnacalis*]
LstiIR76b	1149	482	Cluster-2624.38713	3'lost	3	0.00	86.46%	gb | BAR64809.1 | ionotropic receptor [*Ostrinia furnacalis*]
LstiIR75d	1794	597	Cluster-2624.35781	Compete ORF	3	0.00	72.01%	ref | XP_028169804.1 | ionotropic receptor 75a-like isoform X1 [*Ostrinia furnacalis*]
					L. sticticalis GR			
LstiGR1	1374	457	Cluster-2624.12537	Compete ORF	6	0.00	93.23	ref | XP_028161313.1 | gustatory and odorant receptor 22-like [*Ostrinia furnacalis*]
LstiGR2	1113	370	Cluster-2624.4010	Compete ORF	6	3e-87	60.74%	ref | XP_028175089.1 | gustatory receptor for sugar taste 43a-like [*Ostrinia furnacalis*]
LstiGR64a	1212	403	Cluster-2624.34712	Compete ORF	6	0.00	98.86%	gb | WYN03153.1 | gustatory receptor 7 [*[Loxostege sticticalis*]

Table S2. Unigenes of candidate odorant binding proteins

Gene name	Length (nt)	ORF (aa)	Unigene reference	Status	Signal Peptide	Evalue	Ident	BLASTp best hit
LstiPBP1	426	141	Cluster-2624.31796	3'lost	Y	3e-70	73.72%	gb | ADT78496.1 | pheromone binding protein 2 [*Ostrinia nubilalis*]
LstiPBP2	429	142	Cluster-2624.32604	Complete ORF	N	4e-76	74.65%	gb | ADT78499.1 | pheromone binding protein 5 [*Ostrinia nubilalis*]

LstiPBP3	282	93	Cluster-2624.31900	5’,3’lost	N	4e-44	97.10%	gb | ACF48468.1 | pheromone binding protein female 2, partial [*Loxostege sticticalis*]
LstiGOBP2	486	161	Cluster-2624.32578	Complete ORF	Y	2e-118	100%	gb | ABY75632.1 | general odorant binding protein 2 [*Loxostege sticticalis*]
LstiOBP1	492	163	Cluster-2624.3670	Complete ORF	Y	1e-116	98.77%	ref | NP_001079003.1 | odorant binding protein IA-like precursor [*Mus musculus*]
LstiOBP2	429	142	Cluster-2624.32099	3’lost	Y	4e-71	94.55%	gb | ACF48467.1 | pheromone binding female 1 [*Loxostege sticticalis*]
LstiOBP3	423	140	Cluster-2624.33469	Complete ORF	Y	1e-99	99.29%	gb | WYN03124.1 | odorant binding protein 33 [*Loxostege sticticalis*]
LstiOBP4	420	139	Cluster-2624.32637	Complete ORF	N	2e-88	89.93%	gb | BAV56799.1 | odorant binding protein 12 [*Ostrinia furnacalis*]
LstiOBP5	360	119	Cluster-2624.32825	3’lost	Y	2e-49	68.60%	gb | BAV56790.1 | odorant binding protein 3 [*Ostrinia furnacalis*]
LstiOBP6	342	113	Cluster-2624.33606	Complete ORF	N	5e-57	76.11%	gb | ALT31643.1 | odorant-binding protein 13 [*Cnaphalocrocis medinalis*]
LstiOBP7	318	105	Cluster-2624.36680	3’lost	Y	9e-46	66.99%	gb | ALT31643.1 | odorant-binding protein 13 [*Cnaphalocrocis medinalis*]
LstiOBP8	246	81	Cluster-2624.38940	5’,3’lost	N	7e-47	91.36%	gb | BAV56797.1 | odorant binding protein 10 [*Ostrinia furnacalis*]

Table S3. Unigenes of candidate chemosensory proteins

Gene name	Length (nt)	ORF (aa)	Unigene reference	Status	Signal Peptide	Evalue	Ident	BLASTp best hit
LstiCSP1	462	153	Cluster-2624.32786	Complete ORF	Y	3e-108	100.00%	gb | WYN03131.1 | chemosensory protein 11 [*Loxostege sticticalis*]
LstiCSP2	408	135	Cluster-2624.39782	Complete ORF	Y	7e-80	91.87%	gb | WPA71056.1 | chemosensory protein protein 14, partial [*Diaphania glauculalis*]
LstiCSP3	390	129	Cluster-2624.33276	Complete ORF	Y	1e-80	89.92%	ref | XP_028174915.1 | allergen Tha p 1-like [*Ostrinia furnacalis*]
LstiCSP4	369	122	Cluster-2624.32120	3' lost	Y	2e-72	83.61%	ref | XP_063830609.1 | allergen Tha p 1-like [*Ostrinia nubilalis*]
LstiCSP5	363	120	Cluster-4147.0	Complete ORF	Y	1e-61	76.67%	gb | BAV56814.1 | chemosensory protein 10 [*Ostrinia furnacalis*]
LstiCSP6	321	106	Cluster-2624.25708	Complete ORF	Y	4e-70	100.00%	gb | WYN03141.1 | chemosensory protein 21 [*Loxostege sticticalis*]

LstiCSP7	249	82	Cluster-2624.32727	3' lost	Y	8e-47	86.75%	ref | XP_063830609.1 | allergen Tha p 1-like [*Ostrinia nubilalis*]
LstiCSP8	201	66	Cluster-2624.30893	5' lost	N	2e-41	97.14%	gb | BAV56805.1 | chemosensory protein 1 [*Ostrinia furnacalis*]
LstiCSP9	189	62	Cluster-2624.23083	5' lost	N	4e-36	100.00%	gb | WYN03131.1 | chemosensory protein 11 [*Loxostege sticticalis*]
LstiCSP10	153	60	Cluster-2624.33314	5' ,3'lost	N	2e-19	83.33%	gb | APG32551.1 | chemosensory protein [*Conogethes punctiferalis*]
LstiCSP11	171	56	Cluster-2624.33335	5'lost	N	1e-26	82.14%	gb | BAV56815.1 | chemosensory protein 11 [*Ostrinia furnacalis*]
LstiCSP12	138	45	Cluster-2624.30641	5' lost	N	6e-21	86.67	ref | XP_063830357.1 | putative odorant-binding protein A10 [*Ostrinia nubilalis*]
LstiCSP13	129	42	Cluster-2624.31804	5' lost	N	2e-41	97.14%	gb | BAV56805.1 | chemosensory protein 1 [*Ostrinia furnacalis*]

S4. Nucleotide sequences of all identified candidate olfactory genes in the Loxostege sticticalis

>LstiPR1 Cluster-2624.14381; orf1 len=990 frame:2 start:551 end:1540 putative pheromone receptor OR7a [Ostrinia nubilalis]

ACTAACGATTTTTATTTTTTATTTAGAGACGTAAAACGAGTTGTAGACATAAAGTATATACGGGTGCTGCGTTCTTACTTGCGTATTAT

AAGCGCATGGCCGGCCAAACATGTTGGCGACACACCAACGAAATGGGACCGAATAAAAGGCAACCCCGTTCTGGTCTTAAGCATC

ATAAACTTTTTAACTGGACTACTTTACTTGAAGGAAAACATTGGGAAAATTAAATTTTTCGATTTGGGCCAAACTTATATTACTGTGT

TGATGAACTTAGTTTCCGTGTCTCGACAACTCATGGTATACCAAAAATCATATACAGAGGTATCCCGGGATTTTGTGACAAAAGTTC

ATCTGTTCAAC

TGGAAAGATGACTCGGAATACGCTATGGAGATACACATATTGGTTCATAAAATATCTCACTTCTTTGTAATGTACATCCATGGGCTGA

TGTTCATCGGACTCAGTATGTTTAACTTGACACCTTTGTACAACAACTATAGTAATGATGCGTTTACCAAGAGACTCCACGGAAACG

CTACTTTGGAACATGCTGTGTATTATTCGCTGCCTTTTGACTACACCACTCAAATTCCAGGATACATTGTTGTATTTACCTACAACTGG

TTCATATCTTTAGTGTGCTCTATCAACTTTTGCTCGGTGGACACGTATATGTCTCTATTAGTGTTTCATCTTTGGGGACATCTAAAAAT

CCTCATTCACAATTTGGAGCACATACCCAAACCTAGTGGGCTAAAATCTTCCGCAAATATAAACGGTGCAACCCAAACTGAGAGGT
ACAATGAAGATGAAACTCAGCAAGTATTTGAGCGATTGAGAGAGCTGATTAAACATCACTGTCTTATTCGAAATTTTATCAGCATTA
TGTCCAGTGCCTTCGGATACGTATTACTCGTATATCTGGGATTCCATCAAGTCTGTGGATGTATACTGCTTCTGGAATGCTCTTCATTG
GTAAATATTACGTAA

>LstiOR1 Cluster-2624.12894; orf1 len=1329 frame:1 start:31 end:1359 odorant receptor 15 [Heortia vitessoides]
AAATATCAAACAAGACAAGGCAATGTCTATTTTAAAAGCCATTCCCCTCTCGAACCAAACATGTTTATGAATTCGAAGTTTCAGCAG
CTACTTGGCACAATGTGGGAGAACCTTCGCAAGTTCGGGCTGGGGCATTGCGACCTGCCCACCATGGTGTGGAACGTAGCCTTCAT
GCTGCGGGGGTTCACCCTCAACATCGACAGCAGGTTTACTGGACGTATCCCTAAAATATTCTACATCACCACCATCATCATCGCGTT
CTGCTACCTCTATTCTTACTTCTTCTCGATGCTCTGGTTCGTATTCGTGCGCTGCATCGAGACCGGTGACGTGACAGCCGCCATGATA
GTCTTTCCGCTGGGCATCACCAGCGAGATCGGCATCGCCAAGTTTATCTACACTTGTGTCTACAGGAAGAAAGTTCGCCAGCTCCT
CCAACAGTACCTGGAATATGACTCTCAGATCCCCCAGGGCTCTCGTTTATCCAGGCACCTTCTACAAGCCCTTCGAAACGTCAAAC
GACGCGCACTCATCTACTGGATCTTTATCGTCAGCAATGGCACCCTCTACATCCTCCAGCCACTGGTTATGCCAGGGAGGGTACCGA
TGGAAGAAGTTTTTGTTCTATATGGTTTGGAGCCAGAACTGGAGACTCCTAATTATGAAATAACATACGTTCTCTGCACTTTTGGGT
CCGTCTGTACTTGCTACCTAACCTCCAACGTGGCTGCGTTCCTTATCATCGTCAGCGGCTACGTGGAATCCCAACTATTAGCACTCA
GCGAGGAAATCCTGAATGTTTGGGATGACGCCGAACTCGAGTACAAAGTCATAGACAACGCCGATGAAGAAGAATTTGAAAACAA
AGAGAAATATGATGCTATAAATGAGTCAGTTAAAATCCGACTTAAAGATATCGTCAAAGGACATACAACTAATATAAACTTATTACTG

CAAGTCGAAGACATCTACAGGGGTGCGTTCGCGTTCGAGTTTTGCATACTTTCTGTAGGTTTGATAGCGGAACTTCTAGGTGGGTTG
GAGAACACATACATGGAAGTTCCTTTTGCTATAATCCAAGTTGGGATGGACTGTCTAATTGGACAGAGAGTAATGGATGCATGTGAC
ACTTTTGAAAGTGCCGTGTATAGTTGCAAATGGGAGAGGTTCAATGAGGCCAATATGAAGACAGTGTTCATGATGCTCATGAATTCG
CAAAAGACCCTGACGCTGACGGCTGGGGGTATAGCAGTCTTAAACTTTGTTTGTCTCATGTCTGTGTTTAGGTCAATTTACTCAGCG
TTTACAACCTTACAGTCCGTTATGTAG

>LstiOR2 Cluster-2624.8556; orf1 len=1245 frame:1 start:31 end:1275 putative odorant receptor 92a [Ostrinia furnacalis]
AAATTCATCTCCACCAAAATGCCCGAAAAATCTTACGGTACTGTAAAAAGCAATTTGCGCGAAGAATTGAATTACATAAATTCTATG
GGTTCCAAAATATTTCTTTACCCTTTTAGTGGAAGATCTAAGCTAGTTGACATCTGCTACTTGTTTGTGTGTTTTCTAGTGGTCGTGA
CAGCTACTCAACTCTTGACTGCGCTGCTTGTCACTGATTTGAAGGAATGGATAGAGATAGTCAACGTCGCACCCAATTTAGGCGTT
GTTCTGATGACATTACTAAAATACACTAAAGTGCACAACAATCAGCATGTTTACAAAAAGATATTCAAGCACTTTAGCGACGACCTA
TGGGATGTTGTTTTTGATTCTTATGATCATAAGAAGATCGTGATACGCTATACTGCTATTGCGAAGTACGCAACAAGATTTTTGTTCTA
TTACTCTGTGCCTCTTGTCGTTTTCGTCGATTCTTTTCCAAGGATCATAATGTATTTAGAGAATGAAATTATTGGGAATGAAAACCCA
CAATATTTATACCCATTTGACGGTTGGTATCCGTTCGATAAAGTGAACTGGTATTATACTGCTTATCTTTGGGAGAGTTTCATGACATT
TATTGTTGTCTGCGTCTACGCATTCTCAAATATGATCCACGCATCGTATACCTCGTTCATATGTATGGAACTAGAAATTTTGGGAGTAA
GCATCAAAGATCTAATAACTCCTGATGATGTCACCAATATAACAAACCATTTAAAGGTTCAGGAAATTCATAGCCATATAAAACGAA
AACTGAAAACGATAATTAGAAGACATCAATTTTTGGCTCAGCTTGCTTCAGAGTTAAATATAGTGTTGGGGGACATGATGCTTCTCA

ACTATATTTTTGGGTCTGTTTTTATAACGCTTACAATTTTTACTGCTACGGTTGTGGATAACATGTACAAATCTCTACGATACTTTTTCA
TGTTTTGTTCGTTGATTGTGGAAATATTCTTCAATTGTATGATTGGACAAGTCCTTAGCAACCATAGTGAGCAGTTGACAGATGCAAT
ATATTCAGCCGACTGGCCATTTGCCGACAACGAGACGAAAGTGATGCTGCTGATCCTCATGAGGAGAACCCAAAAGCCGTTCGAG
TACACCGCCAATGGCTACCTGGCTATGAATCTTAACAGTTTTAGCGGTGTTTGCAGCATGTCATATCAATTATTCAATTTAATCCGCAC
CGCTTACAGCAAATAA

>LstiOR3 Cluster-2624.28955; orf1 len=1188 frame: -3 start:1462 end:275 putative olfactory receptor 50, partial [Ostrinia furnacalis]
AACAAAATGGATCCTAACAAGCTATCTCACTCATTTTACAAAATTACATATGTTTGGAAATTACTCGGAATGTGGTCAGGTAAATCAT
CAAGTAAAAACTTGAGAATATATTCTTGGCTCTTCGTTACTTTGTATTACATAATGTACAACTTCTTTTACACATTGAGCTTGGTATGC
GCACCACGAGCAGTAGATACAATAGGCGTAGGAGTATATTACTTCACTACATTGTGTGGGTTAGCTGAAATTGTCATGATTCTGAGA
AATCGGCAGAAAATCATCTCCGTCTTTGAAACAATGGATTGCAAGGAGTTCCAAGGAAATAATCGCCAAACTAATGAATACCTTCG
ACAATTTAAGATAACCTTTTCAAGATACTTCAAAGTATATGCAGCGTACTGCTTTATCAGCTCACATACATTTCTTATGTTTCTGCCTT
TATTCAATTACTTTTTTCAACACAAAGAACTGGAAATGCCGATCTGGGAATATTATTTTTTGACCAATGCAACACGGAACAAGTATTT
TTTCTATCTCTACACATACCAAAGTATGGGCATGATAGCAACAATTTTTAATCATATAGTTTACAATGTATTTTTATTCGGAATATTATC
CGTGGCTGTTAGTCAATCTAAAGTACTAAATTGGAATATTGCAAATATAAGGCTCGCTGATGAGGATATTTTAAAGAGCACGGAAGA
AAAGGAACGGTTGTATTTAAATAAATTATATAACTGTTTGAAACATTATGAAATCATCTTGAAGTATTGTGAAGATGTACAAGATTTA
ACAAGCTTTTTAATATCTTTGAATTATGGATTGTCAGTTTTCACTTTATGTTTTAGCATGTATATGTTCCTTCTGCCTACGAATAGTAAC

ACTTTGGTTTATATGGGATTTTACCTAAGCGCGATATTGATCAAAAACTTTGTTCCTTCATATTTAGGCAGTGAACTAACAAATGAGA
GTAACAATCTACGGTTCGCAGTATACAGTTGCGATTGGGTTCCACGATCTAAGAATTTTAAAACTTGTCTCATGATTTTCGTGGAAC
GAGCTCGCAGGCCTCTGCTTATTAAGGGCCTCAAAGTAGTGCCACTGTCTTTGGCAACATTCACTTCGATTGTGAAAACAGCGTAC
TCCTTTTTCACTCTTCTGAGAGGGGCTCAAGATCAATTAATATAA

>LstiOR4 Cluster-2624.24537; orf1 len=1083 frame:2 start:2 end:1084 odorant receptor 82a-like [Ostrinia furnacalis]
GTTTCGTGGTCTCTTAAGTTCTGCAACGTTTACCATAATATGGATTTGTTCAATTATATCAAAAGGGTCTTTCGCGATGCGAAAAGTC
GCTTGCAGGAAAATATTACGAAAGTTTGTTGTCGCTTGTTAATTTTGTTCCAAGTGTGGCCGGTTTTTCCATTAGGGGAAACACTAT
TTTTGTTCCATTCTGGATATTGCACTTGTCTCTCCTATTTTACATCTATGGCGTGGGCTGCGCTGTATACCAGATCAAGTACGCAGAG
GATGCACGCGACTTCATCAAGAGCTTCGTCAACGTGTCTCTCCTCGTGCTCATTGCGAATAACAGCCACTGGTTCTTGCAGAAAAG
ATCTTTGCTAAAAACAGCTTTGAAAGAGATCAGTGAGAGCGACGTGATGGCCACTGCCAATGAAAGTTTCCGCCAGAAGCATGAG
AGATCGGTGCAGAAGATTAAGAGGATCCTGTTTATCTTCTATGGTTTCAATTTATTAAATGCCACGTTCGTGTACTTGCCGCATCGTG
CTGATGTGCTGAATAGCTACTCTATGACTCCGTGCTTCGGAATGGAGCCGCTAACTTCATCACCTAATCGAGAGATTTGTATGACACT
CCTTTGTATACAAGAGATCACTATCATGGTCGTCGTTCTAAATTACCAGGCACTCCTTTTGCTTCTGATAGCCTACACTGCTCTCATG
TATACGTTGCTCGCTGATGAAATTATGACTCTCAACAATTTCGATCGGGAGACTTATTACAATAATCCTACGGTGAAGCTTATCCTAC
CAGATTTGGTTAAACGTCACGCGATTCTTCTAAGCATCATTGATAAGTTGAAGGCTTTATACAGTGGTTCAATTGGGGTAAACTTCG
GGTCGAATGCTGTGTGCATTTCGTTGTTCTTTTATTTGCCTCTTCAGGAATGGCTTCAGTTCATGCCAGTGCTAGTGTACTGTTTTCT

GGTGTTCTTTCTTTATTGCTTTTTATGCCAGAGGCTGACAAACGCAGCTGAACTCTTCGAGATGTCTGTGTATGCGTGTGGGTGGGA
GAATTTCGAGTTGAAGGAGAAGAAAGCTATATAC

>LstiOR5 Cluster-2624.43243; orf1 len=990 frame:1 start:1 end:990 putative olfactory receptor 22 [Ostrinia furnacalis]

GTATCAACGACAATGCTTAGCGCTGTCTGTTGCATCAAAGTCGCTACGTTCGTAGCTTGGCAAAAAGATTGGAATAGCATCTTCGAG
TACGTTTCCAAATTGGAAAAACGACAGTTGTCTCAAAAAGACAAAGTAACAGATGCTATAATTGGTGGGTATATTAAATACTCCCGT
CGCCTTATGTTCTTTTATTGGGCTCTGGTAGCAGCAACGATTACGACTATTTGCGTTGCACCTTTAGTTGGCTTTTTGTCATCTGCAG
AATACAGAGAACGTATAAGAAACGGAACTGCTCCGTATCCAGAATCAGTAAGCGCTTGGATGCCTTTTGATAGGACCCGAGGTTTA
GGGTATTGGGTGACGATTATTAGTCATAATTCGATAATGTGCTACGGAGCCACCATCGTGGCAAATTACGACGGTAACGCCATGGTTT
TGATGACGTTTTTCGCTGGGCAGCTGAAAATTCTTAAAGAGAATTGCTCGAGATTATTTGATGATGAGGAGGAAACAACTTGCGAA
GATGCGATCAAAAAAATTAGGGACTGCCATTATCACCATCAACTGTTGATCAAGTATACAAAGGTTTTCAATGGCTTGTTATCTCCA
GTTATGTTTACTTATGTGATTATCTGTTCTTTGATGATCTGTGCAAGTGCCATCCAAATAACTAAGGGAGGAACAACGACAGTGCAA
CGTATCTGGATATCCGAATACGTGATAGCGTTGATCGCACAGCTGTTTTTGTACTGCTGGCATAGTAGCGAGGTGTTGGCAAGGAGT
GATGAAGTAGAAGACGGTGTGTATGCCAGCGCGTGGTGGTCGCGCAGCATCCGAGTGCGCAGATGCGTGCTGCTGCTCGCGGGCC
AGCTGCGCACGAGTGTCGTGTTCACTGCGGGGCCGTTCACTGAAATGACTTTACCCACCTTTGTTGCTATATTGAAGGCGTCGTAC
AGCTATTACACTTTGTTAGTCAACAAAGATGACTAA

>LstiOR6 Cluster-2624.35048; orf1 len=891 frame: -1 start:893 end:3 putative olfactory receptor 41 [Ostrinia furnacalis]

GGGGCTTTCTTTAAGATGTTCCTAATGTACTGGAAAAGGGTCCAAACCAAATCAATAGTGGACCAAATCAACAGCGACCACGAAG
CTTTCAACCACCTTCCACGCAAACAGCAAGACATAGCGTTTCTCTACATAAAAGCTGGTGTTCGCAACGTCGAACGTATCTGGGCT
CCTTTAGTCTCGGTTGCTATTATGATGTTCCCTGGTATGGCTGTTATCTTGACCCTTTACAGCTACACGTTCAATGACATACCCAAAA
AATACATGATCCACGAATTAAACCCTCCTTTTTCTACGGATCCTGAGGACATGTCAAGATCGCCATATTTCGAGGTTCTTTTCGTATA
CGAGACTGGGGCTGCCATAATTTGTGTGCTGAACTATACTGCCTATGATGGGTTGTTCGGTGTAGCGACGAACCATGCTTGTATGAA
GATGAGCTTGTGCTGTGTGAAGTTGAATGACGCGTTCGCGTGTGAAGATCAGGAGGAAATGTATAAAGGCGTTCTAGCGTTCATTG
AAGAGCAGCAGAAGATGTATAAATTCGTCGACCTGATTCAAGAGATTTTCAATATATGGCTGTTTGCTATTTTGATGAGCACTATGAT
ACAAATTGGATCTTTACTTTTCCATATTTCAGCGGGCTACGGGTTCGATCTGCGCTACACGCTGTTCAGCTTTACTTCTGTTGTGCAC
ATCTTCCTGCCTTGCAAACATGCGGCTACACTGAAAAGCATGTCGACAGAAATGGCGACAATGATCTACATATCCGGCTGGGAGCG
CTCCCGCGACCGGCGCGTGCTGCGCATGATCCCCTTCATGCTTGCGCGCGCGCAGGTGCCCAACTACATCACCGCCTTTGGCATGT
TCGTGTTTGATATGGAGCTGTTT

>LstiOR7 Cluster-2624.38479; orf1 len=822 frame:3 start:1542 end:2363 uncharacterized protein LOC114362478 [Ostrinia furnacalis]

CAATTTTCTATGTTCCCTGACCCATCTTTGAATCACTTTTTTGTCCCTCATGATTCGTTGATAGAGGATATAATATTTGTTTCCATGTCA
ACAGGTTTGGAACCTATGGAGAAATCACCAAATTTCGAGTTCGGATATGCCCTCGTCGCTGCCGCCGTCTGGTTCCTCTGCTACGTG
CCAGCCAACGTCACCGCAGTCCTCATCGTCGTCGCCGGCTACATCGAGGCACAAATGCTGGCCCTCACCCAGGAACTTCTCCACGT

TTGGTCTGACGCCGAGCAGTACTACAAAAACCTTAATTTCACTGTACTTAAAAGAGGATCATTCGTTGACGCTAACTACAAAAAAA
GAGTTCTCAACGAATTCGTAACGATGAGGCTCATTGATATAATACAAAAGCACGCAAGAAATGTATACCTTTTGCAGTTGTTAGAAG
ATGTATTTCGTGGAGCAATCGCTTTGGAATTCGTGCTCCTAGTTCTGGGTTTGATTGCTGAACTGTTAGGTGGTTTGGAGAATACTTT
TTTGGAGATGCCATATGCATTCGTTCAGGTGGCCATGGACTGCTGGACTGGTCAGCGAGTGATGGATGCGAGCGCACAGTTCGCAG
CTGCTGTGTATGCTTGTCAATGGGAAATGTTTGACGTCGCTAACATGAAAATTGTGTTACTGATGCTGGCGAGCGCCCAGAAGACG
ATGAAGCTGTCTGCTGGAGGCGTGACGATGCTCAGCTTTGAAAGCCTGATGGCCGTTGTGAGATCGATTTATTCTGCTTACACTACT
TTACAATCAACTTTAAAAATGAATGTGCATATTAAATAA

>LstiOR8 Cluster-2624.53277; orf1 len=819 frame:1 start:1 end:819 odorant receptor 4-like [Ostrinia furnacalis]
TCGACTTCTGGAGTGCCCAACGACAAAATGTCGGAAGAACTTATCTTCGATAAGTCTTTGCAGAACATAAAGTTTATATTTCGATTA
TCTGGCTTGAATATTGATCATAAGAAAAGAACCTTCAAGCAAAATTGTATCTATTTGTTCAATTTACTTTGGCACCAAACTGATCTCC
TTTCAGCTTTATCATGGGTCTTATGTGGAATGTTTACTGGAAAAGTCTTCACGGAGCTTACCTTCGTCGCTCCTTGTTCTGTTTTCGG
CATTCTTGCTGATACAAAAGGTGTACTCTTCATCCTTCAGGAGAGAAAGATATATAGCTTGATGAATAACCTTAGAGACTTAGAAAT
CAAAGCCAAAGATTTTGTAGGCACCAGAAGAGATAGCTTGATTAAACCAGATATATACCTTCTGGACATTGTCATTAAGGTGTTAAA
TATACTAAACGGCCTAATGATTGTAGTCTTCGATATGTGCCCTCCGATTTTTATAGCGATAAAGTATTTTACTACAGGGCAGTTGGAAC
TGATGCTGCCATTTTTGGATGTCTATTTTTTTGATGGATTCGACCTTAGGTATTGGCCTTTTCTGTATGCCCATCAGGTTTGGTCCGTG
TGCGTCTCCCTCCTCGAGATCTGCGCCACAGACTACTTCTTCTTCACCTGCTGCACTCACATCAGGATCCAGTTCAAACTCCTTCAA

CACCAGTTCCAAGATATCCTGACCAACAGGAGCATAACTGCTGATTCAACCAATCAGATGCCCATTCAAGCCCAATTTCGAGAACT
GGTCAAGTGGCATCAGGGAATCATTACGTGA

>LstiOR9 Cluster-2624.20941; orf1 len=774 frame:2 start:107 end:880 putative olfactory receptor 50, partial [Ostrinia furnacalis]
AACAAAATGGAGCCTTCTAAGCACTCCCAGATTTTCAATTTGACTATAACTCTTTGGAAA
ATATTTGGATTGTGGTCAGACAAATCGTCAAGCATATGGTTTCGATTGTATTCTTATATATTTGTAACTGTCATTTCATTTGGCTACTTT
TTCTTTTATACTTTAAGCTTATTTTACACACCACGGACATTGGAAATATTTGTATCTCAAGCAATATATTACTTCATTACATTCTCTGGT
ATTATTAGAGTTGTGGCAATCTGGATAAAGCATCCGCAATTGATCTCCAATTTCAAAATGATGGATTGCAAGGAATTCCGAGAAAAC
GACGGAAAAACGAACGAATACATTAGACAATTTGAGAATGGCTACAAAAGGTACTTCAGAGCATATGCAATTCACTGCAGTGGTTC
TGCATACACATTTATTCTTGTTATACCTTTATTGAATCATTTTTTTCGTCAAGAAGAACTGAAACTACCGATCTGCGGAAATTACTTCC
TGACTGATGATCAAAGGAACAAGTACTTTCTCTACTGGTACTTATACCAAACTGCAGGCATGGTAGGAACAGTCACTAATAACATTA
CTTCTCATACACTTATTTGTGGACTTAATTACATGGCAATAACGCAGTTTAAAATACTGAATTATAATATTGCAAATATAATGCTCGATG
ATGATGACATCCCCAAAAACATACAGGCAAAGGAAAGGATTTATTTAAATAAATTATTAAAATGTCTGAGACACTACGAAATTATTTT
GGAGTAA

>LstiOR10 Cluster-2624.29671; orf1 len=759 frame:2 start:32 end:790 uncharacterized protein LOC114363237 [Ostrinia furnacalis]
AATAATTTTACTACAATGGACGGCAAATACCCCGAGGAATTCATAAATTCGTTACTTATAAGTTTGAGCTACTTTAAAAAATGCAACA

TTGATGTTATCCATTCAAATAATAGCTTGTGTGGAAAAATCTGGTGGTTTTTCAACATACCGAGTTTTATTTTGAACTACATAACTTTG
ACGATGCACATTATAAAAATCTTTACGGAAGGCGTAGATCCTTTTGAGAATGTTTACATGATACCGGTGTGGCTAATTACAACCCAA
GAATTTTTTGTAAGTATTATAATAATTCAGAAGGAAAAAGAAATAATTACACTCATCGAACATTTTGGTTCCATATGGAGAACCAAAG
ACCTGACAGAATATCAATCAAACCATAAGAAAACTACAATGAAACAACTAAACTTTGGACAAAAGATTTTCGAAATAATGTCTTTA
ATAGTAGCCTGGCTGTATATGCTGATGCCTCTTGCTGAGACGTTGTTCAGGAAGTTCATCTTAGACCAAGAAGCAGAGTTGATGTTG
CCGTACGCTTCCGTCTACCCTTTTGCAGTCGACAGTTGGGCTACGTATTTGGGGGTTCTTGCTTTCCAAATATATAACATGTTATTTGT
GATCTTCATGTACTTGGGGAGTAATTTACTATTAGTTTCTCTGAGCACTGGTCTCAGCATTCAGTTTGATTTACTTCAAGCGGATTTG
ATCAACATAAAGCCAACAAATAACAGAGAAAATATAGTTTTTGAAATTAATGACGAA

>LstiOR11 Cluster-2624.48663; orf1 len=639 frame: -2 start:985 end:347 uncharacterized protein LOC114358585 [Ostrinia furnacalis]
ACTCCGTACTTCGAGATAGGATTCATTTTGCACAGTATATTCATGTTTGAAGCTGCGTTTACTATAGTTGTAATCGATATATGGTTTGT
GGTGATGATGATTTTCTTCTGCATGTCTTGCGATAGTACGGTAAAAATTCTTGCAGTAAAAAAAATACGTAATGAGAGCCGACTTGA
ATATGCCGCTCGATTAAACGACAGTCTGAGGAAGTTCTACAAATCACACGTAAAACAAATTGACTTTCTAAACACTTTAAACGCGA
TGTTCAAGTGGCTGGCTTTATTGCCGCTCATCAATGTGGCGTTGTGCGTTTGCAATATACTTCTACTTATGAGTAAGGGAATAGATTT
GACGTTTATATCAAACATATTTCCGGTTGTCGCAGAGTTGTTTGTATACAATTGGTTTGGAGAGCAGATTAAGAGCAAGGCTGAAAA
CTGGAAAACTGCTCTATTGGAGTTCGACTGGATACACTTACTGCCTAAGGATAGAAAATGTTATTACATGATGGTGTGTTATATGCAA
AAAGAATTTGGTATTAAAACTGCGATCGGTACTGATCTCTCACTTTTGACGATGACCACGACGCTCAAATTCAGCTACCAGGCATTT

ACTGTGCTGCAGACTGTGGATGTATGA

>LstiOR12 Cluster-2624.37565; orf1 len=432 frame: -1 start:433 end:2 uncharacterized protein LOC114363237 [Ostrinia furnacalis]

CTTTCCCTCACTACAAAAGTTTGTGCAAGCATTGAAATTAATTTTACTACAATGGAGGGCATATACCCCGAGGAATTCATAAATTCGG
TACTTAAAAGTTTGAGCTACTTTAAAAAGTGCAACATTGATGTTTTCGATTCAAAAAATAGCTTGTATCGGAAATTCTGGTGGTTATT
CAACATACCGAGTTTTATTTTGAACTACATAACTTTGACGATGTACATTGTAAAAATCTTTACGGAAGGCGTAGACCCATTTGAGAA
GATTTACATGATACCGGTGTGGCTAGTCACAACTCAAGAATTTTTTGTATGTATTATAATAATACAGAAGAAAAAAGAAATAAGGAC
AGTCATCGAACATTTAGGTTCCATATGGAGAACCAAAGACCTGTCAGAATATCAATCAAACAATAAGAAAACTAAAATGAAA

>LstiIR25a Cluster-2624.35050; orf1 len=606 frame:1 start:1 end:606 ionotropic receptor [Ostrinia furnacalis]

CTCAAAGAGAGCTTCTGGTTCGCGCTGACGTCGTTCACCCCCCAAGGGGGTGGAGAAGCACCCAAGGCCTTGTCTGGAAGGACG
CTGGTGGCTGCGTACTGGTTGTTCGTGGTGCTGATGCTGGCCACCTTCACGGCCAACTTGGCAGCCTTCCTGACTGTTGAGAGGAT
GCAGACGCCAGTGTCATCGTTGGAGCAGTTGGCGAGACAGTCCAGAATAAACTACACAGTAGTGGAAGGGTCCACCATCCACCAG
TACTTCATCAATATGAAGTTCGCTGAAGACACCTTGTACAGAGTATGGAAGGAGATAACTCTAAACGCAACGTCAGATCAAGCACA
ATACAGGGTTTGGGACTACCCCATCAGAGAGCAGTATGGTCACATACTCTTAGCTATTAACGCTTCAGGCCCAGTACCGGACGCGA
AAACCGGTTTCGAGCAAGTGAACGAACACACGGACGCTGATTTTGCGTTCATCCACGACTCTGCTGAAATAAAGTACGAAGTAAC
CCGGAACTGTAACCTGACGGAAGTGGGCGAGGTCTTCGCGGAGCAGCCCTACGCCATCGCGGTGCAGCAGGGCTCCAGGCTGCA

GGAGCACCTG

>LstiIR40a Cluster-2624.47244; orf1 len=1008 frame: -1 start:1104 end:97 ionotropic receptor 40a [Ostrinia furnacalis]

CGAGACCAACTCAGACTCCTGAACAACTGCTGCTGGTTCACAACTACACTCTTTCTCAGACAATCCTCAAGCAAAGAGCCGTCCA
AAACTCACAAGGCTCGTCTGGTCTCCGTCCTCGTGTCCCTAGGTGCCACCTATGTGATTGGCGACATGTACTCAGCGAACCTCACT
AGCTTGCTGGCGAGGCCTGCGAGGGAGAGACCTATAGGGACCCTGCCAGCGTTGGAGGAGGCCATGAGAGAACGGGGCTACGAG
CTGGTTGTGGAAAGGCATAGCTCTTCTTTAGCTATTTTGGAAAACGGTACCGGCGTATACGGCCGTCTCGCGCGGCTGATGCGTCGG
CAGCGCATCCAACGCGTGCGCAGCGTGGAGGTGGGCGTGCGGCTCGTGCTCACGCGCAGGCACGTCGCCATACTGGGGGGGAGG
GAGACGCTGTACTATGACACGGAGAGATTCGGGTCACACAATTTTCATTTGAGTGAAAAACTCTATACGCGATATTCTGCTATAGCC
CTTCAGATTGGATGTCCATTCTTGGAAACCTTTAATAATGTTGTGATGACCCTATTTGAAGCTGGTATCTTAGCAAAAATGACAACAG
ATGAGTACAAGAACCTTCCAGAACAGTCTAGAAGATCGGAGCCGGTTACAGAGAGTGAT
AAACCAAACAACGAAATCACAGGAGATTCACCTTCGGCATCACAAAGCGGAACGACGCAAGGGGAATCAACAAAAGCGCTGGA
GCCTGTCTCGTTGAGGATGCTGCGAGGCGCGTTCTGCTTGCTTGGGATTGGACATCTTTTGGCAGCTATAGCACTCGGAGTAGAAAT
CCAAATCCACCGGCGATCCAAGAATTTTATCAAGATAATGGAGCCGAATGGAGGGAAGAACATGCCTCGAATGAGGGCTCTGAAG
AAAGCCAGCAAATGCGTCAGGCAAGGAGTCAGGAGAGTCGTCAGGGCTGTCTGCAGATCCATTGATAGGGCTTTAGGGCCTGGTG
TGCAGTAG

>LstiIR75.1 Cluster-2624.42427; orf1 len=924 frame: -1 start:925 end:2 ionotropic receptor 25a [Ostrinia furnacalis]

CGTTTTGTATTCATCATCGAAGTCCACGATTCGGACCTGGCGCACCAGATCGGGCGGGCGCTGAAGGTGGCTGAGGACCAGAGGA
CGGGAGTGAGGGTCAGCGATGCCGTGGTGCAGCTGGACAGGGAGAATGAGGAGGAGAGCTATAGAAGACTCTGCTCAGCACTAT
CCAAAGGCACATCCCTGGTCGTGGACCTGTCCTGGGCGCCGTGGGACATGGCACAACAGCTGTGTTCAGACGCAGGGCTGCCACT
CGTCCGCGCGGCGCTCGGCTCGCAGCAGCTGTTGGCTGCCTTGGACCAGTACTTGGAGAGCAGAAACGCTACCGACGCCGCCATT
CTAACGGAGAGTGAGGGCGAGGTAGACAAAACCCTCTACGAGCTGCTGGGCAGGTCCAACGTCCGACTGTGGGTCCACGCGGGG
CTCACCCGTGACTCAGCCAAGGCCCTCAAGAGTATGAGACCAGAGCCTAGCTTCTACGTCATCGTTGGGGAGAGCGGGTTCGTCAT
GGACACTTACAGAAGGGCAGTAAAAGAGAAGCTGGTCCGCCGCGACTACCGCTGGAACCTCGTCCTCACAGACTACTCTGGAGA
CACCATAGACGTAGCCCAGCTGGTCCTCCCGACCATGATACTCCATGTAGACCAGGTGGAGTGCTGCCGGCTGCTGGGGCTGAGGG
AGGAGTGCAGTTGCCCATCGGATTTGAAGCGAAAGCAGTACATCCTCAACGCCCTCCTCATCTACATAACCGAAACATTCTCCAAG
CTGGAGAGAGAACTGCCTGTGGTGAGCACTAGGGTGGACTGCGACAATGTCCAGGGTTCAGAGATGAACAGTACCAGAGACAGG
ATCCTGAGGCAGTTCAGTGAGGATACTGAGATGAACAATGATACTGTTTTCTTTTGGGATGATGAAAGGTCCGGC

>LstiIR75a Cluster-2624.50941; orf1 len=1422 frame:1 start:1 end:1422 ionotropic receptor 75a-like [Ostrinia furnacalis]

TTCAATTTCAATATTACTTCAAGTACAACGTCTGGCGAACCTGCAGAAGAGAAAGTAATCGACAAAAAGAAACCCAAAGCGATAAT
AGTTGATAAACATGGACTTATAGAAGACTACAGAGAAACCAAAGTGTTGTCTGCTAGAAGACACGACTTAAGAAGACACACGCTC
ACTATGGTCAATGTTATCACGGATAGCAATGATACTAGGAAGCATTTAGATGACAGACTACATCTTCATCAGGACTCCATAACCAAA

ATGTCATACAGCGTCGTAAAGATCTGCTTCGAGATGCTCAACGCCAGTGAGGAACTGATTTTCACCCACACCTGGGGGTATCGTGA
CAAGAACGGTAACTGGCAGGGCATAGTCGACAACCTTATCAAGAAGAAAGCTGATCTTGGTACCCTGACAATCTTCACTCAGGAG
CGCATGCAAGTGGTGGACTACATCGCGATGGTGGGCTCCACTGCGGTCCGCTTCGTCTTCCGCGAACCTCCTCTCTCATACATCTCC
AATATCTTCACTCTGCCGTTCACTGGGGCCGTCTGGCTGGCCATAGTCGTCTGCGTCCTTGGCTGCTCCGTGTTCCTGTACATCACTT
CCAAATGGGAGGCTACTATGAGCATGCATCAATTCCAACTGGATGGCTCCTGGGCTGATGTGATCATTCTAATCATCGGTGCTGTCCT
TCAACAAGGCTGTACACTGGAACCAAGATACGCCGCAGGAAGAAGCGTTACCCTCCTGCTATTCCTGGCGCTGACGATTCTGTACG
CCGCGTATTCAGCCAACATCGTCGTGCTGCTGAGGGCCCCCAGCTCCTCAGTGAGGACGTTACCGGATCTGCTGAACTCCCCACTC
AAACTTGGAGCTAGCGACTTCGAATACAATAGATATTTCTTTAAGAAACTGAAAGATCCTATACGCAAATCGATTTACGACAAGAAA
ATTGCTCCAAAAGGGAAGAAACCGAACTACTACTCGATGGAAGAAGGAGTGGAGAAGATCAGAAAAGGTTTGTTCGCTTTCCAC
ATGGAGCTGAACCCTGGCTATCGCCTGATCCAGGAGACGTACCACGAGGATGAGAAGTGCGACCTGGTGGAGATCGACTACATCA
ACGAGATCGACCCGTGGTTGCCGGGGCAGAAGCGCTCGCCTTTCAAGGACCTCTTCAAAATCAATTTCATCAAAATCCGCGAATCT
GGTATCCAAGCATGCATCCACCACCGTCTCCACGTGCCAAAGCCGAAGTGCTCCGGCACCGTTTCCACCTTCAGCAGCGTGGGCAT
AACGGACATGTATCCTGCGATGCTGGCCACCCTGTACGGCATGCTGCTGGCGCCAGCTGTGCTGCTGCTGGAGATTGCTTACCATCG
TTTAACGGTGCTACGGAAAAGGAAGATAAAGACAAGGAAGTAG

>LstiIR75d Cluster-2624.35781; orf1 len=1812 frame:1 start:16 end:1827 ionotropic receptor 75a-like isoform X1 [Ostrinia furnacalis]

TCTACTGGACTCGTCATCATGGATACAATAAGCTTAATACCAGCATTTTTCCTTTCGAAAAATATATATTTTTTGACAACTTTCCTATG

CTGGAATTCTGAGGAGTTGCACAAGCTGTGGCGCTTGGGGCAGCAGCAGGGCTTGCGGGTACGCGCGATGGCTGCGGGCCCAGC

CACGCCGCCCCTGCCGCCCGACGACCTGCACCGGGAGGGGGTCGTGCTCGACCTCGCCTGCCCCTACGCCGACCACATCATACAA

GCTGCATCAGAGACCCGCGGCTTCAACTACCGATACGCTTGGCTTCTTCTCCACAACTCCTCATTCGACGCTACGTCATTGGACTCC

GTGTTGTCTGGCTCCGTGATCCTGCCCGACGCGGACGTCACATTCGCCTCCGATGACAAGTTGCTGGACGTTTACCGAATAAAGGC

CGATCAGCCTTTGTTGGCCACCACTTTGGGTGTGGTCAGAAACAGTACCCGGCGGGACCTTGAGCAGATGTGGGGGGTTTTAAAA

AGTACTGTCTCGCGGAGAAAGAATTTGAACAACGTGTTTTTGAAAGGTGCCACTATAATAACTCAGCCGCAGAATTTCAAAGGTTG

GAATGACTTGACCGTGAGACATATCGATACTTTTCCGAAGTTGATGTACCCACTGCTAATGCATTGCGCGGAAGACTTGAATTTCCG

CCTGAACCTGCTCCAAGTGGAGTTGTACGGCGACGAGCGCAACGGGTCGTTCGATGGACTGGCGGGGATGCTGCAGCGGCGCGA

CATCGAGGTGGGCGTCACCACGCTGTTCATGCGCCACGACCGGCTCAACGTGATGCACTTCTGCTCCGAGACCCTCGAGCTCAAG

GGTGCGTTCATATTCCGTCAGCCGCCGCAGTCGTCGGTGAACAACGTGTTCCTGCTGCCGTTCAGCCGCGGCGTGTGGGCGGCGA

GCGCGCTTGTGTTCACAGCGGCAGGAGGGCTGCTGGCCGCACTGAGTCGCCCGCGCTGGCTGCGCGACGCGGACCCCGACCTGG

TGCAGCTCAGCGCCGCCGAGGCGTTCACCTTCGCCGTTGGAACCATATGCCAGCAAGGTTGCTACGTGAACCCGCACGCGGTGTC

AGTGCGCATGCTGATGTTCTTCACGCTGCTGGCGTCGCTGTTCACGTTCACGTCGTACTCGGCCAAGATCGTGGCCATCCTGCAGA

CGCCCAGCGACGCCATCCAGACCATCGACGACCTCACGCACTCGCCCATGGCGCTCGGCGTCCAGGAGTCCACGTACAAGCGGGT

CTACTTCGCCGAAAGTGACGACCCCGCCACGCAGCGG

CTGTACCGGCGCAAGCTGCTGCCGCAGGGCGAGCGCGTACCTGAGCATCGTGGACGGCATCGCGCGCGTGCGCAACGGCCTCT

TTGCCTTCCAGGTGGAAGAGAGTTCGGGCTACGACGTCATCAGCAAGACGTTCACGGAGCAGGAGAAGTGCGGCCTGAAGCAGA

TCCAGGCCTTCAAGCTACCCATGGTGGCCGTGCCCATCCTGAAGCACTCGGGCTACAGAGAGCTGTTCGCTGCCAGACTGCGCTG
GCAGCGGGAGACGGGGATCATGGACCGCGAGCGGCGCGTGTGGATGGCCTCCAAGCCGCGCTGCGACTCCGACGGCGGCGGCTT
CCTCAGCGTGCGCCTGTCGGACGTGCTGCCCGCCGTGCAGGTACTGATTTACGGCATGCTGCTGGCTGCCATTCAGCTGTTCGCTG
AGATAGCCCTGCATCGCGCTACGGAGCGGATCAAACGTAAAAACAAACTTAGAGGAAAGAGGGAATAA

>LstiIR76b Cluster-2624.38713; orf1 len=1455 frame:2 start:62 end:1516 ionotropic receptor [Ostrinia furnacalis]
AATGCCATGGCCACTGGAATAGAGCTAATCATATCGTCGATATGCAACGCCACATTTTGCCAACCAGTGTACGACAACCCTTTATTA
GAGAAGCAGGCATCATCATCTATCGATCAGTACCGAGATTTGATTAAAGAAATCAATGGAAAACATCTTAAAATTGGGACTTACAAT
AACCGTCCTATAAGCTGGGTGGAGCGCGGAGAAGATGGCGCTCTCATCGGCCGCGGAGTATCTTTCGTTCTGGTTGACATCCTGCA
GAAGAGGTTCAACTTCACTTACGAGGTGGTGGTGCCGGAAAAAAACTTCGAAATAGGCGGGACCAAGCCGGAGGACTCTTTGATA
GGCCTTGTGAATAATAGCTTAGTGGACATGGCAGCAGCGTTCATACCGAAGTTGACGCGGTTCCACGAGATGGTCAGGTTCTCGTA
CGACCTGGACGAAGGCGTCTGGGTGATGATGCTGAGCCGACCGAAGGAATCTGCAGCCGGCTCGGGACTTCTGGCTCCGTTTAAC
AACGCCGTTTGGTACTTGATCCTCGTAGCAGTGTTGTCTTATGGTCCCTGCATCACGCTGCTGACAAAACTACGGTCCAAGTTGGTC
CCAGATGGCGAGAAGTACATCCCAATGTCACCAAGCTTCTGGTTTGTTTACGGCGCTTTCATCAAACAGGGGACCAATCTCGCCCC
TGAAGCGAACACAACCCGCGTCCTATTTACGACCTGGTGGATCTTCATAATCCTGCTCTCCGCGTTCTACACGGCGAACCTGACCG
CCTTCCTGACGCTGTCCAAGTTCACCCTGGACATCGAGAGTCCCCAGGACCTGTTCAAGAAGAACACCAGATGGGTCTCTGCTGA
AGGCGGCGCGGTGCAGTACGTCGTTTCTAGTCCGAACGAGGACATCTACTATCTCAGCCGAATGATAGCCACTGGTCGAGCTGAAT

TCCGTTCAATGAATAGTCTCTACGAATTTCTTCCACTGGTATCCGGAGGTGCAGTCCTAGTTGAAGAACGAATCGGCATCGACGAGC
TCATGTATGGTGACTACCAACAAAAGGCACGGGAAGGCGTGGCGGAGGCTGAAAGGTGCACTTACGTGGTAGCACCCAACTTATT
CATGAGCAAGCTGCGGGGATTCGCGTACCCTAAGAATAGCCAGCTCGCCCCGCTCTTTGATACCGTCTTGACGTACGTGCTGCAAG
CTGGCATAGTAGATTACCTAGAGCACCGCGACTTGCCTAGCACGAAGATTTGCCCGCTCGACCTCCAATCCAAGGACCGGCAGCTC
CTGAACAGCGATCTCTACATGACCTACATGATCATGGTCACTGGCCTTTCTGCTGCTGTCGCCGTCTTTATTGGAGAGATCATGATAA
AACGCTACGTAATCAAAGACTCCAAACCCAAGAAGCCTAAGCGCAAGAAGACTAAATACGAGAAGAACCGTCATATT

>LstiGR1 Cluster-2624.12537; orf1 len=1380 frame:3 start:156 end:1535 gustatory and odorant receptor 22-like [Ostrinia furnacalis]
TCGAACATGGACGAGGAAAAACAAATGTTCCGGATATACAACACAAACCAGATTAACGGGAAACAAAAAAATACTAACGGAATAA
GAGAAGAATATGACGCGAAGGATATATACGGTCCTGAGATCACGGACAAAGATGGGGCGTTATTGGATGAGCATGACAGTTTCTAT
CACACCACCAAGAGTCTACTAGTGCTATTCCAGATCATGGGCGTTATGCCTATCATGAGGGTACCAAAAGATGCTCAAACCACAAAT
AGAACTACTTTCAACTGGATATCCAAGGCAACTTTATGGGCGTATCTTGTGTGGAGCCTCGAATGTATCATCGTTGTAAGGGTGGGC
AAAGAGCGCTTAGCAACCTTCCAGCAGAACACGAACAAACGCTTCGACGAAGTGATCTACAACATAATCTTCTTGAGCATTCTGAT
ACCGCACTTCCTCCTACCTGTTGCTTCGTGGCGGCACGGGCCGCAGGTGGCGATATTTAAGAACATGTGGACCCATTATCAGCTAAA
ATACCGAAAAATAACCGGGACGCCCATAGTGTTTCCGAACCTGTACATCTTGACATGGGGGCTGTGCGTGTTCTCCTGGGGCCTCA
GCTTTGCAGTGATATTGTCACAACACTACCTGCAAGAGGATTTCGAATTGTGGCATTCGTTCGCCTACTACCACATCATTGCTATGCT
CGACGGCTTCTGCTCTTTATGGTACATAAATTGTAACGCGTTCGGGACGGCTTCCCGGGGTCTAGCCATGAACCTGCACAAAGCTCT

GAAGGCAGAGCACCCGGCCTTGAAGCTGGCGCAGTACCGCCATCTGTGGGTCGACTTGTCGCATATGATGCAGCAACTTGGTCGT
GCATACTCCAACATGTACGGAATATACTGCATGGTCATCTTCTTTACCACCACCATATCCCTCTACGGAGCCCTATCGGAGATACTGG
AGCGAGGTCTAAGCTACAAAGAGATGGGGCTATTCGTAATAGTCGGGTACTGTATGACCCTGCTCTACATTATATGCAATGAGGCAT
ATCACGCTACGAGAAAGGTGGGGTTCGAATTCCAAGTTCGCCTTCTGAACGTGAACCTCGGAGCCATAGACCGCAGCACCCAGCG
AGAAGTGGAAATGTTCCTGGTGGCTATCGCGAAGAACCCTCCTATCATGAACCTTGATGGCTTTACTAATATCAACAGGGAGCTTTT
TGCTGCTAACATCTCCTTCATGTCAACATACCTCATCGTCTTGATGCAGTTCAAACTCACTTTGTTGCGACAAAGTGCAAGGAAAGC
TATCAAGACCGTGGTCAAGGCTATCTTCAATACCACCACTTTGGGTCCTGATGATGAAGATGATGACGTGGAAGAAGAATGA

>LstiGR2 Cluster-2624.4010; orf1 len=1146 frame:1 start:1 end:1146 gustatory receptor for sugar taste 43a-like [Ostrinia furnacalis]
ATTCCAGTTGCATCAGGTTTTCGAGCCCTCGTCATGGACCTATCCGAGCCTTCAGACCGCTCTGTCCGCGCATTCTCAACTACCCGC
CGATGTGTGTGGTTGTGCGATTTCAGCATAGTGATGACGATCTCTTGCCTGGCTGTGTTCAGAGGGCCTAGACGGACGAGAGCTGT
TCTAAGTTGCTTGGGTCATGTACAGAAGATCAATCACAACTTGGATGTGCCACGGCCCACAATTTGGAAGGAAATCAAGAAATATG
TTGCAATCATTTTATATACCACATTTATAGTTTCCACTATTGTAGGCGATTTCGTCTTTATTCTAGCCATAGCCAACTATTTTGACCCGA
CCGGTGAACAAACTAGGATAACATATTTCTATACCGGCTACTACTACGCCTACACAACACTCTTCTTTACTGTCATGCAATTCATCAT
TTGCGCCCTCGAAATATACAGGACTATGAGATCCTTGAATATTCTTCTAGAAGAAGTTGCGCAGGCGGCTTCTCTGAAAAAAACAA
AAATCCTTAATCCTTTTGTCGGAATCAATGTTGTGCAAATGAAGGCATTGAACAAAATTACTGACTCTTTGTCAGTCAATGACAGCG
TATATCCAGGAATTACCAAGAGCTCAAAGACGGCGCACGAGACGATCCGCCATCTCTCTCTGATGTACAGCAGCGCGTGCGAGGCG

GTGCGACAGCTGAACGACTGCTTCGCTCCTGAACTCCTGGCGCTGCTCATGTCATTCCTCCTGCACCTGGTCGTCACTCCGTACAA
CTGCATTGCTAATATTTCCGGTGGAAAGATCAACATGCAGCTATTGACAATGCAGCTGCTCTGGTGCTCAATACACTTCGTCAGCCT
GATCGTCATGGTGGAGCCGTGCCACATCACACAGCGAGAGATGGGCCGCACCAACTTTCTGGTCAGTCAGTTGATGCTGCAAAAC
ACCGATGAACTGGTCACAAACGAGCTGAATGTCTTCGGCCGGTATCTCTACCTGAACGATGTCGTCTACTCGCCTATGGGTATCTGT
GTTCTGAGCAGATCTCTTGTCGCTTCGATCCTCGCCAGCGTCACAACATACCTCGTCATAATGATGCAATTCCAAGCCACAGAGAAT
ATAGTATATCATGGATAA

>LstiGR64a Cluster-2624.34712; orf1 len=1242 frame: -2 start:1728 end:487 gustatory receptor for sugar taste 64a-like [Ostrinia furnacalis]
ATTGTCGTCAAAATAGTCAAATACATAATTATGAGTGAGACGCCGAATTTGTCAAAATTGGAGTCCCGACAGCAAGACAAATTTCTT
CCCACTCTCACACATATCTTCACTGTAGCCCAGTGGTTTGGTATACCTTCATATGGGAACAAATTTGCAATTTGTTGGGCAGTCATTG
TTTTATGCATGTTGACGGTCGTCGAAGGTGCTGCTATTTGGATGATGATAAGGCTTTTGGCTGGTATCGCTAAGCATATAGACGATGG
ACGTGGTCTAACAGCAAGACTTTCTGGGAGCATATTCTATGCGAACGGATTTCTATCCCTTATTCTGTCCTGGAAGTTCATGTACTCG
TGGAAAAGGCTCTCCTTCTATTGGAAGAGAGCTGAGTTAGTAGACGTGTCTTTAGCGATTCCTGATGAAGCTATACAAAGAAAGGT
CATCGTTGTGACTTGCTTTGTCTCAGTGTGTGCTTTTGCAGAACATTTATTAAGTATGATATTGGCTATTGGTATAGATAGCCCTCCAA
TGGATTTTTTAGAGAGGTATATTCTTAATTCTCATGCGTTCCTCATAACACCAAATACCTACTCCTTATGGTCAGCCATACTGATTTTC
TTCGTGAGCAAGATAGCAACAATCCTTTGGAACTTTCAAGACCTTATAATAATCTTGATAAGCATGGGGCTGACGTCGCGGTACCAC
AGATTGAATTCATTTGTAAATATGTGTGTGAAGAATGAAAAAATCAATAGGGATAAAACGAGTGTAACAGAAAAATACGTGCGCAC

CCACCAATGGCGTCGGATTCGCGAGGCGTACGTGCGCCAGGCGGCACTCGTGCGCATGGTCGACGCGAACATCGGCGCGCTGGTG
CTACTGTCCAATGTCAACAATTTTTACTTCATCTGCCTTCAACTGTTCTTGGGCCTAACCAAATCCCAAGGATCCCTTGTAAGCTACC
TATACTACTTCATCTCTCTCGGCTGGCTACTCTTCAGGGCGTGTAGCGTCGTCTTAGCGGCTGCAGATGTACATATCCATTCGAGACG
GGCCCTGGAGTACCTTCAGACATGTCCTGGCACTGGGTTTAATATTGAGATAATGAGACTCAACAACCAGCTAAGCCACGACTTTG
TGGCGTTGAGTGGGATGGGATTTTTCTCGTTGAGCAGACAAACGTTATTAGAGGTGGCCGGGAACATAATAAAATATGAGCTCGTG
CTGATTCAGTATGACAAATGA

>LstiPBP1 Cluster-2624.32152; orf1 len=408 frame:2 start:2 end:409 pheromone binding protein-A [Ostrinia nubilalis]

ATGACATATCCAAAGATGTGGACGTCGAAGATACTGGTGATGATGGTGGCAGCGTGCGTGATGACTGTGATGGTGGACTCCTCGCA
ATCGGTGATGACGTCGATGACAAAGAATTTTATAAAGGCTTATGAAGCATGCGCGAAAGAGTACAATCTCCCAGAGTCCACAGGAC
AGGAACTGATAAACTTCTGGAAGGAAGGTTACACAGTGACGAGCCGTGAAGCCGGCTGCGCCATCCTCTGCCTGTCCTCCAAGCT
GGATCTGCTAGATCCTGAGGGGAAACTCCACCATGGAAACACTGTGGAGTTTGCCAAATCGCATGGATCTGACGATGCAATGGCTC
ACAAGGTTGTGGAAATTCTCCACTCGTGCGAGAAAGCGGCTGCACCTAATGAAGACATGTGTCTG

>LstiPBP2 Cluster-2624.32604; orf1 len=492 frame:2 start:2 end:493 general odorant-binding protein 1-like [Ostrinia furnacalis] > BBB15981.1 pheromone binding protein-C [Ostrinia nubilalis]

AAAGGGTTTGTGGGTGTCCCTATCACGCTTGCACTAGTCCTGATAGGAGTAAGGGAGATAGATATGGTACCGGAGGCGATGAAGCA

GTTGACCCATGGATTCCTTAAAGTTTTAGAGGAATGTAAAACAGAGATGAATCTATCAGAAGGCGTGATATCTGATCTCTACCACCT
TTGGCGGGAGGAGTACGACCAGATCAGCAGAGACGCCGGCTGCGTCATCCACTGCATGAGCAAGAAGCTGCAGCTCTTCGGGGA
CGACGGGAAGATACACCATGAGAACATCAAGGAGTTTGCTGTCAAGAATGGTGCTGAAGAAAAAGTAGCAGCCCAACTGCTGAG
CCTAGCCCACGAATGCGAGAAAAAGCAGGAAGGCATAGAAGACGAGTGCGAGAGAACCTTGGAGGTGGCCAAGTGCTTCCGAA
GAGACGTGAAGCAGATCGACTGGGTGCCCAAGATGGAAGTACTCATGACTGAGGTTATAGAAGCATGA

>LstiPBP3 Cluster-2624.31900; orf1 len=282 frame:1 start:187 end:468 pheromone binding protein female 2, partial [Loxostege sticticalis]
ATTCTTGATTTTGCGCACAAGCAGGACCTGCTGCACTATGAACAGTACCGCCTGCATCACCAGAACGCCTACCAGTTCGCGAAGGA
CCATGGTGCTGACGAAGCCACTGCCAAGCAGATAATGACCATCGTCCACGAATGCGAGGAGAAGTTCGCGACCAACGAGGACCAC
TGCGCGCGCGCCATGGAGGTGTCCCGCTGCTTCCGCGACCACATGCACCGGTTGCAGTGGGCTCCGTCGGTGGACGTGCTGGTCG
GAGAGATCCTGGTTGAGATGGCTTAG

>LstiGOBP2 Cluster-2624.32578; orf1 len=501 frame:2 start:611 end:1111 general odorant binding protein 2 [Loxostege sticticalis]
AAGGAACGGTACAACATGTCTTCGGCTTGGATTCTCTTGGGGCTGGTGATGGCTGCCGTGTCTTCGGTGAGAGGAACTGCTGAGGT
GATGAGCCATGTGACTGCTCACTTCGGGAAGGCTCTAGAAGAGTGCAGGACTGAGTCGGGTCTCTCGCCCGAGATCCTGGAGGAG
TTCCAGCACTTCTGGAGCGAGGACTTCGAGGTGGTCCACCGCGAGCTGGGCTGCGCCATCATCTGCATGTCCAACAAGTTCTCCCT
GCTCCAGGACGACACGCGCATCCACCACGTGAACATGCATGATTACGTCAAGAGCTTCCCCAACGGCGAGGTCCTATCAGAAAAG

ATGGTGAGCCTCCTTCACAACTGCGAGACGCAATACAACGATATGACCGACGACTGCGATAGGACTGTCAAGGTGGCTGCCTGCTT
CAAGGCTGATGCGAAGAAGGAAGGTATCGCGCCCGAAGTGGCTATGATAGAAGCCGTCATGGAAAAATATTAG

>LstiOBP1 Cluster-2624.3670; orf1 len=513 frame:2 start:11 end:523 odorant binding protein IA-like precursor [Mus musculus] > AIA65154.1 odorant binding protein 6 [Mus musculus domesticus]

AAAAGAAAGTCTTGTACCATCATGGCAAAATTTCTGCTGCTTGCTTTGACATTTGGACTGGCACATGCTGCGATGGAAGGACCATG
GAAAACTGTTGCTATTGCTGCTGATAGAGTAGACAAGATAGAGAGAGGTGGAGAACTGAGAATCTATTGTCGTAGCCTTACCTGTG
AAAAGGAATGCAAGGAAATGAAAGTCACATTTTATGTACTTGAAAATGGACAGTGCTCATTGACCACAATCACTGGGTATTTGCAA
GAAGATGGCAAGACCTACAAAACTCAGTATCAAGGGGATAATCATTATGAACTTGTGAAGGAGACACCTGAGAACTTAGTATTTTA
TAGTGAGAATGTGGACAGAGCTGACCGGAAAACAAAATTGATATTTGTTCTTGGCAATAAACCTTTAACTTCTGAGGAAAATGAAA
GACTTGTGAAATATGCTGTGTCAAGCCACATTCCTCCTGAAAACATTAGACATGTCCTGGGTACAGATACCTGTCCTGAGTAA

>LstiOBP2 Cluster-2624.32099; orf1 len=462 frame:2 start:2 end:463 pheromone binding protein female 1, partial [Loxostege sticticalis]

GACCCGTTCACTGACATACTAGGACATCATACAATGTTGAAAGTGGCACTTACAGCAGCTATTTTAGCCCTCTCTCTAGGAGGTTCG
TACAGCAGTCCAGCTCAGGATAAATCTGCTAAACCCAAAGATGACAGTACGGCGAAGGATATGATGATGGACCAGGGAGACATGA
CTTCGGGAGAGGCTGTGAAGAATAGGGTAGATTTGAATGAAATAATGAACCAGTGCAATGATTCGTTCCGGACTGAAATGGCGTAC
ATAGAGGCTCTCAATGAAAGCGGCAGCTTTCCAGACGAGACCGATCGAACACCTAAATGCTACGTCCGCTGCGTGCTGGAGCACT

CCGGAGTGGCGTCGGAGGATGGCGTGTTCGACGCTGCGCGCGGCTGAAGTGTTCGCAGGAGAGCGTGGGGGAGAACCATGA
CCGATCTTCAGGACCTGGCTGCCGCTTGCGCTGAT

>LstiOBP3 Cluster-2624.33469; orf1 len=447 frame: -2 start:496 end:50 odorant binding protein 5 [Ostrinia furnacalis]

GGAAAACTATTTACATCTAGGAAGATGCTTCGTGTATCAATTTTTTTGGTGACTTTATGTGTGGGATATTTGAGTGCTGAAAAGCCAG
TTGTATACTTGGTCCCCGAGAAGATAGTCCAAACACTCCCACAGGTGATGGAATGCATTGCCGAAACTGGAGTCGACTTCGAAGTA
TTGAACAAACTGCGAACTGGTGTTTCAGCTGATAGCAAAGACCCGAGGATCGCTAAATTCGCCCACTGTGGTATGAAAAAAAACG
GGCTTGCCAAGAAAAATGGTCGTCCAGAAATTGATAAATTAATGGCCTTTTATCCTAGCAGTGCAGACAAGGCAGCCATCAGAAAG
GTCATGGAAGAATGCGACAAAGAAGGAAAAAACCCAGTGGACACTTCTTACAAATTCGCGCAATGCTTCGAAAAGAATGCGCCCG
TGAAAGTAATGTTTTAA

>LstiOBP4 Cluster-2624.32637; orf1 len=438 frame: -1 start:745 end:308 odorant binding protein 12 [Ostrinia furnacalis]

TTAAGGCATAGTAAAATAATGCTCCTTGTAATATTAGCCAAATTTTTAGTGATACTGGCATCATGCGAGGCGATGACAATGAAACAGA
TAAAAAATACCGGCAAGATGATGAGGAAATCCTGCCAGCCGAAAAACAATGCCGCTGATGAAAAAATCGACCCACTTAACGAAGG
CATTTTCATAGACGAGAAAGAAGTCAAATGCTACATGGCTTGCATCATGAAAATGGCTAACACAATAAAAAATGGGAAGCCAAATT
ACGAAGCGGCCATAAAGCAGATCGACCTGCTCCTCCCTGAAGAAATCAAGGCACCAGCCAAAGAGGCTGTGACGGCGTGCAAGA
AAGTTCCGGACGCATACAAAGACACGTGCGATGCAGCCTTCCACGTGTCCAAATGCATCTACGAGCACAATCCTAGCATATTCTTTT

TCCCGTAA

>LstiOBP5 Cluster-2624.32825; orf1 len=378 frame: -1 start:380 end:3 uncharacterized protein LOC114366134 [Ostrinia furnacalis]

AAAAATATTTTTAAAATAATGGCTAAGTTCACGATTTTGTGCCTAGGTGTTTTGGCCGCCGCCATAAGCAGTGCTAGAGCACTTACC
CCAGAGGAGCTGACGAAAATCGAAGGCGACATGTTGGTCCACGTCCAAGATTGTGCCAAGAAGTTTGACGTAGATGAATCTGACC
TGAAGAAAGCCAAAGAAGAGGAGAACATTGACGGTGTCGACCCTTGCCTGATAGGCTGCGTCTTCAAGAATATTAAGCTGGTTAA
CGACAAGGGTTTATACGACCCTGATGTAGCCATCGAGAGCAGCAAGAGCTACCTGTCCGATGATGAGGACAAGGCCAAGTTCGCA
GAAATCGCCAAGGACTGTGCTTCAGTGAACGACGAG

>LstiOBP6 Cluster-2624.33606; orf1 len=345 frame: -1 start:377 end:33 uncharacterized protein LOC114366121 [Ostrinia furnacalis] > BAV56796.1 odorant binding protein 9 [Ostrinia furnacalis]

GGCATGGAGTGTATAGGAGACAATCCAATCACTGAGCAGGACATCAACGACTTACGAGCGAAGAAGGCGCCTTCAGGACCTGGTG
GTCCCTGTTTCCTAGCTTGTATCCAGAGAAAGATTGGTGTGATGGACGAACATGGTATGATGCAAAACGAGAACGCGTTGGAACTT
GCAAAAAAAGTATTCCAAGATGAAGAAGAGCTCAAAATTATTGCCGACTACCTACATTCGTGCAAAAGTGTGAATGACGTCTCTGT
CAGTGATGGAGAAAAGGGCTGTGAGCGTGCAATGGCCGCCTTCAAATGTATGATCACAAATGCACCAGCGTTCGGCATCGAAGTC
TAG

>LstiOBP7 Cluster-2624.36680; orf1 len=327 frame: -1 start:329 end:3 uncharacterized protein LOC114366121 [Ostrinia furnacalis] > BAV56796.1 odorant binding protein 9 [Ostrinia furnacalis]

GACTCCAAAATGTTTTGTCGTGTTATTTTGCTGTCTAGTGTCTATTTTTTGGCTTTGACCCCCTATTCTATTAATGCCATGACTGAAGC
ACAGAAAGAAATGATCAAGCAGCACTTCGAACAACTTGGCATGGAGTGTATAGGAGACAATCCAATCACTGAGCAGGACATCAAC
GACTTACGAGCGAAGCAGGCGCCTTCAGGACCTGGCGGGCCATGTTTTCTAGCTTGTATCATGAGAAAGATTGGTGTGATGGACGA
ACACGGTATGATGCAAAACGAGAACGCGTTGGAACTTGCAAAAAAAGTATTCCAGGATGAAGAAGAG

>LstiOBP8 Cluster-2624.38940; orf1 len=246 frame: -3 start:259 end:14 uncharacterized protein LOC114363418 isoform X1 [Ostrinia furnacalis]

GTGGACTCTAAGGGAATCCCGCGCGAGGCTGAGCTGTGGGGCAAGGTGCAGTCGTCGGTGACTTCTCAGCAGTCGCGAGCCGCG
CTAAGAGACCAGATAAGGGCCTGCTTCCAGGAGTTGCAGTCGGATGCCGAAGACAACGGCTGTTCCTATTCAAACAAACTGGAAC
GATGTTTGATGCTGCGCTTCTCCGACCGCCTGAAGGCAGATAGAAGCAAGACGCAAGCAAACAACCAGAAAACTTAA

>LstiCSP1 Cluster-2624.32786; orf1 len=561 frame:1 start:937 end:1497 chemosensory protein 14 [Ostrinia furnacalis]

ACCACTAGCTTGCTTCGCACGTGCAATTGCAACAGCGGTCGGCGAGTCAGTCGATACAATAGAAGCAATCAGAACGTCGGGAGAC
GGTCATCTGTCACCATGTGGTTCAAGCTTATATTCCTGGCAACATACGTATCGGTTGTCGTCACTGACATGGGTCCTCCAGGGTTCC
AAAGGACGTTCAGCGAAGGGGTCACGAGTAAAGGGTACAGAGTGGTGTACGGCGATGAAGATTTGACGGTAATCAACGAGGTGG
TGGGGAGTATGGACAAGGATGACATCCTGAAGGCTAAAGTGCACCTCAACGAAGGGATAAAGCCGTTGCCGGCTGAGGACGTCA

AGTGCCTGATGTCTGTCGATCGATACTGCTCGATGGAGATGCGGAAAGTCAAGGGGATACTCATCCAGGCCCTCAAAGATGATTGC
GAGAAGTGTTCGATCCCAGAGAAAGAAAGTGCTGGAAAGGTGGCAGCATCCATGATGGCGCATGACCCAGTCGGCTGGAAGCTTT
TCCTGACTAGGTACGACGGACTGTCCAAGGTTCAGAGGATTCTAGGATAA

>LstiCSP2 Cluster-2624.39782; orf1 len=411 frame:3 start:498 end:908 chemosensory protein 5 [Conogethes punctiferalis]
TGCATGAACTTTTTAGAGGATCACCATTTGCAAAAAATCTTCATCATGAGGGCCATAGTACTCCTATCTTGCCTGGTCATGGTCTACG
CAGCAGACAAGTACAGCTCCAAATACGACAACTTTGACGTGGAGACCCTGATCTCCAATGACAGACTCCTGAAGGCATACATCAA
CTGCTTCTTGGAAAAAGGGCGGTGCACACCTGAAGGAGCTGATTTCAGAAAGGCACTCCCAGAAGCGGTAGAGACCACCTGCGC
GAAATGTACAGAAAAACAAAAGAACAACATCAGGAAGGTCATCAGGGCGATCCAACAGAAACACCCCAAGCAGTGGGACGAGC
TGGTAAACAAGACCGACCCCTCTGGCAAGCACCGCGCCGACTTCGACAAGTTCATCCAGGGTAGTAGCTAG

>LstiCSP3 Cluster-2624.33276; orf1 len=411 frame:2 start:440 end:850 allergen Tha p 1-like [Ostrinia furnacalis] > XP_028174916.1 allergen Tha p 1-like [Ostrinia furnacalis] > BAV56808.1 chemosensory protein 4 [Ostrinia furnacalis]
TTTCTCCATTCCAGATCCACAATGAAGTCACTCGTCCTAGTAGCTTTATCTCTCCTGGTGGCGGTTGCCTGGGCCCGTCCCGGCGCC
ACCTACACCGACAAGTGGGACCACATCAACGTGGACGAGATCCTGGAATCCCAGAGACTCCTCCGAGGATACGTTGACTGCCTCC
TCGACAAAGGCCGCTGCACCCCCGACGGAAAAGCCCTGAAGGAAACTCTTCCTGACGCCCTAGAACATGACTGCTCAAAATGCAC
GGCGAAACAAAAGGAGTCTTCCGAAAAAGTTATCAGGCACCTCATCAACAAACAACCAGATTTCTGGAAGGAGCTTTCCACCAAA

TACGACCCCGAAAACATCTACCAAGAGAAATACAAGGACAAAATCGAAGAAGTCAAGAGCAAGAACTAA

>LstiCSP4 Cluster-2624.32120; orf1 len=408 frame:1 start:1 end:408 uncharacterized protein LOC114363440 [Ostrinia furnacalis]

ACAGTACCTGAGAACGGCGACCGAGCACCATCAAACAAAATGAAAACCATCGTAGCACTG

TGCGCACTTATGGCGGTAGCGCTGGCCCGCCCTGAGGAGACCTACAGCGACACGTGGGACAACTTCAACGCGCAGGAGTTGGTCG

ACAACGTCCGCCTGCTGAAGAACTACGGCAAGTGCTTCCTCGACCAGGGCCCGTGCACGTCTGAGGGTCAGGACTTCAAGAAGA

GGATCCCTGAAGCCCTCAAGACCGACTGTGGCAAGTGCTCCCCGAAACAACGCGAGCTGATCAGGACTGTGGTCAAGGGCTTCC

AGGCCAAGCTGCCTGAGGTCTGGGCTGAGCTGGTCAAGAAGCACGACCCTGAGGGCACATACAAGGATTCCTTCGAAGCCTTCCT

CAACTCCAAC

>LstiCSP5 Cluster-4147.0; orf1 len=444 frame: -2 start:460 end:17 chemosensory protein 10 [Ostrinia furnacalis]

TTGTGGCCAGACTGGTTACTAGTCAGTGCAACGTCACCTTTTGCAGCGAGTACAAGAGCAATTATCAAACACACAATCAACATGAA

GTTCATCGTAGTTCTTGCTGTCATCGTGGGCCTGGCCATGGCTGATGAGAAGTACACCAGTGAGAACGACAACTTCGATGTGGATG

CCCTGGTGGCCAACATCGACGAGCTGAAGAAGTTCTCCGGCTGCTTCCTGGACATCAACGACTGTGATGCTGTGGCCGCTGATTTT

AAGAAAGACATTCCTGAAGCTTTCCAGCAGGCCTGCGCCAAATGTACCGACGCCCAGAAGCACATATTCAAGAAGTTCATTGCTG

GCCTGAAGGAGAAGTTGCCCCATGACTACGAAGCTTTCATGAAGAAGTACGACCCTGAGAGCAAATACTACCCGGCTTTGGAAAA

GGTTATCAATGTTTAG

>LstiCSP6 Cluster-2624.25708; orf1 len=375 frame:3 start:105 end:479 chemosensory protein 10 [Conogethes pinicolalis]

GAACCACTCAGTGCAAGGAAAGTAAAAGTGCTACAGTGGGAGAGCACCGCTAACATGCAGATCGCCCTGGTTCTAGTGATGCTCG
CAGCATGCGCGTACGCAGCGGAGACGCCGCGGCCGCAGGTGTCCGACACCGCCTTGGAGGATGCCCTCAACGACAAGCGCTTCAT
CCAGAGGCAGCTCAAGTGCGCGCTGGGCGAGGCGCCTTGCGACCCCATCGGGAAGAGGCTAAAAACTCTTGCGCCACTGGTGCT
GCGTGGTGCCTGCCCCCAGTGCTCCCCGCAAGAAACGAAGCAAATCCAGCGCACGTTGTCGTACGTCCAGCGGAATTACCCGCAA
CAGTGGGCCAAGATCGTGCGCCAGTACGCGGGCTAA

>LstiCSP7 Cluster-2624.32727; orf1 len=273 frame: -3 start:275 end:3 uncharacterized protein LOC114363440 [Ostrinia furnacalis]

CAAACCATTGCTTTTTTCCAGAAAATGAAAACCATCGTAGCACTGTGCGCACTTATGGCGGTAGCGCTGGCCCGCCCTGAGGAGAC
CTACAGCGACACGTGGGACAACTTCAACGCGCAGGAGTTGGTCGACAACGTCCGCCTGCTGAAGAACTACGGCAAGTGCTTCCTC
GACCAGGGCCCGTGCACGTCTGAGGGTCAGGACTTCAAGAAGAGGATCCCTGAAGCCCTCAAGACCGACTGTGGCAAGTGCTCC
CCGAAACAACGCGAGCTG

>LstiCSP8 Cluster-2624.30893; orf1 len=213 frame: -1 start:419 end:207 uncharacterized protein LOC114363443 [Ostrinia furnacalis]

GAGTTGAAAGAGCATCTGCAAGAAGCGATCGAGACCGGCTGCGAGAAGTGTACTGAAGCCCAAGAGAAGGGCGCATACACCGCC
ATCGAGTACCTGATCAAGAATGAGCTGGACATCTGGAGGCAGCTGGCAGCCAAGTTCGATCCTGAAGGCAAATGGAGGAAGACGT

ACGAGGACCGTGCCAGAGCCAACGGCATCGTCATCCCTGAGTAA

>LstiCSP9 Cluster-2624.23083; orf1 len=201 frame: -2 start:810 end:610 chemosensory protein 14 [Ostrinia furnacalis]

CGATACTGCTCGATGGAGATGCGGAAAGTCAAGGGGATACTCATCCAGGCCCTCAAAGATGATTGCGAGAAGTGTTCAATCCCAGA
GAAAGAGAGTGCAGGAAAGGTAGCTGCATCGATGATGGCGCATGACCCAGTCGGCTGGAAGCTTTTCCTGACTAGGTACGACGGA
CTGTCCAAGGTTCAGAGGATTCTAGGATAA

>LstiCSP10 Cluster-2624.33314; orf1 len=153 frame:1 start:1 end:153 chemosensory protein [Conogethes punctiferalis]

GAGACCCAGCGTACTGGCACCCGCCGCGTCATCGGCCACCTCATCAACAACGAGCCCGAGTACTGGGCCCAGCTGACCGCCAAGT
ACGACCCCCAGCACAAGTACGTCGTCAAGTACGAGGACGAACTCAGGATTGCCAAGGCCCAGAATTAG

>LstiCSP11 Cluster-2624.33335; orf1 len=327 frame: -1 start:380 end:54 chemosensory protein 11 [Ostrinia furnacalis]

AAAGCAGAAGAAAAGTATACTGATCGATTTGATGACATCAATTTTGAAGAAATCCTCGCGAATAGGCGGCTGCTGGTGCCATACCT
CAAGTGTGTGCTGGATAAGGGAAGGTGCACTCCTGAAGGGAAGGAGTTAAAAGTTCACATTCAAGATGCGATGCAGACTGCGTGC
GCGAAATGCACCGACAAACAAAAGGTCGGTGCAAGGAGGGTGGTCAACCACATCCGCGAGAAGGAACAGGAATACTGGGAAGA
GTTGTTGAGCAAGTACGACCCTAAGGGCGAATACAAAAGCATCTACGAGCCTTTCCTTGCTGGTAAAGAATAA

>LstiCSP12 Cluster-2624.30641; orf1 len=138 frame: -1 start:506 end:369 putative odorant-binding protein A10 [Ostrinia furnacalis] > XP_028174947.1 putative odorant-binding protein A10 [Ostrinia furnacalis] > BAV56807.1 chemosensory protein 3 [Ostrinia furnacalis]

GATCTCATAGCCACGAACTGCGGGAAATGCACCCCAGAACAGAAGAAGAGGCACGAAGAAGTAAATAAGTTCATCCTGGAGAAAT
ACCCCACTGAGTACAACGCAGTTGTAAACAAGTACAGACCTAAGGCTGAATAA

>LstiCSP13 Cluster-2624.30893; orf1 len=213 frame: -1 start:419 end:207 uncharacterized protein LOC114363443 [Ostrinia furnacalis]

GAGTTGAAAGAGCATCTGCAAGAAGCGATCGAGACCGGCTGCGAGAAGTGTACTGAAGCCCAAGAGAAGGGCGCATACACCGCC
ATCGAGTACCTGATCAAGAATGAGCTGGACATCTGGAGGCAGCTGGCAGCCAAGTTCGATCCTGAAGGCAAATGGAGGAAGACGT
ACGAGGACCGTGCCAGAGCCAACGGCATCGTCATCCCTGAGTAA

S5 Protein sequences of all olfactory genes constructed the phylogenetic tree

OR:

>LstiPR1

MNLVSVSRQLMVYQKSYTEVSRDFVTKVHLFNWKDDSEYAMEIHILVHKISHFFVMYIHGLMFIGLSMFNLTPLYNNYSNDAFTKRLH
GNATLEHAVYYSLPFDYTTQIPGYIVVFTYNWFISLVCSINFCSVDTYMSLLVFHLWGHLKILIHNLEHIPKPSGLKSSANINGATQTERYN

EDETQQVFERLRELIKHHCLIRNFISIMSSAFGYVLLVYLGFHQVCGCILLLECSSLVNIT

>LstiOR1

MFMNSKFQQLLGTMWENLRKFGLGHCDLPTMVWNVAFMLRGFTLNIDSRFTGRIPKIFYITTIIIAFCYLYSYFFSMLWFVFVRCIETGD

VTAAMIVFPLGITSEIGIAKFIYTCVYRKKVRQLLQQYLEYDSQIPQGSRLSRHLLQALRNVKRRALIYWIFIVSNGTLYILQPLVMPGRVP

MEEVFVLYGLEPELETPNYEITYVLCTFGSVCTCYLTSNVAAFLIIVSGYVESQLLALSEEILNVWDDAELEYKVIDNADEEEFENKEKY

DAINESVKIRLKDIVKGHTTNINLLLQVEDIYRGAFAFEFCILSVGLIAELLGGLENTYMEVPFAIIQVGMDCLIGQRVMDACDTFESAVY

SCKWERFNEANMKTVFMMLMNSQKTLTLTAGGIAVLNFVCLMSVFRSIYSAFTTLQSVM

>LstiOR2

MPEKSYGTVKSNLREELNYINSMGSKIFLYPFSGRSKLVDICYLFVCFLVVVTATQLLTALLVTDLKEWIEIVNVAPNLGVVLMTLLKYT

KVHNNQHVYKKIFKHFSDDLWDVVFDSYDHKKIVIRYTAIAKYATRFLFYYSVPLVVFVDSFPRIIMYLENEIIGNENPQYLYPFDGWYP

FDKVNWYYTAYLWESFMTFIVVCVYAFSNMIHASYTSFICMELEILGVSIKDLITPDDVTNITNHLKVQEIHSHIKRKLKTIIRRHQFLAQ

LASELNIVLGDMMLLNYIFGSVFITLTIFTATVVDNMYKSLRYFFMFCSLIVEIFFNCMIGQVLSNHSEQLTDAIYSADWPFADNETKVML

LILMRRTQKPFEYTANGYLAMNLNSFSGVCSMSYQLFNLIRTAYSK

>LstiOR3

MDPNKLSHSFYKITYVWKLLGMWSGKSSSKNLRIYSWLFVTLYYIMYNFFYTLSLVCAPRAVDTIGVGVYYFTTLCGLAEIVMILRNRQ

KIISVFETMDCKEFQGNNRQTNEYLRQFKITFSRYFKVYAAYCFISSHTFLMFLPLFNYFFQHKELEMPIWEYYFLTNATRNKYFFYLYTY

QSMGMIATIFNHIVYNVFLFGILSVAVSQSKVLNWNIANIRLADEDILKSTEEKERLYLNKLYNCLKHYEIILKYCEDVQDLTSFLISLNYG

LSVFTLCFSMYMFLLPTNSNTLVYMGFYLSAILIKNFVPSYLGSELTNESNNLRFAVYSCDWVPRSKNFKTCLMIFVERARRPLLIKGLK
VVPLSLATFTSIVKTAYSFFTLLRGAQDQLI
>LstiOR4
MDLFNYIKRVFRDAKSRLQENSYESLLSLVNFVPSVAGFSIRGNTIFVPFWILHLSLLFYIYGVGCAVYQIKYAEDARDFIKSFVNVSLLVL
IANNSHWFLQKRSLLKTALKEISESDVMATANESFRQKHERSVQKIKRILFIFYGFNLLNATFVYLPHRADVLNSYSMTPCFGMEPLTSSP
NREICMTLLCIQEITIMVVVLNYQALLLLLIAYTALMYTLLADEIMTLNNFDRETYYNNPTVKLILPDLVKRHAILLSIIDKLKALYSGSIG
VNFGSNAVCISLFFYLPLQEWLQFMPVLVYCFLVFFLYCFLCQRLTNAAELFEMSVYACGWENFELKEKKAIY
>LstiOR5
MLSAVCCIKVATFVAWQKDWNSIFEYVSKLEKRQLSQKDKVTDAIIGGYIKYSRRLMFFYWALVAATITTICVAPLVGFLSSAEYRERIRN
GTAPYPESVSAWMPFDRTRGLGYWVTIISHNSIMCYGATIVANYDGNAMVLMTFFAGQLKILKENCSRLFDDEEETTCEDAIKKIRDCH
YHHQLLIKYTKVFNGLLSPVMFTYVIICSLMICASAIQITKGGTTTVQRIWISEYVIALIAQLFLYCWHSSEVLARSDEVEDGVYASAWW
SRSIRVRRCVLLLAGQLRTSVVFTAGPFTEMTLPTFVAILKASYSYYTLLVNKDD
>LstiOR6
MFLMYWKRVQTKSIVDQINSDHEAFNHLPRKQQDIAFLYIKAGVRNVERIWAPLVSVAIMMFPGMAVILTLYSYTFNDIPKKYMIHELNP
PFSTDPEDMSRSPYFEVLFVYETGAAIICVLNYTAYDGLFGVATNHACMKMSLCCVKLNDAFACEDQEEMYKGVLAFIEEQQKMYKFV
DLIQEIFNIWLFAILMSTMIQIGSLLFHISAGYGFDLRYTLFSFTSVVHIFLPCKHAATLKSMSTEMATMIYISGWERSRDRRVLRMIPFML
ARAQVPNYITAFGMFVFDMELF

>LstiOR7

MFPDPSLNHFFVPHDSLIEDIIFVSMSTGLEPMEKSPNFEFGYALVAAAVWFLCYVPANVTAVLIVVAGYIEAQMLALTQELLHVWSDAE

QYYKNLNFTVLKRGSFVDANYKKRVLNEFVTMRLIDIIQKHARNVYLLQLLEDVFRGAIALEFVLLVLGLIAELLGGLENTFLEMPYAF

VQVAMDCWTGQRVMDASAQFAAAVYACQWEMFDVANMKIVLLMLASAQKTMKLSAGGVTMLSFESLMAVVRSIYSAYTTLQSTLK

MNVHIK

>LstiOR8

MSEELIFDKSLQNIKFIFRLSGLNIDHKKRTFKQNCIYLFNLLWHQTDLLSALSWVLCGMFTGKVFTELTFVAPCSVFGILADTKGVLFIL

QERKIYSLMNNLRDLEIKAKDFVGTRRDSLIKPDIYLLDIVIKVLNILNGLMIVVFDMCPPIFIAIKYFTTGQLELMLPFLDVYFFDGFDLR

YWPFLYAHQVWSVCVSLLEICATDYFFFTCCTHIRIQFKLLQHQFQDILTNRSITADSTNQMPIQAQFRELVKWHQGIIT

>LstiOR9

MEPSKHSQIFNLTITLWKIFGLWSDKSSSIWFRLYSYIFVTVISFGYFFFYTLSLFYTPRTLEIFVSQAIYYFITFSGIIRVVAIWIKHPQLISNF

KMMDCKEFRENDGKTNEYIRQFENGYKRYFRAYAIHCSGSAYTFILVIPLLNHFFRQEELKLPICGNYFLTDDQRNKYFLYWYLYQTAG

MVGTVTNNITSHTLICGLNYMAITQFKILNYNIANIMLDDDDIPKNIQAKERIYLNKLLKCLRHYEIILE

>LstiOR10

MDGKYPEEFINSLLISLSYFKKCNIDVIHSNNSLCGKIWWFFNIPSFILN

YITLTMHIIKIFTEGVDPFENVYMIPVWLITTQEFFVSIIIIQKEKEIIT

LIEHFGSIWRTKDLTEYQSNHKKTTMKQLNFGQKIFEIMSLIVAWLYMLM

PLAETLFRKFILDQEAELMLPYASVYPFAVDSWATYLGVLAFQIYNMLFV

IFMYLGSNLLLVSLSTGLSIQFDLLQADLINIKPTNNRENIVFEINDE

>LstiOR11

MFEAAFTIVVIDIWFVVMMIFFCMSCDSTVKILAVKKIRNESRLEYAARLNDSLRKFYKSHVKQIDFLNTLNAMFKWLALLPLINVALC

VCNILLLMSKGIDLTFISNIFPVVAELFVYNWFGEQIKSKAENWKTALLEFDWIHLLPKDRKCYYMMVCYMQKEFGIKTAIGTDLSLLT

MTTTLKFSYQAFTVLQTVDV

>LstiOR12

LSLTTKVCASIEINFTTMEGIYPEEFINSVLKSLSYFKKCNIDVFDSKNSLYRKFWWLFNIPSFILNYITLTMYIVKIFTEGVDPFEKIYMIPV

WLVTTQEFFVCIIIIQKKKEIRTVIEHLGSIWRTKDLSEYQSNNKKTKMK

>SexiOR13

MGIMMSKKFLKMDLKLSTVKIFSDGSDLEGIENVEDIVYLRILKKIMWVIDGWPKEANKSQFFRYYICILDMVSLIPGTLYLKINTGKIPS

FELGHTYITVFMNAIAALRTVLVLTKRYNEIIFYFLKEVHLFNFRRKSKYAYETHILVHKISHFFTMYVFMLMCCGILLFNLTPIYNSYAAG

MFKDERPPNATFDHAVYFALPFDTATNFKGYVVVSLYNWYISITCSTYFCIIDLTIFIMVFHLWGHMRVLTYNLEHFPKPASVMAAADDP

NAYKNCENKYNEEESVEVFLRLRDCIKIHSLVINFSSMMADSFGWTLLVYLFFHQVSGCLLLLECSQLDTAALARYGPLTIIIFQQLIQLSI

IFELLGSSNDRLIDAVYSVPWEYMDTANRKNVFFMLRQSHRSMNLKACSMVTVGVQTMIAILKTSFSYFVMLRTVADEEE

>PsauOR4

MKILSDLTDLEGVDKTEDIQYMKILRRSMWIIDSWPRAPQQSRTYRFYCIFKMVCCFVPGIIYLRNNTGKLSSFEMGHTYITVFMNGIAL

SRAMMIVGPKYNEFLFYFLNEIHLFNYRNKSDYSYKTHVLVHKISHFFTVYLLILMCLGLTLFNLTPMYNCYSNGMYRFERPENATFDH

AVFYSLPFDYTTNVKGYMALFTFNWYISVCCSSYFCVVDLTISLLVFHLWGHMRILKYNLENIPKPASVLVDAEENRAVGKEHKYTEEE

AREVHRRLRDNIHYHSIIIDFQSKMSDCFGEVLLVYFLFHQVSECLLMLECSQMNQEALLRYGPLTVVIFQQLIQLSIIFELLGSSNDKLID

SVYCVPWEHMDTKDRKLVLVMLRQVQRSMNLKAMGILTVSVQTMVTILKTSFSYFVMLRTVAEED

>SlitOR13

MDIKLSSVKFFSDGSDLEGIEKVEDILYLRILKKIMWVIDGWPKEAYKSQFFRYYICILDMVSLIPGTLYLKINTGKIPSFELGHTYITVFM

NAIAALRTVLVLTRKYNEIIFYFLKEVHLFNFRRKSKYAYETHILVHKISHFFTMYVFVLMCCGILLFNLTPIYNSYAAGMFRDEPPPNATF

DHAVYFALPFDTSTSFKGYAVVSLYNWYICITCSTYFCIIDLTIFIMVFHLWGHMRVLSYNLENFPKPASVLAAADAHAYTVCENRYNEEE

LVEVFIRLRDCIQIHSLVINFSSMMADSFGWTLLVYLFFHQVSGCLLLLECSQLDTAALARYGPLTIIIFQQLIQLSIIFELLGSSNDRLIDSV

YSVPWEYMDTANRKNVFVMLRQSQRSMNLKACSMITVGVQTMIAILKTSFSYFVMLRTVADEEE

>OnubOR7a

MMFITDGSDLIGVTRVLEIKYMVIIRSAFRVVGAWPSKFIGDVQTTSDVVVKYIQLVLNVVCQVAGILYLRENMDKLSFFELGHSYITVL

MSLVSMSRIITHCTEAYQEIFSLYVRKIHLFNVRNDSEHAMEMHTKIHKLCYFLTFFIHAFMTLGILMFNLIPMYSNYINGKFNRETGAFS

GVSNATMEHAVYYLWPFNDTTHPIGYAIIVAFNWYISLVCSINFCTFDLFLYHLVFHIWGHLKILIHNLETFPRPIGAIRNEKQNDYTEEES

KQIYERLKKLVQHHNLIIDFIARISDTFGLSLFVYLCFHQVCGCILLLECSTLELSALIRYGPLTAIAFLLLIQVSLVFELLGSMTESLMNAV

YDLPWEYMEVRHRRTVHIMLRQSQVSLNTRALNMVDIGSRTMIAIIKTSLSYFVMLRTFATDD

>OnubOR7b

MMFITDGSDLIGVTRVLEIKYMVIIRSAFRVVGAWPSKFIGDVQTTSDVVVKYIQLVLNVVCLVAGILYLRENMDKLSFFELGHSYITML
MTVVAFSRIATHCTEAYQEIFSLYVRKIHLFNVRNDSEYAMEIHTKIHKWCYFLTFFIHAFMTLGILMFNLIPMYSNYINGKFNRKTGAFS
GVSNATMEHAVYYLLPFNDTTHPIGYAIIVAFNWYISLVCSINFCTFDLFLYHLVFHIWGHLKILIHNLETFPRPIGAIRNEEQNDYTEEESK
QIYERLKKLVQHHNLIIDFIARISDTFGLSLFVYLCYHQVCGCILLLECSTLELSALIRYGPLTAIMFQLLIQLSLVFELLGSMTESLMNAVY
DLPWEYMEVRHRRTVHIMLRQSQVSLNTRALNMVDIGSRTMIAILKTSLSYFVMLRTFATDD

>SlitOR1

MDIKLSSVKFFSDGSDLEGIEKVEDILYLRILKKIMWVIDGWPKEAYKSQFFRYYICILDMVSLIPGTLYLKINTGKIPSFELGHTYITVFM
NAIAALRTVLVLTRKYNEIIFYFLKEVHLFNFRRKSKYAYETHILVHKISHFFTLYVFVLMCCGILLFNLTPIYNSYAAGMFRDEPPPNATF
DHAVYFVLPFDTSTSFKGYAVVSLYNWYICITCSTYFCIIDLTIFIMVFHLWGHMRVLSYNLENFPKPASVLAAADAHAYTVCENKYNEE
ELVEVFIRLRDWIQIHSLVINFSSMMADSFGWTLLVYLFFHQVSGCLLLLECSQLDTAALARYGPLTIIIFQQLIQLSIIFELLGSSNDRLIDS
VYSVPWEYMDTANRKNVFVMLRQSQRSMNLKACSMITVGVQTMIAILKTSFSYFVMLRTVADEEE

>OfurOR7

MVIIRSAFRVVGAWPSKFIGDVQTTSDVVVKYIQLVLNVVCQVAGILYLRENMDKLSFFELGHSYITVLMSVVSMSRIITYCTEAYQEIFS
LYVRKIHLFNVRNDSEYAMEMHTKINKLCYFLTFFIHAFMTLGILMFNLIPMYSNYISGKFNRETGAFSGVSNATMEHAVYFLWPFNDTT
DPIGYAIIVVFNWYISLVCSINYCTFDLFVYHLVFHIWGHLKILIHNLETFPRPIGAINEEQNDYTEEESKQIYERLKKLVQHHNLIIDFIARI
SDTFGLSLFVYLCYHQVCGCILLLECSTLELSALIRYGPLTAITFQLLIQVSLVFELLGSITESLMNAVYELPWEYMEVRHRRTVHIMLRQS
QVSLNTRALNMVDIGSRTMIAIIKTSLSYFVMLRTFATDD

>HvirOR13

MKILSDGSDLEGVEKVEDIFYINLARKSMWILDSWPKAFNASSKYRYFVLALNVATLIGGAIYLRNNTGVLSSFELGHTYITVFMNCITC

SRCLMILSKDYNHVMSLFVQKIHLFHHKHKSDYAYLTHIFIHKISHFYTVYLLGLALNGLFLFNMIPFYNCYSRGMFRDVIPANATYDHA

VFYSVPFDYTTKFKGYLAMTSFNVFISYTCTSYFCVVDLTISLVIFHLWGHMRLLTYHLANFKKPASVLESNDNNKDEIKDHSYTEEELK

EVFSKLREYIQHHNLILEFSSEMSNAFGPALLAYMVFHQVSGCILLLECSQLDTKTLVRYGPLTIVIFQQLIQISVIFELLGSSNDKLIDGVY

LVPWEYMDTKNRKLVFTMLRQSHRSINLTMMSMVTVGVQTMTAILKTSFSYFVMLKTVAEEE

>HassOR13

MKILSDGSDLEGVEKVEDIFYINLARKSMWILDSWPKNPNESTTYRFVVLALNVTTLIGGAIYLRNNTGVLPSFELGHTYITVFMNCITC

SRCLMILSKKYNQVMSSFLNKIHLFHHRHKSEYAYKTHIFIHKISHFYTVYLLWLALNGLLLFNMIPFYNCYSRGMFRDVIPANATYDHA

VFYSVPFDYTTKFKGYLAMTSFNCFISYTCTSYFCVVDLTVSLVIFHLWGHMRLLTYHLANFKKPASILESNDNTDAIKDHSYTEEELKE

VFSKLREYIQHHNLILNFSSEMSSAFGPALLAYMVFHQVSGCILLLECSQLDMKTLVRYGPLTVVIFQQLIQISVIFELLGSSNDKLIDAVY

LVPWEYMDTKNRKLVYVMLRQSQRSIDLKMMSMLTVGVQTMTAILKTSFSYFVMLKTVAEEEQ

>HvirOR14

MTGIRDFFFNYEAKDGVTNPTEYPYMIMSRHLLTVITCWPKKPKEGLNARAKLRAKIWVIVQKIFHMSLCLLTTLGMAMYIGLHKKSM

SFLELGHLYISLLMTVVIFSRITTLCLNPKYRAVSTEFLTKIHLFYYKDDSEFSMQIHKQVHKISHLFTLYLTGQMIAGLSLFNLTPMYNNF

SAGKYKKGGLKNSTFEHSLYYSYPFNASSDVGGYIVSNICDWIISYLCSTWFCTLDLFLSIMVFHVWGHFKILLHDLDHFPRPANLTTFK

LDNSNITLTSEKFSSIELGQVSEKLKKCIEYHRKIVSFTDEMSEVFGPMLFVYYGFHQTSGCLLLLECSQMTVAALVCYLPLTIMLFQQLI

QLSIIFELVGSVSDKLKDAVYSLPWEAMDIKNKKTVAIFLMNVQEPVHVKALGLAEVGVTSMTAILKTSMSYFTFLRSK

>HassOR1

MKILSDGSDLEGVEKVEDIFYIRIARKSMWILDSWPRTPNESVIYRYFVLALNITTLVGGAVYLRNNTGVLSSFELGHTYITVFMNCITCS

RCLMILSRKYNEVMFSFVQKIHLFHHRHKSEYAYKTHIFIHKISHFYTVYLLGLALNGLLLFNMIPFYNCYSRGMFRDVIPANATYDHSV

FYSVPFDYTTKFKGYIAMTSFNCFISYTCTSYFCVVDLTISLVIFHLWGHMRLLTYHLANFKKPASVLESNENTHAIKDHSYTEEELKEVF

SKLREYICHHNLILNFSSEMSSAFGPALLAYMVFHQVSGCILLLECSQLDMKTLVRYGPLTVVIFQQLIQISVIFELLGSSNDKLIDAVYLV

PWEYMDTKNRKLVYVMLRQSQRSIDLKMMSMLTVGVQTMTAILKTSFSYFVMLKTVAEEE

>OfurOR19

MEGLEAYPEEFVNSLKLSLEYYKRVNITFFGSKTSFWDKYRHFFVFGIPFAFFYYTVTMYMVKVVAEGLDPFAKPDMIALWLISTQVIFK

YILFTKNKEGVRLVIEHLGAVWRMTDLTKEQILIKNSSLKFLKYGLYIYNKSCMTTAWQYFLYPFISMLFKHIFWGNEIEMVLPFPCEYPF

AVDNWPVYLAVYALQIIGALQMVHLYLAPNFLLTNLSIHISTQFRLLQDDLINIKPTNNKKTKYQYDMEITKYYEGKEYTIEDFVRRHQD

IILLTRQLNDAFNKMVFVNLVISTVVVCFFAVAVKTTIDPAYKLTNGAALVAYMANLLIVCYCSEMLSISSTGIALSAAKNMWYDGDLRY

QKIICIIIMRSQKPCTLLALNYYSISMKTFNKALKTTYSYFSLASHIYDGRKERKYTTEY

>CpunOR40

GFEIYEEEFVKPLFMSLDMLKRSNVMFCGPEVSFTKKYWRFFYMIPLLVLHYISLSAYILKLFTEGLDPFEKADMLPLWLATVEYWINTV

ILIINQDEIRNFIVHLGSIWRITGLNEKQMTIKTGILKRLYYVGIAVNKVGLAMSWQYLLAPLCETVIRRLFLKQEVELQLPFDCVYPFETK

DWPIYLAVYAFQVYCIFRTIYVYQGVGWLLVVLTTHMHLQFLLLQEDLVQVKPEIKGRPIKSLSEDADEITQYVDRPYFRIDDFVRRHQV

LISLADKLDSSCNKLTFTIMLFATLIICFFAVAAKASKGAAYALNNYGAVVVILMSILILCYCCQLLSSSSSGIAMAAAKNLWYKGDLRYQ
TNIRFIMMRSLKPCSLTLLGFSPIDLGTFNKVLKTTWSYFSLASQMYDERD

>CpinOR40

MYGFEIYEEEFIKPLFMSLDILKRSNVMFCGPEVSFTKKYWRFFYMVPLVVLHYISLSAYILKLFTEGVDPFEKADMLPLWLATVEYWIN
IVILMINQDKIRDLIVHMGSIWRITGLNEKQMKIKTGALKRLYYAGIAVNKVGLAMSWQYLLSPLCETVIRRLFLKQEAELQLPFDCVYP
FETKDWPIYLAVYAFQVYCIFHIIYVYQGVGWLLVVLTTHMHVQFLLLQEDLVQVKPEIKGRAIKTLSEDADEITQYVDGADVRIDDFV
RRHQDLISLAEKLDSSCNKFTFTIMLFATLVICFFAVAAKASKGAAYALNNYGVVVVKLMSMLIQCYCCQLLSSSSSGIAMAAAKNLWY
KGDLRYQTNIRFIMMRSLKPCSLTLLGLTPIDLGTFNKVLKTTWSYFSLASQMYDARD

>CpunOR19

MDGFEIYHKEFLKPLIVTLKMLNQFNVRFCSSEVPFLTRNWRFFYLVPLWILHIISLMSFIVNLIMEGNDPFEEAYMIPICIISFEHCIKMCIL
MKKREEIRDVITNIGNIWRSTGLTDDQKKTKEEILKKLYNAGLVFNNIGIAVTIQYLMVPLFETLVRRLILKQEVELMLPLTIIYPFEIKTWP
LYLVIYGFQVYSLLCAACVYLGFGWLLVVLTCYLNIQFLLLQEDLIHVKPETSNRLSYLPEEEGEITQYWEPSPFGIDDFVRQHQTVILLT
DKLNAAVNKITFTIILFATLNISFFAIAVKASAGAVDMVNNFGAIIVIMMNIFILCYCSQLLSSSSSGIALAAGKNLWYKGDLSYQTKIRFIM
MRSQKPCSLTSLDFSPIGLETFNKVLKTTWSYFSLASQMYDERY

>BmorOR4

MEELRGLPEDYLEPLLPCLDLIKRCNVTFFDNGTLFNRYWRYFYIIPCVVAFYIFLSAYMMQVFEGQLELSELAYVVSVYVVSNQAIVKA
IIVVLKSKEIRSIILELGQAWRTEDLSDAQINKKKILLKRLNFVSKAFYWMNIIGSWQYMLTPLCEILIRKYVLKQEPEHVLPFSCKFPFDP

TANWPTYLGVYSFQTYSMFLIVYCYVGVELLMVNLCAHISTEFALLHEDLRNLTPLRNSKGKGLGNGIRKIVRHHVKLIRLSQQLDNIF
NIMIFINLSSVTFTICFFGFAAKVARTAIEKAKNFIGVIALIVQIFNLCYYAELLQDASSTIADAAYENLWYNGGIDHQKNLLFIIKRSQNPCS
LTSMKYSPIALNTFTAILSTAWSYFSLISSLYEGETT

>MsepOR85

MEALPDIPGEFIEPILKCLNLLENCHLYLYAVKQGFWKKYVHPVQNVVCLVAYQLALTMYLGNLFRGNLKVDEIAYVASVYVVSMQAIL
KAVVVMLRKSEIKAIILQLGSMWRTKDLTEVQINKKNALLKRLKFCYAVYYWINMIGSWQYILAPLLETVFRKFILQQECGLLLPFGCSF
PFDPTGNWARYIGVYIFETYSMFRIVYFYLGTEFLMIILCSHLATAFTLLQEDLLNLKPGNLATLKIIIDSHHKLISLTQRLDDVFDKIIFINL
TSASISICFFGLSAKVAHGALQMANNFAAVLCLILPLFNLCYYGEMLREASAGMADSVYNNPWYQGDLRYQKLLLFIIKRSQKPCYLTS
LKYNPITLNTFTTVLSTTWSYFSLASSVYEGES

>SlitOR40

MEELPEISKEFIEPILPCLGLLRNVHILIYAHGKPFWNEKCHLTLNIVSVVTYLFSLTMYMGKVFRGELQLEELAYVISVYVVSVQAILKG
LVVVLYKKEIRAIILQLGGMWRVEHLTEVQINKKNTLLRRLKFCYAVFYWINMIGSWQYILAPLLETVTRKFILLQECGLLLPFSCSFPFD
PTGNWARYIGVYIFETYSMFRIIYVYLGTEFLMITLCSHLTTAFTLLQEDLLNNEPGNFRNLKTIIVHHQKLISISKQLDDIFNKVIFVNLSS
ASISMCFFGFCAKVAHRALDMANNFIAVLTLTLPLFNLCDYAERLREAVPETMLFNFFEIQ

>SlitOR4

MEELPEISKEFIEPILPCLGLLRNVHILIYAHGKPFWNEKCHLTLNIVSVVTYLFSLTMYMGKVFRGELQLEELAYVISVYVVSVQAILKG
SVVVLYKKEIRAIILQLGSMWRVEHLTEVQINKKNTLLRRLKFCYAVFYWINMIGSWQYILAPLLETVTRKFILLQECGLLLPFSCSFPFD

PTGNWARYIGVYIFETYSMFRIIYVYLGTEFLMITLCSHLTTAFTLLQEDLLNNEPGNFRNLKTIIVHHQKLISISKQLDDIFNKVIFVNLSS

ASISMCFFGFCAKVAHRALDMANNFIAVLTLTLPLFNLCDYAERLREASAGMADSVYNNLWYLSDKHYQKLLWFILRRSQKPCCLTSLK

YSEIGLNTFSAVLSTTWSYFSLASSLYESET

>SlitOR67c

MEVLKDFPEDFAKALKTSFEMLKNFNVRYLNDDQPFVKKYWRFSYIFIIIFIHGFSMSIHMPELLTGDEMTQFAYLIPSILVTIHAIFKSIVL

IPMTRQISTFISELGSLWRVKFTEKQFQDKDAVLWRLDFINRASYWVTLSGSAQYLLSPLFETLFRRFILKQDCKLLLPFASDFPLDHTNN

WMFYILVYIFQLYSMFLLVSMYTGAALIMISSCALLGAEFLMLKDDLSRVKPRNNNEIRDSNNTNEGVNNDNELTIEEFVKRHQKLLGL

SRQLDNVFNGMVFIDLLFVGITTCAFSFMGQFARGPGYMLVSYIGIASNMFTVLYLCYYGELLTRASSSIGDTAYENLWYKACKRHKMA

IFLIIKISQKPCCLTSIRYAEVSMKMFTKVVSTTWSYFSLMNTLYSDSN

>OfurOR32

MKIIPKGIIEKCTRTVGDRSEIDEVMRAILITQRAVGNQILDPYWSWMKSLPHQLVLGAFMFYVLIGTWEFVGGTDDVKLIAEGSFTYIV

AALIPSRYYFFLMARKDFQKLYIAFKTTVCKFITDDSEEKMEQLLKKTRSLVKFMLFSSNFPMAIYFLAAMWHYVQGEKRTISKTTSILM

PMRSPYHEIGLFAHSFFIFEAGFLILVPDMWFVVIMLFFCSACDSAAKFLIVEERRNESKLQYATRLNDSLRKFYVAHVKLIDFLDVLNSV

FKWLALLPLISVGVGICIILLLITQGVDWAFLSNILPVMGELFVYNWFGEQIINKAEKWSLALLNFDWINLSAKDKKCYYIMVSYMQKK

FRMKTAIGSEFSLLTMSTCVKGGYQAFTVLQSATHKRE

>HvitOR35

MKIISRSTIDKFTYTLGERNEVDQMMSFTLLIQRLLGQQMLDPCWTCLRFWLHHVFGLALIIYVLFGTIHFLKNTTNDKLIAEACYTFVV

TFSFPLKYILFVFNKRVFRELYLEVKTTLYEVIKKNSVDGGKSVLEKVKKRVYLLYGLTMCPVTSYVATAIWCYLNGKRITVSKTTSILMP

MKTPYHEIGLILHSIFMFSISSVVIVIDMWFVLLMYFFCLSIDGTKNMLNISARTSGESQLEYALRLNAGLRNFYNTHVKQVEYLHKMNI

MFKWLGLLPLIYLAINGCIIFLLLRKGVDWTFASNMLPVFAELFAYNWFGEQIKVKMQELNLALLNFDWTNMELKDKKCYFIIVSYMN

KAFSLKTAIGTDLSLITMTEVLKVSYQTCTVLQTMD

>CpunOR32

MKFSIENYTHTVGNKNEMNGIMRIVLVLQRFFGQHILNPDWTWKRFCLYQLATFALFSYVLAGTFAILQETDDIKMIAEATYTVVVTGV

SFVKYCIITSKRFVFRRLYVELKSSLYDIVRDDSEEKMKKVFDDGKKSVYYIFVFTLCPIIMYIARVLWYNLHGQKVTLSLTTSILMPMET

PYYQLGLILHSIYFIEVSFIVIQVDMWLVLFVFFFCVTSDITLKILTVEKRKQGEDRIEYAVRLNDSLKRFYRLHVKQVNFLSMLNGTFKW

ITLVTQVNICICICIVLLLVRKGAESAFALNILPSIAELIGFSWLGEQIKTKTNNWKMALLDFDWINLQQKDKKNYYILMCYMNQEFGLQ

SAIGGDLVLITVSKVLKFSYQVYTVLQSM

>CpinOR32

MKFSIENYTHTVGNKNEIDGLMRIVLVLQRFFGQHILNPDWTWKRFCLYQLATFALFLYVLAGTFAILKETDDIKMIAEATYTLVVTGVS

FIKYCIITSKRFVFRKLYVELKSSLYDIVRDDSEEKMKKVFDDGKKSVYYIFFFALCTIIMYTARVMWCNLQGQKVTLSLTTSILMPMETP

YYQLGLILHSIYFIEVCFIIIQVDMWLVLFVFFFCVTSDITLKILTVEKRRQGENRIEYAVRLNDSLKRFYRLHVKQVNFLSMLNDTFKWIT

VVIQVNICICICTVLLLVQKGAESAFAVNILHSIAELIGFSWLGEQIKTKTNNWKMALLDFDWTNLQQKDKRSYYILMCYMNQEFGLQS

AIGGDLVLITVSKVLKFSYQVYTVLQSM

>MsepOR47

MKIFSSQEYFKTIGEWNDFDNVMRLPLFVQEILGQNVLDPTWNLRTRMAKQIFFIILITYVMIGTKEFLKDATDITEIGEAYYTFQITFFFS
VKYLLFINTRETFKKSYMMAKTSVLDIIRADSTEKLTEMLGKVKIVVRVLFAGVLCPVVMYLVVAFWHYVTGTRVTLSKTTSILMPMTT
PYYEIGLVLHTIYLVEMAFTYCVIDLWFAVLMFTFCVASDSVVNKLKVESRGPDETELEYMDRLNNTLRSFYKNHAILMEYFNITSHMF
KWPALIPLVSVLFAVCLILLCMTEQIQWMFLSNLVPIMSEIFAYNILGEQINSKGIQFYTALLEFDWASMRLKDKKNYLIIISYLNKDFKMK
TALGTELSLVTLTSVLKSGYQVFAMINTMET

>LstiOR1

MFMNSKFQQLLGTMWENLRKFGLGHCDLPTMVWNVAFMLRGFTLNIDSRF
TGRIPKIFYITTIIIAFCYLYSYFFSMLWFVFVRCIETGDVTAAMIVFPLGITSEIGIAKFIYTCVYRKKVRQLLQQYLEYDSQIPQGSRLSRH
LLQALRNVKRRALIYWIFIVSNGTLYILQPLVMPGRVPMEEVFVLYGLEPELETPNYEITYVLCTFGSVCTCYLTSNVAAFLIIVSGYVESQ
LLALSEEILNVWDDAELEYKVIDNADEEEFENKEKYDAINESVKIRLKDIVKGHTTNINLLLQVEDIYRGAFAFEFCILSVGLIAELLGGL
ENTYMEVPFAIIQVGMDCLIGQRVMDACDTFESAVYSCKWERFNEANMKTVFMMLMNSQKTLTLTAGGIAVLNFVCLMSVFRSIYSAF
TTLQSVM

>OfurOR50

VFVTLTTALHFVFYTLNLIYTPRKIEIFATQAVYYFSSVSGLFKIGTVLTKQQEILSTFEIIDCKEFLGNCSETKKYLQQFRRTYFRYFRLYLT
FCMCCAVVFLCGLPLVNYFFRHEDLKLPVCEYYFLTDEVRKNYIFYWFDYQILGLIVTIVYNSTTHTFLCGLILMGITQFKILNFNIANIRL
DEDIEINNQEEREQALINKLNQCLKHYDIILKYCENVQNIADVFFFVQFSLAAITICFCMYMLILDLSDKDKVFTTCFISAMLLENYTPSFL
GSHLTAESDNLRIAAYSCNWTPRSHSFKKSLILLMERAHRPVVIVALKMVPINLETFASMVKTAYSFFTLLSGAQE

>OfurOR10

MFRLKEKDVIASNTQSQVFKPNIFFWKIFGWWPEVTSTIYYRCYYISFLSLTSVVYLFLFTLSLLYSPIELEIIIAQAMFYFTEISGLSKIFMI

VIRREDIQKAFKMLDSEEFQGDDVIPREIINKNKVYYLKYYRACATFYYIGSFFLLFLPIIEYVAGHADLELPLCQYYFLSEHVRDKYFNV

IFIYQFFGLFVLISGNVNIDTFICGLLLMAIAQFRMLNWKMSNLKMNPLDLENESDDEETIMMRKLNKCLKHYDLILEYCDHIQDVLSA

AIFAQYGTAAATMCLSMCTVLMPMTSEDWLFMGCYIGAMTLEIFLPGLLGAELMNESQKLVAAAYSADWIPRSESFKRSLRLLVERAN

RPIVITGLKMFTLSLETFTSIIKLAYSFFTLLKNVQETEIA

>OnubOR2a

MFLLREKDVISSNTQSQVFKPNIFFWKIFGWWPEVTSTIYYRCYYISFLSLTSVVYLFLFTLSLLYSPIELEIIIAQAMFYFTEISGLSKIFMIV

IRREDIQKAFKMLDSEEFQGDDVIPREIINKNKVYYLKYYRACATFYYIGSFFLLFLPIIEYVAGHADLELPLCQYYFLSEHVRDKYFNVIF

IYQFFGLFVLISGNVNIDTFICGLLLMAIAQFRMLNWKMSNLKMNPLDLENESDDEETIMMRKLNKCLKHYDLILEYCDHIQDVLSAAI

FAQYGTAAATMCLSMCTVLMPMASEDWLFMGCYIGAMTLEIFLPGLLGAELMNESQKLVAAAYSADWIPRSESFKRSLRLLVERANRP

IVITGLKMFTLSLETFTSIIKLAYSFFTLLKNVQETEIA

>CpunOR10

MEPTQSQVFDLNIFLWKIFGMWHHETSTIYYKYYCVTFQMLYSVTYTFLFTLSLMFTPNDLELIISQCMFYFTQLAGVTKIAMIILRKKYI

FEAFHILDCIEFQGDDVETRKIVNENKIFYKKYWKACFVCYNSGGFFILCLPLINYVVRRTELDLPLCQYYFLSDHTREKYHSGLFFYQF

TGLVVIILSNISTDTFIIGLLMMAITQFRVLNWKLTNLNFSSLDEELVTMVDKEVLFVEKLKKCLVHYDLLLKYCNIIQDVTSYSIFAQFGT

AAVTLCVSMCTFLRPMTNNDFMTSVTYMTSMAIEVFLPAYYGAELTHESEKIVMAAYSSDWIPRWKSFKRSLLLVMERAKRPVVITGL

KMFTLSLETFAAIIKMAYSIFTLLKNAQNQNEN

>GmelOR45b

MSMKLSDNFKINTFFWKIFAIWPGNQRNDYYKYYSFVYILINLVIYNILLTLNLIFTPRKIEIFIREVIFYFTEVAVTVKVLMIIIYRKKIIAIF

YLMNCDEFRGEDSDTVNIVKNDNLIYKVGWIIYAVICNFAYTIITFLPVLMNFIWNTKLELPICRYYFLNDDIRNDYFVYWFIYQSFGIYG

HMMYNVNVDTFIAGLILIAISQIKVLQFKLANLKMNATLKSQEEQEHLQILKLKKYLQHYDILLRYCSSVQEILDVVIFVQFGMASVIIC

VAMYGLLLTTTKERFVFMVFYFSTMLSQIFIPSFLGTRLFYESENLVFAAYNSEWIVRSEKFKLNLKLFMERAKSPIVLKGLKLFTLSLGA

FTSIMKTAYSFLTLVKNVVDQQDEKI

>GmelOR27

IFAIWPGNQRNDYYKYYSFVYILINLVIYNILLTLNLIFTPRKIEIFIREVIFYFTEVAVTVKVLMIIIYRKKIIAIFYLMNCDEFRGEDSDTVNI

VKNDNLIYKVGWIIYAVICNFAYTIITFLPVLMNFIWNTKLELPICRYYFLNDDIRNDYFVYWFIYQSFGIYGHMMYNVNVDTFIAGLILI

AISQIKVLQFKLANLKMNATLKSQEEQEHLQILKLKKYLQHYDILLRYCSSVQEILDVVIFVQFGMASVIICVAMYGLLLTTTKERFVFM

VFYFSTMLSQIFIPSFLGTRLFYESENLMFAAYNSEWIVRSEKFKLNLKLFMERAKSPIVLKGLKLFTLSLGSFTSIMKTAYSFLTLVKNVH

DQQDEKI

>MsexOR59a

MSSFCQTDIFKPNFFFWKCFGIWGGRTENKNYKYYSFSYLFVTLFVFNILLTINLIYTPLKIESLIREVIFYFTEIAITVKVLMILVMRSKILD

VFNLLDCKEFQGDDEESKQIIETNHSFYRTCWKLNAVLSNISFASNVFAPLFINLIWTAKIEFPVCKYYFLSDEMRDKYFIFWFIYQSIGIY

GHMMYNVNVDSFIAGLLLMAITQLKVLNAKFTKFKLEKRHEKHHILIQNKIQILRLNRYLKHYDCVLRYCEIIQDLLSVTMFVQFGMAS

AIICVIMCGLLLPSTTETFVFMVTYLFAMTIQIFVPAWLGTQLSHESCGLVFAAYNCEWIPRSMSFKRSIMIFVERANNPIQLTGLKMFPLS

LATFTSIMKTAYSFFTLFRNLQDHDDGAN

>MsexOR23

MSSFCQTDIFKPNFFFWKCFGIWGGRTENKNYKYYSFSYLFVTLFVFNILLTINLIYTPLKIESLIREVIFYFTEIAITVKVLMILVMRSKILD

VLNLLDCKEFQGDDEESKQIIETNHSFYRTCWELNAVLSNISFASNVFAPLFINLIWTAKIEFPVCKYYFLSDEMRDKYFIFWFIYQSIGIY

GHMMYNVNVDSFIAGLLLMAITQLKVLNAKFTNFKLEKRHEKHHILIQNKIQMLRLNRYLKHYDCVLRYCETIQDLLSVTMFVQFGM

ASAIICVIMCGLLLPSTTETFVFMLTYFFAMTMQIFVPAWLGTQLSHESCGLVFAAYNCEWIPRSMSFKRSIMIFVERANNPIQLTGLKMFP

LSLATFTSIMKTAYSFFTLFRNLQDHDDGAN

>CpinOR56

MESGQSRVFKYNIFFWKCLGLWSDETSGYYYKYYSVAFQTFYSGIFTFLYTLNFMFTPFDLEIVISQSMFYFTQLAQIAKIAMIVLKRKYI

LRAFNMLDCIEFQGVDIKTTEIVNKNKYSYTNYWKACCVYYHISCFSIIILPIIKYFVIGTVIELPLCQYYFLGDDIREKYYKALLFYQFTG

IIMIMYSNLNTDTFINGFLMMAITQFKVLFWKLTNLDPSSLDQGVISTKDKEVLLSHKLKQCLIHYDLILQYCSIIQDMMSFAIFGQFGTA

AVTICVSMCFFLKPMTNEDLLIITFYLLAMIFQIFLPTYLGAQLTEQSELIVTAAYNTDWIPRSKSFKRSLGLLMERAKTPIVITGLKMFTLS

LENFSSIMKMAYSLFTLLKNSQAET

>GmelOR46a

MSTKQSDIFNVNTFFWKVFALWPGNIPYKYYKYFSFVYLIINFIIYNILLSLNLIYTPKKVELLIPEMLFYFTEVTVAAKILMIFVYRNKIIAI

FELINCNEFKGDNSDTKGIIKKDNLIYKRGWITLTLLGNFGFLSLVILPLIASLIWNRKLELPICKYYFLADEFRDKYFKYLHIYQSTAKYG

HMMYNVNIDSFLAGLMLIAISQMKVLNYKLTNLKGNTSSREDRKELENAQISKLNACLRHYDIILRYCKSIQDISDVVLFVQFGMGSVII
CVTLYGLLLPSIKASRMFMISYVMGILSEILIPSYLGTQVSYESEKLVVAAYDSEWISRSEKFKKSLKIFMERAKTPIVLTGLKLFPLSLTTFI
WVIRTAYSFLTLVRNVQDRQEGKV
>GmelOR28
AIWPGKLSNKFYKYFSFMYISINLVIYNILLTINLIYTPKKIEMLIPEMIFFFTEVSVTVKVLMTIVNRKKIIVILDLMNCDEFKGEDFNTSYI
IEKDISIYKMGWKINATLSNFAYASLIFLPIIMSFIWNTKLELPTCNYYFLSDDIRDDYFIYWFIYQSFGMYGHMMYNVNIDTFIAGLIFIAV
SQMKVLNFKLLNLKVNANSGLDEKEQDRLQTLKLTKCLEHYNILLRYCMSIQEILDVIIFVQFAMASIIICVTIYGLLLLSTNKSLMFMVS
YLLAMLCQIFIPSFLGTLLSFESENLVFAGYNSEWIPRSEKFKKILKIFMERAKTPIVLTGLKLFPLSLGTFTSVMKTASSFLTLVRNVQDRQ
DGKI
>CpunOR56
MESTAQSRVFKYNIFFWKCLGLWSDETSGYCYKYYSVAFQSFYSGMFTFLYTLNLMFTPFDLEIIISQSMFYFTQWAQITKIAMIVLRRK
YILRAFNMLDCTAFQGEDEETREIVNKNKCSYTNYWKACCVYYHIISLSIIILPIIKYFVNGTVIELPLCQYYFLGDNIREKYYKVLFAYQF
TGIIMIMYSNLNTDTFINGFLMMAITQFKVLYWKLAHLDSSSLDQGVINEKDKEILMIDKLKQCLIHYDLILQYCNIIQDVMSFAIFGQFG
TAAVTICVSMCFFLKPMTNEDLFITTFYLLAMIFQIFLPTYLGAELTEQSELIVTAAYNSDWIPRSESFKRSLGLLMERAKTPIVITGLKMFT
LSLENFTSIMKMAYSLFTLLKNSQVEA
>HvitOR16
AIYWFLFTLSLLFTPIDLEIIIAQAMFYFTEIAGLSKIGMIIFKQNEILNLFELLDSEEFQGDEEMPMKIVNDNKVYYLKYWKACAIFYYTG

SFFLLFLPVFEHFFGNVDLIFPLCQYYFLSDNIRDSYFTIIFLYQFFGLFVLITCNVNIDTFICGLLLMAITQYKVLNWKLSNLCLKPTELSL
NREEQDKILMNRLNKYLKHYDQVLRYCRDMQKIMGFSIFAQFGTGAATICVSMCTFLRPMTNEDWMFMGCYIGAMTLEIFLPGLLGA
QLTQESADLAYAVYCSDWISQPENFKRNLRLMVESANKPIVVTGLQMFSLSLETFASIIKMAYSFFTLLKNVQAD

>OfurOR82a

MEFIKRHYREFRSRMQDYSYDSLLSIVNFVPSLVGFSILGNKISAPFWILHLSLLFYIYGVGCTVYQVKYAGDARDFIKCYVNVSLLILIA
NNSHWFLLKRPLLKSILLEISQSDALATANEAFRGKHKRAVQRIKRILYMFYGFNLTNAMFVYLPNRMDVKNSYSMTPCYGMEPLTASP
NKEICSALLLIQEISIMTVVLNYQALLVVLIAYTALLYRLLSEEIMTLNNYDRQTYFNNPIAKTMLHELIKRHVILLSIIDKLKSLYSGSIGIN
FGSNAVCMSLFFYLPLQEWLQFMPVVVYCFLVFFLYCFLCQRLTNAAEYFEQCVYSCGWENFDVKEKKAIYFMLRQAQRPVEILAADII
PVNISTFATTLQAMFKFVTVVKV

>OnubOR82a

MEFIKRNYREFISRMQDYSYDSLLSIVNFVPSLVGFSILGNKISAPFWILHLSLLFYIYGVGCTVYQVKYAGDARDFIKCYVNVSLLILIAN
NSHWFLLKRPLLKSILQEISQSDALATANEAFRGKHKRAVQRIKRILFMFYGFNLTNAMFVYLPNRMDVKNSYSMTPCYGMEPLTASPN
KEICSALLLIQEISIMMVVLNYQALLVVLIAYTALLYRLLSEEIMTLNNYDRQTYFNNPIAKTMLHELIQRHVILLSIIDKLKSLYSGSIGINF
GSNAVCMSLFFYLPLQEWLQFMPVVVYCFLVFFLYCFLCQRLTNAAEYFEQCVYSCGWENFDVKEKKAIYFMLRQAQRPVEILAADIIP
VNISTFATTLQAMFKFVTVVKV

>OfurOR11

MEFIKRNYREFRSRMQDYSYDSLLSIVNFVPSLVGFSILGNKISAPFWILHLSLLFYIYGVGCTVYQVKYAGDARDFIKCYVNVTLLLLIA

NNSHWFLLKRPLLKSILQEISQSDALATANEAFRGKHKRAVQRIKRILFMFYGFNLTNAMFVYLPNRMDVKNSYSMTPCYGMEPLTASP
NKEICSALLLIQEISIMMVVLNYQALLVVLIAYTALLYRLLSEEIMTLNNYDRQTYFNNPIAKTMLHELIKRHVILLSIIDQLKSLYSGSIGI
NFGSNAVCMSLFFYLPFQEWLQFMPVVVYCFLVFFLYCFLCQRLTNAAEYFEQCVYSCGWENFDVKEKKAIYFMLRQAQRPVEILAAD
IIPVNISTFATTLQAMFKFVTVVKV
>CpinOR11
MDLFEHVKNIFSNVKTRLKHNTYADLLWLVNIVPSFAGFSILGDTLWAPFWIVHLSLLMYIYGVGCAVYQIRDAQNTGDFIKSFVNVSLF
VLIVNNSYWFMMKRPLLKSTLAAISKSDEMATVNVIFREKHKRYLMKIKFILLTFYGFNVGNAAFVYLPHRADVLNDYAMTPCYGMEP
LTSSPNREICMTLLCLQELSIMTVVLNYQALLLLLIAHTALLYHLLSDEIMTLNNFNKDELYNNPAAKELLPVIIHRHSIILSVIFKLKALYS
VPIGVNFGSNAVCISLFFYLPLQEWLQFMPILVYCFLVFFLYCFLCQRLSNAAEVFEMAVYACGWENFDINERKAVYVMLRQAQKPVEL
LAADIIPVNISTFATTLQAMFKFVTVVKF
>CmedOR32
MEVFERVKQSILNTKFRLQQNTYASLLWLVNIVPSIAGFSIQRDTISAPFWIAHLSLLVYIYGVGNVVYQIEYAQNAGDFIKSYVNISLFVL
IANNSYWFIKKRPLLKSTLDQISESDQLSIVNETFREQHARSVSKIKRILFIFYGFNYGNAAFVYLPHRVDVRNNYAMTPCYGMEPLTSSP
NREICLALLCAQEFSIMTVVLNYQALLLLLIAHTALLYDLLSAEIMTLNNFDRKLHYNNPAAKDLLPVIIKRHALILSVIDKLKALYSVPIG
VNFGSNAVCISLFFYLPLQEWLQFMPILVYCFLVFFLYCFLCQRLSNAAEEFEIAVYACGWENFDRNNRKAVYVMLRQAQRPVELLAAD
IIPVNISTFATTLQAMFKFVTVVKF
>HvitOR6

MDLIDKLKNVFREIKMRLQDNSYDSLLWIVNIVPNLAGFSIRGDTISALFWIVHLTMLTYLYGVGSLVYQIRFARTPGDYIKSYVNVSLIV
LIANNSYWFIKKRPLLKLTLGELCESDELSRSNPIFRTIHERAIFRIKIIILTFYGFNLTNAIGVYLPHRVDVLNNYAMTPCVGMEPLSSSPNR
EICLTLLFLQEISIMTVVLNYQALLLLVIAHTALLYRLLSYEIMKLTEFDAVKYFNNPNVNILLPVLIRRHAIILRIIENLKALYSVPIGVNFG
SNAVCMSLFFYLPLQEWLQFMPVLAYCFLVFFLYCFLCQRLTNAAEFFERAVYACGWESFTTKERKAVYFMLRQAQKPVEILAADIIPV
NIATFATTLQAIFKLFTVFKF

>BmorOR54

MGLNTIKEFFVNVKRRFQDVSIDSLLWIVNIVPSLAGFSIRSDRVSAPFWIVHWSLLVYVYAVGNAVYQWKFANEAIDYITSFINVSLLILI
GNNSWWFLANRRLLKSVLHKIEVNDELSRRSEQSRLKHKKLLKIIKRIVLVFYMSNYVNASFIYLPNRVDVLNNYAMTPCVGMEPLTVS
PNRELCLTILCMQEFSIMTVVLNFQALLLCFIAHTAVMFQILADEIMALNNYENLEEHQAYVKEMLPIFVKRHSLTLSAVDNYKSLYSVP
LGVNFGSNALTILLILYLPVLEWFKFIPIFVFCFMLFFLYCFLCQKLVNASEAFETAIYCCGWENFALREMKMIYVMLHQAQKPVELLAA
DIVPVNMNTFATTLQAMYKFVTVVKF

>SlitOR46

MFLLFYVYAVGNFWYQWKFAHGAGDFIKSYVNISIIVIIGNNSVWFVKQRPLLRTVLKKIEQSDELSRRSSFLMRKHEKLMKIVKRIVLI
FYGFNYIDAFFIYFPHRVDLRNNYSMTPCVGLQPLTASPNREICMTILTLQEFTINIVALNYQALLLFLIAHTAAMYQMMAYEMMALNDY
KKENLGQVNKKLSSLIERHCLTLDVVDNLRSLYSVPLGVNFGSNAVCISLFFYLPLRECLQFMPVFVYCSLVFFLYCFLCQRLINSAEVFA
RAVYCCGWENFGLKEKRLVFVMLRQSQKPVELLAADIIPVNIYTFATTLQAMFKFVTVVKF

>OnubOR49b

MDPTKQSYAFNTTVTLWIIFGLWSNKSSNKFYKWYCFVFVALTTALHFVFYTLNLIYTPRKIEIFATQAVYYFSSVSGLFKIGTVLTKQQEI
LSTFEIIDCKEFLGNCSETKKYLQEFRSTYYRYFRLYLTFCMCCAGVFLCGLPLVNYFFRHEDLKLPVCEYYFLTDEVRKNYIFYWFDYQ
ILGLIVTIVYNATTHTFLCGLILMGITQFKILNFNIANIRLDEDIEINNQEEREQALINKLNQCLKHYDIILKYCENVQNIADVFFFVQFSLA
AITICFCMYMLILDLSDKDKVFTTCFISAMLLENYTPSFLGSHLTAESDNLRIAAYSCNWTPCSHSFKKSLILLMERAQRPVVIVALKMVP
INLETFASMVKTAYSFFTLLSGAQE

>CpinOR10

MESGQSRVFKYNIFFWKCLGLWSDETSGYYYKYYSVAFQTFYSGIFTFLYTLNFMFTPFDLEIVISQSMFYFTQLAQIAKIAMIVLKRKYI
LRAFNMLDCIEFQGVDIKTTEIVNKNKYSYTNYWKACCVYYHISCFSIIILPIIKYFVRRTELELSLCQYYFLNDQTRVKYHGGLFFYQFT
GLVVIILSNISTDTLINVLLMMAITQFRVLNCKLTNLRFSSLDEELVTTVDKEVLYVEKLKICLVHYDLLLKYCNIIQDVTSYAIFAQFGTA
AVTLCISMCTFLKPLTNNDFMTSLTYEYMTSMLLEVYLPAYYGAELTNGSEKIVMAAYSSDWIPRWESFKRSLLWSWSVRKGLL

>BmorOR12

MTRITDVFSLNFIFWKFLGLWGKSAPSKYNMAYTVFYLFASLFVYDIFLTLNLIHTPRKLETLVRETMFYFNHLVAVTKILMMFIMRKKIL
VIFDLLDCEEFKPNDENSQEIMKRKTDFYYIYWRIVAVTSNLSCFMLVIGPLIKMLIWKIELGLPVCKFYFMSDELRNKYFVIWYIYQSFG
IYNQMVNNLNLDTFNCGMLWMAVGQLQILKTKFVNLKLNDFENGLDLKSRDDMQIERLRKYLTHYEIILKYCATVQDILNITIFVQLG
MSSIVICVGLCGFVAMPSNTETAIFMSSYLITMTMQIFVPSWMGTQISFECGELMSAAYCCEWIPRSKLFKRSLILFVERAKTPVRITGLKI
FTLSLDTFTSIMKTTYSFFTLIRQLQVDEVN

>BmorOR61

MARITDVFRLNFIFWKFLGIWGKSAPSKYNMAYTALYLSASLFVYDIFLTLNLIHTPRKLETLLRETMFYFNHLVAMTKILKMFIRRKKIL

VIFDLLDCEEFKPSDEDSQEIMKRKNEFYYIYWRIVAVTSNLSCFMQVVGPLIKMLIWKSELGLPVCKYYFMSDEFRNKYFVIWYIYQSF

GIYNQMVNNLNLDTFNCGMLWMAVGQLQILKTKFVNFKLNDIENSLDLKTRDDMQTERLRKYLTHYEIILKYCATVQDILNITIFVQLG

MSSIVICVGLCGFVAMPSNTETAIFMSSYLITMTMQIFVPSWMGTQISFECGELMSAAYCCEWIPRSKLFKRSLILFVERAKTPVRITGLKI

FTLSLDTFTSIMKTTYSFFTLIRQLQVDEVN

>HarmOR7a

MPSDLSKIFEQTLRFLNFFGVWAGKTPSKYYKYFSFISVFLAVVCYNLLLTINLVYTPRNIELILREVIFFFTEITVTAKVFTVILMRDEIIEV

LNFIDNDKFIGDNGKKDGILYKTHKGYKFSWRVYNILAHAAYIFDIIAPLFLAFLRGTKSELPVCKYYFLDDEFRDSYFFILFLYQAIGMY

GHMIYNVNVDTLSAGFLAIAVAQLKLLNSNLTTLKLSAEECKLPREIQEKIQITRLNKLLRHYEFILNYCGAVQNVLSISLFIQFGVGSLIIC

VVMCSLLMPASMESRLFMVIYLLMMTGQIFVPGYLGTLLTYESQELVTAAYNCEWLVRSKSFRKDLILFRERAARPIRISGMKMFPLSLV

TFIAIMKTAYSFFTLIRNVQDK

>HzeaOR7a

MPSDLSKILEQTLRFLNFFGVWAGKTPPKYYKYFSFISVFLAVVCYNLLLTINLVYTPRNIELILREVIFFFTEITVTAKVFTVILMRDEIIEV

LNLIDNDKFIGDNGKKDGILYKTHKGYKFSWRVYNILAHAAYIFDIIAPLFLAFLRGTKSELPVCKYYFLDDEFRDSYFFILFLYQAIGMY

GHMIYNVNVDTLSAGFLAIAVAQLKLLNSNLTNLKLSAEECKLPREIQDKIQLTRLNKLLRHYEFILNYCGAVQNVLSISLFIQFGVGSLII

CVVMCSLLMPASMESRLFMVIYLLMMTGQIFVPGYLGTLLTYESQELVTAAYNCEWLVRSKSFRKDLILFRERAARPIQISGMKMFPLS

LVTFIAIMKTAYSFFTLIRNVQDK

>HzeaOR59a

MPSDQSRMFDPPLTVLKIFGVWEGRTPSKYYKTFSFLFLFVSWFFYNFLLTLNLVYTPRSVELFLREVIFYFTEITVTSKFLTVLLLRDKIL

EAFSVIDSDEFVGDYENKDGILYRTNKGYSFCWKVYNVLALIAYTCDIIMPVVIDLIRGTKSVLPICNYYFLSEDFRDSHFVILYLYQSIG

MYGHMMYNLNMDSLAWGLLAVGIAQIKVLNKNFTDLKLSAEESKLPLEIQDNVQKTRLFKLLRHYEAILNFGALTTCVIMCSLLMPGT

MVYRIFLVIYLLAMAGQIAVPGFFGTLLTHESEELVTAAYNCEWIERSQSFKRTLILFRERAGTPIIISGMKMFPLSLVTFVAIMKTTYSFFT

LIRNAQET

>HarmOR59a

MPSDQSRMFDPPLTVLKIFGVWEGRTPSKYYKTFSFLFLFVSWFFYNFLLTLNLVYTPRSLELFLREVIFYFTEITVTSKFLTVLLLRDKIL

EVFSIIDGDEFVGDYENKDGILYRTNKGYSFCWKVYNFLALIAYTCDIIMPVVIDLIRGTKSVLPICNYYFLSEDFRDSHFVILYLYQSIGM

YGHMMYNLNMDSLGWGLLAVGIAQIKVLNKNFTDLKLSAEESKLPLEIQDNIQKTRLLKLLRHYEAILNYCDAIQNLLSATFFFQFSFG

ALTTCVIMCSLLMPGTMVYRIFLVIYLLAMAGQIAVPGFFGTLLTHESEELVTAAYNCEWIERSQSFKRTLILFRERAGTPIIISGMKMFPL

SLATFVAIMKTTYSFFTLIRNAQET

>HarmOR42a

MPSDQSKMFDQTLTALKIFGVWKGRTPSKYYKYYTLVFLFFSWGFYNFLLTLNLVYTPRSLELFLRELMFYFTEMPVTSKFLTVLLLRD

EILEIFNFIDCDEFVGDYENKDGMLYKTNMRYRLVWKLYFVLSHVAFTCDIILPIVFDVIRGDKSELPICKYYFLSDEDRDSHFMYLFLYQ

SIGMYGHMFYNLNVDSFAAGLLAVAIVQMKILNKKFRNLKLSAEERNLPFEIQDNIQIIRMNKLLRHYEIILNYCDAIQNVFSATFFFQFS

FGAMTTCVIMCSLLMPATVEYRVFLVIYLLSMAGQILVPSLLGTLLTHESAELVTAAYNTEWIGRSESFKKSLMLFRQRAATPIKITGLKM

FPLSLVSFIAIMKTT

>HzeaOR33b

MPSDQSRMFDPPLAVLKIFGVWEGRTPSKYYKTFSFLFLFVSWFFYNFLLTLSLVYTPRSVELFLRELMFYFTEISITSKFFTVLLLRDKIL

EVFSVIDSDEFIGDYENKDGILYRTNKGYSFCWKVYNVLANIDYTCVIIMPVVIDLIRGTKSVLPICNYYFLSEDFRDSHFVILYLYQSIGM

YGHMMYNLNMDSLAWGLLAVGIAQIKVLNKNFTDLKLSAEENKLPLEIQDNIQKTRLFKLLRHYEAILNFGALTTCVIMCSLLMPGTM

VYRIFLVIYFFAMAGQIAVPGFFGTLLTHESKELVTAAYNCEWIERSQSFKRTLILFRERAGTPIIISGMKMFSLSLVTFVAIMKTTYSFFTLI

RNAQET

>OfurOR41

MNLLNFFKKYTEDDLINIQEHHFESFNKTYQWIAFTLTLGIMFPNPATDRFRIISINVLLVCVFPLAMMVLIDMYKCWMVKDIFNIIRHSTI

VGPFLGAFFKMFLMYYKRAQAKEILDEINRDHASFNFLPRKQQDIAFLNVKKGVFNVERLWAPIVSIAIMTFPGMAVVMTLYSYAFSDN

PKRYMIHEVKPPNSRDPEDMLKSPYFEILFVYETGSAIICVLNYTAYDGLFGIATNHACLKMSLCCMKLKEAFRCDSTEDMYKGILTFIEE

QKKMFRFVDLIQDTFNIWLGTILTSTMIQIGSLLFHISAGYGFDLRYTLFSFTSVVHIFLPCKNAATLKDMSTEMSTMIYSSGWERSRERRI

LRMIPFMVARAQVPNYITAFNLFIFDMELFVFILRTSYSMYTLIRS

>CpinOR41

MIFGSKKNKQLDDIEEQAEFTFRPFHETYRWIATSLTLGLMYPNPATDRTRLILIVTSLLMMQPVIVFILIDMYMCWLKRDIFNIIRHSTIIG

PFLGAFFKMLLMYYKRVQAKELIDEMNRDYSSYNRLPRHCRAAAAACVRASVTHTERLWAPLVGIAIMTFPGMAVVATLCSHVFAATP

QRYMVHDLNRPFREPEARFDSPYFETLFLIMFGGAIICVFNYTSYDGLFGLMTRHACLKMSIYCMRLRDGFRSDEPEELYRQLMEFIREQ

RRMFRFGALIQDAFNIWLGTILISTMIQVGSLLFHISAGYGFDVRYMLFSVCSVVHILLPCKNAANLKMMSMETATLVYCCGWERTSCK

RVRRMIPFLIARAQRPIRIMAFYMFQYDMELFVNIMRTSYSMFTLLRS

>HzeaOR85c

MDEELEFKPFHETYRVITFSLCIAMIYPNPRTEKWRLFSIPILIATVAPVAIMIFLDMYKCWTNGDIVNIIRHSTVVGPFLGGFFKMILMYH

KRVQAKQILDEFDRDHYMFNTVAETYKDIARASIRNCQIYSERLWACLVTTCVMTFPVMAIVLNIYNFMFKSEPTKYMIHDLEKPFSKE

PEERFESPYFELLFVYMFYAAILYVVNFTGYDGFFGLCVNHACLKMELYCKALEEAMMADKEEVYGRVIAVIREQCRMFRYVDLIQDT

FNIWLGIIFIATMIQICTCLYHITEGYGFDIRYMIFVYGAVVHIYLPCRYAAKLKAMSMETSNRFYCSGWERVDDERVRKMIVFMIARAQ

VPNEITAFNMMAFDMELFLSILQTSYSMFTLLRS

>HarmOR20

EFKPFHETYRLITFSLCIAMIYPNPRTEKWRLFSIPILIATVAPVAIMIFLDMYKCWKNGDIVNIIRHSTVVGPFLGGFFKMILMYHKRVQA

KQILDEFDRDHLMFNTVAETYKDIARASIRNCQIYSERLWACLVTTCVMTFPVMAIVLNIYNFMFKSEPTKYMIHDLEKPFSKEPEERFE

SPYFELLFVYMFYAAILYVVNFTGYDGFFGLCVNHACLKMELYCKALEEAMMADREEVYGRVIAVIREQCRMFRYVDLIQDTFNIWLG

IIFIATMIQICTCLYHITEGYGFDIRYMIFVYGAVVHIYLPCRYAAKLKAMSMETSNRFYCSGWERVDDERVRKMIVFMIARAQVPNEITA

FNMMAFDMELFLSILQTSYSMFTLLRS

>HarmOR85c

MDDELEFKPFHETYRLITFSLCIAMIYPNPRTEKWRLFSIPILIATVAPVAIMIFLDMYKCWKNGDIVNIIRHSTVVGPFLGGFFKMILMYH

KRVQAKQILDEFDRDHYMFNTVAETYKDIARASIRNCQIYSERLWACLVTTCVMTFPVMAIVLNIYNFMFKSEPTKYMIHDLEKPFSKE

PEERFESPYFELLFVYMFYAAILYVVNFTGYDGFFGLCVNHACLKMELYCKALEEAMMADREEVYGRVIAVIREQCRMFRYVDLIQDT
FNIWLGIIFIATMIQICTCLYHITEGYGFDIRYMIFVYGAVVHIYLPCRYAAKLKAMSMETSNRFYCSGWERVDDERVRKMIVFMIARAQ
VPNEITAFNMMAFDMELFLSILQTSYSMFTLLRS
>HvirOR20
EFKPFHETYKVITFTLCIAMIFPNPRTEKWRLISIPLLIATVAPVASMIFLDMYKCWTNNDIVNIIRHSTVVGPFLGGFFKMILMYHKRKEA
KQILDEFDRDHFMFNDFSETYKDIARASIRNCQIYSERLWALLVTTCVMTFPVMAIVLNIYNFLFKSEPTKYMIHDLEKPFSESPEERFESP
YFELLFAYMFYAAILYVVNFTGYDGFFGLCINHACLKMELYCKALEEAMVADREEVYGRVVAVIREQCRMFRYVDLVQETFNIWLGIIFI
ATMIQICTCLYHITEGYGFDIRYMIFVYGAVVHIYLPCRYAAKLKAMSMETSNRFYCCGWEKVDDERVRKMVLFMIARAQVPNEITAFN
MLAFDMELFLSILQTSYSMFTLLRS
>SlitOR41
MIFIDMYKCWMAKDIVNIIRHSTVVGPFLGGFFKMILMYHKRIQAKQILDEINRDYENINNYSEIYKDIARASVKNCQIYSERGWAITVV
TCVMTFPVMAISLNVYNFVFKSEPVKYMIHDLEKPFSEDPEDRFESPYFEIIFVYMFYCSILYVVNFTGYDGFFGLAINHACLKMDLYCK
ALEEAFKADAHEVCGRVIGVIKEQCRMFQFVDLIQDTFNIWLGIIFLATMIQICTCLYHITEGYGFDLRYMIFVTGAVIHIYLPCRYAAKLK
AMSLETANRFYSSGWEQVDDQRVRKMILFMVARAQVPNEIVALNMLAFNMELFVSILQTSYSMFTLLRS
>SlitOR30a
MEQQPEFTTFHETYKLITFALSVGMIYPNPKTEMWRLASIPFLIATITPLATMIFIDMYKCWMAKDIVNIIRHSTVVGPFLGGFFKMILMY
HKRIQAKQILDEINRDYENINNYSEIYKDIARASVKNCQIYSERGWAITVVTCVMTFPVMAISLNVYNFVFKSEPVKYMIHDLEKPFSED

PEDRFESPYFEIIFVYMFYCSILYVVNFTGYDGFFGLAINHACLKMDLYCKALEEAFKADAHEVCGRVIGVIKEQCRMFQFVDLIQDTFN
IWLGIIFLATMIQICTCLYHITEGYGFDLRYMIFVTGAVIHIYLPCRYAAKLKAMSLETANRFYSSGWEQVDDQRVRKMILFMVARAQVP
NEIVALNMLAFNMELFVSILQTSYSMFTLLRS

>CpunOR41

MIFDSKKNKQENDIEEQTEYTFRPFHETYRWIATTLTLGLMYPNPATDRTRLILIAASLLMMQPVIVFILIDMYMCWLKRDIFNIIRHSTIIG
PFLGAFFKMLLMYYKRVQVKELIDEMNRDYSSYNQLPRHCRAVAAACVRASVTHTERLWAPLVGIAIMAFPGVAVVGTLCSHVFAAAP
QRYMVHDLNRPFREPEARFDSPYFETLFLLMFAGAIICVFNYTSYDGLFGLMTRHACLKMSIYCLRLHDGFRSDEPAELYRRLMEFIREQ
GRMFRFGALIQDAFNIWLGTILISTMIQVGSLLFHISAGYGFDLRYMLFSVCSVVHILLPCKNAANLKMMVTYLHGLHVTNKKLM

>HvitOR32

MAMKFKSKMSFSEKHEVKEHQFPPCQERFNYITVMMTIGFMNPYSANPKLRFIGIACTLVVTIPLMFMIVFDIWRCWMRRDIFNIIRHST
IAGPLAGTYVKMLIMFVKRDNVKYMLDEINSDFEAYNNLPTHYKDLAVDHIKNIVFFTKGIWGGTISICAISFPSLPIIFTVYSYYMDDKP
VVYMVHEVVVPFMKPEARFESPLYDILFIYEVSLVMICLVNYTGFDGFFGLVTGHACLKMDMYCQRLEDAFRAGREEMWKEVVGFIE
DQKKLFRYTEIIQDTFNVWLGTIGVCTMIQIGSLLYHVFAGFGFDLKYFMFSITTVVHIFLPCYYSSKVKSTSEHTATRIYCSGWEGARSP
RAWRVMPFLLARAQVPKRITAFYMFEYGMEFFAFILRTSYSTYTLLRS

>MsexOR13a

MDEPEFKPFHKTYQIITFALSVGMIYPNPATDKMRLASIPISILTILPLACMIFLEMYQCWTQGDIVNIIRHTTVLGPFLGGLFKMFLMYHK
RKQAKQILDEFERDYHLYNSFTGDYLNIARDGIRNSLIYSERGWAITVTTCVMTFPVMAILLNVYNYT

FKFQATKYMIHDLNKPGASPEARFDSPYYEIMFFYMMYCSLLYVINFIGYDGFFGLSINHACMKMNLYCKMLEDAWKAEPNERYRRVV

AVIDEQCRMFEFVNLIQDTFNIWLGIIFLATMIQICTCLYHITEGYGFDLRYMIFVSGATIHIYLPCRYAAKLKAMAAETATHFYSSGWERV

NDRRLRNMLLFMVARAQTPLQITAFNMITFDMELFVSIMQSSYSMFTLLRS

>MsexOR7

MDEPEFKPFHKTYQIITFALSVGMIYPNPATDKMRLASIPISILTILPLACMIFLEMYQCWTQGDIVNIIRHTTVLGPFLGGLFKMFLMYHK

RKQAKQILDEFERDYHLYNSFTGDYLNIARDGIRNSLIYSERGWAITVTTCVMTFPVMAILLNMYNYTFKFQATKYMIHDLNKPGASPE

ARFDSPYYEIMFFYMMYCSLLYVINFIGYDGFFGLSINHACMKTNLYCKMLEDAWKAEPNERYRRVVAVIDEQCRMFEFVNLIQDTFNI

WLGIIFLATMIQICTCLYHITEGYGFDLRYMIFVSGATIHIYLPCRYAAKLKAMAAETATHFYSSGWERVNDRRLRNMLLFMVARAQTPL

QITAFNMITFDMELFVSIMQSSYSMFTLLRS

>BmorOR14

MSNYIFKPFHETYRIITFTMIAAMIYPNPATEKRRLIYIGLMLLSVIPLAFMIVTEMYEFFMASDLNNTIRHSTVIGPFIGGFVKVALMYYK

RRQANELVSEINRDHLAYNGLKGEDREIAASSIRNCQIYCELGWTLIVMSCGLSFPVIAILLKIHSFTFKLDSTKHMIHDINNPFTDDPEDR

FESPFFEIMFVYTFFSSFIYIINYVGYDGFFGLCINHACLKMKLYCRALEDAMRSDSRRHEKIVAVIEEQRRTYEYIALIQDTFNIWLGLIYV

ATMIQMCTCMYHIVQSFNIDVRYIIFVISIIHIYLPCRYAANLKCMAAETPTLIYCCGWESVSDLRIKRMMPFMVARSQVIVEITAFNMFAF

DMELFVWIMKTSYSMFTLMRS

>OfurOR4

MEEESLFDKSLKKITFAFRLTGLNIENDKRNFKQNCVYLFNFLWLNTDIVGALQWVLYGIASGKNFTELTYVAPCLTLSILGDIKAVFMIL

NEKKVHILMDNIRSLELKAKEFENSEREDMIKPEIKFLNIIISVLNVLNCLMIVVFDASPLILIAVKYFTTGQLELMLPFLDVYPFDSFDLR
YWPFAYIHQIWSECIVLLEICATDYFFFTCCTHIKIQFKLLQHQFQEIIPSRSVSAVDSIYQAAIRTKFQELIKWHQEIIRSANMLEGVYSKST
LLNFCTSSLVICLTGFNVTTIDDKAFVMTFIIFLFMSLLQVFFLCFFGDILMSSSIDVSNAVYNSRWYLTDVMMGRNVLLVQTRAQDPCKL
TAAGFADVNLRAYMKILSTAWSYFALLQTIYSTRS
>OfurOR25
MEEESLFDKSLKKITFAFRLTGLNIENDKRNLKQNCVYLFNFLWLNTDIVGALQWVLYGIASGKNFTELTYVAPCLALSILGDIKGVFMI
LNEKKVHILMDNLRSLELKAKEFENSEREDMIEPEIKFLNIITSVLNVLNCLMIVVFDASPLILIAVKYFTTGQLELMLPFLDVYPFDSFDL
RYWPFAYIHQIWSECIVLLEICATDYFFFTCCTHIKIQFKLLQHQFQEIIPSRSVSAVDSIDQAAIRTKFQELIKWHQEIIRSANMLEGVYSKS
TLLNFCTSSLVICLTGFNVTTIDDKAFVMTFIIFLFMSLLQVFFLCFFGDILMSSSMDVSNAVYNSRWYLTDVMMGRNVLLVQTRAQDPC
KLTAAGFADVNLRAYMKILSTAWSYFALLQTIYC
>OnubOR4
MEEESLFDKSLKKITFAFRLTGINIENDKRNFKQNCVYLFNFLWLNTDIVGALQWVLYGIASGKNFTELTYVAPCLTLSILGDIKGVFMIL
NEKKVHILMDNLRCLELKAKEFENTEREDMIEPEIKFLNIITSVLNVLNCLMIVVFDASPLILIAVKYFTTGQLELMLPFLDVYPFDSFDLR
YWPFAYIHQIWSECIVLLEICATDYFFFACCTHIKIQFKLLQHQFQEIVPSRSVSAVDSIDQAAIRTKFQELIKWHQEIIRSANMLEGVYSKS
TLLNFCTSSLVICLTGFNVTTIDDKAFVMTFIIFLFMSLLQVFFLCFFGDILMSSSMDVSNAVYNSRWYLNDVMMGRNVLLVQTRAQNPC
KLTAAGFADVNLRAYMKILSTAWSYFALLQTIYSTRS
>CmedOR25

SDFDESLKKINFAFRLTGLNIQNKKRTRKQNCIYLFNFLWLNTDIIGALSWVFEGILNGKNFTELTYVAPCVTLSVLGDVKAIYLLLNEKK
VLNLIEHMRNLEKKANGFVNSEQENLTRPDIKFLNVVIKVLNVLNCLMIVVFDLSPLILIAVKYFTRGELELMLPFMDVYPFDGYDLRY
WPWAYAHQIWSECIVLLDICATDYLFFTCCVHLRIQCRLLQHQFQEVIAPRSISAIDSIDMEEVKNKFKELLKWHQDIISTASMLEGVYSQ
STLFNFLTSSLVICLTGFNVTTINDKTFVITFITFLFMSLLQVFFLCLFGDILMNSSMEVASSVYNSRWYLSDVDMGKNVLLVQTRAQKPC
KVTAAGFADVN

>CpunOR25

MSDHTEKPNGISSCNLNQNLDNVEAKEGMQKEILFDQSLQKIKFAFRLTGLNIQNEKRSMKQNCVYLFNFLWLNTDIIGALFWVIDGML
SGKNFTELTYVAPCLTLSILGDVKAIYLVLNESKVHTLIANLRKLEQKANQFENFEQDKLIEPDIRLFNVVIKVLNVLNCLMIVVFDLSPLI
IIAVKYFTTGELELMLPFLDVYPFDSFDLRYWPFAYIHQIWSECIVLLDICATDYLFFTCCTHIRIQFRLLQHQYQEIIEPVNLSAVGTIDRSR
IKTKFKDLAVWHQEIINSASMLEGVYAKSTLFNFMTSSLVICLTGFNVTTIDDKAFVITFIIFLFMSMLQIFFLCYFGDILMSSSMEVTNAV
YNSRWYLSDVGMGRNVLLVQTRAQKPCKVTAAEFADVNLRAFMRILSTAWSYFALLQTVYSSRSK

>CpinOR25

MSEHTEKPDGSSCDLNQNLDSVDAKEAIQKEILFDQSLQKIKFAFRLTGLNIQNEKKTMKQNCVFLFNFLWLNTDIIGALFWVIDGMLS
GKNFTELTYVAPCLTLSILGDVKAIYLVLNESNVQTLIAKLRKLEQKANEFENVEQEKLTEPDIRLFNVVIKVLNVLNCLMIVVFDLSPLII
IAVKYFTTGELELMLPFLDVYPFDSFDLRYWPFAYIHQIWSECIVLLDICATDYLFFTCCTHIRIQFRLLQHQYQEIIEPVSVSAVGTIDRSR
ARTKFKDLAIWHQEIINSASMLEGVYAKSTLFNFMTSSLVICLTGFNVTTIDDKAFVITFIIFLLMSMLQIFFLCYFGDILMSSSMEVTNSV
YNSRWYLSDVGMGRNVLLVQTRAQKPCKVTAADFADVKARRLLRAFMRILSTAWSYFALLQTVYSSRS

>HvitOR25

MPGQLLFDKSLEKINFLYQLTGFNVCSNKKNRTFKQNCVFLFNFLWVNTDNVGALYWIINGILGGKPFTEVTFVAPCVSMTCLGIIKGVF

FIINERKVSFLMEKLKALEEKRQEFGHIDDDEVIKPEIRFLEAVTKVLNVLNVLMVIVFDLCPLIVIAVKYFKTGQLELMLPFLDVYPFDS

FDLRYWPFAYMHQIWSECIVLLQICAADYLFFTCCTYIRINFRLLQRQFEQVISSNSVSIVKPNKGELKARFDSLIGWHQEIISSANMLELV

YSKSTLFNFLISSLVICLTGFNVTAINDVAYVMTFMIFLFMSLLQIYFLCYFGDLLMNSSVEVANAVYKSRWYLSDAGVGRSVALIQVRA

QHPCKLTAAAFADVNLKAFMKILSTAWSYFALLQTIYGSHA

>GmelOR67c

MTLQPLLFDESLKKIEFLFNWTGLSLESGKRTPIDTLKCRAIFIFNFIWLNSDIFGAISWFADGIENGKSFTELTYLFPCTTLSFLASMKSIIH

IRKENMVNELIDDMRSLETQKLREKTNGSVLEMAKKEIRFLNIVLNVLNTFNVMMVVVFTVVPLILIAIHYLRTSEVQLMLPFLNVYPF

DPYDIRFWPFVYTKQIWSECIVLLEICATDYFFYTCCTHLRIQFQLLHEAIVSIIPSAGISAKVSYDEEMRSSFVEFIKWHQKLIKSADVLE

VIYTRSTLLNFVISSVVICLTGFNVTTNTDLAFVVLFLTFLFMGLLQAYFLCFFGDLLMRSSLEINEAIYNCRWYLAESTFGNDLHIVQMR

SQKPCKLTAAGFADVNLRAFMRVLSSSWSYFALLRTLYSTHDTQ

>OnubOR92a

MAEQPIDRSLRKIRFIFRYSGMNLEERSRTWLQNFIYMFNFLWILTDIVGAFNWIFEGVNKGRNFIELTIVTPCLSLGMMAEFKTGFVVAH

EERLFRLINNLREMETKRLVGPSANKIVKEESKFLYALIFALIVINVVLIILFGFGPLVVIVVKYFVSGKLELTLPFLDVYPVDCYDLRIWPF

AYIHQIWTTVIVLSEIFAVDFLFYICCTHIKMQFKVLNLEVTKVIAEERSARIIEDETSRNKFNELVKWHQDIIGSASMLEEIYSKSNLVNYL

SSSLIICLTGFNVTAINNMTIVVTFAILCVAGMLQIFFVCFFGDMLKDSSTAVSTAVYNSRWYLADAAFGKSALIMQTRAQKPCTVTAAGF

ADVNLKAFMKILSSSWSYFALLQTVYRTSEN

>HarmOR12

MMEEEPLLIDKTVKKIEFLFRCTGINIKSGTKTRKDMIKSRTVYIINFLWLNIDLAGAVMWFFTGIANSKSFTELTYVAPCITLSFLGNLKS

LFLILREKHVDKLIQVLRDLEINEKARPKSEETDAIIKYEHNFVTTVISVLNVLYFVLLVAFALSPVSLVALKYFTTNELELLLPFLIVYPFD

PYDIRYWPWVYLRQIWSEVVVIIDICTADYLFYTFCTYIRMQFRLLKHYIERVIPEEDASGRLTNIEDVRAEFVLLIKWHQDLISSANMLE

TVYTRSTLFNFVSSSVLICLTGFNVMAISDVAFVATFLSFLFMSLLQIFFLCFFGDLLMTSSTEISEAVYNCRWYLADTSLGKDLLLVQTRA

QTPCKLTASDFSEVNLKAFMKILSTAWSYFALLQTLYGAPT

>HassOR12

MEDEPLLIDKTVKKIEFLFRCTGINIKSGTKTRKDMIKSRTVYIINFLWLNIDLAGAIVWFFTGIANSKSFTELTYVAPCITLSFLGNLKSLF

LILREKHVDKLIQVLRDLEINEKSRPKSEETDAIIKYEHNFVTTVISVLNVLYFVLLVAFALSPVSLVALKYFTTNELELLLPFLIVYPFDPY

DIRYWPWVYLRQIWTEVVVIIDICTADYLFYTFCTYIRMQFRLLKHYIERVIPEEDDAGRLTNIEQVRAEFVLLIKWHQDLISSANMLETV

YTRSTLFNFVSSSVLICLTGFNVMAISDVAFVATFLSFLFMSLLQIFFLCFFGDLLMTSSTEISEAVYNCRWYLADTSLGKDLLLVQTRAQT

PCKLTASDFSEVNLKAFMKILSTAWSYFALLQTLYGAPT

>OfurOR92a

MAEQPIDKSLRKIRFIFRYAGMNLEERPRTWCQSFIYVVNFLWIATDIIGEINWIFEGVSKGTSFVELTHVAPCLSLGTMSEFKTAFVVAHE

KSLFRLIGNVREMERKRLVGPIAHKIVKEESKFLYNLVFAMKMVNWVLVVVFDFGPLVWIVVKYFIYGELELLLPIIDIYPFDCYDLRIW

PFAYIHQIWTAWVVVTEILGVDCLFYICCTHVMIQFKILSHEVTNVIAESRSAKRIEVTQLREKFNELVKWHQDIINSAGLLEDIYSKSTLV

NFLTSSLIICLTGFNMTALDNVRMTVAFAFFVVAMLQIYFLCFFGNMLMDASTDVSTAVYNSRWYLSDAAFGKSALIMQIRAQKPCIVTA

AGFAEVNLRAFMKIISTSWSYFALLRTVYQDI

>OfurOR23

MAEQPIDKSLRKIRFIFRYAGMNLEERPRTWCQSFIYVVNFLWIATDIIGEINWIFEGVSKGTSFVELTHVAPCLSLGTMSEFKTAFVVAHE

KSLFRLIGNVREMERKRLVGPIAHKIVKEESKFLYNLVFAMKMVNWVLVVVFDFGPLVWIVVKYFIYGELELLLPIIDIYPFDCYDLRIW

PFAYIHQIWTAWVVVTEILGVDCLFYICCTHVMIQFKILNHEVTNVIAESRSAKRIEVTQLREKFNELVKWHQDIINSAGLLEDIYSKSTLV

NFLTSSLIICLTGFNMTALDNVRMTVAFAFFVVAMLQIYFLCFFGNMLMDASTDVSTAVYNSRWYLSDAAFGKSALIMQIRAQKPCIVTA

AGFAEVNLRAFMKIISTSWSYFALLRTVYQDI

>CpinOR23

MAELPVDQSLKKIKFLYRLSGMNIEDRDKNTSEKAVYMFNFLWILTDIILAIEWGIVGFVKGLDFIQITHVLPCLNLGMLTEMKTLYLVLY

EKKLKQLLRDLRDLERIRLPNNSYAKKIADTDAKFLHNLIKVTWTVNFCLMILFNSGPLALMAVKYYLTGKLELFLPFLDVYPFDSYDL

KYWPWAYLHEVWTACIVLSEIFAVDFLFYVCCTHIGIQFKVLKRELEQLITGKGLLADRDDQLKEKLADIVRWHQQIISCAEMLEVMYS

KSTLFNYISSSLIICLTGFNSMVIDDMAIVISFFMLAVVEVLQIFFLCYFGGKLIDSSTDVSSGAYNSKWYLTDIPTRKTILLIQTRAQNPCK

LTAAGFADVNLRAFMKILSTSWSYFALLQTMYGSSKVSH

>HassOR7

MKIKMSKPLKFDQSIEKLGVLFRFSGMNIKNKIVTPLDTIKYGWLYTLNFLVVFSAIIGSVYYVILGIKQGKNFIEVTSVAPCLTFSILSMIK

SLYHLMYEEHIQELIDLLTELEIRENNREKCIEKEEIIANETGFLNKVINVLYVLNCSMIVVFDMTPIVMIAVKYYKTNEFEMLLPYLDVFS

FIPYELKYWPFAYIHQIWSECVVLLDMAAADYLFFTCCTYIRVQFKLLQYDFERMIPDRSISKGVFYEENELRNKFTELLKWHQDIIYSST
ILEIIYSKSTLFNFLSSSLVICLTGFNVTIVDDIVIIITFLTFLSMALMQVFFLCFFADLMMTASLEITNSVYNCKWYSANIKVGKQILFVQTR
AQEPCKLTAAGFADVNLNAFMRVLSSAWSYFALLQTVYGAK

>HarmOR7

MKIKMSKPLIFDQSIEKLGVLFRFSGMNIKNKIVTPLDTIKYRWLYTLNFLVVFSAIIGSVYYVILGIKQGKNFIEVTSVAPCLTFSILSMIKS
LYHLMYEEHIQELIDLLTEHEIRENNREKCIEKEEIIANETGFLNKVINVLYVLNCSMIVVFDMTPIVMIAVKYYKTNEFEMLLPYLDVFSF
IPYELKYWPFAYIHQIWSECVVLLDMAAADYLFFTCCTYIRVQFKLLQYDFERMIPDRSISKGLFFEENELRNKFTELLKWHQDIIYSSTI
LEIIYSKSTLFNFLSSSLVICLTGFNVTIVDDIVIIITFLTFLSMALMQVFFLCFFADLMMTASLEITNSVYNCKWYSANIKVGKQILFVQTR
AQEPCKLTAAGFADVNLNAFMRVLSSAWSYFALLRTVYGAK

>CpinOR19

MDGFEIYHKEFLKPLMVTLKMLKHSNVRFCKSEVPFLERNWRFFYIVPIMILHTISLTAFIVNLIMDGNDPFEDAYMIPICFISLEHCIKMC
ILMKKREQIRDVITNIGNIWRSTGLTDDQKKMKEELLKKLYNAGLVFNNIGMVVTIQYLMVPLFETLVRRLVLEQEVELMLPLTINYPFEI
DTWPLYLAIYGFQVYSLHCTACAYLGFAWLLVVLTCYLNIHFLLLQEDLILVKPETSNRLSYLPEEEGEITHYWEPSTLTIDDFIRQHQTVI
LLADKLNAAVNKITFTIILFATLNISFFAIAVKASAGAVDMVNNFGAILVIMMNIFILCYCGELLRSSSSGIALAAGKNLWYKGDLGYQKKI
RFIMMRSQKPCSLTALDVSPISLETFNKVLKTTWSYFSLASQMYDERN

>HarmOR4

MEEFQNLPEEYRENIEHSLNLLTNGNILIFHKPNTFFEKSFRPYVVICSVVTYLASLTMYLGKVFRGELQLTELSYVVSVYMVSVQAILK

AAIAALNKEEIGLIILELGRLWRTQDLTEEQINKKNAQLKRLKFCYAVVYWINMIGSWQYILAPLLETVFRKFILHEECGLLLPFSCSFPFD

PSGSWGRYIGVYIFETYSMFRIVYFYLGVEFLMISLCSNLATEFTLLQEDLQNVKPERNNIALKAIIANHQKLISLSQQLDNVFDKVIFVN

LTSAAVPLCFFGFSAKVAHGVLQMVNNFAAVISLILPLCNMCYFGEQIREASAGISDAVYHNLWYRGDIRFQKILCFVQRRSQKPCCMTS

YKFSPIALTTFTTVLSTTWSYFSLASSLYEGEN

>HzeaOR4

MEEFHNLPEEYRENIEHSLNLLTNGNILIFHKPTTFFEKRFRPYVVICSVVTYLASLTMYLGKVFRGELQLTELSYVVSVYMVSVQAILK

AAIAALNKEEIRSIILELGRLWRTQDLTEEQINKKNAQLKRLKFCYAVVYWINMIGSWQYILAPLLETVFRKFILHEECGLLLPFSCSFPFD

PSGSWGRYIGVYIFETYSMFRIVYFYLGVEFLMISLCSNLATEFTLLQEDLQNVKPEHNNLALKAIIADHQKLISLSQQLDNVFDKVIFVN

LTSAAVPLCFFGFSAKVAHGVLQMVNNFAAVISLILPLFNMCYFGEQIREASAGISDSVYHNLWYRGDVRFQKILCFVQRRSQKPCCMTS

YKFSPIALTTFTTVLSTTWSYFSLASSLYEGEN

>OfurOR52

MPEKVYCALKKLTQIELDFLSILGSKLFLYPFIGRTKRVYACYMLVSLLLIVTAAQLLTALLVADVKEWIEIINVAPNLAVVLMALLKYTK

VHVNRDLYKKIFEHFRDELWDVISDSVEHQNIVTQYAAKTKFISRFLIYYSIPLVIFVDSFPRIIMYLERELLGSDDTKYLYPFDGWYPFDK

VDWYYTVYLWESFMTMNVVVVYAFTNMIHSSYTLFICLELKILGSSIKNLVTSEDVANIVNRTKLQETHGDIKRKLKTIIIRHQLLAQIVS

EFDIVQGDVTLANYLFGSVFLTLTIFSSTVVDNMYKSLRYFFMFCSLLIEVFFYCMIGQILTDDSDCLTDAIYSADWPYANTETKMALLII

MTRTQRPFKYTAKGYIAMNLNSFSGVCSMSYQFFNLLRTAYSQ

>CmedOR33

MSDKSYDWMKNKLLHELNFLRSLGIMIFIYPFIGQSKTANIGYGFVFFLIILTATQLIITLCLKDFKDWVQIVNVVPNLAVVLMSALKYIT

VYMNQPIYHKIFEHFRNDLWDVVHDCKQHKKIVVKYSVLAKFVTRFLFYYSVVLIIFVFSFPRLMMYLETFLTGEECEALYPFDGWYPF

DKAHWYYVAYIWEGFMTSVVVCIYGIPNMFNSSFTIFMCMELKVLGRHIENLITRDDVVNLSKYESVRKTHLDIKRRLRYIIIRHQFLAQ

ISAEFDAVLGDAMLVNYVFSSVFITLTIFTATAVENLYMRLRYFFMFCSLMVEMFHQCMIGQILSDHSEELAEAIYSADWTYADNSTKKM

LLILMVRTQRPFEYTANGYLAMNLQSFSGICSMSYQFFNLLYTAYN

>CpunOR52

MPDNHAYSYNVMQKKMLHEIKFLSLLGVATFVYPFIGRSKIVLGCYSFIYLLVLLTATQLIITLYLKDFSDWVEIINVVPNLAVVLMAVLK

YSKVHHNQRFYKKLFDHFRNDLWDAVSDCEQHRKIVTKYTDISRYVTRFLFYYSVVLVIFVFSFPRFIMYLQKIITGEECHLYPFDGWYP

FDKVSWYYVAYIWECFMTFVVVCIYGFAGIFTSAITIFICMELKVLGSSIQMLISPSDAAKLTKSPNDKKIHQDIRKRLRSIIIRHQVLAKLS

ADFDGVLGDIMLINYVFSSVFITLTIFTATVVENLYMRMRYFFMFCSLMVEVFQQCMIGQILSNHSEELSESTYFADWTYADNNTKIMLLI

LMTRTQRPFEYTANNYLAMNLQSFSSICSMSYQFFNLLYTAYN

>HvitOR5

MPVTEYKFLRANFLLELDYLSNMGSKVFIYPFIGRSRVVIYSFGTIYAMLLITSAQLFGSLCLTGFKDWFEIINVAPNLGVVLMAVLKYTK

IHSNQKVYDEIFTHFRDKFWEGVFNTDGHKKIVTKYTSFTKFVLRFVVYYSLPLVIIVNSFPRLIMFYENDVNGKESVYLYPFDGWYPFN

KVKFYYVAYFWESLMTAVVIGIYTFTNMIHGSYTVFICMELKVLGNSMENLISSKDVDKIKKGINVRKTHADIKNRLRNIIIRHQFVTHIA

RELDNVLGDSMLLNYVFGAVFICLTVFTATVVDDLYKTLRYFSMFCSLMVEIFFQCIIGQVLSDHSEQLTESIYSADWPNADKESKTMLLI

FLAGTQRPLQFTANGYLAMNLDSFSGICKLSYQLFNLLLTMYS

>CpinOR52

FLSLLGVATFVYPFIGRSKVVLGCYSLIYFLLLLTATQFIITLYLKDFSDWVEIINVVPNLAVVLMAVLKYSKVHLNQSFYKKLFDHFRND

LWDVVYDCDQHRKIVIKYTNISRYVTRFLFYYSVVLVIFVFSFPRFIMYLQKKITGEECHLYPYDGWYPFDKVSWYYVAYIWECFMTFV

VVCIYGFVSIVTSAITIFICMELKVLGSSIRMLISPSDAAELTKSPNDKIIHQDIRKRLRSIIIGHQVLAKLSADFDGVLGDIMLINYVFSSVFI

TLTIFTATVVENLYMRMRYFFMFCSLMLEVFQQCLVGQVLSDHSEELSESIYSADWTYADNNTKIMLLIFMIRTQRPFEYTANNYCAMN

LQSFSRIFSMSYQFFNLLYTAFN

>MsexOR17

MLKTVDSLKQELLFEMDYVNNIGQKIFAYPFIGRSKTAFFFYRTTYFLVVLTAIQLFATLCLTKFKDWFEIINIAPNFGVCLMIVIKYSKIYS

HRSVYDKILRHFRYDLWDVVSDSKDHRAILQQYTKTTRLIVRFQYYYTLILIVIVDLFPRIIMLYEAEFLGNEDQYLYPFDAWYPFDKVK

WYYPAYIWESFMTAVVIFVYVFANMIHISYTRHICMELKILGNSMENLITAEDIVTITKNKDVDKLHENIKSKLKVIIKRHQYLAEITSELD

DLLGDAMLLTYIFGSVFICLTAFTATVVGDIYMTVRYVSFFLSLLVEVFVQCIIGQILIDHSEKFERAIYSADWPHSELKTKKMLLILLTRA

QKPFVYSANGYLVMNLDTFCGICSLSYQFFNLLRTAYN

>SlitOR85c

MEYEHKSYDEIKQDFLGEMDFISNIGTKMFIYPFIGRSKIITYCFYITYGLLFLTSAQLIVTLCIICVESFDWFEIINVAPNIGVCFMILIKYKK

INDNKELYNEIFKHFRFDLWDTVFDTEEHKKILNRYTQTTRLILRFEFYYTIGLAVIVDLFPRIIMIYQNDILDKEKQYLYPFDGWYPFDKI

EWYNTAYIWESFMTTVVIFIYVFVNMLHISFTRYICFELKILGSTMEDLINEDDVVKIKKGREIANVHKKISNKLKFIISKHQFLARITSDL

DQVLGDGMFLTYMFGSVFICLTAFTATVVDDLYKSMRYFSFFCSLLVEVFFQCIMGQLLIDHSNKLEKAIYFADWVYANNSTKKMLLIFL

IRSQKPFEFSANGYLTMNLDTFSGICSLSYQFFNLLRTAYSE

>HzeaOR92a

MADKSYDSLKENFLHEMDFISNLGVKMFIYPFVGRSKLATYCYHITYGLLFFTMTQLMITLTLICINDIDVFEIINVAPNIGVCLMILIKYG

KIHDNKVLYDQINKHFRFDIWDAISDTPAHKEILNNSTWMTKIILRFEFYYTIGLVIVIDLFPRLIMVYQNNILGKEKQYLYPFDGWYPFD

KIQWYDAAYIWESFMTTVVIFVFVYVNMLHMSYTRFICLELKILGSSMEGLISNEEVVKIKKRKDVDRIHCNIRKKLKFIICKHQFLDRIV

SDLDEVLGDGMFLTYLFGSVFICLTIFTATVVDNLYKSMRYFSFFCSLLVEVFIQCIIGQLLIDHSNKLERAIYFADWVYADSDTKKMLLIF

LMRSQKPFGLSAKGYLTMNLDTFSGVCSLSYQFFNLLRTAYSE

>HarmOR92a

MLLQQCIAQYIQFIDLKKCKITLLSGQAVRKNHFLTMADKSYDSLKENFLHEMDFISNLGVKMFIYPFIGRSKLATYCYHITYGLLFFTM

TQLIITLTLICINDIDVFEIINVAPNIGVCLMILIKYGKIHDNKVLYDQINKNFRFDIWEAISDTPAHKEILHNSTWMTKIILRFEFYYTIGLVI

VIDLFPRLIMVYQNNILGKEKQYLYPFDGWYPFDKIKWYDAAYIWESFMTTVVIFVFVYVNMLHMSYTRFICLELKILGSSMEGLISNEE

VVKIKKRKDVDRIHCNIRQKLKFIICKHQFLDRIVSDLDEVLGDGMFLTYLFGSVFICLTIFTATVVDDLYKSMRYFSFFCSLLVEVFIQCII

GQLLIDHSNKLERAIYFADWVYADSDTKKMLLIFLMRSQKPFGLSAKGYLTMNLDTFSGVCSLSYQFFNLLRTAYSE

>BmorOR85d

MAKTFDCIKDEFLIEMDYISKIGSKLFVYPFVGRSKFTMCCYYVTFFFLVLTSVQLFVTLCLTRFESSFEVINIAPNLGVCLVIIIKYSKIHT

KRISYQKFYNHFRYELWDVVLDSVDHRNVLETYVKTARLIARFFIYYGITLSLLVALLPRIIMLYENSVLQKNLYLYPFDGWYPFNKIKW

YYIAYVWESVMTTVVIINFVCTNTIHISYTRLICMELKVLGISIENLLKSKVRDSITQSKIEDFHENIKVNFKTILKRHQLLGNVVSELNIIM

GDGMLLTYISGSVFICLTAFTATVVNDFYMTLRYFSFFCSLLVETFIQCIMGQLLIDHSEDFENSIYFTDWPIADLSTKKMLLIMLIRAQKS

YVFTANGYFIMNFDTFGGICSLSYQLFNLLRTTYNKEL

>OfurOR94a

MLSTVCVVKAFTFVTWQDHWKDVIDNVSLLEKRQLSKKDKITDKIISEYTNYARRVTNFYWTLVAATVFTVILAPLVCFLSSPDTRERIR

DGYEPYPEIMSSWVPFDRSRGLGYWVTVLEHILICFYGGGIVATYDSNAVVLITFFAGQMKLLSVNCSRLFDDEKEMTYEDDMEKIRAC

HYHHLMLIKYSKILNSLLSPVMFLYVIICSLMICASGIQLTTEGTTTMQRIWIAEYLMALIAQLFLYCWHSNEALVMSNKVDDGVYASA

WWSRSIRVRRCVLLLAGQLRKSVVFTAGPFTKLNVPTFIAILKGSYSYYTLLNNKDD

>OfurOR22

MLRNFLLSLENDNHPLLSPTLWGLQKWGLWQPNKVLNSNISNFIHFAATLFVISQYVELWLIRDNLNYALRNLSVTMLSTVCVVKAFTF

VTWQDQWKDVIDNVSLLEKRQLSKKDKITDKIISEYTNYARRVTNFYWTLVAATVFTVILAPLVCFLSSPDTRERIRDGYEPYPEIMSSW

VPFDRSRGLGYWVTVLEHILICFYGGGIVATYDSNAVVLITFFAGQMKLLSVNCSRLFDDEKEMTYEDDMEKIRACHYHHLMLIKYSKI

LNSLLSPVMFLYVIICSLMICASGIQLTTEGTTTMQRIWIAEYLMALIAQLFLYCWHSNEALVMSNKVDDGVYASAWWSRSIQVRRCVL

LLAGQLRKSVVFTAGPFTKLNVPTFIAILKGSYSYYTLLNNKDD

>OnubOR94a

MLRNFLLSLENDNHPLLSPTLWGLQKWGLWQPNKGLNSKINNFIHFAATLFVISQYVELWLIRDNLNYALRNLSVTMLSTVCVVKAFTF

VTWQDQWKDVIDNVSLLEKRQLSKKDKITDKIISEYTNYARRVTNFYWTLVAATVFTVILAPLVCFLSSPDTRERIRDGYEPYPEIMSSW

VPFDRSRGLGYWVTVLEHILICFYGGGIVATYDSNAVVLITFFAGQMKLLSVNCSRLFDDEKEMTYEDDMEKIRACHYHHLMLIKYSKI

LNSLLSPVMFLYVIICSLMICASGIQLTTEGTTTMQRIWIAEYLMALIAQLFLYCWHSNEALVMSNKVDDGVYASAWWSRSIQVRRCVL
LLAGQLRKSVVFTAGPFTKLNVPTFIAILKGSYSYYTLLNNKDD
>HvitOR9
NFIHITATLFVVSQYVELWCIKSDLDLALRNLSVTMLSTVCVIKAGTFVMWQEHWKNVLQYVSNLEKDQLSRKDKVTDTIISEYTVYSR
RVTYFYWSLVSATVLTVILAPFAGYISSDEYRARISAELAPYPEIMSSWVPFDRTRGIGYWASVLEHSLICFYGGGIVAIYDSSAVVLMAFF
AGQLKLLSENCSRMFESDVVDMEQEMLKIAECHHHHKNLIKYSKILNSLLSPVMFLYVIICSLMICASAIQITTDGTTTMQRIWIAEYLM
ALIAQLFLYCWHSNEVSVMSDKVDDGIYKSSWWSRGVVMRRSVLLLAGQLRKNIVFTAGPFTKLNLPAFITILKGSYSYYTLLSNKED
>MsexOR4
MLERLSKYLENPNHPLLGPTLYGLKCWGMWQPLGVNRIIYNAIHFFAILFVISQYVELWFIRSNMELAIRNLSVTMLSTVCVIKAGTFVF
WQKSWNDVIDYVSGLENIQLSKRDRITNSVISEYTKYSRSITYSYWVLVTATVFTVILAPLVGFLSSSDKDLMLNGTLAYPEIMSSWLPF
NRSRGFGYWVAAIEHSLICFYGGGVVANYDSNAIVLMSFFAGQLKLLSINCARLFNDNEVLKYSDTMKRIKDCHHHHVEIVKFSMVLN
SLLSPVMFLYVIICSLMICASAIQLTAEGTSNMQRIWISEYLMALIAQLFIYCWHSNEVLHMSSKVDEGVYASHWSAQNVRVRRSVVLLG
GQLRRPIVFTAGPFTKLTISTFVAILKGSYSYYTLLSKKED
>SlitOR42
MVFKFLTVSEDPDNPLLGTIISLLRCGGLWEKDRLKNFLHNLVHFVAFIFVLSQYVELWVIRNDLEMAMRNLSLTMLSTVCVFKACNLV
FWQNTWKELFDYVSELERSQLAKKDDTINKIIFQYVKYARRVTYLYWSLVTATVLIVSLAPLLIYWSSPTYRHNIRNGTLPYPEIMSSWT
PFDRTRGIGFCVATVYQMSACVYGGIVVANFDSTAVVIMTFFAGQLKVLSANCSRLFGDGNELINYDETVKRIRECHLHHLYLVKFSAVL

NSLLSPVMFLYVIICSLMICASAAQLTTEGTTTVHQIWIAEYLMALIAQLFLYCWHSNDVFYLSNQVDDGVYSSAWWSQNVRTRRNLLL

LGGQLRKPIVFTAGPFTKLNMATFVTILKGSYSYYTLVAKKED

>SlitOR2

MVFKFLTVSEDPDNPLLGTIISLLRCGGLWEKDRLKNFLHNLVHFVAFIFVLSQYVELWVIRNDLEMAMRNLSLTMLSTVCVFKACNLV

FWQNTWKELFDYVSELERSQLAKKDDTINKIIFQYVKYARRVTYLYWSLVTATVLIVSLAPLLIYWSSPTYRHNIRNGTLPYPEIMSSWT

PFDRTRGIGFCVATVYQMSACVYGGIVVANFDSTAVVIMTFFAGQLKVLSANCSRLFGDGNELINYDETVKRIRECHLHHLYLVKFSAVL

NSLLSPVMFLYVIICSLMICASAAQLTTEGTTTVHQIFFFLYLMALIAQLFLYCWHSNDVFFLSNQVDDGVYSSAWWSQNVRTRRSLLLL

GGQLRKPIVFTAGPFTKLNMATFVTILKGSYSYYTLVAKKED

>HzeaOR2

MVLKFLDRLEDPNNPLLGPTVLALRYGGLWQKDRVKHFLYNLVHFIAFLFVVSQYVELWIIRKNLEMAMRNLSLTMLSTVCVFKACNL

MLWQNSWKELIDYVSELERSQLSKNDAVVNKIISDYVKYARRVTYLYWALVTATVVTVILAPLFIYLSSPNYQESIKNGSAPYPEIMSSW

TPFDRSRGLGFCGATLYQMLACFYGGTVVANFDSTAVVIMTFFTGQLKVLSVNCERLFGDGNELVDYDETVKRIKDCHLHHYYMVKFS

SVLNSLLSPVLFLYVIICSLMICASAVQLTTEGTGNMQRIWIAEYLMALIAQLFLYCWHSNDVLYMSNKVDDGVYSSAWWSQNVRIRRS

LLLLGGQLRRPIIFTAGPFTLLNMATFVAIMKGSYSYYTLLAKKDD

>HarmOR2

MVLKFLDRLEDPNNPLLGPTVLALRYGGLWQKDRVKHFLYNLVHFIAFLFVVSQYVELWIIRKNLEMAMRNLSLTMLSTVCVFKACNL

MLWQNSWKELIDYVSELERSQLSKNDPVVNKIISDYVKYARRVTYLYWALVTATVVTVILAPLFIYLSSPNYQESIKNGSAPYPEIMSSW

TPFDRSRGLGFCGATLYQMLACFYGGTVVANFDSTAVVIMTFFTGQLKVLSVNCERLFGDGNELVDYDEAVKRITECHLHHYYMVKFS
SVLNSLLSPVLFLYVIICSLMICASAVQLTTEGTGNMQRIWIAEYLMALIAQLFLYCWHSNDVLYMSNKVDDGVYSSAWWSQNVRIRRS
LLLLGGQLRRPIIFTAGPFTLLNMATFVAILKGSYSYYTLLAKKDD

>GmelOR4

MLKTLRALENPDHPLLGPTLKGLKLWGLWMGAGWNPVIYNTIHAWAVFFVISQYIELWYQRSDLPSALRNLSVSMLSTVCIVKSITFVY
WQNEWREVLNFVSRLERYQLSKNDKTTNCIIKKYTDYSRRITYVYWSLVTATVLTLILAPLVDFLCSPEDRLIIKNGTKPYPEIMSSWAPF
DRTRGFGYWTVTIEQSLICFYGGGIVANYDTNAFVLMSFIAGQMKIVRKNCERLFEGKDARYDVILKRITYCHYHHLSLVKFAKLFNSL
LSPVMFLYVIICSLMICASAIQLTTKSTTSMQKIWIAEYLLSLISQLFLYCWHSNEVFVTSNEVDRGLYKSDWWSSNTRVRRSLLLLGGQL
QKRVVFTAGPFTTLSLPTFITILKGSYSYYAILSEKAD

>OfurOR17

MEKSPNFEFGYALVAASVWFLCYVPANVTSVLIVFAGYIEAQMLALTQELLHIWADAEQHYANINLNTLKRGTFVDAKYKKRVINEFIT
MRLHDIIRKHATNVHILHLLEEVFKGAIAFEFLFLIMGLIAELLGGLQNTILEMPYAFVQVAMDCWTGQRVMDASAEFAAAVYACNWE
MFDVPNMKIVLLMLASAQKTMKLSAGGVTMLSFECLMSVVKNIYSAYTTLRSAFTINTHAH

>CmedOR29

MEKSPNYEIGYTLVGASVWFLCYVPANVTVFLIVFAGYIQAQMLALAEELLHLWPDAELHYKNLDFSEFRRYNFNKERVLNDFIRRSLE
DIIRKHAMNVNLLKQLEELFAGAIALEFMLLMLGLIAELLGGLQDTYLEVIYAFVQVAMDCWIGQQVMDASVAFERAVYGCRWENFD
VSNMKVVLQILGNAQRTMKLSAGGVTMLSFASLMSVVKSIYSAYTTLRTAIK

>OfurOR9

MLYYSQLTVRRKVIKNIVDGYLACDAQTLKSDRFRQNLLKGLRIVKKRGLIFWMVIIGNGTIYIMKPIVTPGRHIMEDLFIIYGLEPMFES

PNYEIGFLLTAGGVICTCYLPANITALLTVLIGYTEATMLALSEELVHLWSDAQEHYNKYLLETQVDNAGALVTPNDDIKNQIINKYIKQK

LEEIVKIHTTNINLIQQIEHVFRGAIAVEFLLLITGLISELLGGLENTYIEMPFALMQVAMDCLTGQRMMDACDKFENSVYDCKWENFNV

ANMRTVLLMLQNAQKTMVLSAGGMTQLSFTCLMTVIRSIYSAYTTLRSMMA

>MsexOR28

YCDLPTMLWNVAFLLRGITLNIDSRHKKRIPFILYVITVVITLSYFYVFLVSMAWFVFIRSPQTGDLLAAMVVLSLGISSEIGTLKFFYTFV

YINKVQKIVKDYLECDSLIVPGSRFSGNLLRALRNVKKRAIVYWLVIIINGITYVTKPMFMRGRHHMEDRYVIYGLEPMFESPNYEFAYF

LMTAGLCFICYPPANVTVFLIVVVGYTEAQMLALSKELLHLWTDANEHYQKNINQHETTLINAQASKNKIINDYVRYRLKEIIKMHAFN

IHLVRQVEFVFRGAIAIGYVFLTLGLIAELLGGLENTYLQIPFALIQVAVDCYTGQKVTDASLIFERAVYDCKWENFDKMNMKTVLLLLQ

NSQKTMTISAGGITMLNFSCLMSVIKSIYSAYTTLRTTMK

>MsexOR50

MISIWTGIRKFGLEYCDLPTMLWNVAFLLRPLTLNIDSRHKKPIPLLFYVLTVVITSSYFYVYLISMSWFVFVRSLETKELIPAMVVLSLGI

SSEIGTLKFFYTFVYIDKVRKIVDEYLECDALVVPFSRFSKNLLTTLRFVKKRAIIYWLVIIGNGFAYWSKPLFMKGRHHLEDNLVIYGLE

PMLESPKYEIAYFLMTAGVCFICYPPANVTILLIVVVGYTEAQMLALSEEILNIWDDANDHYNNLPSTNQNTNELKSKIINKYIKDRLKDI

TKSHARNINLLRQVEFVFRGAIAIGYVFLILGLIAELLGGLENTFLQIPFAFIQVAIDCFTGQRVMDANIVFETAVYDCKWENFDKTNMKT

VLLLLQNSQKTLTLSAGGXTMLNFKCLMSVIKSIYSAYTTLRT

>OfurOR16

MSLWSTIRKFGLGYCDLPTMLWNVSFMLRALTLNIDSRYKKRIPLIFYIIFALVAASYFYIYLISMAWFVFWHSRETGDLVAAMVVASLGI

SSEIGTAKLIYMFLYRNKVRELVDMYLDCDALVKPGSRFANNLTKTLRNVKKRAMIFWIVIMGNGVVYVLKPLLISGRHIMEDLFTPYG

FDPVYESPNYEIVFLLMTAGVLFTCYLPANITAFLIIITGYTEGQMLALSKEMLNLWSDAQQFYLDHRTFDLDTTRPVVTLDSEQITKKKI

VNEYVKKRLHEMIKIHTTNINLLNHVERVYRGAIAIEFGILVLGLIFELLGGLENTYLEVPFALMQVAMDCLTGQRVMDASKAFEDAVY

DCKWENFDVANMKTILLMLQNSQKTMRLSAGGVTTLSFSSLMMVFRSVYSAYTTLRTTMNK

>BmorOR38

MNLSQSVNEQANEYVKMRLERISKIHSPMLPFEDIQDFRELCCIPLAVYAVTGSITASYVYAFLISLLWFLFARCTDPEDFQVAMVVFSLGI

SSEIGSTKFFNSIIYIKELRKLFKDYLLYDATCPAQGRLRLHLLTTLRYVKRRAIIYWLVIIGNGFIFAIKPLLVEGRHLAQDDLVLIGLEPMR

QSPNYEIAYAIMTMGVCFICYPPAHVTMFLIIIVGYTEAQMLALSEELKHLWNDAIEHYEKHSRTEREADAAMKSKILNSFVNFRLVQIIK

SHSTNVNLIGRVENVFRGSLAVGYVFLIVGLIAELLGGLENTYLQVPFALIQVAIDCFIGQRVNDANIDFEKAVYDCKWENFDKRNMKIV

LLLLQNAQKTVSLSAGGIAKLNFSCFMSVIKSIYSAYTTLRTTMK

>BmorOR35

MKLWQSIREFGLEYCDLPTTLQNVASLLRAITLNIDSRHTARIPFICYVMTVVITLSYFYVFLVSMAWFVFVRSAETRDYLAAMVVLSLG

ISSEIGTLKFFYTFIYIKKVQRIVREYLECDHMVVPESRFADNVLKTMRNVKKRAILYWVVVIGNGVVYVTKPLFMSGRHHMEDRYIVY

GLEPMFESPNYEVAYFLMMFGLCFICYPPANVTVFLIVVVGYTEAQMIALGEEMLRIWEDAVAHYNNKYHTVGALTNSSEKNKIINQYV

KFRLTEIIKMHTTNIQLLRQVEFVFRSAIAMGYVFLVLGLIAELLGGLENTYLQIPFALIQVLVDCYTGQKVMDASSLFEQAVYDCKWEN

FDKSNMKTVLLILQNSQKSMRLSVGGITVLGFSCMMSVMKSIYSAYATLRTTMS

>CpinOR55

MENLRKFGLNYCDLPTMLWNVTCLLRVLTLNIDSRYKKRVPAVFYVITAAVSAGYFYVYLVSMVWFVFWRCRQTGDLIAAMIAFSLGI

SSEIGSCKLLYMLIYEKTVRDIVDGYLACDALNTLGSRFSQNVLKVLRFVKKRALFFWIVIVGNGVTYVLKAVVTPGRHMMEDGFLLY

GLEPMLESPNYEIAFFLCCLGVCYTCYLPANITAFCIVLVGYSEATMLALGEELINLWSDAQQCYKDTHNTIDTTNAIKEDLNRKNIIINK

YIKARLKDIVKIHMININFIRQFELVFRGAIALEFLLLITGIIAELLGGLENTYIEMPFALMQVAIDCLTGQRLMDACVIFENSVYDCKWEN

FNVANRRTVLLMLQSSQKTLVLSAGGVATLSFSCFMTIIRSIYSAYTTLQSTMVLT

>HassOR33

PMLLPGRHFMDDVVLLYGLEPMFETPNYQISFVLMGSSCVLICYLCANISAFLIIVTGYVQAQMLALSEELTHLWEDAEENYRGTELEDI

TDDGDHNDKNKDAILNDYVTVHLKDIAKSHAENINLLGQIEGTFRGAIAIEFCLLVIALIAELLGGLQNTYMEVPFALMQVGMDCLIGQ

RVMDAGAVFEDAVYDCKWERFNKKNMKTAMVLLLNAQRPMTISAGGVTTLSYVSFMTIIKSIYSTYTTLRSTMHEP

>CpunOR55

MENLRKFGLNYCDLPTMLWNVTCLLRVLTLNIDSRYKKRVPAVFYVVTAVVSAGYFYVYLVSMAWFVFYRCRQTGDLIAAMIAFSLGI

SSEIGSCKLLYMFIYERTVRDIVDGYLACDALNTLGSRFSQNVLKVLRFVKKRALLFWIVIVGNGAIYVLKAVVTPGRHMMEDGFLLYG

LEPMYESPNYEIAFFLCCLGVCYTCYLPANITAFCIVLVGYSEATMLALGEELINLWSDAQQCYKDTHNTIDTTNAIKEDPNSKNIIVNNY

IKARLKDIVKIHMININLIRQFEIVFRGAIALEFLLLITGIIAELLGGLENTYIEMPFALMQVAIDCLTGQRMMDACVIFENSVYDCKWENF

NVANRRTVLLILQSSQKTIVLSAGGMAKLSFSCFMTVIRSIYSAYTALQSTMVFA

IR:

>LstiIR25a

MLATFTANLAAFLTVERMQTPVSSLEQLARQSRINYTVVEGSTIHQYFINMKFAEDTLYRVWKEITLNATSDQAQYRVWDYPIREQYGH

ILLAINASGPVPDAKTGFEQVNEHTDADFAFIHDSAEIKYEVTRNCNLTEVGEVFAEQPYAIAVQQGSRLQEHL

>LstiIR40a

MYSANLTSLLARPARERPIGTLPALEEAMRERGYELVVERHSSSLAILENGTGVYGRLARLMRRQRIQRVRSVEVGVRLVLTRRHVAILG

GRETLYYDTERFGSHNFHLSEKLYTRYSAIALQIGCPFLETFNNVVMTLFEAGILAKMTTDEYKNLPEQSRRSEPVTESDKPNNEITGDSP

SASQSGTTQGESTKALEPVSLRMLRGAFCLLGIGHLLAAIALGVEIQIHRRSKNFIKIMEPNGGKNMPRMRALKKASKCVRQGVRRVVR

AVCRSIDRALGPGVQ

>LstiIR75.1

MAQQLCSDAGLPLVRAALGSQQLLAALDQYLESRNATDAAILTESEGEVDKTLYELLGRSNVRLWVHAGLTRDSAKALKSMRPEPSFY

VIVGESGFVMDTYRRAVKEKLVRRDYRWNLVLTDYSGDTIDVAQLVLPTMILHVDQVECCRLLGLREECSCPSDLKRKQYILNALLIYIT

ETFSKLERELPVVSTRVDCDNVQGSEMNSTRDRILRQFSEDTEMNNDTVFFWDDERSG

>LstiIR75a

MVNVITDSNDTRKHLDDRLHLHQDSITKMSYSVVKICFEMLNASEELIFTHTWGYRDKNGNWQGIVDNLIKKKADLGTLTIFTQERMQ

VVDYIAMVGSTAVRFVFREPPLSYISNIFTLPFTGAVWLAIVVCVLGCSVFLYITSKWEATMSMHQFQLDGSWADVIILIIGAVLQQGCTL

EPRYAAGRSVTLLLFLALTILYAAYSANIVVLLRAPSSSVRTLPDLLNSPLKLGASDFEYNRYFFKKLKDPIRKSIYDKKIAPKGKKPNYYS
MEEGVEKIRKGLFAFHMELNPGYRLIQETYHEDEKCDLVEIDYINEIDPWLPGQKRSPFKDLFKINFIKIRESGIQACIHHRLHVPKPKCS
GTVSTFSSVGITDMYPAMLATLYGMLLAPAVLLLEIAYHRLTVLRKRKIKTRK
>LstiIR75d
MDTISLIPAFFLSKNIYFLTTFLCWNSEELHKLWRLGQQQGLRVRAMAAGPATPPLPPDDLHREGVVLDLACPYADHIIQAASETRGFNY
RYAWLLLHNSSFDATSLDSVLSGSVILPDADVTFASDDKLLDVYRIKADQPLLATTLGVVRNSTRRDLEQMWGVLKSTVSRRKNLNNV
FLKGATIITQPQNFKGWNDLTVRHIDTFPKLMYPLLMHCAEDLNFRLNLLQVELYGDERNGSFDGLAGMLQRRDIEVGVTTLFMRHDR
LNVMHFCSETLELKGAFIFRQPPQSSVNNVFLLPFSRGVWAASALVFTAAGGLLAALSRPRWLRDADPDLVQLSAAEAFTFAVGTICQQ
GCYVNPHAVSVRMLMFFTLLASLFTFTSYSAKIVAILQTPSDAIQTIDDLTHSPMALGVQESTYKRVYFAESDDPATQRLYRRKLLPQGER
AYLSIVDGIARVRNGLFAFQVEESSGYDVISKTFTEQEKCGLKQIQAFKLPMVAVPILKHSGYRELFAARLRWQRETGIMDRERRVWMA
SKPRCDSDGGGFLSVRLSDVLPAVQVLIYGMLLAAIQLFAEIALHRATERIKRKNKLRGKRE
>LstiIR76b
MATGIELIISSICNATFCQPVYDNPLLEKQASSSIDQYRDLIKEINGKHLKIGTYNNRPISWVERGEDGALIGRGVSFVLVDILQKRFNFTY
EVVVPEKNFEIGGTKPEDSLIGLVNNSLVDMAAAFIPKLTRFHEMVRFSYDLDEGVWVMMLSRPKESAAGSGLLAPFNNAVWYLILVAV
LSYGPCITLLTKLRSKLVPDGEKYIPMSPSFWFVYGAFIKQGTNLAPEANTTRVLFTTWWIFIILLSAFYTANLTAFLTLSKFTLDIESPQDL
FKKNTRWVSAEGGAVQYVVSSPNEDIYYLSRMIATGRAEFRSMNSLYEFLPLVSGGAVLVEERIGIDELMYGDYQQKAREGVAEAERCT
YVVAPNLFMSKLRGFAYPKNSQLAPLFDTVLTYVLQAGIVDYLEHRDLPSTKICPLDLQSKDRQLLNSDLYMTYMIMVTGLSAAVAVFI

GEIMIKRYVIKDSKPKKPKRKKTKYEKNRHI

>DmelIR8a

MELPLLVLLLALRFAGSEVLKITFWIEPVQRAEFDTDIAMVLKELDALRLDVKVDDTTLTLTRSEDGLDMQRFCEILSTVGASAVIDLTY

SHWEEGYNLVRSLGIGYVRLERIMRPFLDMFGDFMRQKRANNVAMVFMNARDAVEAMQQMLVGYPFRTLIMDASQTDPGQHFLERI

RSLRPAPTYIALFARAAAMNGIFEKVQKAGLFQRPLEWHFVFLDTRDRVFKYRRQAELCTRFTLNPRAICRSMPMPDLYCGSGFTMQR

AMLLNVLRSLINAAQVSPGYPMAIYQDCNATASSSEVSDPLEKDDYNWLDMVHWSNFLAYAPPLPHIQDQFQSPVPGLTFAVNISAGYY

SSEHEAKTDLAAWSSVGEMRLLNETISPARRFFRIGTAESIPWSYLRREEGTGELIRDRSGLPIWEGYCIDFIIRLSQKLNFEFEIVAPEVGH

MGELNELGEWDGVVGDLVRGETDFAIAALKMYSEREEVIDFLPPYYEQTGISIAIRKPVRRTSLFKFMTVLRLEVWLSIVAALVGTAIMI

WFMDKYSPYSSRNNRQAYPYACREFTLRESFWFALTSFTPQGGGEAPKAISGRMLVAAYWLFVVLMLATFTANLAAFLTVERMQTPVQ

SLEQLARQSRINYTVVKDSDTHQYFVNMKFAEDTLYRMWKELALNASKDFKKFRIWDYPIKEQYGHILLAINSSQPVADAKEGFANVD

AHENADYAFIHDSAEIKYEITRNCNLTEVGEVFAEQPYAVAVQQGSHLGDELSYAILELQKDRFFEELKAKYWNQSNLPNCPLSEDQEGI

TLESLGGVFIATLFGLVLAMMILGMEVLYYKKKQNALEITQVRPVNDSSGSGGNSSTAPPTATSTTKQAWHIPVLEAEEKPAKVSPPPSFE

TATFRGKKLPARITLGDGKFKPRHGLYARRNLGASDSHSGYME

>DmelIR25a

MGSRLDWGVADVALWAIADQIDYHQVFINEVDNEPAAKAVEVVLTYLKKNIRYGLSVQLDSIEANKSDAKVLLEAICNKYATSIEKKQT

PHLILDTTKSGIASETVKSFTQALGLPTISASYGQQGDLRQWRDLDEAKQKYLLQVMPPADIIPEAIRSIVIHMNITNAAILYDDSFVMDH

KYKSLLQNIQTRHVITAIAKDGKREREEQIEKLRNLDINNFFILGTLQSIRMVLESVKPAYFERNFAWHAITQNEGEISSQRDNATIMFMKP

MAYTQYRDRLGLLRTTYNLNEEPQLSSAFYFDLALRSFLTIKEMLQSGAWPKDMEYLNCDDFQGGNTPQRNLDLRDYFTKITEPTSYG

TFDLVTQSTQPFNGHSFMKFEMDINVLQIRGGSSVNSKSIGKWISGLNSELIVKDEEQMKNLTADTVYRIFTVVQAPFIMRDETAPKGYK

GYCIDLINEIAAIVHFDYTIQEVEDGKFGNMDENGQWNGIVKKLMDKQADIGLGSMSVMAEREIVIDFTVPYYDLVGITIMMQRPSSPS

SLFKFLTVLETNVWLCILAAYFFTSFLMWIFDRWSPYSYQNNREKYKDDEEKREFNLKECLWFCMTSLTPQGGGEAPKNLSGRLVAAT

WWLFGFIIIASYTANLAAFLTVSRLDTPVESLDDLAKQYKILYAPLNGSSAMTYFERMSNIEQMFYEIWKDLSLNDSLTAVERSKLAVWD

YPVSDKYTKMWQAMQEAKLPATLDEAVARVRNSTAATGFAFLGDATDIRYLQLTNCDLQVVGEEFSRKPYAIAVQQGSHLKDQFNNAI

LTLLNKRQLEKLKEKWWKNDEALAKCDKPEDQSDGISIQNIGGVFIVIFVGIGMACITLVFEYWWYRYRKNPRIIDVAEANAERSNAAD

HPGKLVDGVILGHSGEKFEKSKAALRPRFNQYPATFKPRF

>DmelIGluR1D

MRSSGVLVLPLLLLQLILNCRKAQSLPDIIKIGGLFHPADDHQELAFRQAVDRINADRSILPRSKLVAQIERISPFDSFHAGKRVCGLLNIGV

AAIFGPQSSHTASHVQSICDNMEIPHLENRWDYRLRRESCLVNLYPHPNTLSKAYVDIVRHWGWKTFTIIYENNDGIVRLQELLKAHGM

TPFPITVRQLSDSGDYRPLLKQIKNSAEAHIVLDCSTERIHEVLKQAQQIGMMSDYHSYLVTSLDLHTVNLDEFRYGGTNITGFRLINEKI

VSDVVRQWSIDEKGLLRSANLTTVRSETALMYDAVHLFAKALHDLDTSQQIDIHPISCDGQSTWQHGFSLINYMKIVEMKGLTNVIKFD

HQGFRTDFMLDIVELTPAGIRKIGTWNSTLPDGINFTRTFSQKQQEIEANLKNKTLVVTTILSNPYCMRKESAIPLSGNDQFEGYAVDLIHE

ISKSLGFNYKIQLVPDGSYGSLNKLTGEWNGMIRELLEQRADLAIADLTITFEREQAVDFTTPFMNLGVSILYRKPIKQPPNLFSFLSPLSL

DVWIYMATAYLGVSVLLFILAKFTPYEWPAYTDAHGEKVESQFTLLNCMWFAIGSLMQQGCDFLPKALSTRMVAGIWWFFTLIMISSY

TANLAAFLTVERMDSPIESAEDLAKQTRIKYGALKGGSTAAFFRDSKISTYQRMWSFMESARPSVFTASNGEGVERVAKGKGSYAFLME

STSIEYVTERNCELTQVGGMLDTKSYGIATPPNSPYRTAINSVILKLQEEGKLHILKTKWWKEKRGGGKCRVETSKSSSAANELGLANV
GGVFVVLMGGMGVACVIAVCEFVWKSRKVAVEERLSAILNE

>BmorIR25a

MIGEAVKHAEDLKQLALDDSIVSLNRENEDEARGKLCSELSKGVSALIDLSWSPWEEAEHLASEAGVPIIRTALGPQPLVAAIDRYLESR
NATDAAILLESEIDVDKVLYELLGRSNTRIWVHAGLTRDSARALKTMRPDPSFYVLVGGSGFVMETYKRAVKEKLVRRVYRWNLVFTD
YLDLSALDLSSVVQPTMILQAHPEDCCRIIASKDCTCSDSFEKTQATLNSLIAYLVDAFGRIDGTFMVKARVDCGDVHSDMSDTKEALH
RIFVEDIDNNETVFYWDQERSGLFLRHRYVLSSYKPDSGLEAVAVWSANQEYRLLPGVTLEPLKHFFRVGTSPAVPWTLPKLDPETGDPL
YNEDGQPIYEGYCVDLIQKLSEAMNFDYEIVSPRSGGFGRRLPNGSWDGVVGDLTTGETDIAVAALTMTAEREEVIDFVAPYFEQTGILI
AIRKPIRKTSLFKFMTVLRTEVWLSIVAALVLTGFMIWLLEKYSPYSAKNNPGAYPYPCRDFTLKESFWFALTSFTPQGGGEAPKALSGR
TLVAAYWLFVVLMLATFTANLAAFLTVERMQTPVSSLEQLARQSRINYTVVEGSSTHQYFINMKFAEDTLYRVWKEITLNATSDQAQYR
VWDYPIREQYGHILLAINASGPVADAETGFKQVNDHTDADFAFIHDSAEIKYEVTRNCNLTEVGELFAEQPYAIAVQQGSRLQEDISRAL
LELQKERFLEQLASKYWNETLRQSCSDADESEGITLESLGGVFIATLFGLGLAMITLAWEVFYYKRKEKNKVQSTKENVERPPIKSAKL
GGKMAVGVARLRKRATKIGKKKNVTIGDSFKPSVSYISVYPKGDYRP

>SlitIR25a

MNGEPEITSAFYFDLSLRTFLTIKSLLDSGKWPNDMKYITCDDYDGKNTPNRTLDLKTAFQEIKETPTYAPFYIPQDDPMNGRSYMEFST
DLLAITVKDGASISSHSLGSWKAGLSSNLTLTDPNNMSNYSAQLVYRIVTVEQKPFIIRDDQAPKGFKGYCIDLIEEIRAIVKFDYEISLAP
DGNFGTMDENGNWDGIIKELVDKKADIGLSSLSVMAERENVVDFTVPYYDLVGITIMMKLPRTPTSLFKFLTVLENDVWLSILAAYFFT

SFLMWVFDKWSPYSYQNNREKYKEDEEKREFTLKECLWFCMTSLTPQGGGEAPKNLSGRLLAATWWLFGFIIIASYTANLAAFLTVSR

LDTPIESLDDLSKQYNIPSATVSMDLRLMTCFQRGGYLGGSFLKFGGSWAKRPFKGGGTAQNLRYGIIPVRDKYIKFWRAMEEAVLPLP

FLGKLYRGVRDSKSFSEGFAWLGDATDVKYHVMTSCDLQSVGDEFSRKPYAIAVQQVSPLKDHFNNAILQLLNKRKLEKLKEIWWNN

NPESMKCEKQDDQSDGISIQNIGGVFIVIFMGIGLACVTLGVEYWWYKWRKRPAVGDVTQVEPAKLTRNNVDKQGEGFNFRGRNLGL

NFKPKF

>DmelNmdar1

MAMAEFVFCRPLFGLAIVLLVAPIDAAQRHTASDNPSTYNIGGVLSNSDSEEHFSTTIKHLNFDQQYVPRKVTYYDKTIRMDKNPIKTVF

NVCDKLIENRVYAVVVSHEQTSGDLSPAAVSYTSGFYSIPVIGISSRDAAFSDKNIHVSFLRTVPPYYHQADVWLEMLSHFAYTKVIIIHSS

DTDGRAILGRFQTTSQTYYDDVDVRATVELIVEFEPKLESFTEHLIDMKTAQSRVYLMYASTEDAQVIFRDAGEYNMTGEGHVWIVTE

QALFSNNTPDGVLGLQLEHAHSDKGHIRDSVYVLASAIKEMISNETIAEAPKDCGDSAVNWESGKRLFQYLKSRNITGETGQVAFDDN

GDRIYAGYDVINIREQQKKHVVGKFSYDSMRAKMRMRINDSEIIWPGKQRRKPEGIMIPTHLRLLTIEEKPFVYVRRMGDDEFRCEPDE

RPCPLFNNSDATANEFCCRGYCIDLLIELSKRINFTYDLALSPDGQFGHYILRNNTGAMTLRKEWTGLIGELVNERADMIVAPLTINPERA

EYIEFSKPFKYQGITILEKKPSRSSTLVSFLQPFSNTLWILVMVSVHVVALVLYLLDRFSPFGRFKLSHSDSNEEKALNLSSAVWFAWGVLL

NSGIGEGTPRSFSARVLGMVWAGFAMIIVASYTANLAAFLVLERPKTKLSGINDARLRNTMENLTCATVKGSSVDMYFRRQVELSNMY

RTMEANNYATAEQAIQDVKKGKLMAFIWDSSRLEYEASKDCELVTAGELFGRSGYGIGLQKGSPWTDAVTLAILEFHESGFMEKLDKQ

WIFHGHVQQNCELFEKTPNTLGLKNMAGVFILVGVGIAGGVGLIIIEVIYKKHQVKKQKRLDIARHAADKWRGTIEKRKTIRASLAMQ

RQYNVGLNSTHAPGTISLAVDKRRYPRLGQRLGPERAWPGDAADVLRIRRPYELGNPGQSPKVMAANQPGMPMPMLGKTRPQQSVLP

PRYSPGYTSDVSHLVV

>DmelIR75d

MKVQVAHWLPLIFFLLVSGTPRVAGSWRSEYSRQDPDPKTRWGNQLPDMLVAYYRHHGVHSLMLVVCHTDIADFRLWKLWQHFNLN

NFYVQVSTESSLRDLQHVDALDEHKDAPPPKSFHANNSTHWETSFLLPALPYKMGILLLEFSSECALNLLRWSAASEHNYFTTNRFWLL

LTEDPGDIDLLEDPEIFIPPDSELRVLHYENVGNFSCSLIDLYKVAAWKPLKRTLVGHNIRNSRHVIHALQHFGSAITYRQDLEGIVFNSAI

VIAFPDLFTNIEDLSLRHIDTISKVNHRLMLELANRLNMSYNTYQTVNYGWRQPNGSFDGLMGRFQRYELDLAQLAIFMRLDRIALVDF

VAETYRVRAGIMFRQPPLSAVANIFAMPFENDVWVSILMLLIITTVVLVLELFFSPHNHDMSYMDTLNFVWGAMCQQGFYVEVRNRSA

RIIVFTTFVAALFLFTSFSANIVALLQSPSDAIQSLSDLGQSPLEIGVQDTQYNKIYFTESTDPVTKNLYHKKIASKGENIYMRPLLGMEKM

RTGLFAYQVELQAGYQIVSDTFSEPEKCGLMELEPFQLPMLAIPTRKNFPYKELIRRQLRWQREVSLVNREERKWIPQKPKCEGGVGGF

VSIGITECRYALGIFGCGAAVSFVLFLFEFIFRHFKQVYRIIKGYREVQR

>DmelIR64a

MHWWLLVFLPLSCQGLPEHELLELELDYGLAEPQRTSLLQSSLILQFSQDYKHIPRITYFTCQKPHLQTPNQIPNAAEHRDAFAAKNFQL

IKSLYESELFVRIVLLDVLAQSPSSGRPNRPGSGPTGGFSQTPSQAQSNSEWLEGVLRMEALRQIAVVDLACGAVSRRFLELASAKMLYS

EKFHWLLIEDFAWHGRTQTAEGSGKRDDGEMEEEEPPGQQIQATDDEDLPSIESFLGGMNLYMNTELTLAKRMSEAAHYTLFDVWNPG

LNYGGHVNLTEIGSFTPTEGIQLHTWFRTTSTVRRRMDMQHARVRCMVVVTNKNMTGTLMYYLTHTVSGHIDTMNRFNFNLLMAVR

DMFNWTFVLSRTTSWGYVKNGRFDGMIGALIRNETDIGGAPIFYWLERHKWIDVAGRSWLSRPCFIFRHPRSTQKDRIVFLQPFTNDV

WILIVGCGVLTVFILWFLTTIEWKLVPHDGSALIKPKGGAPPRHHYQQQQQQEQVEAPVRPITAVSVVVSKEKVEEKQEEYEDSTPIDAG

TLWQRCYQKLNKYIKDRKAKQKKAPERVGLFLESVLFFVGIICQQGLGFSTSFVSGRCIVITSLLFSFCIYQFYSASIVGTLLMEKPKTIKT
LSDLVHSSLKVGMEDILYNRDYFLHTKDPVSMELYAKKITSVPTTKENEADEDEPVDPNPASTDPAKSYRDIVHSHETGAHAKDNAASN
WLDPETGLLRVKHERFAFHVDVAAAYKIIAETFSEQDICDLTEVSMFPPQKTVSIMQKNSPMRKVISYGLRRVTETGILTYHFNVWHSRK
PPCVKKIETSDLHVDMDTVSSALLILLFSYAITLMILGTEILYSKWHNRIQLKWVGAT

>BmorIR75a

MSTKILFILFYCALVTCYNKINIIGSFANHNWKPTSILAPNLCWDDFEVKELTKMLNKIGINVAKSLQRNRTEYFLQHCLIVADFDCGDIEE
FLMKANGEGYFKSPYRWLLINSNEHNTDVLRKLDVLVDSDVVIAKRINEDEVLFEEVYKISESSNMTYLNNRAMWSFKYNLKNLTASS
VALKENSTGIDTNDPNSLKEKIQKTGEIEDYTKNKVLSSRRVNLKRHTLTMVNVITDSNETRKHMDDRMNLHQDSITKMSYAIVRICFE
MLNATEKLMFTHTWGYRDKQGRWQGVVDHLIKKEADLGTLTIFTQERMDRVDYIAMVGSTAVRFVFREPPLAYISNIFALPFNTNVWL
AILICVLGCSGFLYVTSKWEASMGMHPLQMDGSWADVLILIIGAVLQQGCTLEPRYAAGRCVTLILFVALTILYAAYSANIVVLLRAPSSS
VRSLPDLLNSPLKLGASDFEYNRYFFKKLNDPIRKAIYDKKIAPKGKKPNFYSMEDGVEKIRKGLFAFHMELNPGYRLIQETYREDEKC
DLVEIDYINEIDPWVPGQKRSPFKDLFKINFLKIRESGIQAAIHHRLHVPKPRCSGTVSTFSSVGITDMYPAMLATLYGMLLAPAVLLVEIA
HMKLMLVRMKKADTENAYVTPFVN

>SlitIR75d

MELISFILSYFITKDLSMMTAFICWPSEQALELQRSARVAGVRLTVVSELRHSAPMTTSGYFREAMLLDLNCPDTHFVLEKASRSRVLNK
RHSWLLLHNSSAEPALVEETLYAYEILPDADVVWSSPNSLVDVYKTKPNQPLLQVQLGLSRNSSHQELLSLWGALPTAVTRRRDLRNVS
LKGISVVTEPDNFKGWADLRNRQIDTFPKFTYPLMMLLAQDLHFRFDLRQVDFYGVSHNGSFDGLVGHLQRREAEVGLASLFMRHDR

MQVADFFSETCVLACAFIFRQPSRSAVSNVFLAPFSAGVWGASACVAASAALLLVALRRLRQHTRASTDLQLFTLLEAVTFALGSMCQQ

GFHRTPPVTSVRLVMFSTLLTSLFVFTAYSAKIVAILQTPSTALQTIDDLVRSPMTIGVQDTTYKTVYFLESPEKSTQQLYRHKILPQGERA

YHSVVDGIARVRTGFFAFQVEKSSGYDIIKQTFTEREKCSLSEIEAFKPPLVAVPMKKHSGYRELFASRLRWQREVGLMDRARHVWLVS

>SlitIR75q.2

MKKITFIFIAFFFSSINGKTEQANMIVDIIQAANRPSSVIGKLCWTPTKIIHLSSALVKENIQFSANADLTNDYMQFYDEEQQIVFLADLNC

PDIEDYFQMNSTRTFFRAPFRWILFGDTGSVNEDNIVPEAIANVDVLIDSEVLVVRSIDDAYEMHFIYRISPNNTWNTEYYGTWDSENRF

QKSNRFVEPTSLRRLDINEYEISICYVLTNNNSINHLSDGLDDHVDYEFTKVNFPTTNHLLDFLNAGRKYIFAETWGYKVNGTWNGMT

GYLVRGEVEIGGSPMFFTFERVSIVDYISSPTPTRSKFVFQQPKLSYENNLFLLPFNTTVWYCTVGLVFVIYLVLLLVAKWEWKKTKHTIE

TREKDAGVLRANVVDVIILIFGAACQQGSPSELNGRWLGRIVMLVLFLALMFLYTSYSANIVALLQSSSSHIKTLDDLLHSRIKFGVHDT

VFNRYYFSTATEPVRKAIYEKKVAPPGTTPRFMTMDEGVLQMRKGLFAFHMETGVGYKFVGKYFNEGEKCGLREIQYLQVIDPWLAVR

KDTPFREMFKIGTKRIQEHGLQYRENRLMYEKRPKCSGGGSNFVSVSMVDCYPAILILSYGTIVALFFLGLEILVHKREKVLLKLKCLKR

KLRLMTEVILRRNALDVEPLVVEL

>SlitIR75p

MTGISIVLFFLVTQCSVLIQSKDMENINFIKLFILNDQKPTHLIYGGLCWKKELINKLVVEMSNIGVRTSASFKPRSKYQDHAIMYLTDLD

CAQSRTVLSYASSKELFQFTYRWLILVTSPQLQQSKISLLENGPVLVDSDVVLAERVGNMFKMTELHRPGPNGSMISTPRGYYNGSVVD

VRAHRELYRRRRNMRGHAITMSNVIQDSNTTRLHLPREDRLKLQYDSITKACWSAAKIGFEMINATPRYIFSYRYGYKVNGQWSGMIA

DLYANKADMGTNCVIFRDRFDVVTYTDLVAPMRMLFIFRQPPLAYVANVFYLPFSTRVWVTIAVCTAIATVTLFLASKVEIVITKTTTQQ

QLDGGICDVLLLTMSAVTQQGCYIEPRRAPGRMMVFVLFTALMALYAAYSANIVVLLQAPSDSIRSLPQLANAKITLAANDVDYNHFVF
NQSREPLYISIRDRVFPENGKAKLYSLADGVERIRQGLFALHSVAEPVYRQIEATFLESEKCDIATVDYLVTFDSFTPVRKGSPYLELIRVV
HKQIRESGIQSAIRRRYLVSKPHCTTKMSSFSSVGLMDMRPVLILMLYGVAVSVIIVFGEIIVHKLINRYYKQKSKVQMVKTIHY
>SlitIR75q.1
MKYLTFVLNIICLDYCVTFNTNTELQIIVDVAKSYDKPTSVIAKMCWETSKRSKYAPLEAKLAKMLANLDRPMNIRYLRQNETIDNDNY
PNNHLLFILNRTCDDANAFLRWASANHKFRKSHRWLILGKSLIIKDETFNVSPEFDDIRISVDSEVIIIGKINNSEEVSLHTFYKLKPHTKWI
IEDYGTWSFDTGFTKSTTRIESNVIRRKDFMGETLITSVAISDNRTKTDLLGLGNIFIDTPAKSSFRTIVPLFDFLNATKVVKSLILGVLINGS
GMEGLVILSGRSRLVWNCNVHNERTYDNFRIFDPSYTHHTEVCLQTTALVVPEQFIPSAIFHWCLAVHWCIHCYINCHIVRQHDMGFKEI
QRLNKQKIDQTCLPPTWSDITIFVLSAISQQGSSNELKGTLGRLVMFLVFLAFVFLYTSYSANIVVLLQSTSNQIRTLSDLLHSRLELGLEH
APFNKFYFSSAYTADDPIKKALVDTKIAPKGVLTNVMNIEQGVRIMQKKPFAFNMNTGXRVQNCFSNLKNHGKVSGLQEIEYIPNSNPW
LCSRRLSPYGELFKVGYIRIQEHGLSDRENRLIYAKKPACTVMGGSFGSVNMVDLHPVCLVLLYGMILAFLLLGVEILVHRKQMKMRN
QARVECNVKLCSKVASCEIRFWFCIAYKMCSNDAFIAKILLIFFTVNRKLLMASPPLTSVTGDPMAFNVLIDDFH
>SlitIR1
MLAQKLTKENVRVSVRRLNGDNVDVVRVAHQTTVPVGVLVDGHCDQTQTLMNQASFNKLFDAVHSWLILTDFEDDNCTEYVMQTFQ
WLNLSVNADVAVVANRGDSFAIIDVYNFGKIQGNHLETALLGTWQPDQGLEIILKGYKYYNRWDFHNLTLRAISVIVDQPKVFYPEMLS
EMTYTSGVAAMTKITTQMLNTIKERHNFRFNYSIASRWIGSPERNSTMAVTNTLFWEEQDLSSTCARIFPKWLNWVDIYHPPTTNLQTK
FYYSLIPETGCRGQYEGTGFLTLGCSHGGLGGCVLLSPGIPRTRGSWQPRLRMEKQTQARDCMPSFRVFAAGLSTRLGRRRSGCWSQTL

SSQGRRTNPASDRTDEHAAVQLTTPAVWCPGCWTLRAPFPSANLEGLINSDFELVLEDIGYTRGWLDNPGFFYYSGFKNVKEDELRDKK

VTKAKRTVSVLQNVNKGVELLRTGKYAFHTEPYTASQVISKTYEDKELCNLGALQMMLPAHVYIMAQKKSPYKEFFDWSLLRLLERG

HVKAIRARFAGTMSACSGAQPRALALGQAAPAFLMLASFAVLSCFILVLEVLWKRVQLKNRGQ

>DmelIR76b

MATGIELLVAAALCVACPPLNDSPPTNLIQMGENGTLSPVTELPMDVDASEAGFDADAPVETLETINRKKPKLREMLDWIGGKHLRIAT

LEDFPLSYTEVLENGTRVGHGVSFQIIDFLKKKFNFTYEVVVPQDNIIGSPSDFDRSLIEMVNSSTVDLAAAFIPSLSDQRSFVYYSTTTLD

EGEWIMVMQRPRESASGSGLLAPFEFWVWILILVSLLAVGPIIYALIILRNRLTGDGQQTPYSLGHCAWFVYGALMKQGSTLSPIADSTR

LLFATWWIFITILTSFYTANLTAFLTLSKFTLPYNTVNDILTKNKHFVSMRGGGVEYAIRTTNESLSMLNRMIQNNYAVFSDETNDTYNLQ

NYVEKNGYVFVRDRPAINIMLYRDYLYRKTVSFSDEKVHCPFAMAKEPFLKKKRTFAYPIGSNLSQLFDPELLHLVESGIVKHLSKRNLP

SAEICPQDLGGTERQLRNGDLMMTYYIMLAGFATALAVFSTELMFRYVNSRQEANKWARHGIGRTPNGQSVAPSRWLRGWRRLNSGH

GQLLGASTHGQNVTPPPPYQSIFNGGSHGDPLNRWRRPLANGNALGNGVLLGGDSEGGVRRLINGRDYMVFRNPNGQSQLVPVRSPS

AALFQYSYTE

>DmelIR93a

MNPGEMRPSACLLLLAGLQLSILVPTEANDFSSFLSANASLAVVVDHEYMTVHGENILAHFEKILSDVIRENLRNGGINVKYFSWNAVR

LKKDFLAAITVTDCENTWNFYKNTQETSILLIAITDSDCPRLPLNRALMVPIVENGDEFPQLILDAKVQQILNWKTAVVFVDQTILEENA

LLVKSIVHESITNHITPISLILYEINDSLRGQQKRVALRQALSQFAPKKHEEMRQQFLVISAFHEDIIEIAETLNMFHVGNQWMIFVLDMVA

RDFDAGTVTINLDEGANIAFALNETDPNCQDSLNCTISEISLALVNAISKITVEEESIYGEISDEEWEAIRFTKQEKQAEILEYMKEFLKTN

AKCSSCARWRVETAITWGKSQENRKFRSTPQRDAKNRNFEFINIGYWTPVLGFVCQELAFPHIEHHFRNITMDILTVHNPPWQILTKNSN
GVIVEHKGIVMEIVKELSRALNFSYYLHEASAWKEEDSLSTSAGGNESDELVGSMTFRIPYRVVEMVQGNQFFIAAVAATVEDPDQKPF
NYTQPISVQKYSFITRKPDEVSRIYLFTAPFTVETWFCLMGIILLTAPTLYAINRLAPLKEMRIVGLSTVKSCFWYIFGALLQQGGMYLPTA
DSGRLVVGFWWIVVIVLVTTYCGNLVAFLTFPKFQPGVDYLNQLEDHKDIVQYGLRNGTFFERYVQSTTREDFKHYLERAKIYGSAQEE
DIEAVKRGERINIDWRINLQLIVQRHFEREKECHFALGRESFVDEQIAMIVPAQSAYLHLVNRHIKSMFRMGFIERWHQMNLPSAGKCNG
KSAQRQVTNHKVNMDDMQGCFLVLLLGFTLALLIVCGEFWYRRFRASRKRRQFTN

>BmorIR93a

MKIWVLGVLCLAISVQGEDFPSLITANASIAVILDRQYLGDKYQTVLDELKDYIKELARVELKHGGVLVHYYSWTTISLNKGFLAVFSIA
SCEDTWELFSRTEEEDLLLFALTEVDCPRLPQRSAITVTYSEPGEELPQLLLDLRSSNAISWKSAVILHDDTLGRDMVSRVVQSLTSQIDEE
SARPVSVTVFKMKHEMNEYLRRKEMHRVLSKLPVKYIGENFIAIVTSDVMTTMAEIARELLMSHTMAQWLYVISDTNAHASNLSGFIN
TLNEGENVAFIYNITENGPDCKNGLMCYSQEMMSAFISALDAAIQAEFDVAAQVSDEEWEAIRPSKVQRRDILLKHMQQYILAKSVCG
NCTLWRALAADTWGVTYRQNDVPEQINEHANGSTGVIEHLELMNVGIWRPIDAMTFADLLFPHVHHGFRGKELPIITYHNPPWTFLQA
NESGAIVKYSGLMFDIVNQLAKNKNFTIKIMLPSHVKHEFSNETDMMHSQSARLTIAAIAKGHAALAAAPFTVLPGPNQGINYTIPVSTQ
PHTFIVARPRELSRALLFLLPFTTDTWLCLGFAVILMGPMLYIVHRLSPYYEAMEITREGGLATIHNCLWYIYGALLQQGGMYLPRADSG
RLVIGTWWLVVLVIVTTYSGNLVAFLTFPKLEAPVTTISELLKNSDAYTWSVTKGSYLEMELKNSEEPKYKRLIKEAELLKETGGIEGTIH
AARGTLDRVRGQRHLIFDWRLRLTYLMSADHIATETCDFALAVEDFMEEQVAMIVPAGSPYLPVINKEINRMHKAGLISKWLSAYLPKP
NRCLKISTVTQEVSNHTVNLSDMQGSFFVLFLGFFSASTVLILEWFYNRRKRKSEEIVIKPYVE

>BmorIR21a

MDRRSLYGLLFIFYIISSQEIISYHSESLLKNASRNLLWNKKITSIIKEHNDFAYHYESDLHFGNRIKNVKSKRAVDPVFHGHPKTREELWY

ERFLNRSSVFDQTPSLIKLIQNITLTYLNECTPVILYDSQIKLKESYLFQNLLRNFPVSFVHGYINEHSQLQEPKLLQPVRECLHFIIFLSDVK

VSAKVLGKQSESKVVVVARSSQWAVHEFLSSSFSRGFINLVVIGQSFKEDDDSTIESPYILYTHKLYTDGLGASKPVVLNSWSHGKFSRN

VNLFPPKMTGGYAGHRVVVAAANQPPFVFRRIKSDLDGGNPRVVWDGIEIRLLHLLAEKNNFSIEIVEPQELHLGSGDAVAKEIAKGRA

DIGVAGMYLTIDRTREMDVTFAHSQDCAVFITLMSTALPRYQAILGPFHWHVWVALTLTYLFGMFPLAFSDKHTLRHLINNSGEIENMF

WYVFGTFTNCFTFLGRNSWSKTDKITTRLLIGWYWIFTIIITSCYTGSIIAFVTLPMFPETVDTIHQLLAGFYRVGTLDRGGWERWFLNSS

DPNTNKLLKKLELVPNVEAGIMNTTKAFFWPYAFLGSKAELEYIVQSNFTKTTSKRAVLHISNECFVPFGVTIGFPNNSLYTAKLNNDLR

RMVQSGIVDKIVDEVRWEMQRSSNGKLLSAVGGSLKVSAAEEKGLTLEDTQGMFLLLAAGFLIAATALISEWIGGFSKLCRFRKKKNTL

VNSSTKEDSINMPPTDSKDFKTETESVLHFCSRSTSPGSNESLDGQIINVTEESIEIHKQFTSEWDSRRSSSVDLEKEVKEIFERDLRRRGA

ALNNCQDVVLETRQSTASNNAFGDAVK

>SlitIR76b

MAGIELIISSICNATFCEVPYNETYQAPDSLAEKDTNFMSLMKEVNGKNIKVTTYNNTPLSSTELENGTVVGKGVAFTILNILRKKFNFTY

EVVLPTKNFELGAKISDDSIIGLLNSSKVDMAVAFIPTLLPYREWVSFSIDLDEGVWVMMLKRPKESAAGSGLLAPFNDLVWYLVLAAV

LTFGPCITFFTRVRSKLITDDEGVLPLKPSFWFVYSAFLKQGTNLSPEAHTTRVLFVTWWLFMILLSAFYTANLTAFLTLSKFTLAIETPKD

LYQKNNRWVASAGSSVEHVVKTEGEDLYFLNAMISSGKARFLSVAGDKDFLDFVKKGAVLVKEQTVVDHLMYNDYISKKDVEESEKC

TYVVAPSAFMKKQRAFAYPVGSKLKGLFDPVLTQIFQAGILDFLKRSDLPSTKICPLDLQSKDRKLRNSDLIMRYMVMVAGSATAVAVFG

AEVFIKRYVSGKLNKNKKSKRKKSKTGKSLKSHDDSRPPPYDSLFGKNPKFNVETTRMKMINGREYYVFETSNGDKKLIPARAPSSFLY

RSDK

>DmelIR40a

MACNELHNGYRAKFLTIVYWIAATYVLADVYSAQLTSQFARPAREPPINTLQRLQAAMIHDGYRLYVEKESSSLEMLENGTELFRQLYA

LMRQQVINDPQGFFIDSVEAGIKLIAEGGEDKAVLGGRETLFFNVQQYGSNNFQLSQKLYTRYSAVAVQIGCPFLGSLNNVLMQLFESGI

LDKMTAAEYAKQYQEVEATRIYKGSVQAKNSEAYSRTESYDSTVISPLNLRMLQGAFIALGVGSLAAGVILLLEIVFIKLDQARLWMLC

SRLQWIRYDRKV

>DmelIR67c

MFCWLIFLNIILLSDRSESWSAREVIHQFNHDQQLQLNIYLDCNDVELQIGQEVSNLFVNSTADKMKILGRFSSHSLIIACFKDSTRNRTL

NGVKELLWGLQYLPILFVVDSNMDFYFQQALRHGFIHVLALNFMNGSLYTYKPYPKVEVHQIKDMQKFYKLTKLRNLQGQAVRTTVE

TMTPRCFRYRNRHGQLVYAGYMYRMVKEFISTYNGTEEHVFGNVDTVPYKEGLAALKNGEIDMMPRIIHALEWYYFYRSHILYNIKTY

IMVPWAEPLPKSLYFIQPFRGTVWITIMVSFVYASIVIWWIRYRQQGNSSLTQSFMDVLQLLFQLPLSKIWHFNMGTHQVVSFIVLFVFGF

MLTNLYTAQLSSYLTTGLFKSQINTFDDLFREKRTLLVESFDAEVLHNMTKEKIIQKEFESIILITSIEEVFKHRKSLNTSYAYEAYEDRIAFE

LSQQRYLRVPIFKILKEVYDQRPVFVALRHGLPYVELFNNYLRRIFESGIWIKLQEDSFLEGIASGEISFRKSKSREIKIFDKDFYFFAYILLG

MGWCVSTIALFLELWSFKYSVTNVLHEG

>BmorIR40a

MKPFFLMLFLLLTDADCFFDIKEIISDKMTKLPKDFNVAIKDIAESLPSKEMTVVRGNSTNIRSQDVFELLRLLCQHNIQVVNLDIAAMEN

KEMYYGYLKKALDVSDERTNLILCEPYECENLLLELRENNLIHRTILYIFFWPYGSVSDRFLNTMVEAMRVAVITNPRESVFRIYYNQAT
PNRLNHLSLVNWWAFRLYKSPLLPSADKVYKNFRGRVFDVPVLHAPPWHFVKYNNDSSINVTGGRDDKLLKLIANKLNFRYRYYDPP
DRSQGSGIIGNGTFKGTLGLIWKRQADFFLGDVTMTWERLQAVEFSFLTLADSGAFLTHAPAKLSETLAIIRPFRWEVWPLVCATLFITGP
ALWIVIAAPSLWQRKKRDQMGLLNNCCWFTVTLFLRQSSTKEPSSTHKARLVTVLISLGATYVIGDMYSANLTSLLARPAKEPPIGTLPA
LEEAMREHGYELVVESHSSSLSILENGTGVYGRLAKLMKRQRVQRVHNVEAGVRLVLNRRRVAVLGGRETLYYDTERFGSHNFHLSEK
LYTRYSAIAFQIGSPYLETINNVVMTLFEAGILGKMTTDEYKNLPEQSRRSEPVTESENLSTEKTGETAAVTQIQNETSKGLEPVSLTMLR
GAFCLLGIGHLLAGVTLLIEIQLYRRARKRALPPQTRNPTNTFKAKAKKCILRGWRRIKAAAILAIDRALAPDRGID

>SlitIR40a

MRRQRVQRVRNVEVGVRLVLSHKRVAVLGGRETLYYDTERFGSHNFHLSEKLYTRYSAIALQIGCPYLETFNNVVMTLFEAGIVAKMTT
DEYKNLPEHARRSDPVTESDKQGGEVMGESAATSSQTPQGESTKGLQPVSLRMLRGAFCLLGIGHLLAAISLAVEIQLHRRSKRRRKPE
HNEHRKAQKLLVLGKSVMLFKRGCKKVCTSVFTSIDKALGSDNKDYFDKMAPDLGNLFLIVVCLLTSRVFLVHTGVLCRIFSSLQPVLY
LIEQNSYFAVSIKIFTRVSENARLPVCTRGAREGNSNFVFKCFFYLSRLKVVATVIVK

>DmelIR84a

MIKLQVKVISLPLIILTAFLRVLQIESINTNFLELAAFEDFLRSEHLSHVLVVRGDDADGDWKIECHQKLLANYRVQFYRPEMSANFEDL
MFYGSPRTAVLVLNSQHVLVRRQVFGVASEAGYFNNSLAWFILGSGRESLPVEQLIDQLLSGYRMGIDADITVALRGPDNASMLFYDVY
RISRQANTPLIIEKKGLWTHSGGYQKLGNFKNTWVIRRRNFLNVTLIGSTVLTEKPPGFGDMEYLADDKQLQQLDPMQRKTYQLFQLV
ERMFNLSLAISLTDKWGELLDNGSWSGVMGQVTSREADFAVCPIRFVLDRQPYVQYSAVLHTQNIHFLFRHPRRSHIKNIFFEPLSNQV

WWCVLALVTGSTILLLFHVRLERMLSNMENRFSFVWFTMLETYLQQGPANEIFRLFSTRLLISLSCIFSFMLMQFYGAFIVGSLLSESARS
IVNLQALYDSNLAIGMENISYNFPIFTNTSNQLVRDVYVKKICKSGEHNIMSLQQGAERIIQGRFAFHTAIDRMYRLLLELQMDEAEFCD
LQEVMFNLPYDSGSVMPKGSPWREHLAHALLHFRATGLLQYNDKKWMVRRPDCSLFKTSQAEVDLEHFAPALFALALAMVASALVFL
LELFLHWLPDFRRRLGTMST
>DmelIR31a
MNLLISMFILILAAGEGEIIPSMEESVVTNFVKSLVKTKQAIVFSCLFKDFKEISLALMRINQFVSVVNLNQSYSLTSILTRENYARTSVMV
NARCSGSSELLFEASENRYFNKTYQWFLWGVDLEVQSLFPLNLNYVGPNAQITYVNETADGYAYWDIHSKGRHLKSNLEINLIATLIND
TLNIARDIFHLQSIDFRGQFNGLTLRGASVIDKEDIISNEQIESILSRPTKDAGVAAFIKYHYELLGLLRERFNFTVNFRNSRGWAGRLGNT
TFRLGLLGIVMRNEADIAASGAFNRINRFAEFDTIHQSWKFETAFLYRYTSDLDTHGKSGNFLSPFSDRVWLFCLLTLGAFSIIWVLFEIID
YKILRIRVNSQKLEHLNQKSSVICIKTTCIERILQTFGACCQQGLDPNPVDRSVRFLVMTLFLFSLVMYNYYTSSVVGGLLSSSDQGPSTV
DEITASPLKISFEDIGYYKVLFRESQNRSITRLIEKKLSSSRSLNELPIFSHIEDAVPYLKAGGFAFHCEVVDAYPVISEYFDANEICDLREVS
GLMEVEILNWILHKNSQYTEIFKTAMCNAQEKGFVERILRRRQIKKPACQSLYTVYPVSLSGVLPGFVILICGFGASLLLLCLEKVYAHFG
PRKFCGF
>DmelIGluRIB
MRFGLKLSCLWPSFLLWLTWSSGGGGGRWVGVSAQPSLTEKIPLGAIFEQGTDEVQSAFKYAMLNHNLNVSSRRFELQAYVDVINTAD
AFKLSRLICNQFSRGVYSMLGAVSPDSFDTLHSYSNTFQMPFVTPWFPEKVLTPSSGFLDFALSMRPDYHQAIIDTIQFYGWRKIIYLYDS
HDGLLRLQQIYQGLRPGNESFQVELVKRISNVSMAIEFLHTLEQIGRFENKHIVLDCPTEMAKQILIQHVRDLRLGRRTYHYLLSGLVMD

DRWESEIIEFGAINITGFRIVDTNRRLVREFYDSWKRLDPQMSVGAGRESISAQAALMYDAVFVLVEAFNKILRKKPDQFRNNVQRRSQ
TLMVAQAAASTSSDGYNYSASGGGGGNGGAGGGFAGSDSGGSGGMASRALDCNTAKGWVNAWEHGDKISRYLRKVEIEGLTGDIKF
NDDGRRVNYTLHVVEMTVNSAMVKVAEWNDDAGLQPLNAKYVRLRPHVEFEKNRTYIVTTVLEEPYIMLKQVAFGEKLHGNNRFEG
YCKDLADLLAKELGINYELRLVKDGNYGSEKSSAHGGWDGMVGELVRKEADIAIAAMTITAERERVIDFSKPFMSLGISIMIKKPVKQT
PGVFSFMNPLSQEIWVSVIFSYIGVSIVLFFVSRFSPHEWRLVQQQPQQSQSPDPHAHHEQLANQQPPGIIGGAPLPAPPGPPTPGAQTAA
GAAALQAALSAGSPGSGGSSSAVVNEFSVWNSFWFSLAAFMQQGCDLSPRSVSGRIAAASWFFFTLILISSYTANLAAFLTVERMVTPIN
SPEDLAMQTEVQYGTLLHGSTWDFFRRSQIGLHNKMWEYMNSRKHVFVPTYDEGIKRVRNSKGKYALLVESPKNEYVNAREPCDTM
KVGRNLDTKGFGIATPLGSALKDPINLAVLTLKENGELIKLRNKWWYEKAECSTHKDGETSHSELSLSNVAGIFYILIGGLLVSVFVAILE
YCFRSRDSRSASSGSGMGLGMGLGGGMSGGSLGKANGSMMLGPSSAVPGGMPSSHQRSTLTDTMHAKAKLTIQASRDYDNGRVGYL
NCASLQYYPPAQLSATPPDAGDSLHMNAHGQV

>SlitIR41a

MLLPTISLPLEILLNTIITQYLDSSYCVTVFSDKPLSPIISTSFIYLIPDEENLVEQIYNVSERGCSDYIVRMRDPQNFMTAFERVVHIGNVRR
SDRKIIILPYNEEYNDNNDENLPSLIFSMKGSEYLANMLMVVNHNSSNSDCKEFDLITHQYVGPDDVSNLPKYLDRWDSCSQQFENNA
NLFPHDMTNLFGKTLRVACFTYKPYALLDIDTAIEPLGRDGVEIRIVDEFCRWVNCTVEVVREDVDQWGEIYKNESGGIGVIGSVVKDR
ADLGITALYSWYEEYRVMDFSVAGVRTAITCIAPAPRLLSSWEMPLMPFTWYMWLAVVFTYFICLNWDFNSTGIWFIIVSILNAFGMMIG
QSQYEGKPSWKIRSVTGWLLIAGLILSSAYGAGLASTFTVPRYEPSIDTVQDIVDRKMEWGATHDAWIFSLTLSTEPLVKELVSQFRIYSF
DELKRKSFTRSMAYSIEKLPAGNFAIGEYVTQEAILDMMVMLEDFYYEQCVVMMRKSSPYTEKVSQLIGRLHQSGLLLAWETQVALKH

LNYKVQVEVRLSRSKNDVGTTKALNLGNVMGIFIVYAIGLMLSIATFLGELYVHHHKQKKERIHVD

>AtraIR25a

MLATFTANLAAFLTVERMQTPVSSLEQLARQSRINYTVVEGSTIHQYFINMKFAEDTLYRVWKEITLNATSDQAQYRVWDYPIREQYGH

ILLAINASGPVPDAKTGFEQVNEHTDADFAFIHDSAEIKYEVTRNCNLTEVGEVFAEQPYAIAVQQGSRLQ

EDL

>HvitIR8a

MLATFTANLAAFLTVERMQTPVSSLEQLARQSRINYTVVESSTIHQYFINMKFAEDTLYRVWKEITLNATSDQAQYRVWDYPIREQYGHI

LLAINASGPVPDAKTGFEQVNEHTDADFAFIHDSAEIKYEVTRNCNLTEVGEVFAEQPYAIAVQQGSRLQ

EHL

>HkahIR25a

MLATFTANLAAFLTVERMQTPVSSLEQLARQSRINYTVVESSTIHQYFINMKFAEDTLYRVWKEITLNATSDQAQYRVWDYPIREQYGHI

LLAINASGPVPDAKTGFQQVNDHADADFAFIHDSAEIRYEVTRNCNLTDVGEVFAEQPYAIAVQQGSRLQ

EDL

>AZB49403.1:592-754 ionotropic receptor 8a [Heortia vitessoides]

MLATFTANLAAFLTVERMQTPVSSLEQLARQSRINYTVVESSTIHQYFINMKFAEDTLYRVWKEITLNATSDQAQYRVWDYPIREQYGHI

LLAINASGPVPDAKTGFEQVNEHTDADFAFIHDSAEIKYEVTRNCNLTEVGEVFAEQPYAIAVQQGSRLQ

EHL

>DglaIR20

MLATFTANLAAFLTVERMQTPVSSLEQLARQSRINYTVVEGSTIHQYFINMKFAEDTLYRVWKEITLNATSDQAQYRVWDYPIREQYGH

ILLAINASGPVPDAKTGFEQVNEHTDADFAFIHDSAEIKYEVTRNCNLTEVGEVFAEQPYALAVQQGSRLQ

ERL

>CpinIR5

MLATFTANLAAFLTVERMQTPVSSLEQLARQSRINYTVVEGSTIHQYFINMKFAEDTLYRVWKEITLNATSDQAQYRVWDYPIREQYGH

ILLAINASGPVPDAKTGFEQVNEHTDADFAFIHDSAEIKYEVTRNCNLTEVGEVFAEQPYSLAVQQGSRLQ

EQL

>PxylIR25a

MLATFTANLAAFLTVERMQTPVSSLEQLARQSRINYTVVEGSTIHEYFINMKFAEDTLYRVWKEITLNATSDQAQYRVWDYPIREQYGH

ILLAINASGPVPDAKTGFEQVNEHTDADFAFIHDSAEIKYEVTRNCNLTEVGEVFAEQPYAIAVQQGSRLQ

EDL

>GmelIR25a

MLATFTANLAAFLTVERMQTPVSSLEQLARQSRINYTVVEGSTVHQYFINMKFAEDTLYRVWKEITLNATSDQAQYRVWDYPIREQYG

HILLAINATGPVPDAKTGFDQVNEHTDADFAFIHDSAEIKYEVTRNCNLTEVGEVFAEQPYALAVQQGSRLQ

EDL

>GmelIR8a

MLATFTANLAAFLTVERMQTPVSSLEQLARQSRINYTVVEGSTVHQYFINMKFAEDTLYRVWKEITLNATSDQAQYRVWDYPIREQYG

HILLAINATGPVPDAKTGFDQVNEHTDADFAFIHDSAEIKYEVTRNCNLTEVGEVFAEQPYALAVQQGSRLQ

EDL

>AgriIR25a

MLATFTANLAAFLTVERMQTPVSSLEQLARQSRINYTVVEGSTVHQYFINMKFAEDTLYRVWKEITLNATSDQSQYRVWDYPIREQYG

HILLAINATGPVPDAKTGFDQVNDHTDADFAFIHDSAEIKYEVTRNCNLTEVGEVFAEQPYALAVQQGSRLQ

EDL

>HarmIR8a

MLATFTANLAAFLTVERMQTPVSSLEQLARQSRINYTVVEGSSVHQYFINMKFAEDTLYRVWKEITLNATSDQAQYRVWDYPIREQYG

HILLAINASEPVPDAKTGFQQVNEHTDADFAFIHDSAEIKYEVTRNCNLTEVGEVFAEQPYAIAVQQGSRLQ

EEL

>PintIR25a

MLATFTANLAAFLTVERMQTPVSSLEQLARQSRINYTVVEGSTVHQYFINMKFAEDTLYRVWKEITLNATSDQAQYRVWDYPIREQYG

HILLAINASGPVPDAKTGFHQVNEHTDADFAFIHDSAEIKYEVSRNCNLTEVGEVFAEQPYAIGVQQGSRLQ

EAL

>SlitIR8a

MLATFTANLAAFLTVERMQTPVSSLEQLARQSRINYTVVEGSSVHQYFVNMKFAEDTLYRVWKEITLNATSDQAQYRVWDYPIREQYG

HILLAINASQPVPDAKTGFQQVNEHTDADFAFIHDSAEIKYEVTRNCNLTEVGEVFAEQPYAIAVQQGSRLQ

EDL

>AageIR25a

MLATFTANLAAFLTVERMQTPVSSLEQLARQSRINYTVVEGSTVHQYFINMKFAEDTLYRVWKEITLNATSDQTQYRVWDYPVREQYG

HILLAINASGPVPDAKTGFQQVNEHTEADFAFIHDSAEIKYEITHNCNLTEVGEVFAEQPYALAVQQGSRLQ

EDL

GR:

>LstiGR1

MDEEKQMFRIYNTNQINGKQKNTNGIREEYDAKDIYGPEITDKDGALLDEHDSFYHTTKSLLVLFQIMGVMPIMRVPKDAQTTNRTTF

NWISKATLWAYLVWSLECIIVVRVGKERLATFQQNTNKRFDEVIYNIIFLSILIPHFLLPVASWRHGPQVAIFKNMWTHYQLKYRKITGTPI

VFPNLYILTWGLCVFSWGLSFAVILSQHYLQEDFELWHSFAYYHIIAMLDGFCSLWYINCNAFGTASRGLAMNLHKALKAEHPALKLAQ

YRHLWVDLSHMMQQLGRAYSNMYGIYCMVIFFTTTISLYGALSEILERGLSYKEMGLFVIVGYCMTLLYIICNEAYHATRKVGFEFQVR

LLNVNLGAIDRSTQREVEMFLVAIAKNPPIMNLDGFTNINRELFAANISFMSTYLIVLMQFKLTLLRQSARKAIKTVVKAIFNTTTLGPDD

EDDDVEEE

>LstiGR2

MDLSEPSDRSVRAFSTTRRCVWLCDFSIVMTISCLAVFRGPRRTRAVLSCLGHVQKINHNLDVPRPTIWKEIKKYVAIILYTTFIVSTIVGD

FVFILAIANYFDPTGEQTRITYFYTGYYYAYTTLFFTVMQFIICALEIYRTMRSLNILLEEVAQAASLKKTKILNPFVGINVVQMKALNKIT
DSLSVNDSVYPGITKSSKTAHETIRHLSLMYSSACEAVRQLNDCFAPELLALLMSFLLHLVVTPYNCIANISGGKINMQLLTMQLLWCSI
HFVSLIVMVEPCHITQREMGRTNFLVSQLMLQNTDELVTNELNVFGRYLYLNDVVYSPMGICVLSRSLVASILASVTTYLVIMMQFQATE
NIVYHG
>LstiGR64a
MSETPNLSKLESRQQDKFLPTLTHIFTVAQWFGIPSYGNKFAICWAVIVLCMLTVVEGAAIWMMIRLLAGIAKHIDDGRGLTARLSGSIFY
ANGFLSLILSWKFMYSWKRLSFYWKRAELVDVSLAIPDEAIQRKVIVVTCFVSVCAFAEHLLSMILAIGIDSPPMDFLERYILNSHAFLIT
PNTYSLWSAILIFFVSKIATILWNFQDLIIILISMGLTSRYHRLNSFVNMCVKNEKINRDKTSVTEKYVRTHQWRRIREAYVRQAALVRMV
DANIGALVLLSNVNNFYFICLQLFLGLTKSQGSLVSYLYYFISLGWLLFRACSVVLAAADVHIHSRRALEYLQTCPGTGFNIEIMRLNNQ
LSHDFVALSGMGFFSLSRQTLLEVAGNIIKYELVLIQYDK
>DmelGR64f
MKILPKLERKLRRLKKRVTRTSLFRKLDLVHESARKKAFQESCETYKNQIENEYEIRNSLPKLSRSDKEAFLSDGSFHQAVGRVLLVAEFF
AMMPVKGVTGKHPSDLSFSWRNIRTCFSLLFIASSLANFGLSLFKVLNNPISFNSIKPIIFRGSVLLVLIVALNLARQWPQLMMYWHTVE
KDLPQYKTQLTKWKMGHTISMVMLLGMMLSFAEHILSMVSAINYASFCNRTADPIQNYFLRTNDEIFFVTSYSTTLALWGKFQNVFSTF
IWNYMDLFVMIVSIGLASKFRQLNDDLRNFKGMNMAPSYWSERRIQYRNICILCDKMDDAISLITMVSFSNNLYFICVQLLRSLNTMPS
VAHAVYFYFSLIFLIGRTLAVSLYSSSVHDESRLTLRYLRCVPKESWCPEVKRFTEEVISDEVALTGMKFFHLTRKLVLSVAGTIVTYELVLI
QFHEDNDLWDCDQSYYS

>DmelGR64a

MKGPNLNFRKTPSKDNGVKQVESLARPETPPPKFVEDSNLEFNVLASEKLPNYTNLDLFHRAVFPFMFLAQCVAIMPLVGIRESNPRRV

RFAYKSIPMFVTLIFMIATSILFLSMFTHLLKIGITAKNFVGLVFFGCVLSAYVVFIRLAKKWPAVVRIWTRTEIPFTKPPYEIPKRNLSRRVQ

LAALAIIGLSLGEHALYQVSAILSYTRRIQMCANITTVPSFNNYMQTNYDYVFQLLPYSPIIAVLILLINGACTFVWNYMDLFIMMISKGL

SYRFEQITTRIRKLEHEEVCESVFIQIREHYVKMCELLEFVDSAMSSLILLSCVNNLYFVCYQLLNVFNKLRWPINYIYFWYSLLYLIGRT

AFVFLTAADINEESKRGLGVLRRVSSRSWCVEVERLIFQMTTQTVALSGKKFYFLTRRLLFGMAGTIVTYELVLLQFDEPNRRKGLQPLC

A

>DmelGR5a

MRQLKGRNRCNRAVRHLKIQGKMWLKNLKSGLEQIRESQVRGTRKNFLHDGSFHEAVAPVLAVAQCFCLMPVSGISAPTYRGLSFNRR

SWRFWYSSLYLCSTSVDLAFSIRRVAHSVLDVRSVEPIVFHVSILIASWQFLNLAQLWPGLMRHWAAVERRLPGYACCLQRARPARRLK

LVAFVLLVVSLMEHLLSIISVVYYDFCPRRSDPVESYLLGTSAQLFEVFPYSNWLAWLGKIQNVLLTFGWSYMDIFLMMLGMGLSEML

ARLNRSLEQQVRQPMPEAYWTWSRTLYRSIVELIREVDDAVSGIMLISFGSNLYFICLQLLKSINTMPSSAHAVYFYFSLLFLLSRSTAVLL

FVSAINDQAREPLRLLRLVPLKGYHPEVFRFAAELASDQVALTGLKFFNVTRKLFLAMAGTVATYELVLIQFHEDKKTWDCSPFNLD

>DmelGR64e

MARTTGDPAKRRRCMSRIKFWRRSRVGSEVVEKDTKRFKLSLIKAWLLRIRQEDYKYSGSFQEAIKPVLIIAQIFALMPVRKVSSKFAED

LTFTWFSVRSYYALVTILFFGVSSGYMVAFVTSVSFNFDSVETLVFYLSIFLISLSFFQLARKWPEIAQSWQLVEAKLPPLKLPKERRSLAQ

HINMITIVATTCSLVEHIMSMLSMGYYVNSCPRWPDRPIDSFLYLSFSSVFYFVDYTRFLGIVGKVVNVLSTFAWNFNDIFVMAVSVALA

ARFRQLNDYMMREARLPTTVDYWMQCRINFRNLCKLCEEVDDAISTITLLCFSNNLYFICGKILKSMQAKPSIWHALYFWFSLVYLLGR
TLILSLYSSSINDESKRPLVIFRLVPREYWCDELKRFSEEVQMDNVALTGMKFFRLTRGVVISVAGTIVTYELILLQFNGEEKVPGCFEN

>DmelGR64c

MQQSGQKGTRNTLQHAIGPVLVIAQFFGVLPVAGVWPSCRPERVRFRWISLSLLAALILFVFSIVDCALSSKVVFDHGLKIYTIGSLSFSVI
CIFCFGVFLLLSRRWPYIIRRTAECEQIFLEPEYDCSYGRGYSSRLRLWGVCMLVAALCEHSTYVGSALYNNHLAIVECKLDANFWQNYF
QRERQQLFLIMHFTAWWIPFIEWTTLSMTFVWNFVDIFLILICRGMQMRFQQMHWRIRQHVRQQMPNEFWQRIRCDLLDLSDLLGIYD
KELSGLIVLSCAHNMYFVCVQIYHSFQSKGNYADELYFWFCLSYVIIRVLNMMFAASSIPQEAKEISYTLYEIPTEFWCVELRRLNEIFLS
DHFALSGKGYFLLTRRLIFAMAATLMVYELVLINQMAGSEVQKSFCEGGVGSSKSIFS

>DmelGR64d

MERSVQENTLHYTIGHVLIIARIFGVLPLAGINPNGKPENVRFRWFSPYILFFVVAFTFVIADFMLSTKIVLNDGLQLYTMGSLSFSVICIFC
FGSFIKLSRRWPHIIRETALCERIFLKPCYANQEGLNFTRFLRRWALILLVAALCEHLTYVGSAAWSNYVQIRDCNLKVGFVENYFLRERQ
ELFSVFEYRAWMVFFIEWNTMAMTFVWNFGDIFLFLMCRGLKIRFQQLHWRIRQNLGKPMAKEFWQEIRSDFLDLDSLLKLYDKELSG
LILVCCAHNMYFICVQVYHSFQVKGAFMDELYFWFCLLYVISRLMNMMLAASSIPQEIKDISNTLYEVRSSPWCDELGRLSEMLRNETF
ALSGMGYFYVTRRLIFAMAGALMGYELVLFRQMQGAVVQKSICSRGPGSSMSIFFS

>BmorGR64a

MDKDKFQEFLPTMSRIFSMTRYFGVSTCKPSIAFGWTVILLLMLLAIEVGAIWKIVRLLGGWAVHSTDRGFTARLSGCIFYGNALLSLILS
IKFVSSWEQLSERWSRTETDPGLRLPSDSRIKRRTVLVSAFVMTCACVEHMLSMMSATGFDCPPEEYTERYILSSHGFLVQNDEYNLWL

AIPIFIMSKLATALWNFQDLIIILISMGFTSRYNRLNTYVHRVVMLERNLKEGAQVSSENYMRFQIWRRIRQAYVRQAALVRLVDDQLGA

LVLLSNVNNLYFICLQLFLGINSKDRGSFINRLYYFISLGWLMFRACGVVLAAADVYIHSKKALISLYLCPELAYNLEIKRLKYQLKNDE

VALTGMGLFSLNRELLLEVAAAVLKYELVLVQYDK

>BmorGR64e

MDTTKQPIVSGLSNQLEALNFKALLSDNSINLNRNRKHIKSTTRGNFRERILRKVQNRISPEPIQEDSKIPLTCQFQLFQTAMKHLLISGQF

MGLNPVSRISDHSPTKIRFTVLSWKFVYGVTIGIAQACATVLCFCKLLKDSVNIVALAYFAFYLSTGCNTFIFLRVASKWPTLIKHVYETQ

LDSYIDVKVKNKCFAAYIIFFSMSMTEHMLSLLSKFVITMDCLPKGSDLFESYIIRNFPWLFEFDVPYYLPIGVILQFLTLVSTINWSYSDLF

IVCMSIYLTSILKQINKKIEMAGNSNHLPIPFWRTLREDYTRATRLVRSFDDTISSVIFLSFASNLFFICLQLYNILSNGVTSKYNLLKEMCP

NYPSGPLGGYEQIMYLLFSLSFLLGRSLVVSLVAAKVHSASMVPASALYNIPRNMYCSEIQRFLDQVHGDKVALSGLRFFYVTRSLVLSV

AGTIVTYELVLLQFSNED

>BmorGR8

MAPRSVRSMVGTSKKDMLKGGFYETVRIPLYIYRLIGILPISGLWHRSSKYNRFSLKSFYTIIYAPTIVMQTFLLLVHIYDLFAFFFGHQRL

GRLIYHMNFYTITILIFMGSRKWKNVIKEIETIELTLPRLRNSKKALALTKSFVFAFFVFSLAEVVLILQFTLRLTKQRHVLPGDSGLYLRS

YFVYIFPYLYDHFPFSYVMGFIVQIIKVQGIITLNMVNCSVVILSIYLTNRLKHYNRIVFAKGSKTNNTRLKWVELNLLYTRISNLVKIIDK

NLNPFVFISFTANLSYICAQLFYILNKLTSSRTVKITSFLEDKRSDWETVLYISISFALVVLKVLLVSITAAEVHTTSREPLRLLYTLPTAEYTI

ETQRLMTQVYYSNLSLSGLNFFHITRGMLLGMVATLLTYEIVLLQI

>HarmGR7

MSSRGFGQFLRDGNLILPEQPNHDDFLTVMEKVFKWSCLIGVLGSKRHINYAWSGFILLVLLFMESQAIWKVIKALAGWAIDTAGQRSV
TARLAGTIFYTIAILSLILSSRLYRSWGQLSALWARVERIMAVKAPPDKTLKRRMYFFLGFMTVCSLLEHIMSVVSAIGLDCPPALIIKRYV
LISHGFMILRHEYSDWYALPLIFMSTLASLLWNFQDVLIVLISMGLTSRYSRLNQCLAKICALERKQMDSDKKNETTKVYAWRKLREAY
VKQAMLVRKVDDAIGGIIILSCFCNFYFICLQLFLGITQSKASEPIKTAYYFMSLGWICFRVICVVLAASDINVHSRLGLKYIYTHDSHSYN
IEMGRLQDQLSKDYVALSGKGFFYLSKSILLQMAGAIITYELMLIQFDDQGTDDVQLNLTKNAIGV

>HarmGR5

MSSKEFKQFLRQNKLLLPQQPIHDDFLGVIEKVFHWSCFYGVFGSKRFISLIWSTLILGSLVIIEVLAIWKVIRALAGVARDMSGHRSVTA
RLAGTIFYSISILSLVLISKLYYNWRINIAGVWAKVERSVGVKIPVDKTLKCRMSFVAGLMTFCSFFEHALSILASVGFDCPPSLILKRYVL
VSHGFIFMGQDYSEWFAMPLVVISTIATLLWNFQDQLIVLISMGLTSRYRRLNECLAKFCELEKQHMDSDKKVEAVKVYTWRKMREAY
VKQAMLVRKIDVALGGIIILSCSCNFYFICLQMFLGITQGMSTDFLTGLYYMVSLAWLCIRVLSVVLAASGVNTHSKLALNHLYTYETHC
YNVEVERLQDQLTKDYIALSGMGFFYLNKTILLQMAGAIITYELVLIQFDDQGSDGIALNATNI

>HarmGR1

MGQTSFRRNMSFWIPVKKNKVDVAKPKVKNITSFQDALRVTVIIGQVFSLLPFVGVFTNVASNVKFVKTSWKCVYSLLSLIGQMFMAV
LCINKLAKTTVSLNGTSPVIFYVTTCVTMMLFFQVARRWPALVQHISKAEDMDPNFDCSLTRKCNITCAVVLILALCEHILSLLSAFAGAS
ACYSGMDTYEGFVTHFYPWVFSYLPYSIVLGVITQFLHFQSTFIWNFSDLFVICMSYYLTSRLEQVNRKLLAAQGKYLPEIFWRATRED
YCRATQIVRKVDEVISGVVFISFANNLFFICLQLFNTLEDGLKGTGECTPKLKKIVVSKSGPLGGHEAAAYFLFSLVYLLSRSVAVSLIASQ
VNSASSVPAPVLYDVPSPVYCVEVQRFLDQVNGDKVALSGLQFFSVTRGLLLTVAGTIVTYELVMFQFNSSTPTLNITSPTVVTHTITTLA

T

>HarmGR5a

MGQTSFRRNMSFWIPVKKNKVDVAKPKVKNITSFQDALRITVIIGQVFSLLPFVGVFTNVASNVKFVKTSWKCVYSLLSLIGQMFMAVL

CINKLAKTTVSLNGTSPVIFYVTTCVTMMLFFQVARRWPALVQHISKAEDMDPNFDCSLTRKCNITCAVVLILALCEHILSLLSAFAGASA

CYSGMDTYEGFVTHFYPWVFSYLPYSIVLGVITQFLHFQSTFIWNFSDLFVICMSYYLTSRLEQVNRKLLAAQGKYLPEIFWRATREDY

CRATQIVRKVDEVISGVVFISFANNLFFICLQLFNTLEDGLKGTGECTPKLKKIVVSKSGPLGGHEAAAYFLFSLVYLLSRSVAVSLIASQV

NSASSVPAPVLYDVPSPVYCVEVQRFLDQVNGDKVALSGLQFFSVTRGLLLTVAGTIVTYELVMFQFNSSTPTLNITSPTVVTHTITTLAT

>HarmGR6

MGQTSFRRNMSFWIPVKKNKVDVAKPKVKNITSFQDALRVTVIIGQVFSLLPFVGVFTNVASNVKFVKTSWKCVYSLLSLIGQMFMAV

LCINKLAKTTVSLNGTSPVIFYVTTCVTMMLFFQVARRWPALVQHISKAEDMDPNFDCSLTRKCNITCAVVLILALCEHILSLLSAFAGAS

ACYSGMDTYEGFVTHFYPWVFSYLPYSIVLGVITQFLHFQSTFIWNFSDLFVICMSYYLTSRLEQVNRKLLAAQGKYLPEIFWRATRED

YCRATQIVRKVDEVISGVVFISFANNLFFICLQLFNTLEDGLKGTGECTPKLKRIVVSKSGPLGGHEAAAYFLFSLVYLLSRSVAVSLIASQ

VNSASSVPAPVLYDVPSPVYCVEVQRFLDQVNGDKVALSGLQFFSVTRGLLLTVAGTIVTYELVMFQFNSSTPTLNITSPTVVTHTITTLA

T

>HarmGR11

MPSKLFLKTFNMATRHRLDKREICGLHSTVRGTLFCSRVMGLLPVSGLTCPTSRRLRFTFRSPYTILYVASLFGQLLMFVMTLCWLMMN

GISLANITNAVFYTSSLISSLILLHIGRCWPALVGSVETLERELPPFHRNVASISNVTTIFILTAAIVEHLLSVFYGLKVACACDSNNVAENYF

RFNMPWIFDYTPFTIWKGALSELFNIQSTFVWSLNDLLIMVISIYLTEHLLIHNELLKKAAEQEHFSCLEFRTQYLKIVRLVKLINGQFGIYI

LTSFGSNLYWICTQLFYSLSRTQTGHFITCTFKDSPTKVPPANEYENPLCPWMLGEKGALNGVEHSIYFTYSFSFLLLRTLLVLLLAARIHS

NSVAPLYVLYGIPSSRFHIEVERFIAQINNLKVAMSGLDFFYVTRTMIVTLLGTIVTYELVLLQFNR

>DmelGR21a

MSFWAVSRGLTPPSKVVPMLNPNQRQFLEDEVRYREKLKLMARGDAMEEVYVRKQETVDDPLELDKHDSFYQTTKSLLVLFQIMGV

MPIHRNPPEKNLPRTGYSWGSKQVMWAIFIYSCQTTIVVLVLRERVKKFVTSPDKRFDEAIYNVIFISLLFTNFLLPVASWRHGPQVAIFK

NMWTNYQYKFFKTTGSPIVFPNLYPLTWSLCVFSWLLSIAINLSQYFLQPDFRLWYTFAYYPIIAMLNCFCSLWYINCNAFGTASRALSD

ALQTTIRGEKPAQKLTEYRHLWVDLSHMMQQLGRAYSNMYGMYCLVIFFTTIIATYGSISEIIDHGATYKEVGLFVIVFYCMGLLYIICNE

AHYASRKVGLDFQTKLLNINLTAVDAATQKEVEMLLVAINKNPPIMNLDGYANINRELITTNISFMATYLVVLLQFKITEQRRIGQQQA

>DmelGR63a

MANYYRRKKGDAVFLNAKPLNSANAQAYLYGVRKYSIGLAERLDADYEAPPLDRKKSSDSTASNNPEFKPSVFYRNIDPINWFLRIIGV

LPIVRHGPARAKFEMNSASFIYSVVFFVLLACYVGYVANNRIHIVRSLSGPFEEAVIAYLFLVNILPIMIIPILWYEARKIAKLFNDWDDFE

VLYYQISGHSLPLKLRQKAVYIAIVLPILSVLSVVITHVTMSDLNINQVVPYCILDNLTAMLGAWWFLICEAMSITAHLLAERFQKALKHI

GPAAMVADYRVLWLRLSKLTRDTGNALCYTFVFMSLYLFFIITLSIYGLMSQLSEGFGIKDIGLTITALWNIGLLFYICDEAHYASVNVRT

NFQKKLLMVELNWMNSDAQTEINMFLRATEMNPSTINCGGFFDVNRTLFKGLLTTMVTYLVVLLQFQISIPTDKGDSEGANNITVVDFV

MDSLDNDMSLMGASTLSTTTVGTTLPPP

IMKLKGRKG

>HassGR1

WSLSFAVILSQHYLQDDFELWHSFAYYHIIAMLDGFCSLWYINCNAFGTASRGLAMNLHKALEAEHPALKVAQYRHLWVDLSHMMQQ

LGRAYSNMYGIYCMVIFFTTTISLYGALSEILEHGLSYKEMGLFVIVGYCMTLLFIICNEAYHASRKVGLEFQVRLLNVNLGAVDRSTQR

EVEMFLVAISKNPPIMNLDGFTNINRELFTANVSFMSTYLIVLMQFKLTLLRQSARKTLKTIVRAVFNTTTTILDDDFTD

>HassGR2

GYERIMILRSIRRFDEYIYAILFVIFLVPHFWIPFVGWGVAHQVAIYKTNWGKFQVRYYRVTGENLKFPNLKTTIVIISVGCLLLAVCFLLS

LCILMDGFLLRHTTAYYHIITMINMNCALWYINCKGIKIASQSLSECFRRDVEAECSAKLISRYRYLWLNLSELLQSLGNAYARTYSTYCL

FMFANITIAVYGALSEIVDHGFGFSFKEMGLFVDAAYCSTLLFIFVDCSHNSTLTVAAGVQDTLLSIDVLAVDRPTQKEIDHFIQAIEMNPA

VVSLKGYAHVNRELLTSAISMIAIYLIVLLQFKISLPRDPQIVAT

>HassGR3

MTVPIPNGFPVQINSKPKNKIIFLDVTPVSTPIKPHSPNVVAPMRNNLVAPHISNDIIYENIKPVFTLLRIMGVLPITRPSACVNQFQIASSSM

LYAILVFISLVSYVLYLSLHKVQILRTAEGKFEEAVIEYLFTVYLFPMIAVPLLWYETRKIANVLNGWVDFEMVYKQLSGRTLPVKLYKKA

LAMAVIIPILSTTTVIVTHVTMVHFKPMQLVPYVFLEILTYMLGGYWYLLCETLSICANILAEDFQNALRHIGPAGKVAEYRALWLRLSK

LSRDTGIANCYTFTFVNLYLFLIITLSIYGLLSQISEGFGIKDIGLALTAFCSISLLFFICDEAHYASHNVRTNFQKKLLMVELSWMNTDAQT

EVNMFLRATEMNPSQISLGGFFNVNRTLFKSLLATMVTYLVVLLQFQISIPDESQNRDEEEEVPYNITSATTEAMTTSTTTIMTTVLTTLA

KKKKKN

>DmelGR43a

MEISQPSIGIFYISKVLALAPYATVRNSKGRVEIGRSWLFTVYSATLTVVMVFLTYRGLLFDANSEIPVRMKSATSKVVTALDVSVVVMAI
VSGVYCGLFSLNDTLELNDRLNKIDNTLNAYNNFRRDRWRALGMAAVSLLAISILVGLDVGTWMRIAQDMNIAQSDTELNVHWYIPFY
SLYFILTGLQVNIANTAYGLGRRFGRLNRMLSSSFLAENNATSAIKPQKVSTVKNVSVNRPAMPSALHASLTKLNGETLPSEAAAKNKGL
LLKSLADSHESLGKCVHLLSNSFGIAVLFILVSCLLHLVATAYFLFLELLSKRDNGYLWVQMLWICFHFLRLLMVVEPCHLAARESRKTIQ
IVCEIERKVHEPILAEAVKKFWQQLLVVDADFSACGLCRVNRTILTSFASAIATYLVILIQFQRTNG

>DmelGR28b

MDIEMAKEPVNPTDTPDIEVTPGLCQPLRRRFRRFVTAKQLYECLRPVFHVTYIHGLTSFYISCDTKTGKKAIKKTIFGYINGIMHIAMFV
FAYSLTIYNNCESVASYFFRSRITYFGDLMQIVSGFIGVTVIYLTAFVPNHRLERCLQKFHTMDVQLQTVGVKIMYSKVLRFSYMVLISMF
LVNVLFTGGTFSVLYSSEVAPTMALHFTFLIQHTVIAIAIALFSCFTYLVEMRLVMVNKVLKNLAHQWDTRSLKAVNQKQRSLQCLDSFS
MYTIVTKDPAEIIQESMEIHHLICEAAATANKYFTYQLLTIISIAFLIIVFDAYYVLETLLGKSKRESKFKTVEFVTFFSCQMILYLIAIISIVEG
SNRAIKKSEKTGGIVHSLLNKTKSAEVKEKLQQFSMQLMHLKINFTAAGLFNIDRTLYFTISGALTTYLIILLQFTSNSPNNGYGNGSSCC
ETFNNMTNHTL

>BmorGR9

MPPSPDLRADEPKTPCLVGGAHAFILKISSFCGLAPLRFEPRSQEYAVTISKGKCFYSYILVTFLVICTIYGLVAEIGVGVEKSVRMSSRMS
QVVSACDILVVAVTAGVGVYGAPARMRTMLSYMENIVAVDRELGRHHSAATERKLCALLLLILLSFTILLVDDFCFYAMQAGKTGRQW
EIVTNYAGFYFLWYIVMVLELQFAFTALSLRARLKLFNEALNVTASQVCKPVKKPKNSQLSVYATSVRPVSCKRENVIVETIRVRDKDD
AFVMMKTADGVPCLQVPPCEAVGRLSRMRCTLCEVTRHIADGYGLPLVIILMSTLLHLIVTPYFLIMEIIVSTHRLHFLVLQFLWCTTHLI

RMLVVVEPCHYTIREGKRTEDILCRLMTLAPHGGVLSSRLEVLSRLLMLQNISYSPLGMCTLDRPLMVTVLGAVTTYLVILIQFQRYDS

>BmorGR9X2

MTMDLNVITATKCDEFTRIYYKELCCLEATKIDQLPREMPPSPDLRADEPKTPCLVGGAHAFILKISSFCGLAPLRFEPRSQEYAVTISKGK

CFYSYILVTFLVICTIYGLVAEIGVGVEKSVRMSSRMSQVVSACDILVVAVTAGVGVYGAPARMRTMLSYMENIVAVDRELGRHHSAATE

RKLCALLLLILLSFTILLVDDFCFYAMQAGKTGRQWEIVTNYAGFYFLWYIVMVLELQFAFTALSLRARLKLFNEALNVTASQVCKPVK

KPKNSQLSVYATSVRPVSCKRENVIVETIRVRDKDDAFVMMKTADGVPCLQVPPCEAVGRLSRMRCTLCEVTRHIADGYGLPLVIILMS

TLLHLIVTPYFLIMEIIVSTHRLHFLVLQFLWCTTHLIRMLVVVEPCHYTIREGKRTEDILCRLMTLAPHGGVLSSRLEVLSRLLMLQNISY

SPLGMCTLDRPLMVTVLGAVTTYLVILIQFQRYDS

>BmorGR9X1

MKYDPFSGRVQKTILSPVISVREPPAQLTSRECCNPSNILFNCYLSLMGLEATKIDQLPREMPPSPDLRADEPKTPCLVGGAHAFILKISSF

CGLAPLRFEPRSQEYAVTISKGKCFYSYILVTFLVICTIYGLVAEIGVGVEKSVRMSSRMSQVVSACDILVVAVTAGVGVYGAPARMRTM

LSYMENIVAVDRELGRHHSAATERKLCALLLLILLSFTILLVDDFCFYAMQAGKTGRQWEIVTNYAGFYFLWYIVMVLELQFAFTALSLR

ARLKLFNEALNVTASQVCKPVKKPKNSQLSVYATSVRPVSCKRENVIVETIRVRDKDDAFVMMKTADGVPCLQVPPCEAVGRLSRMRC

TLCEVTRHIADGYGLPLVIILMSTLLHLIVTPYFLIMEIIVSTHRLHFLVLQFLWCTTHLIRMLVVVEPCHYTIREGKRTEDILCRLMTLAPH

GGVLSSRLEVLSRLLMLQNISYSPLGMCTLDRPLMVTVLGAVTTYLVILIQFQRYDS

>BmorGR10

MTMSIKPRLQCMVPPSLALALRVSRLAGIAPLKFVAKQSNIMIRLSTSLCVYSYLLVTALNVCTLIAVMIDFSVPVKLSIRMQTETKRFV

WIADVVIVGILSGVGVYTAPIQMRRLIAYLHRIHKINSDLGTYSSSLTDKMLHRLTIGMLLITSVIIVTDFTFVMYLADLNHRQLLIAIMY
WCYYCSYFIAHLLEMQFVLIAALALSSLKLVNNGLRTLLHQSGIESLTEIPNSNEQHTANAILPQPPKKSVNNSIDTLAFVVTKRSVRFPT
AGWMDQRTIRRLALSYGSICEVVRQIDNNNGIIVLLLLASFLLHLVVTPYYLIISFVTESPHTGFEKVLNPILQTVWCLYHTFGLVMIIEPC
HRTHEEMETTRELVSRVMCSADPRDPISIELEMFFRQLVLNKASYAPLKVCTLTRSLVATILGSITTYLIVIVQLEIKNMQ

>HarmGR4

MLWIETHHYLGVESAKVEEVTAAPVPSESGSRPSRPTHCVVGGTHAFILRISSFFGLAPLRFESRSNGFTVSISGAMCVYSYILVTVLVICT
IFGLVAEINVGVELSVRMSSRTSQVVSTCDVLVVVATAGAGVYGAPRRMRNMLKFMENIASVDTSIGGQYSLVTERKLCGIILAILIFFSIL
IADDFTFYALQAKKLDREWDVVTNYLGFYLLWFVVLILELQFAFTALSVRARFSAVNDALALTARQVSIPVEKPKSSSPLNIYAIRVAPVD
SQHSANVSLLVDTMTGREHVVIIKRTASGEPRLVVSPCDAVRRLAALHGTLCDVVNSIDDSYGLPLVVILISTLLHLIVTPYFLIMEIIVST
NRIHFLVLQFLWCVTHMLRMIVVVEPGHYTIAEGKRTEGLVCRLMTSAPSTGVLPSRLEIFSRQLMLQSVSYAPMGMCTLHRPLIASVIG
AVTTYLVILIQFQRYDN

>HarmGR43a

MSAFPDTECVVTGTLSMMLRMSQIAGVAPLSFRRTHGGWYIRTSRAANCYGKALSFCLWFLSSFTIAIDILIQPERSFRTRTNSTRIVWLA
DVATVAIVVCAAAFTGISRMRCLTVYALKLEEINLRLSLFHEEPSNEANRRLIAVSSMIFVVSTILVDYSIFIYQVITEHGKIVTSCMYIFYNI
STIVQQVILVTFSETVTSVLTSLQMLNNCLKNLLQEILDSSELTNCALNYDSYINMHPNRSAIPNKSINSVVDTMAVYKGYRKSSIKAVPS
TIRRLALLYCSICDVIRLVNDSHGLILVALMLCLLLHLVITPYHVITNICEFINKERSDRSSPLLQLNWAVLHFVNLLLIVEPCHRTHEEMEQ
TRHLISQMIRYTPSEHGVLLTELQMFYQHLILNEVSYAPLKMFSLNRSLIVTILGSITTYLVVVVQL

>HassGR4

MLWIQTHHYIGVESAKVEEVTAAPVPSESGSRPSRPTHCVVGGAHVFILRISSFFGLAPLRFESRSNGFTVTISGAMCVYSYILVTVLVICT

IFGLVAEINVGVELSVRMSSRMSQVVSTCDVLVVVATAGAGVYGAPRRMRNMLKFMENIASVDTSIGG

QYSLVTERKLCGIILAILIFFSILIADDFTFYALQAKKLDREWDVVTNYLGFYLLWFVVLILELQFAFTALSVRARFSAVNDALALTARQVS

IPVEKPKSSSPLNIYAIRVAPVDSQRSANVSLLVDTVTGREHVVIIKRTASGEPRLVVSPCDAVRRLAAPHGTLCDVVNSIDDSHGLPLVVI

LISTLLHLIVTPYFLIMEIIVSTNRIHFLVLQFLWCVTHMLRMIVVVEPRHYTIAEGERTEGLVCRLMTSVPSTGVLPSRLEIFSRQLLLQSV

SYAPVGMCTLHRPLIASVIGAVTTYLVILIQFQRYDN

>HarmGR43a

MSAFPDTECVVTGTLSMMLRMSQIAGVAPLSFRRTHGGWYIRTSRAANCYGKALSFCLWFLSSFTIAIDILIQPERSFRTRTNSTRIVWLA

DVATVAIVVCAAAFTGISRMRCLTVYALKLEEINLRLSLFHEEPSNEANRRLIAVSSMIFVVSTILVDYSIFIYQVITEHGKIVTSCMYIFYNI

STIVQQVILVTFSETVTSVLTSLQMLNNCLKNLLQEILDSSELTNCALNYDSYINMHPNRSAIPNKSINSVVDTMAVYKGYRKSSIKAVPS

TIRRLALLYCSICDVIRLVNDSHGLILVALMLCLLLHLVITPYHVITNICEFINKERSDRSSPLLQLNWAVLHFVNLLLIVEPCHRTHEEMEQ

TRHLISQMIRYTPSEHGVLLTELQMFYQHLILNEVSYAPLKMFSLNRSLIVTILGSITTYLVVVVQL

>HassGR4

MLWIQTHHYIGVESAKVEEVTAAPVPSESGSRPSRPTHCVVGGAHVFILRISSFFGLAPLRFESRSNGFTVTISGAMCVYSYILVTVLVICT

IFGLVAEINVGVELSVRMSSRMSQVVSTCDVLVVVATAGAGVYGAPRRMRNMLKFMENIASVDTSIGGQYSLVTERKLCGIILAILIFFSI

LIADDFTFYALQAKKLDREWDVVTNYLGFYLLWFVVLILELQFAFTALSVRARFSAVNDALALTARQVSIPVEKPKSSSPLNIYAIRVAPV

DSQRSANVSLLVDTVTGREHVVIIKRTASGEPRLVVSPCDAVRRLAAPHGTLCDVVNSIDDSHGLPLVVILISTLLHLIVTPYFLIMEIIVST

NRIHFLVLQFLWCVTHMLRMIVVVEPRHYTIAEGERTEGLVCRLMTSVPSTGVLPSRLEIFSRQLLLQSVSYAPVGMCTLHRPLIASVIGA

VTTYLVILIQFQRYDN

>OnubGR64a

FLPTLTHIFTVAQWVGIPTYGNKMSLVWAIIVLSMLTAIEAAAIWMLIKILTGVAKHIDDGRGLTARLSGSVFYGNGFLSLILSWKFISSWR

RLSVYWKRAELLDATLGPPDTTIQRRVIVVACFISICSIVEHLLSMFMAIGFDTPPMDYLHKYILNSHAFLIRPDTYSLWTAIPIFFISKIATIL

WNFQDLIIVLISMGLTSRYHRLNLYVNSLVKKENVDKEKRISTAKYVRNQKWRRVREAYVRQATLVRMVDAQIGALVLLSNINNFFFIC

LQLFLGLNKTGGSLMSYFYYFLSLGWLLFRACSVVLAAADVHIYSRRALEYIRLCPVSGYNVEIKRLNNQLSHDFVALSGMGFFWLSR

QTLLEVAGNIIKYELVLIQYDK

>OfurGR64a

FLPTLTHIFTVAQWVGIPTYGNKMSLVWAIIVLSMLTAIEAAAIWTLIKILTGVAKHVDDGRGLTARLSGSIFYGNGFLSLILSWKFISSWR

RLSVYWKRAELLDATLGPPDATIQRRVIIVACFISICSIVEHLLSMFMAIGFDTLPMDYLHKYILNSHAFLIRPDTYSLWTAIPIFFISKIATIL

WNFQDLIIILISMGLTSRYHRLNLYVNSLVKKENVDKEKRISTAKYVRNQKWRRVREAYVRQATLVRMVDAQIGALVLLSNINNFFFICL

QLFLGLNKTGGSLMSYFYYFLSLGWLLFRACSVVLAAADVHIYSRTALEYIRLCPDSGYNVEIKRLNNQLSHDFVALSGMGFFWLSRQ

TLLEVAGNIIKYELVLIQYDK

>PintGR64a

TPPQWKNKARETDVFLPTLTKIFKFSKWFGIPTYGNKIYFIWATIILLLLFTIVVASIVKVIRSLAGLAQHDSDGRGVTARLSGAIFYTNGL

LSQILSWRLIASWKRLSHHWLRVEITSLHLPQDQKIRRRVVIVTCFVAVCAMSEHMLSMMSATGFTIHPEEYFDRYMLSSHGFLFHTENY
SLWKVIPTFIMSKLATVFWNFQDLIIILISMGLTSRYNRLNFYVKHIVAKSRRNRSKTDSDMFLHIQIWRKLREAYVKQSSLVRTFDRHLG
PLVLLSNINNLYFICLQLFLGLNRGERGVVNRMYYFLSLSWLLFRACSVVLAAADVNLHSKRALMYLSQCPDTGYNLEVKRLKHQLTH
DKVVLSGMGLFSLDRQNLLGVAGNIVKYEMVLFQYDK

>GmelGR64a

TKEDDPSLRTITRIFTIAKWFGIPTYGNKIAFVWAIVLLIMLFAIEFAAIWKVIKSLAGLVDNKDERGVTARLSGAIFYCNGLLSLILSWRLI
SSWKWLSTRWEKIETTSTLHIECDRNIRRRIMFVTNLVGICACIEHMLSMMSATGFEIPPEKYFEKYILSSHGFLIDYDDYTLWLAIPVFM
LSKSATILWNFQDLIIILLSMGLTSRYHRLNVYVKNLVKRSRNEKKFVSEINTQMQEWRKLREEYVRQAALVRMVDKELGPLMLLSNIN
NFYFICLQLFLGINKGEKGIINRTYYFLSLGWLLFRACSVVLAAADVHLHSKKALSYLNVLPKSGYNVEIKRLKNQLAHDFVALTGMGL
FSLDRQKLLEVTANIIKYELVLIQFDK

>AgriGR64a

MSARENDLFLHTLTHVFTIAKWFGIPTYGNKLTFLWAIILLGMLVAIEVAAIWKVIISLAGLVDNKDERGVTARLSGAIFYCNGLLSLILS
WKLISSWKWMSKRWEKMETTSTLRIECDPKIRRRVIFVTNLVGLCACIEHMLSMMSATGFQIPPESYFKKYILSSHGFLLDQDDYTLWIA
IPVFILSKSATILWNFQDLIIILLSMGLTSRYHRLNLYVKNLVQRSRKENKFISEINSQMQEWRRLREEYVQQAALVRMVDKELGPLMLLS
NVNNFYFICLQLFLGINKGEKGLINRTYYFLSLGWLLFRACSVVLAAADVHLHSRKALRYLHDLSKSGHSIEIRRLKNQLTHDFVALTG
MGLFSLDRQKLLEVTANIIKYELVLIQFDK

>AtraGR64a

LTRISNKWKNNNEHSDIFLPTLTKIFKLTKWFGIPTYGNKIAYFWAIVILLLLLAIEVASLAKVIRSLAGLAKHDTDGRGVTARLSGAIFYA

NGLLSQILCWRLVSSWKILSQHWKKVENTSLYLPPDPRIGKRVLFVTCFVAVCAFVEHMLSMMSATGFSIPPEQYFERYILSSHGFLLNPE

SYSLWYAVPVFILSKQATILWNFQDLIIILISMGLTSRYHRLNLYVNRVVTEERRNGGSKKLGPDIFLRIQIWRSLREAYVRQSSLVRTFDG

HLGGLVLLSNINNFYFICLQLFLGLHRGGKGLVNRLYYFLSLGWLLFRACSVVLAAADIRLHSKRALLYLSQYPESAYNIEVKRLKHQL

ANDNVALSGMGLFTLDRQKLLEVAANIVKYELVLIQ

YDR

>McinGR64a

NEQEKYRKSEKLTRDKFLPTIEAVFKIATWFGIPTYGGNVAFSWALTTLAMLAAILTAAIWKLIRLLAGLSQNNQNGKNRVSERLSGSIFY

GNALLSLILSWIFVKKWRNISMKWLRLETHGLRLPPDMTIKRRVVLVSALISAFAFVEHILSMISATGVHCPVEEYLERYVLSSHGFLLRQ

YEYTIWLAIPIFIMSKTATILWNFQDTIIILISMGLTSRYYRMNSFVKRVVGNEKRAEKDKKLGSPYCFKINNWRKIRQAYVRQAALVRRI

DMDLGALVLLSTLNNLYFICLQLFLGIRHIDGTLINKIYYFYSLGWLLFRACSVVLSAADIKLHSKRALSYLYICPSSVYNIEIKRLQDQLS

NDKIFLSGMGFFSLDRQKLLQVAAMIVKYELILLQYD

>PsauGR8

NLVKTLNKKKCDDFLPVLNNVFLIARYFGISGFGLTLAFGWCLILFAMLVATNCVAIWRIVTLLGGWLKKSSDNGLIGRLSGAIFYANAL

MSLFLSSKFVHSWRKLSSYWLSMETNTALDFPPDVKIKKRTIFITAFVASIAVVEHALSMVSATGINFPPEEFFYRYVTISHGFLLRTQDYN

LWYAIPIFIVSKLATALWNFQDLIIILISMGLSSRYHRLNLYVSHVITIEKRFEVKKRFGTDLYLQIQVWRRLREAYVRQSTLVRMVDRRLG

SLVLLSNINNLYFICLQIYLGIHKSSGGTISRCYFLFSLGWLIFRACSVVLAASDVHLHSQRALKCLHSCPSASFNIEMKRLQYQLAHDFV

ALTGMGFFSLRRELLLEVAAAILKYELVLIQYDN

>CsasGR6

FLLTMTSILSHAKWFGVSVKSCSVFFFWAIIVLILLTAIEVGAIWKVIKVWMGLDGRSDTLEGSLTIRLAGSIFYGNAFISLLLLWKLASSW

KMLSIYWAIAEVNSGLKLPSDSKLRKRIVTVTNFIAIFGVAEHFLSITGNINFELQTSGYLQQYILKSHAFLIKPNDYNIWLALSIFIVSNLA

TVLWNFQDLVIILISMGLSSRYYRINSYLYDTVKTERRLRNVEKGSTTIYKQQQTWRRLREAYVRQAALVRMIDRELGALVLLSQINNFY

FICLQLYNGLHRSTTSLSSHIYYMSSLGWLLFRAISVVLAAADVNISSKRALPYLYMCPRSAYNVEIKRLEQQLTHDFVALTGMGFFSLN

RKMLLEVAGNILKYELVLIQYD

>PglaGR10

FLPWMKKIFTYAKWIGIPVYSGKVALVWAFTLFILFFCLEVCAIWKLIRLLTGITVNSSAGRNLVARLSGSIFYANGLISLVLTWRFIGAWD

DIANYWLRNELTSGLNIAPDKFIKRKIVFVSTFVSVCACVEHLFSMIAAIGFQCCSLEECFRTYILRSHGFLLQPYEYDLWVAIPVFVLSKL

ATVLWNVQDLVIILISMGPTSRYNMLNEYTEKLIQREYQIKRNYNKLDTYFLVDRWRKLRRAYARQANLVRLLEKRLGPLVFLSNLNNL

YFICLQLYLGIRIKSRSWIGRTYYLISLAWLLSRACGVVLAASTILLRSKNSLPYLRSCSSSTYNIEIRRLINQLTYDTVCLSGMRLFSLSRQ

SLLEVVATVIKYELILLQFD

>PrapGR4

PEREDDFLPTVTCMFAFAKTFGIPTYGGKLALIMSVTILVMLIILEIFSIWKLVRTLNGWNQNPNGSVIARLSGSIFYGNGLLSMLITWKLA

RKWPSLSKVWLELETKCSLHFPPNAKIRRRLLFVVGYTVFCAFVEHVLSMMSSTGLDCPLQEYFERYILSSHGFLITKHEYNLWLAIPIFI

LSKQATVLWNFQDLIIILISMGLTSRYNRLNVFLRRLVMYENDMKEHNLQQNLVKRVYLWRRLRIAYVHQSQLVKRVDKCLGGLILLSN

LNNLYFICLQLFLGISQEKGTVINRIYYFYSLSWLMFRASGVLLAAADVHLHSQKALPALYRCSSAAYNIEIKRLKYQLQHQFISLSGMD

LFYLSRQKLLEVAGAIVKYELILLQYDK

>MsexGR4

DEFLPTIANIFKIARIFGISKYGFNLAFMWTLMIFITLISVEVTSFWKFVKVLDGWAEDKFARRGFTERVSGSVFYGNATLSLILSCKFVNS

WKRLSRRWRKVEIEGSLRFPPDRWIQWKVTAVSAFIGVCALMEHILSMMSAIGLHCAPSQYLRKYILNSHGFLLRINEYSLWFAIPIFILS

KISTMLWNFQDLMIIVISMGLTSRYNRLNMYVGHIIKIERKLSDSPKVRSDLHVHNEIWRRIRESYVRQAELVGMVDKEFGALILLSNIN

NLFFICLQLFLGLNATARGALINKLYYFISLGWMLFRACTVVLAASNVHMHSKKALVFLYSCPKSGFNIEV

>PxutGR10

FLPCIKKIFTYTKWTGIPVYGGKIAITWALVLFIMLCCLEAAAIWKLIRLLTGVAVYSSAHRSLVARLSGSIFYANGLISLILTWRFIGAWDC

LSQHWLRNDLLTGLNVESDKYIKRKIIFVTTFVSVCAIVEHIFSMIAAIGFNCSSVEECFRTYILISHGFLLQSYEYDLWLAIPIFILSKLATIL

WNLQDLTIILMSMGLTSRYNMLNLYTRKLIQREYQKKQNYSKLTYLHIDQWRKLRRAYARQANLVRSLERHLGPLVLLSNLNNLYFICL

QLYLGIRITSRSWISRTYYLLSLTWLLSRACGVVLAGAAIILYSKKSLPDLKSCLSATYNIEVKRLITQLTYDSVGLSGMKLFTLSRQNLLE

VVATVIKYELILLQYDK

>PpolGR10

FLPCIKKIFTFTKWTGIPVYGGRIATTWALLLFILLCCLEAAAIWKLIRLLTGVAVHSSAGRNLVARLSGSIFYANGLISLILTWRFIGSWDD

LSQHWLRNELSSGLNIASDKYIKRNVIIVTIFVSVCAIVEHIFSMIAAIGFRCCSLKECFKTYILISHGFLLQNYEYNLWLAIPIFVLSKLATV

LWNLQDLIIILMSMGLISRYDMLNIYTRMLVQREYQKTQNYNKLTYLHIDQWRKLRRAYARQANLVRELERHLGPLVLLSNLNNLYFIC

LQLYLGIRIMTRSWISRTYYLLSLTWLLSRACGVVLAAAAIILHSKKALPCLRSCLSAAYNVEVKRLITQLTYDNVCLTGMQLFSLSRQSL

LEVVATVIKYELILLQFDK

>PmemGR10

FLPFIKKMFKATKWTGVPVYGGKIATAWALLIFILLSCLEAAAIWKLIRLLTGVAVHSSAGRNLVARLSGSIFYGNGLISLILTWRFIGSWD

DLSQHWIRNELSSGLNISSDKYIKRKIIIVTTFVSVCAIVEHIFSMIAAIGFRCCSLKECFRTYILISHGFLLQSCEYNLWLAIPIFILSKLATVL

WNLQDLIIILMSIGLISRYNMLNIYTRKLIQRECKKTQNYSKLTYLHIDQWRKLRRAYARQANLVRALERHLGPLVLLSNLNNLYFICLQL

YLGIRIMTRSWISRTYYLLSLTWLLSRACGVVLAAATIILYSKKALPNLRSCLSATYNVEVKRLITQLTYDNVCLSGMQLFSLSRQSLLEV

VATVIKYELILLQFDK

>CsinGR14

PHLPKTDEFLTTMNSLFDALRFLAIPVRGGYFVLACSVIHFGLLAAIETGAIYKVIKVLAGLTLYSTGNRSVVARVSGSIFYGNAAVSLVL

MFRLRRSWTDISSTWAHTERCWGLIGPSRDKRLRPKMIFVIIFMAIGTVAEHVMSMISNVGLDCPWSEFLERYILNSHAFILIQSDYNSRL

GVALFVVNKTATIIWNLQDLIIILISMGLSSRYRRLNLCVQELEGQLSSRRRKSDYEEYIQQQTWRRIREAYVRQAALVRSVDKELGPLLL

LSNSNNFYFICLQLFLGIGGQKSNIIDKSYVLVSIVWLLLRACTVVLTAANINIESKVALKSLYSCPKECFNIEVQRLQNQLSRDSVALSGM

GFFYLDRNMLLDVASAIFRYELVLLEFDK

>PmacGR10

FLPCIKALFTYTKWTGIPVYGGKIATIWALILFILLCCLEAAAIWKLIRILSGVAVYSSASRNLVARLSGTIFYANSLISLVLTWRFIAAWDDL

SQHWLKNELLSGLNTTKDKYIKRKIIFVTTFVSVCAIVEHIFSMMAAIGFHCGSLEGCLRTYILTSHGFVLQSYEYNLWFAIPIFILSKLATI

LWNLQDLAIILISMGIVSRYSMLNLYTKKLIQREYQKKQNYSKLSYQHIDQWRKLRRSYARQANLVRLLEKHLGPLVLLSNLNNLYFICL
QLYLGIRIMSRSWISRTYYLLSLTWLLSRACGVVLAAAAIILYSKKALPDLRSCSSATYNIEVKRLIIQLTYDNVGLSGMKLFSLSRQSLLE
VVATVIKYELILLQYDK
>PrapGR7
HDTFLQFMNTIFKWARCFGVPGFGCMLWQIWAAVVLTSLALTEGGAIWKVIRALAGWARDTANHRSVTARFAGAMFYAASLISQILCW
RLSYNWRALSKYWASVEWVLNIKYVPPDRNLKKRLMAVTALMATGATVEHLMSILATTGIDCPISDILKTYVLKSHGFLLLDQEYYKM
IAVPVLFVSNIATILWNFQDILIVLISMGLTSRYHRLNMCVATVCSELNKERKWSKDNEILQVYLWRKIREAYVKQALLVRKINSAFGLILL
FSNFFGFYFICLQLFLGITQGFTGDIFQKMYSLVSLAWICVRTCCVVLAAADIYENSKRALPYLYTCRAQFYNIEIERLQDQLDKDNIALS
GLGFFNLTRTVLLQVASSVITYVLVLVQYDN
>AdisGR8
VKKLNQKKCDDFLPILNRVFHMARFFGVSGAGVSLAFGWCLIMFALLVGTIGVAIWRVATLRSASNGIIARLAGAIFYSNGLMSLYLSSK
FVHSWRTLSSYWLSMETNTALAFPPDVKIKKRTIFITAFVAVMAVVEHVLSMVSATGFGFPIEEFFYRYVTLSHGFLMKTEEYTLWYAIPI
FIVSKIGTILWNFQDLIIILISMGLSSRYHRLNLYVSKVVTIEKRLEDKSRFGTELYLQMQVWRRLREAYVRQSTLVRMIDRHLGSLVLLS
NINNLYFICLQIYLGIHKQSGGTIKRLYFLFSLSWLVFRACSVVLAASDVHLHSRRALKCLHSCPSAAYNV
>PmacGR6
IQQPPRDEFLTLMQQIFRCARWFGIAGTSKYYWKLWGIVLLLCLTVVEIGSIWKVVKALAGWAVSTTGHRSVTARLAGATLYTNAVLSLI
LTWRISSSWGKIADYWIIIERAIIARIPRDEYLDKKMIAVGLFFIICGLAEHTMSVLAVVGLDCPPSHLLKRYILVSHGFLLQPNDYSTSVA

MSLIILSTVATILWNFQDMLLVLITLGLASRYRRINSYVASYCTKNEKETDFKNLDTVEIFTWRKIREAYVKQATLVRKVNDKVGPLILLS
NSCNFYFTCLQLFLGITQGMTDTGLTQMYYLSSFIWLCLRTTLVVLSAADVNVSSKGALPYIYEYPTKEYNLEIERLQYQLSKDYVALSG
MGFFFLTKSLLLQMAGAVVTYELVLIQFD

>AdisGR7

EQPYHDEFLTAMERVFKWTCLIGVLGSKKYICYVWSAFILIVLLFMEIEAIWKVIKALAGWAIDTAGQRSVTARLAGTFFYTIAILSLVLA
SKLYRSWGDLSALWARVERIMAVKAPPDRSLKRRMYTVVCFMTVCSLLEHIMSMISSVGLDCPPSLIIKRYVLISHGFMILRHEYSDWY
ALPLMLMSIVATLLWNLQDQLIVLISMGLTSRYHRLNQCLAKMCALEKKKVDSEIFQKNEATKVYTWRKLREAYVKQAMLVRKVDEA
IGGIIILSCFCNFYFICLQLFLGITQSRSSEPIKTAYYFLSLGWLCFRVTSVVLPASDINTHSRLALSHIYTHDTHYYNVEVGRLQDQLSKDY
VALSGNGFFYLSKSILLQMAGAIITYELVLIQFD

PBP:

>LstiPBP1

MTYPKMWTSKILVMMVAACVMTVMVDSSQSVMTSMTKNFIKAYEACAKEYNLPASTGQELINFWKEGYTVTSREAGCAILCLSSKLD
LLDPEGKLHHGNTVEFAKQHGSDDAMAHKVVEILHSCEKAAAPNEDMCLVALDVS

>LstiPBP2

MVPEAMKQLTHGFLKVLEECKTEMNLSEGVISDLYHLWREEYDQISRDAGCVIHCMSKKLQLFGDDGKIHHENIKEFAVKNGAEEKVA

AQLLSLAHECEKKQEGIEDECERTLEVAKCFRRDVKQIDWVPKMEVLMTEVIEA

>LstiPBP3

ILDFAHKQDLLHYEQYRLHHQNAYQFAKDHGADEATAKQIMTIVHECEEKFATNEDHCARAMEVSRCFRDHMHRLQWAPSVDVLVGE

ILVEMA-

>CpinPBP4

MAATFKWRLVSILILGLAVNVPVDASQEVMKKMSATFFKLLEECKKELSVADDMIQGLVRFWLEDSELGERELGCVIICMAEKQDLVL

MEDYRMHHENAYNFAKNHGADDAMATAVVKVIHTCEEQFTSNPDHCARVMEVSKCFRDEIHRLKWAPSIELLIGEMIGEA

>CmedPBP5

MDGNVKWRLAAILVLILAANVDRVKSSQEVMKKMSTTFFKLLDECKKELSVSDDLIQGLVRFWREDADLGARELGCVIMCIASKQDL

VILEDYKMHHENAYNFARDHGADDETAKAIVKIVHDCEKNFDSNPDHCSRVMEVAKCFRDEIHKLKWAPSVEVLIGELMSEA

>MvitPBP3

MVEIVKWRLATILVFCLLANGKASQEVMTKMSATFFKLLEECKKEASVTDDLIQGLVKFWNEDSELGARELGCVIICMATKHDLVDAE

EFRMHHENAYNFAKDHGADDEMAKSVVKSIHGCEEQFVGNPDHCARVMDVTRCFRGEMHRLKWAPPVEVLMGEMLAEV

>OnubPBP4

MADATKWRIAAILVICFAVNLNTVMSSEEVMTKMGVTFFNVLEECKKELKVTTNINEGLVRFWSQGAAPERELGCVFLCMAHKKDLL

EDQKRLHHENAHQFAKGHGADDDKATEIVSLLRECEQQFITITDDCSRALEVARCFQAHMQRLQWAPSMEVMVEEILAGMA

>OnubPBP-D

MADATKWRVAAILVICFAVKLNTVMSSEEVMTKMGVTFFNVLEECKKELKVTTNINEGLVRFWSQSAAPERELGCVFLCMAHKKDLL

EDQKRIHHENAHQFARGHGADDDKATEIVSLLRECEQQFITITDDCSRALEVARCFQAHMQRLQWAPSMEVMVEEILAGMA

>OfurPBP4

MADATKWRVAAILVICFTVNLNTVMSSEELMTKMGVTFFNVLEECKKELKVTTNINEGLVRFWSQGAAPERELGCVFLCMAHKKDLL

EDQKRIHHENAHQFARGHGAEDDKATEIVSLLRECEQQFITITDDCLRALEVARCFQAHMQRLQWAPSMEVMVEEILAGMA

>OfurPBP-D

MADATKWRVAAILVICFTVNLNTVMSSEELMTKMGVTFFNVLEECKKELKVTTNINEGLVRFWRQGAAPERELGCVFLCMAHKKDLL

EDQKRIHHENAHQFARGHGAEDDKATEIVSLLRECEQQFITITDDCFRALEVARCFQAHMQRLQWAPSMEVMVEEILAGMA

>MvitPBP1

MELVVKIALMLVAIAGVESSQNVMHKMTVNFAKALEYCKKELNLPDVMNQEFYNFWKEEHELTNRLVGCALICMSTKLDLVDPEGN

MHHGNAQEYAKKHGADDALAKQLVDLVHGCEKSAAKGDDECERALNIARCFKAEIHKLKWAPDPEVVLAEILSEV

>CpinPBP1

MGMLVKLLLVIVASVGVQCSQDILKKMTVNFGKALEACKKEIDLPDSVNAELYNFWKEDYQLTNRQAGCAMICMSTKLDLVDPDGN

MHHGNAHEYAKKHGADDATAKQLVDMLHTCEKSVGKMDDNCERALGIARCFKAEIHKLKWAPDPEVVLAEILAEV

>GmelPBP2

MAGAVFVGLLLAAISVRDVEPSREVLRYITTGFLKTLDDCKHELNLSDHIITDLYHYWKQEYDMLDKDVGCVILCMSKKLNLVDTSGR
LHHGSAKDFAVQHGAAESVADKLVEMLHECEKQSLTIEDSCVRTLEVAKCFRTNIRDLDWSPKVDVIVSEILTVV

>BmorPBP3
MARYNIVVAVLVLGVVGARGSSEAMRHIATGFIRVLDECKQELGLTDHILTDMYHFWKLDYSMMTRETGCAIICMSKKLDLIDGDGKL
HHGNAQAYALKHGAATEVAAKLVEVIHGCEKLHESIDDQCSRVLEVAKCFRTGVHELHWAPKLDVIVGEVMTEI

GOBP:
>LstiGOBP2
MSSAWILLGLVMAAVSSVRGTAEVMSHVTAHFGKALEECRTESGLSPEILEEFQHFWSEDFEVVHRELGCAIICMSNKFSLLQDDTRIHH
VNMHDYVKSFPNGEVLSEKMVSLLHNCETQYNDMTDDCDRTVKVAACFKADAKKEGIAPEVAMIEAVMEKY
>OnubGOBP2
MCSPWVLLGLVMAAVSGARGTAEVMSHVTAHFGKALEECRTESGLSPEILEEFQHFWSEDFEVVHRELGCALICMSNKFSLLQDDTRI
HHVNMHDYVKGFPNGEVLSEKMVKLIHNCEKQFDDITDDCQRTVKVAACFKVDAKKEGIAPEVAMIEAVMERY
>OfurGOBP2
MCSPWVLLGLVMAAVSGARGTAEVMSHVTAHFGKALEECRTESGLSPEILEEFQHFWSEDFEVVHRELGCALICMSNKLSLLQDDTRI
HHVNMHDYVKGFPNGEVLSEKMVNLLHNCEKQFDDITDDCQRTVKVAACFKVDAKKEGIAPEVAMIEAVMERY

>CmedGOBP2

MLSSWVLLGLMMAAVASVKGTAEVMSHVTAHFGKALEECREESGLSPEILEEFKHFWNEDFEVVHRELGCALICMSNKFSLLQEDTRI

HHVNMHDYVKSFPNGEVLSDKMVELLHNCEKQYDAITDDCDRTVKVAACFKKDCQKEGIAPEITMIEAVIERY

>MvitGOBP2

MLSMWYFAGLVMAAVSSVRGTAEVMSHVTAHFGKALEECREESGLSPEILEEFKHFWSEDFEVVHRELGCALICMSNKFSLLQEDTRI

HHDNMHDYVKSFPNGEVLSAKMVELLHNCEKQFDSMTDDCDRTVKVAACFKNDCKKEGIAPEITMIEAVMEKY

>HvitGOBP2

MLSMWYFAGLVLAAVSSVIGTAEVMSHVTAHFGKALEECRDESGLSPEVLEEFKHFWSEDFDVVHRELGCALICMSNKFSLLQDDTRI

HHVNMHDYVKSFPNGEVLSARMVELLHNCEKKFDDVTDDCSRTVKVAACFKVDAKKEGIAPEVAMIEAVMEKY

>CpunGOBP2

MLPIWLYFGLVMAAVSSVKSTAEVMSHVTAHFGKALDECRDESGLSPEILEEFKHFWSEDFEVVHRELGCALICMSNKFSLLQEDTRIH

HINMHDYVKSFPNGEVLSAKMVELLHNCEKQYDAITDDCDRTVKVAACFKNDCKKEGIAPEITMIEAVMERY

>CpinGOBP2

MLPIWLYFGLVMAAVSSVKSTAEVMSHVTAHFGKALDECRDESGLSPEILEEFKHFWSEDFEVVHRELGCALICMSNKFSLLQEDTRIH

HINMHDYVKSFPNGEVLSAKMVELLHNCEKQFEAITDDCDRTVKVAACFKNDCKKEGIAPEITMIEAVMERY

>TniGOBP2

MTSKCCLLLVVMATVAGSVMGTAEVMSHVTAHFGKALEECRDESGLSPEILEEFQHFWREDFEVVHRELGCAIICMSNKFSLLQDDSR

MHHVNMHDYVKSFPNGHMLSETLVTLIHNCEKQYDSLTDDCDRVVKVAACFKVDAKKEGIAPEVAMIEAVMEKY

>SlitGOBP2

MTSKCCLLLVVMAAVTSSVMGTAEVMSHVTAHFGKALEECREESGLSAEVLEEFQHFWREDFEVVHRELGCAIICMSNKFSLLQDDSR

MHHVNMHDYVKSFPNGHVLSEKLVGLIHNCEKQFDSMTDDCERVVKVAACFKVDAKAAGIAPEVAMIEAVMEKY

>HzeaGOBP2

MTSKSCLLLVAMATLTASVMGTAEVMSHVTAHFGKALEECREESGLSAEVLEEFQHFWREDFEVVHRELGCAIICMSNKFSLLQDDSR

MHHVNMHDYVKSFPNGHVLSEKLVELIHNCEKKYDTMTDDCDRVVKVAACFKVDAKAAGIAPEVAMIEAVMEKY

>HassGOBP2

MTSKSCLLLVAMATLTASVMGTAEVMSHVTAHFGKALEECREESGLSAEVLEEFQHFWREDFEVVHRELGCAIICMSNKFSLLQDDSR

MHHVNMHDYIKSFPNGHVLSEKLVELIHNCEKKYDTMTDDCDRVVKVAACFKVDAKAAGIAPEVAMIEAVMEKY

>HarmGOBP2

MTSKSCLLLVAMATLTGSVIGTAEVMSHVTAHFGKALEECREESGLSAEVLEEFQHFWREDFEVVHRELGCAIICMSNKFSLLQDDSRM

HHVNMHDYVKSFPNGHVLSEKLVELIHNCEKKYDTMTDDCDRVVKVAACFKVDAKAAGIAPEVAMIEAVMEKY

OBP:

>LstiOBP1

MAKFLLLALTFGLAHAAMEGPWKTVAIAADRVDKIERGGELRIYCRSLTCEKECKEMKVTFYVLENGQCSLTTITGYLQEDGKTYKTQ

YQGDNHYELVKETPENLVFYSENVDRADRKTKLIFVLGNKPLTSEENERLVKYAVSSHIPPENIRHVLGTDTCPE

>LstiOBP2

MLKVALTAAILALSLGGSYSSPAQDKSAKPKDDSTAKDMMMDQGDMTSGEAVKNRVDLNEIMNQCNDSFRTEMAYIEALNESGSFPD

ETDRTPKCYVRCVLEHSGVASEDGVFDAARAAEVFAGERGGRTMTDLQDLAAACAD

>LstiOBP3

MLRVSIFLVTLCVGYLSAEKPVVYLVPEKIVQTLPQVMECIAETGVDFEVLNKLRTGVSADSKDPRIAKFAHCGMKKNGLAKKNGRPEI

DKLMAFYPSSADKAAIRKVMEECDKEGKNPVDTSYKFAQCFEKNAPVKVMF

>LstiOBP4

MLLVILAKFLVILASCEAMTMKQIKNTGKMMRKSCQPKNNAADEKIDPLNEGIFIDEKEVKCYMACIMKMANTIKNGKPNYEAAIKQI

DLLLPEEIKAPAKEAVTACKKVPDAYKDTCDAAFHVSKCIYEHNPSIFFFP

>LstiOBP5

MAKFTILCLGVLAAAISSARALTPEELTKIEGDMLVHVQDCAKKFDVDESDLKKAKEEENIDGVDPCLIGCVFKNIKLVNDKGLYDPDV

AIESSKSYLSDDEDKAKFAEIAKDCASVNDE

>LstiOBP6

MECIGDNPITEQDINDLRAKKAPSGPGGPCFLACIQRKIGVMDEHGMMQNENALELAKKVFQDEEELKIIADYLHSCKSVNDVSVSDG

EKGCERAMAAFKCMITNAPAFGIEV

>LstiOBP7

MFCRVILLSSVYFLALTPYSINAMTEAQKEMIKQHFEQLGMECIGDNPITEQDINDLRAKQAPSGPGGPCFLACIMRKIGVMDEHGMMQ

NENALELAKKVFQDEEE

>LstiOBP8

VDSKGIPREAELWGKVQSSVTSQQSRAALRDQIRACFQELQSDAEDNGCSYSNKLERCLMLRFSDRLKADRSKTQANNQKT-

>HvitOBP3

ANQRQRRSDDYPRIDNNENTGNQYSHERRNSTDMREQIYVLNATDYDYGAGKGGEKLVSTVPRPCTGNRNYTVEMNINRTKRSEPLL

NKPDMDQCLSQCIFANLQVVDSRGIPRETELWNKVQASVTSQQSRAALRDQIRACFQELQSEAEDNGCSYSNKLEKCLMLRFSDR

>SlitOBP1

MKEGNRYSHERRITNDSGDQLMVINATDDDYSGYGSGNMGEKLLTSVPRPATPSNNINKNNTSRTKRNEPLLNRPDSDQCLSQCVFANL

QVVDSRGIPREAELWNKVQSSVTSQQSRSALHDQIQACFQELQSEAEDNGCSYSNKLERCLMLRFSDRKVDGKGNAKKSSTEQTG

>OfurOBP5

MFRIATLLVLLCVGYLTAEKPVVHLDSEKMVEILPQVIQCVAETGVNIEALEKLRLGVPGEIKDPNLPKFAHCAFLKTGYSHENGRAKVD

KVLKLFPAGDYKAALKKHVQECDKDGKDPVDTTYQFLKCLYTKSPVIVRF

>GcaeOBP6

MHRVTILLVILSVGFLSAEKPTIHLVPEKMGDLMQTLMSCLLESPVDPEVILQLRNGGTPKNIDEKSLQKFTYCSFIKSGYGKKNGHVKV

DKALELYPKDVDKEQIKKVMEECNKEEGKEPAETAFKFMKCFREKSPVRAIL

>CsupOBP1

MRCCAVLFVLAFIGCIYAEQEIVHLPPEKVAQILPVAMQCVGESSVPPEVIFQYASGKSLGNDKKYQKFIHCVFTKTGYADETGHINIDKA

MEVFPKGTDKEAVKKIMEECSKERGEDPPETSFKFAKCFRKKAPVRIAL

>CpinOBP11

MQRVAIILVVWCVGFISAEKPAVRLDKQKTLDIMQSLIQCVAETNVNPEVLIKMRNGDTFPDKVDSNVHKFVNCAFVKTGYGKKNGHV

KAAKMVDLFPDNVKEQMKAVVEKCDSKDGKDPNETTYLFFKCFTEESPVHVVLE

>DglaOBP19

PEKLAVIMPKIMECFVDHPIDTELLTKLRAGKDIPATVDKNFHDFLYCAIVKTGYAKKNGRVHVEKFVELEPKVVDKEVIRKVIVECDKD

GKDPTDTAVTFFKCYRDKNPVTLTLI

>OfurOBP12

MLLTQLAKFLVVLATCEAMTLKQIKNTGKMMRKSCQPKNNAADEKIDPLNEGIFIDEKEVKCYMACIMKMANTIKNGKPNYEAAIKQ

VDLLLPEDMKEPAKEALAACRKVPDAYKDTCDAAFHVTKCIYNHNPSIFFFP

>HvitOBP2

MLLISLAKFLVILAICEAMTMKQIKNTGKMMRKSCQPKNNVADEKIDPLSEGVFIDEKEVKCYMACIMKMANTMKNGKPSYEAAIKQI

DLLLPEDLKEPAKAAVTACKKVPDDYKDTCDAAFHVTKCIYNHNPSIFFFP

>CmedOBP2

MLLVILAKFLMVLAMCNAMTMKQIKNTGKMMRKSCQPKNNVADEKIDPLGDGVFIDEKEVKCYMACIMKMANTMKNGKPNYEAAI

KQVDLLLPEEIKQPAKEALAACKKVPDAHKDPCDAAFHVTKCIYNHNPSIFFFP

>CpunOBP8

MLLVLIAKFLMLLATCETMTMKQIRNTGKMMRKSCQPKNNATDEQLDPLNEGVFIDEKEVKCYMACIMRMANTMKNGEPNYDAAV

KQADLLLPEEMKQPAKEALFACKKVPDDYKDPCDAAFHVTKCIFNHNPSIFFFP

>SlitOBP8

MLLTKIVKFFILVATCEAMTMKQIKNTGKMMRKTCQPKNNAEDEKIDPISDGVFIDEKEVKCYMACIMKMANTIKNGKLNYDAAMKQ

ADLLFPDDIKEPAKEAITACRKVADAHKDICDASFHVTKCIYNHNPGIFYFP

>GmelOBP2

MILIAAAKFLLLLAFCEAMTMKQIKNTGKMMRKQCQPKNNVEDEKIDPINDGVFIDEKEVKCYMACIMKMANAIKNGKLNFEAALKQ

ADLLLPEEIKQPAKDAIVACRKVPDAYKDICDAVFHVTKCIYNQNPAIFYFP

>CpinOBP8

MTMKQIKNTGKMMRKSCQPKNNATDAQLDPLNEGIFIDEKEVKCYMACIMKMANTMKNGKPNYDAAVKQADLLLPEELKQPAKDA

LFACKKVPDDYKDPCDAAFHVTKCIFNHNPSIFFFP

>HvitOBP9

MSQIENCIFSKMFRSAVFLCCFYFMALTPDSTKAMTAEQKAKIKEHFEMLGMECINDNPITEEDINDLRSKKLPSGDGVPCFLSCIMKKIG

VMDNAGMLQKESVLDLAKKVFNDEEELKNIEDYLHSCSHINSEAVSDGEKGCERAFLAYKCMNENASKFDIEV

>LstiOBP6

MECIGDNPITEQDINDLRAKKAPSGPGGPCFLACIQRKIGVMDEHGMMQNENALELAKKVFQDEEELKIIADYLHSCKSVNDVSVSDG

EKGCERAMAAFKCMITNAPAFGIEV

>CmedOBP13

MYRAVTLCGLFFMALTPYFANAMSEEQKARIREHFETIGMQCITDNPITEQDINDLRGKKPPSGAGAPCFLACVMRNIGVMDDAGMLQ

KESVLELAKKVFEDEEELKIIADYLHSCSSVNSASVGDGAKGCERAMLAYTCMIENAPKFGIDV

>GmelOBP13

MYRSTILFCCLCLFNFMHSTIAITDEQRAQIHQHFEMIGIECIKDHTVTEDDITNLRTRKLPSGAGAPCFLACMMRKIGVLDDQGLMQKE

NALELARKVFEDEEEVQKISDYMHSCAHVNTASVSDGEKGCERAFLAYKCMMENAPQFGFDI

>LstiOBP5

MAKFTILCLGVLAAAISSARALTPEELTKIEGDMLVHVQDCAKKFDVDESDLKKAKEEENIDGVDPCLIGCVFKNIKLVNDKGLYDPDV

AIESSKSYLSDDEDKAKFAEIAKDCASVNDE

>OfurOBP3

MAKFLVLCLGVLAAAISSTKALTSEELLKIEADMLVYVKDCADKFSVSDDDLKEAKEKENVDNISPCFLACVFKNANLINDKGLYDPEV

VTGKLSDKYLSNDEDKAKMAEIAKDCTKVNDESVSDGAEGCERAKHLLVCFAKHKNALKGGR

>CmedOBP26

MMKFNLLLTFGVLAAVLSNARALTKEELETIQKDMLVHVKACSEKFGVSIDEIKAAKEKHDIEGMDPCLIGCVFKSTKLVNDEGMYDP

DVAIEDSNKYLSSDDDKAKFKDIANDCAKVNDEDVSDGKEGCERSKLLLGCFAKHKSELMPEARRR

>CpinOBP16

MMKLNIFVSVAVLAAVLGNARALTKEELGVIENDMIAHVKKCGEQFGVSDEEIKAAKEKKDIGGFDPCLIGCVFKSTKLINDEGVFDPK

VALEHSEKYLSSDDDKAKFKDIADDCAKVNDESVSDGKEGCERAKLLLSCLAKHKDELHPSRR

>GmelOBP11

MNSFLCLVILGIAVSNVTALTNDELDKIHESMVVFIKECSKEFGVSDDELKAAKESGNVDGVEPCLIGCVFKKANFIDGDGKFLPDKVKE

NAKKYLSQEEDQTLFGSVVDTCAAANDEEVTDGDAGCVRSKLVFECFIKHKGDVSTYLYFKQKIKQKCS

>SexiOBP6

MSKFTCLVLCVVAASLSGVHATAEEKAAFIEAVKPYIQECSKEHGVTPEDIKSAKEAGNADGINACFLRCVYNKAGVINDKGEYDADK

ALEKLKKFVSNEDDYAKFAEIGKKCASVNEKSVSDGEAGCERAALLTSCFLEHKSEVHA

>HarmOBP5

MSKFTCLVLCVVAASLSQAYASEEEKAAFREAIKPIVEECSKEHGVSHDELKSAKDNQNADSIKPCFLGCVYKKAEVFNSKGEYDVDK

ALEKLKKFVSNDEAYAKFAEVGKKCASVNDKAVSDGDAGCERGALLTACFLEHKAEVPL

>GmelOBP7

MNSFLSLAVLTIALSYVTALTQEELDKVREPMLNFIKECSKDYGVSDDELKAIKESGKVDGVEPCLIGCVFKKAGFIDGDAKFLPDKIKE

LSKKYLSNEEDIAKFGTIVDTCAAAIDEQVTGGDPACIRSKLVLECYAKHREELIKVTN

>SlitOBP6

MSKFTCLVLCVVAVSLSGVHATAEEKAAFIEAVKPYVQECSKEHGVTPEDIKSAKAAGNADGINSCFLSCVYKKAEVITEKGEYDADKA

LEKLKKFVSNEDDYAKFANIGKKCASVNEKSVSDGEAGCERAALLTSCFLEHKSEISA

>SexiOBP26

MSKFTCIILCVVAASLTKVSHAVTEEEKEAFREAMAPIIAECSEEHGVSEADIKAAKEAASADGIKPCFLGCVMKKIEVLDSKGLYDAET

GLGKLKKFVKDDDEFAKFEDIAKKCLKVNDESVSDGEAGCDRAKLVLGCFLEHKVEMPF

>OfurOBP4

MLRVALVVSIFVLSLGGLYCTPVQTSTKPDDSSAKHDDRSAKPVDSSAKPDDSSVKTKEVMLSSGQDMSSSEDANNTVDLMTFMNQC

NESFRTDMAYIDALMESGSFPDETDRTPKCYVRCVLEHVGVASEEGAFDAARASEVFAGERGGRAMTDVQDLAEACADRNESCKCER

SYRFMRCLMEAEIKEYSSN

>CpinOBP17

MSRVVSVTLLALLALSIGPSVEASTGTSGSSAATSGTTSHDRSTDDVKGKLITPADDQGNMTSAEERALDVPDLMAVMVECNDSFRIEM

GYLESLNESGSFPDEIDRTPKCYVRCVLEKTGVASEDGLFDPAQAAAVFAGERNGVLMTNLEDLATRCAADRNEKCRCERSYNFIKCL

MEAEIKEYVSN

>GmelOBP14

SRVLLLVVLAVQSFVFTELASVSKTGSDVKETDRDDYKNMTSTGLTTVFTELRSSETELDQAMNECNETFRVEMSYLESLNESGSFPDE

TDRTPKCYVRCVLEKTGVASEDGNYDPARTAVIFAGKRAGRPMNDIEEIATGCANRKESCKCERAYQYIKCMMETEIRKYE

>BmorOBP7

MANLVLLLTFVLMTLSMARLKSTEAPKSKTALFNDQDNMGYEELDMEEIMSACNESFRIEYAYLESLNDSGSFPDETDKTPKCYIRCVL

EKTEILSENGVLNPATAALVFAGERNGKPMSDLEEMAVACADRHEKCKCEKAYNFVKCLMYMEIDKYEKKN

>BmorOBP84a

MANPVLLLTFVLMTXSMARLKSTEAPKSKTALFNDQDNMGYEELDMEEIMSACNESFRIEYAYLESLNDSGSFPDETDKTPKCYIRCVL

EKTEILSENGVLNPATAALVFAGERNGKPMSDLEEMAVACADRHEKCKCEKAYNFVKCLMYMEIDKYEKKN

CSP:

>LstiCSP1

MWFKLIFLATYVSVVVTDMGPPGFQRTFSEGVTSKGYRVVYGDEDLTVINEVVGSMDKDDILKAKVHLNEGIKPLPAEDVKCLMSVD

RYCSMEMRKVKGILIQALKDDCEKCSIPEKESAGKVAASMMAHDPVGWKLFLTRYDGLSKVQRILG

>LstiCSP2

MNFLEDHHLQKIFIMRAIVLLSCLVMVYAADKYSSKYDNFDVETLISNDRLLKAYINCFLEKGRCTPEGADFRKALPEAVETTCAKCTE

KQKNNIRKVIRAIQQKHPKQWDELVNKTDPSGKHRADFDKFIQGSS

>LstiCSP3

MKSLVLVALSLLVAVAWARPGATYTDKWDHINVDEILESQRLLRGYVDCLLDKGRCTPDGKALKETLPDALEHDCSKCTAKQKESSEK

VIRHLINKQPDFWKELSTKYDPENIYQEKYKDKIEEVKSKN

>LstiCSP4

MKTIVALCALMAVALARPEETYSDTWDNFNAQELVDNVRLLKNYGKCFLDQGPCTSEGQDFKKRIPEALKTDCGKCSPKQRELIRTVV

KGFQAKLPEVWAELVKKHDPEGTYKDSFEAFLNSN

>LstiCSP5

MKFIVVLAVIVGLAMADEKYTSENDNFDVDALVANIDELKKFSGCFLDINDCDAVAADFKKDIPEAFQQACAKCTDAQKHIFKKFIAGL

KEKLPHDYEAFMKKYDPESKYYPALEKVINV

>LstiCSP6

MQIALVLVMLAACAYAAETPRPQVSDTALEDALNDKRFIQRQLKCALGEAPCDPIGKRLKTLAPLVLRGACPQCSPQETKQIQRTLSYV

QRNYPQQWAKIVRQYAG

>LstiCSP7

MKTIVALCALMAVALARPEETYSDTWDNFNAQELVDNVRLLKNYGKCFLDQGPCTSEGQDFKKRIPEALKTDCGKCSPKQREL

>LstiCSP8

ELKEHLQEAIETGCEKCTEAQEKGAYTAIEYLIKNELDIWRQLAAKFDPEGKRKTYEDRARANGIVIPE-

>LstiCSP9

MEMRKVKGILIQALKDDCEKCSIPEKESAGKVAASMMAHDPVGWKLFLTRYDGLSKVQRILG

>LstiCSP10

ETQRTGTRRVIGHLINNEPEYWAQLTAKYDPQHKYVVKYEDELRIAKAQN-

>LstiCSP11

MQTACAKCTDKQKVGARRVVNHIREKEQEYWEELLSKYDPKGEYKSIYEPFLAGKE

>LstiCSP12

DLIATNCGKCTPEQKKRHEEVNKFILEKYPTEYNAVVNKYRPKAE-

>LstiCSP13

ELKEHLQEAIETGCEKCTEAQEKGAYTAIEYLIKNELDIWRQLAAKFDPEGKWRKTYEDRARANGIVIPE-

>HassCSP24

MKLTVAVALLCLVAESWAASTYTDKWDNINVDEILESQRLLKAYVDCLLDRGRCTPDGKALKETLPDALENECSKCTDKQKSGSDKVI

RHLVNKRPEMWKELSAKYDPNNIYQDRYKDKIEAVKGQ

>HassCSP20

MKILVLLLAAVVTAQYEEDTYGTDHDDLNIAAVVEDKEQFNSFVDCFIDEAPCDEVADTFKSVIPEAVLEACAKCTPAQKHIVRVFNESF

KKKMPEKFQKFKNKYDPEGKYFDNFEAAVAAF

>HassCSP25

MNSAIVLCVVALAGMVLARPDGGTYTTKYDNVDLDEILANDRLLVPYIKCLLDEGKCAPDAKELKEHIKEALENGCAKCTDKQKEGT

RRVIAHLIKKKLQEWEKLKAKYDPEGKYAKKYEKELEEVKNA

>BmorCSP16

MIEWKRFKILHFLSYLGLLVLVVVCAAQQNRPQVTDTALDEALNDKRFIQRQLKCALGEAPCDPIGKRLKTLAPLVLRGACPQCSPQET

KQIQKTLSYVQRNFPQHWAKLVRQYAG

>BmorCSP12

MGDHTQRPVNLDPQKMFMLFIISFIIVPVLKCCGTETSTYTTQYDEVDIKEIMGNERLLVAYIGCLLDKNPCTPEGKELKRNIPDALQSDC

SKCSDKQRENADAWIEFMIDNRPEDWTKLEERYNPDGSYRTKYLEGKHNATSNVDESK

>HarmCSP26

MKSLLILCLVIAAVWARPETYDTRYDDFDAETLVENVRLLKAYGHCFLGTGPCTPEGSDFKKTIPDALRTGCGKCTAKQRHLIRVVVQG

FRSKTPDLWQQLVKKEDPNGQYKEVFTRFLNGSD

>HarmCSP15

RPESQYTNKYDNVNLDEILVNKRLLVPYIKCALDQGKCSPDGRELKSHIREALENYCAKCTPVQQDGTRRVIAHLINHEPDYWRQLSV

KYDRDGKFAVKYEKELRTIA

>HarmCSP16

MKILVLLLAAVVTAQYEEDTYGTDHDDLDIVALVEDKDQFNSFIDCFIDEAPCDDVAETFKSVIPEAVLEVCAKCTPAQKHIVRVFNESFK

KKMPEKFQKFKNKYDPEGKYFENFEAAVGAF

>HarmCSP12

MNSAIVLCVVALAGMVLARPDGDGDKYTSKWDNIDLDEILGNDRLLVPYIKCALDEGKCAPDAKELKEHILEALETGCDKCTDKQKE

GTHRVIAHLIKYKLEEWEKLRAKYDPEGKYAKKYEKELEELKRA

>HarmCSP20

MELNMKTLVLLLLAAVVTAQYEEDTYGTDHDDLDIVALVEDKDQFNSFVDCFIDEAPCDDVAETFKSVIPEAVLEACAKCTPAQKRIVR

VFNESFKKKMPEKFQKFKNKYDPEEKYFDNFEAAVAAF

>HarmCSP17

ASTYTDKWDNINVDEILESQRLLKAYVDCLLDRGRCTPDGKALKETLPDALENECSKCTDKQKSGSDKVIRHLVNKRPEMWKELSAK

YDPNNIYQDRYKDKIEAVKGQ

>HassCSP18

RPDTYTDKYDNVDLDEVLSNRRLLVPYVHCLLEQGKCAPDAKELKEHIREALENACGKCTDAQQSGTRRVIGHLINKEPEFWKQLNA

KYDPNNKYTKKYEKELKEVQEDKQNH

>HassCSP21

MNSAIVLCVVALAGMVLARPDGGTYTTKYDNVDLDEILANDHLLVPYIKCLLDEGKCAPDAKELKEHIKEALENGCAKCTDKQKEGT

RRVIAHLIKHKNADWQKLKAKYDPEGKYTHKYEKELEEVQH

>HassCSP22

MKVLIVAVLALVAPSALGYDEKYDKLDVDKIIGDDALFTAYTDCMLDKGPCTVEHSEDFKKLLPEVIQTACAKCSGIQKTNVRKTVKAL

SDKKPDDFAKFRAKFDPKGEYEKDFSAFMLATD

>HassCSP23

MKLLIVLALVAVVAARPDDAFYDKKYDDFNVDEIIENVRLLKAYAHCIIGDGKCTPEGNDFKRWVPEATKSSCGKCTEKQKVLVAKTIK

AIQEKCPEEYTTLVKQIDPENKYAEDLKNYLAKYGH

>HarmCSP13

MKVLLVLCLFAAAALADDKYTDKYDNINLDEILENKRLLLAYVNCVMERGKCSPEGKELKEHLQDAIETGRSKCTEAQEKGAYKVIEH

LIKNELDIWRELAAKYDPKGDWRKKYEDRARANGIQIPE

>HarmCSP24

KYDNVDLDEILENDHLLIPYIKCTLDEGKCAPDAKELKEHIQEALETGCAKCTDKQKEGTRRVIAHLIKHKNADWQKLKKYDPEGKYA

KKYEKELEEVKNA

>HarmCSP9

MNSLIVFCVLSLAALTIARPDGATYTDKYDNVDLDEILGNRRLMVPYIKCMLDQGKCAPDAKELKEHIKEALENECGKCTEAQKKGTR

RVIGHLINHEADFWNELAAKYDPERKYTTKYEKELKEVEA

>HarmCSP21

MNSAIVLCVVALAGMVLARPDGDGDKYTSRWDDVDLDEILENDHLLIPYIKCSLDEGKCAPDAKELKEHIQEALETGCAKCTDKQKE

GTRRVIAHLIKHKNADWQKLKAKYDPEGKYTHKYEKELEEVQH

>HarmCSP3

MNADWFLIFTLITVVSSDFYNSKYNCFNVQPLLENDRILLSYTKCFLDQGPCTPDAKDFNKVIPEALETTCGKCSPKQKLVIKTVIKAVIS

RHPDAWDQLTEKYDKDKKYKDSFDKFLA

>HarmCSP23

MKLLIVLALAAVVAARPDDEFYDKKYDDFNVDEIIENVRLLKAYAHCIIGDGKCTPEGNDFKRWVPEATKSSCGKCTEKQKVLVAKTIK

AIKEKCPEEYTTLIKQLDPENKYADDLKNYLAKYGH

>HarmCSP8

MKCIYVLSFLLALAAVQAEDKYSTENDNLDIDAVVANVDTLTSFVACFVDQEPCDAVAADFKKDIQEAVTTRCAKCTDAQKHIFYKFIL

GLKEELPRGYEEFGRKYDPENKHFSALENAVSPA

>BmorCSP7

MKGFYVLCFALFAAVYCKETYSSENDDLDIEALVGNIDSLKAFIGCFLETSPCDAVSGDFKKDIPEAVAEACGKCTPAQKHLFKRFLEVV

KDKLPQEYEAFKTKYDPQGKHFDALLSAVANS

>BmorCSP15

MIENFYSKCTISKSVLFLCLIFLPYALNQKYYDSRYDYYDIDHLVQNPRLLKKYLDCFLGKGPCTPIGRLFKQVMPEVITTACAKCTPTQ

KRFARKTFNAFRRYFPETLMELRRKFDPESKYYDAFEKVITNA

>HarmCSP6

MKADCFLFVTLIAVVAADFYNSKYDSFDVQPLLENDRILLSYTKCFLDQGPCTPDAKDFKKVIPEALETTCGKCSPKQKQLIKTVIKAVI

SRHPDAWDQLTEKYDKDQKYKESFDKFLAEQD

>HarmCSP22

MKVLMVAVLALVAPSALGYDEKYDKLDVDKIIGDDALFTAYIDCMLDKGPCTVEHSEDFKKLLPEVIQTACAKCSGIQRTNVRKTVKA

LSDKKPDDFAKFRAKFDPKGEYEKDFSAFMLGTD

>CpunCSP5

MRAIVLLSCLVVVYAADKYSTKYDNFDVDTLISNDRLLKAYINCFLEKGRCTPEGADFRKALPEAVETTCAKCTEKQKNNIRKVIRAIQ

QKHPKQWEELVKKTDPSGKHRADFDKFIQSN

>CpunCSP6

MKTFVALFALVAVALARPQETYNTNYDNFDVKQLVENPRVLKNYGKCFLDQGPCTPEGSDFKKTIPEALKTECAKCTPKQRELIRTVVA

AFQSKLPEVWAELVQKHDPQGTYKKSFDSFLHASN

>CpunCSP8

MKIVILTLCLATAILAQEKYDAVDDDFDISEVLQNDRLLNSYAKCLLDKGPCTPEVKKVKDKLPEALATRCAKCTDKQKQIGKKLAQEV

KNKRPELWKELVAHYDPDGKYQDAFQDYLKP

>CpunCSP7

MKYDDFDIQPLLDNDRILTGYTKCFLDQGPCTPEAKDFKKVIPEALESSCGKCTPKQKQLIKTVIKAMMERHPDFWTELVDKYDKDKK

YRENFNKFIESDDK

>CpunCSP2

MKTIILVGLTIVVAVAWARPQSTYTDKWDHINVDEILESQRLLRGYVDCLLDKGRCTPDGKALKETLPDALENDCSKCTPKQKEASDKV

IRYLINKQPEYWKELSVKYDPDNIYQEKYKDKIQEVKATA

>BmorCSP6

MKSLIVLSCLLAACLAADLSKYENFDVEPIVTSDRLLKAYINCFLDKGRCTPEASDFKKALPDTIATNCGKCTEKQKANVRKVIKVIQQ

KHSTEWEKLVKKHDPSGKHRADFDKFLLGS

>BmorCSP9

MRAVIFLYTCVFVVVGQDINAMMSMPKYDERYDYLDVDDIFRNKRLVRNYVDCLINAQRCTPEGKALKRILPEALRTKCIRCTERQKR

TSVKVIRRLKNEYPEEWAKLASRWDPTGDFTRYFEDYLAKEHFNTIPGSGPTVNVLSLQTTPPPPPPPPSRPASVFTNPPPPVMSTSPRPV

VLNRFRR

>CpunCSP1

MISTKYLIVLCCVAAAVARPSDKYTDKYDNLNIQEILENKRLLKAYVDCVMGRGKCSPEGKELKEHLQEAIETGCEKCTEAQEKGAYTA

IEYLIKNELDIWRELSAHFDPTGKWRKKYEDRARANGIEIPE

>CpunCSP4

MKIITLLLITIAYLTTQANAEETSTYSTKYDGVNLDEVLGNDRLLNNYVNCLLDNGPCTPDAKELKKNLPDAIQNDCKKCSDRQREGAV

QVMEYIIDHREEDWKKLEQKYNSDGSYKKKYLERKQAKEASKESNEQSNEKVNKSDEKKE

>BmorCSP1

ADDKYTDKYDKINLQEILENKRLLESYMDCVLGKGKCTPEGKELKDHLQEALETGCEKCTEAQEKGAETSIEYLIKNELEIWKELTAHF

DPDGKWRKKYEDRAKAKGIVIPE

>BmorCSP4

MKVLIVLSCVLVAVLADDKYTDKYDKINLQEILENKRLLESYMDCVLGKGKCTPEGKELKDHLQEALETGCEKCTEAQEKGAETSIDY

LIKNELEIWKELTAHFDPDGKWRKKYEDRAKAKGIVIPE

>BmorCSP3

MNSLIAFCLFAVLAVALARPDDKYTDRYDNVNLDEVLSNSRLLQPYIKCILDKDRCAPDAKELKEHIREALETECAKCTEAQKKGTRRV

IGHLINNESKSWNELTAKYDPENKFTAKYEKELREIKA

>BmorCSP8

MKTILILCALVSVVVCRPEEYYSSQYDNFDVEQLVGNLRLLKNYAKCFLDQGPCTAEGTEFKKRIPEALRTKCAKCNPKQRHLIRTVVK

AFQTKLPDLWEELAIKEDPKGQYKHEFTAFINAMD

>BmorCSP11

MKLTSFLLVGMAMVSAEFYSSRYDDFDVKPLVENDRILQSYTNCFLDKGPCTPDAKEFKKVIPEALETTCGKCSPKQKQLIKTVIKAVIE

RHPEAWEELVNKYDKDRKFRPSFDKFINEDD

>BmorCSP10

MDNQNNYKNINTGKSRPVTTRPTMKILIIVVMACVAVTWARPESTYTDKWDNINVDEILESNRLLKGYVDCLLGKGRCTPDGKALKET

LPDALEHECVKCTGKQKSGADKVIRHLVNKRPDLWKELAVKYDPDNIYQARYKDKIDAVKGSA

>BmorCSP14

MACVAVTWARPESTYTDTWDNINVDEILESNRLLKGYVDCLLGKGRCTPDGKALKETLPDALEHECVKCTGKQKSGADKVIRHLVNK

RPDLWKELAVKYDPDNIYQARYKDKIDAVKGSA

>BmorCSP5

MNSLIAFCLFAVLAVALARPDDKYTDRYDNVNLDEVLSNSRLLKPYIKCILDKDRCAPDAKELKEHIREALETECAKCTEAQKKGTRRV

IGHLINNESKSWNELTAKYDPENKFTAKYEKELREIKA

>BmorCSP2

QDKYEPIDDSFDASEVLSNERLLKSYTKCLLNQGPCTAELRKIKDKIPEALETHCAKCTDKQKQMAKQLAQGIKKTHPELWDEFITFYD

SQGKYQTSFKDFLES

>BmorCSP13

MKLLLVFLGLFLAVLAQDKYEPIDDSFDASEVLSNERLLKSYTKCLLNQGPCTAELKKIKDKIPEALETHCAKCTDKQKQMAKQLAQGI

KKTHPELWDEFITFYDPQGKYQTSFKDFLES

>HvitCSP18

MQIAPIVAVIALACACAAQTQTPRPQVSDTALEDALSDKRFIQRQLKCALGEAPCDPIGKRLKTLAPLVLRGACPQCSPQETKQIQRTLSY

VQRNYPQQWAKI

>CpinCSP10

MQITLVVLLIAAACAYAAETPRPEVTDTALEDALNDKRFIQRQLKCALGEAPCDPIGKRLKTLAPLVLRGACPQCTPQETKQIQRTLSYV

QRNYPQQWAKI

>OfurCSP17

MQTTLVLLLVVAACAYAAEAPRPQVTDTALEDALNDKRFIQRQLKCALGEAPCDPIGKRLKTLAPLVLRGACPQCSPQETKQIQRTLSY

VQRNYPQQWAKI

>DpunCSP2

RIVFACVVLAVCAAEQTQRPQVSDTALDDALSDKRFIQRQLKCALGEGPCDPIGKRLKTLAPLVLRGACPQCSPQETKQIQKTLSYVQR

NYPQQWAKI

>DpunCSP2

RIVFACVVLAVCAAEQTQRPQVSDTALDDALSDKRFIQRQLKCALGEGPCDPIGKRLKTLAPLVLRGACPQCSPQETKQIQKTLSYVQR

NYPQQWAKI

>EoblCSP14

MQIKYATILSALIAVCIAQSQRPQVSDTALEDALNDKRFIQRQLKCALGEAPCDPIGKRLKTLAPLVLRGACPQCSPQETKQIQKTLSYVQ

RNFPQQWAKI

>EoblCSP9

MQIKYATILSALIAVCLAQSQRPQVSDTALEDALNDKRFIQRQLKCALGEAPCDPIGKRLKTLAPLVLRGACPQCSPQETKQIQKTLSYV

QRNFPQQWAKI

>GmelCSP17

QAVVICAVLYACAAQNDRPQVTDTALDDALNDKRFIQRQLKCALGEGPCDPIGKRLKTLAPLVLRGACPQCTPQETKQIQRTLSYVQRN

YPQQWAKI

>AcinCSP7

MQIKFALVLCSLVTVCVAQAQRPQVSDTALEDALNDKRFIQRQLKCALGEAPCDPIGKRLKTLAPLVLRGSCPQCSPQETKQIQKTLSYV

QRNFPQQWAKL

>HchaCSP16

MQFSHILVICAVGVVCMAQTQRPEVSDTALDDALNDKRFIQRQLKCALGEGPCDPIGKRLKTLAPLVLRGACPQCTPQETQQIQRTLSY

VQRNYPQQWTKI

>AipsCSP5

MQIKYALLLCCVAAMSVAQTQRPAVSDTALEDALQDKRFIQRQLKCALGEAPCDPIGKRLKTLAPLVLRGACPQCTPQETKQIQRTLSY

VQRNFPQQWAKI

>LbotCSP9.2

MQMKLSLCVLACVCACVWAQQGRPPVSDTALEDALNDKRFIQRQLKCALGEAPCDPIGKRLKTLAPLVLRGACPQCSPQETKQIQRTL

SYVQRNFPAEWAKI

>MsepCSP13

MQIKYALLLCCVAAVSLAQTQRPAVSDTALDDALQDKRFIQRQLKCALGEAPCDPIGKRLKTLAPLVLRGACPQCTPQETKQIQRTLSYV

QRNFPQQWAKI

>SexiCSP20

MQIKYALVLCCVAAVSVAQSQRPPVSDTALDDALQDKRFIQRQLKCALGEGPCDPIGKRLKTLAPLVLRGACPQCTPQETKQIQRTLSYV

QRNYPQQWAKI

>GmolCSP5

MKLSLLVVAGVLACASAQARPPVTDTALEDALNDKRFIQRQLKCALGEAPCDPIGKRLKTLAPLVLRGACPQCTPQETKQIQRTLSYVQ

RNFPAEWAKI

>CpomCSP14

MQMKLSLLVVASVLACVCAQARPPVSDTALEDALNDKRFIQRQLKCALGEAPCDPVGKRLKTLAPLVLRGACPQCTPQETKQIQRTLS

YVQRNFPAEWAKI

>OfurCSP10

MKTIMLVAFLVGLAMADEKYTSENDNFDVEALVNNTEELQKFSGCFLDKNDCDAVSGDFKKDIPEAFQQACAKCTDAQKHLFKRFLN

GLKEKLPQDFEAFKKKYDPEDKFFAALDKAINA

>HvitCSP10

MKTIMLVAFLVGLAIADEKYTTEHDDFDVDTLATNPEELKKFSGCFLDKNECNEVAEHFKKDLPEVFAQACGKCTDAQKHLLKKFLDA

IKEKLPEDYEDFKKKFDPEGKYFGDVEKAIN

>CpinCSP9

LIALAIFVGLTAANDKYTTENDNFDVETLIANPEEFKKFTGCFLDTNPCNDISGDFKKNIAEAFQEACAKCTDAQKHLFQRYLATLKEKY

SQDLEAIKKKYDPEGKFFDALEKAVS

>SexiCSP4

MKCIYVLSVLLAFAAVQAEDKYSTENDDLDIEAVVADLDTLKGFVGCFMDAMTCHAVAADFKKDIPDAVATSCAKCTNAQKHIFHKFL

LGLKEKLPSDYEAFKKKFDPQGQYFEALEAAV

>AdisCSP6

MKCIYVLSLLLAFVAVQAEDKYSTENDDLDIDAVVANIDTLRGFVGCFMDTVTCHEVAADFKKDIPEAVETNCIKCTDAQKHIFHKFLL

GLKEKLPSDYEAFKNKFDPENKHFTALEAAV

>AperCSP7

MKYIYVLCLVVLASVYAKETYTTENDDLDIEALVKNLSALREFAGCFLDTVECNPVAGDFKKDIAEAVAQSCEKCTDSQKHIFNRFLEG

LKEKLPTEYEAFKKKYDADGKHFPALEAAV

>DpunCSP11

FLLLCLLAVGYSHATETYTTENDDLDIDRLVNNNDEFLSFIRCFLDMQECNSVAADFKKDLPEAVQTACLKCTQAQKHIFNKFLAGLKE

KLPKEYEEFSNKFDPQGIYFGPLEKAV

>GmolCSP1

MKFLIVLASLVLLAAGQDTYTPENDDLDIDALVSNPEALKAWFNCFVDKGQCDKVQTSFKDDLPEAIQQGCAKCTAAQKVILKKYLAG

LKEKAPADYEVLRQKYDPENKYFGPLEKAI

>EoblCSP6

MKFFYVFALSLVVACSGETYTTENDDLNIEAVVADLPTLQAFVGCFNDKVKCDEKSGDFKKDIAEAIQQACAKCTDAQKHIFKVFLTGL

KEKLPADYAAFKAKYDPENKYFGNLEKAI

>GmelCSP16

MKSFLVLCAVVALVIAEEKYTTNNDDFDIEALIVNVPELKNFNTCFVNDTNCNDVSSDFKRNLPEAVREACAKCTDVQKHIFRRYLEGL

QEKLPQQFEEFRKKFDPEGIYLEPLKAAL
